# Crystal structures of two dysprosium–aluminium–sodium [3.3.1] metallacryptates that form two-dimensional sheets

**DOI:** 10.1107/S2056989020010130

**Published:** 2020-07-31

**Authors:** Jordan R. Travis, Gerard P. Van Trieste III, Matthias Zeller, Curtis M. Zaleski

**Affiliations:** a Department of Chemistry and Biochemistry, Shippensburg University, Shippensburg, PA 17257, USA; b Department of Chemistry, Purdue University, West Lafayette, IN 47907, USA

**Keywords:** metallacrown, metallacryptate, aluminium complex, crystal structure

## Abstract

The two [3.3.1] metallacryptate complexes, namely, poly[[μ_3_-acetato-hexa­kis­(μ-*N*,*N*-di­methyl­formamide)­bis­(*N*,*N*-di­methyl­formamide)bis­[salicyl­hydroxi­mato(2−)]hepta­kis­[salicyl­hydrox­im­ato(3−)]hexa­aluminium(III)dysprosium(III)penta­sodium(I)] *N*,*N*-di­methyl­formamide tetra­solvate monohydrate], [DyAl_6_Na_5_(OAc)(Hshi)_2_(shi)_7_(DMF)_8_]·4DMF·H_2_O or {[DyAl_6_Na_5_(C_7_H_5_NO_3_)_2_(C_7_H_4_NO_3_)_7_(C_2_H_3_O_2_)(C_3_H_7_NO)_8_]·4C_3_H_7_NO·H_2_O}_*n*_, **1**, and poly[[di-μ_4_-acetato-nona­kis­(μ-*N*,*N*-di­methyl­form­amide)­octa­kis­(*N*,*N*-di­methyl­formamide)tetra­kis­[sali­cyl­hydroximato(2-)]tetra­deca­kis­[salicyl­hydroximato(3−)]dodeca­aluminium(III)didysprosium(III)deca­sodium(I)] *N*,*N*-di­methyl­form­amide 6.335-solvate], [DyAl_6_Na_5_(OAc)(Hshi)_2_(shi)_7_(DMF)_8.5_]_2_·6.335DMF or {[Dy_2_Al_12_Na_10_(C_7_H_5_NO_3_)_4_(C_7_H_4_NO_3_)_14_(C_2_H_3_O_2_)_2_(C_3_H_7_NO)_17_]·6.335C_3_H_7_NO}_*n*_, **2**, where shi^3−^ is salicyl­hydroximate and DMF is *N*,*N*-di­methyl­formamide, both consist of an aluminium-based metallacryptand. In **1** and **2**, the metallacryptand encapsulates a dysprosium(III) ion in the central cavity, and the resulting metallacryptates are connected to each other *via* sodium-DMF linkages to generate a two-dimensional sheet. The metallacryptates of **1** and **2** are the three-dimensional analogues of metallacrowns as the metallacryptates contain a metal–nitro­gen–oxygen cyclic repeat unit throughout the complexes. For **1** the building block of the two-dimensional sheet is comprised of only one type of metallacryptate, which is connected to four neighboring metallacryptates *via* four sodium–DMF linkages. In **2**, the building block is a dimeric unit of two metallacryptates. Each dimeric metallacryptate unit is connected to four other dimeric units *via* six sodium–DMF linkages. The two metallacryptates of each dimeric unit can be considered enanti­omers of each other. In both **1** and **2**, chirality is imparted to the metallacryptate due to the Λ and Δ propeller configurations of the four octa­hedral aluminium ions of the metallacryptand shell.

## Abstract 2

The two [3.3.1] metallacryptate complexes [DyAl_6_Na_5_(OAc)(Hshi)_2_(shi)_7_(DMF)_8_]·H_2_O·4DMF, **1**, and [DyAl_6_Na_5_(OAc)(Hshi)_2_(shi)_7_(DMF)_8.5_]_2_·6.335DMF, **2**, where shi^3−^ is salicyl­hydroximate and DMF is *N*,*N*-di­methyl­formamide, both consist of an aluminium-based metallacryptand. In **1** and **2**, the metallacryptand encapsulates a dysprosium(III) ion in the central cavity, and the resulting metallacryptates are connected to each other to generate a two-dimensional sheet.

## Chemical context   

**Figure 1:**
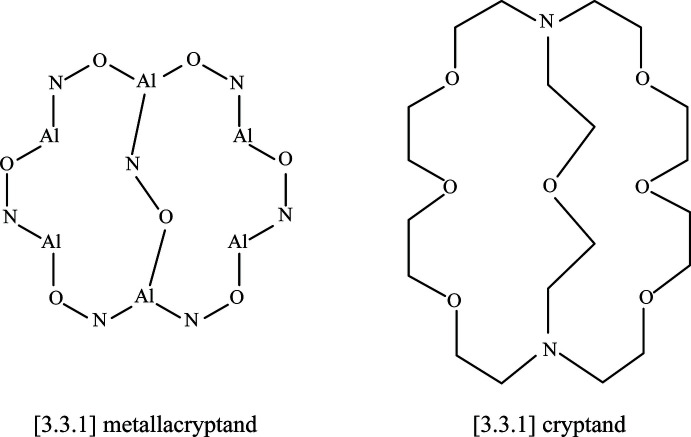
Schematic comparing the [3.3.1] metallacryptand of **1** and **2** to a [3.3.1] cryptand.

Metallacrowns, first recognized in 1989 by Pecoraro (Pecoraro, 1989 ▸), belong to a class of mol­ecules known as metalla­macrocycles that also include, but are not limited to, metalla­helices and metallahelicates (Kramer *et al.*, 1993 ▸; Piguet *et al.*, 1997 ▸), metallacryptands and metallacryptates (Ma *et al.*, 1980 ▸; Saalfrank *et al.*, 1988 ▸; Zaleski *et al.*, 2005 ▸), and metallic wheels and rings (Taft *et al.*, 1994 ▸; Müller *et al.*, 1995 ▸; Murrie *et al.*, 1999 ▸). The archetypal metallacrown (MC) framework is based on a cyclic metal–nitro­gen–oxygen repeat unit similar to the crown ether cyclic carbon–carbon–oxygen repeat unit. Since their inception metallacrowns have been considered not only structural analogues of crown ethers, but also functional analogues, as metallacrowns typically bind a metal ion in the central cavity. In addition, as cryptands can be considered the three-dimensional analogues of crown ethers (Lehn, 1978 ▸), metallacryptands with an *M*–N–O repeat unit (Fig. 1 ▸) can be considered the three-dimensional analogues of metallacrowns (Lutter *et al.*, 2018 ▸). Metallacrown and metallacryptate chemistry has mainly focused on incorporating transition metal ions in the ring repeating unit as these ions impart inter­esting magnetic and spectroscopic properties. The use of main-group metal ions has mainly been limited to gallium (Lah *et al.*, 1993 ▸; Jiang *et al.*, 2019 ▸; Athanasopoulou *et al.*, 2019 ▸), tellurium (Kübel *et al.*, 2010 ▸; Ho *et al.*, 2016 ▸; Ho *et al.*, 2017 ▸; Ho *et al.*, 2019 ▸; Wang *et al.*, 2019 ▸), and tin (Zhao *et al.*, 2010*a* ▸; Zhao *et al.*, 2010*b* ▸). Recently though, gallium-based metallacrowns and metallacryptates have gained renewed inter­est as potential optical imaging agents (Chow *et al.*, 2016 ▸; Nguyen *et al.*, 2018 ▸; Lutter *et al.*, 2018 ▸, 2019 ▸, 2020 ▸).
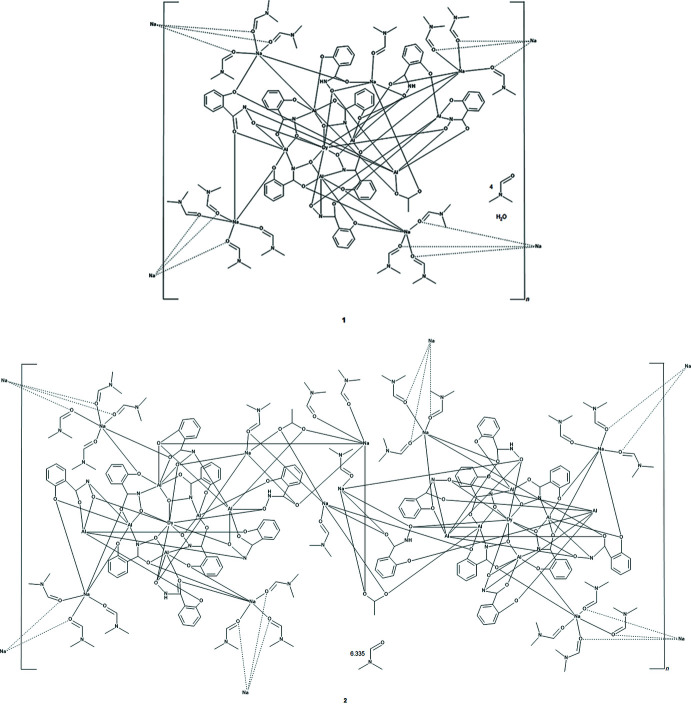
 
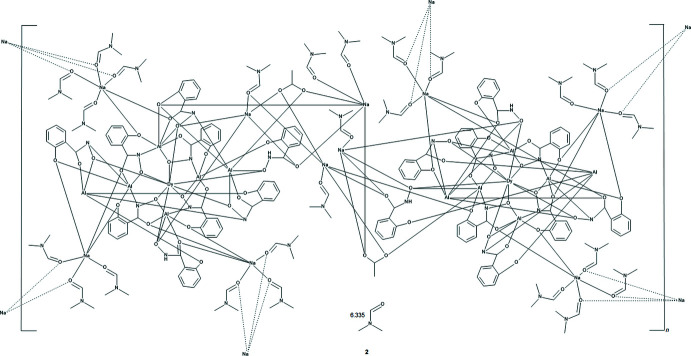


For many metallacrowns and metallacryptates, a common ligand is salicyl­hydroxamic acid (H_3_shi), which can be triply deprotonated to produce salicyl­hydroximate (shi^3−^), and when this ligand set is combined with transition-metal ions in a 3+ oxidation state, a plethora of structural arrangements is possible (Mezei *et al.*, 2007 ▸). In addition, one hallmark of MC chemistry is the ability to easily substitute components of the complexes and still generate similar structures. For instance, in a series of Ln^III^[12-MC_shi_-4] structures with ring manganese(III) ions, the Mn^III^ ions can be substituted with gallium(III) to generate comparable 12-MC-4 structures (Chow *et al.*, 2016 ▸). Thus, a logical extension would be to include aluminum(III) into the MC ring, as this would maintain the charge balance of the overall complexes. However, to date aluminum has not been used to generate either an archetypal metallacrown or metallacryptand complex. Herein we present two [3.3.1] metallacryptate complexes [DyAl_6_Na_5_(OAc)(Hshi)_2_(shi)_7_(DMF)_8_]·H_2_O·4DMF, **1**, and [DyAl_6_Na_5_(OAc)(Hshi)_2_(shi)_7_(DMF)_8.5_]_2_·6.335DMF, **2**, where DMF is *N*,*N*-di­methyl­formamide. Complexes **1** and **2** represent the first metallacrowns or metallacryptates to contain aluminum as a ring metal. Future studies will focus on the luminescent and magnetic properties of these and related compounds, as the aluminum ions do not quench the luminescence of the *Ln* ^III^ ions and the diamagnetic nature of the aluminum ions generates a potential single-ion magnet with the paramagnetic lanthanide ion at the center of the mol­ecule.

## Structural commentary   

**Figure 2:**
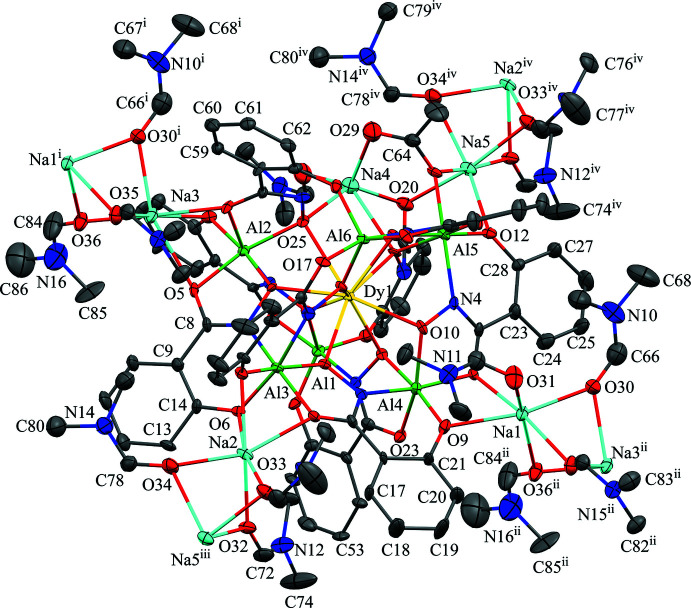
The single-crystal X-ray structure of [DyAl_6_Na_5_(OAc)(Hshi)_2_(shi)_7_(DMF)_8_]·H_2_O·4DMF, **1**, including connections to neighboring sodium ions [symmetry codes: (i) *x* − 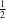, *y* − 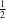, *z*; (ii) *x* + 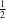, *y* + 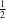, *z*; (iii) *x* − 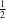, *y* + 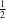, *z*; (iv) *x* + 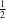, *y* − 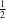, *z*]. The displacement ellipsoids are at the 50% probability level. For clarity, labels have only been added to the metal ions and some of the carbon, nitro­gen, and oxygen atoms. In addition, the solvent water and DMF mol­ecules, the hydrogen atoms, and disorder have been omitted. Color scheme: green – Al, yellow – Dy, light blue – Na, red – oxygen, dark blue – nitro­gen, and gray – carbon. All figures were generated with the program *Mercury* (Macrae *et al.*, 2020 ▸).

**Figure 3:**
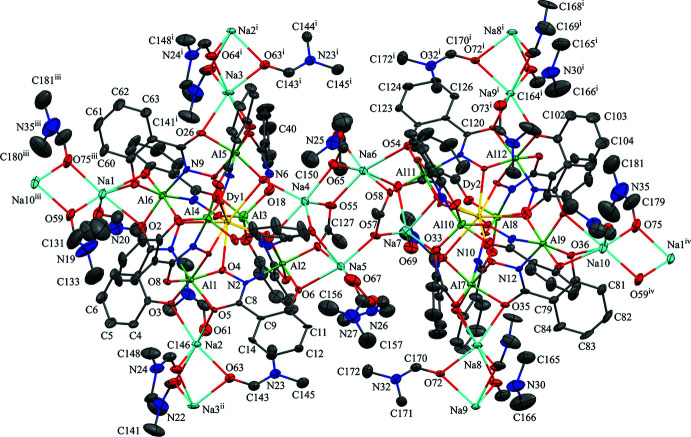
The single-crystal X-ray structure of [DyAl_6_Na_5_(OAc)(Hshi)_2_(shi)_7_(DMF)_8.5_]_2_·6.335DMF, **2**, including connections to neighboring sodium ions [symmetry codes: (i) *x*, *y* − 1, *z*; (ii) *x*, *y* + 1, *z*; (iii) *x* + 1, *y*, *z*; (iv) *x* − 1, *y*, *z*]. The displacement ellipsoids are at the 50% probability level. For clarity, labels have only been added to the metal ions and some of the carbon, nitro­gen, and oxygen atoms. In addition, the solvent DMF mol­ecules, the hydrogen atoms, and disorder have been omitted. See Fig. 2 ▸ for additional display details.

**Figure 4:**
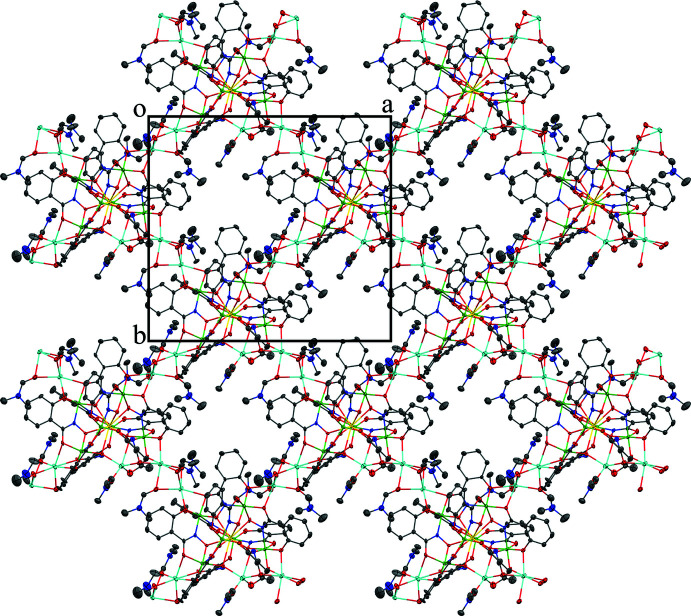
The two-dimensional network of **1** generated by the bridging Na^+^ ions between the metallacryptate units. Each metallacryptate is connected to four adjacent metallacryptates. The view is along the *c* axis of the unit cell. See Fig. 2 ▸ for additional display details.

The two-dimensional structures of the metallacryptates of **1** and **2** are very similar; however, there are key structural differences. Both compounds are synthesized in the same manner with a 1:16:16:32 ratio between the reactants, Dy(NO_3_)_3_: Al(OAc)_2_OH: H_3_shi: NaOAc; however, the crystals have different space groups (*Cc* for **1** and *Pc* for **2**). At this time it is not clear what factors induce the crystallization of **1** or **2**. In **1** there is only one type of metallacryptate in the two-dimensional network (Fig. 2 ▸), while in **2** there are two different but very similar metallacryptates. Compound **2** can be considered a dimeric unit of two metallacryptates (Fig. 3 ▸). When it is necessary to distinguish the two metallacryptates for **2**, the designations **2A** (associated with Dy1) and **2B** (associated with Dy2) will be used. Each metallacryptate is connected to its nearest neighbors to generate a two-dimensional sheet (Figs. 4 ▸ and 5 ▸), which will be described in greater detail below. The individual repeat unit of the two-dimensional network is akin to a metallacrown but can more accurately be described as a metallacryptate. As a cryptand is considered a three-dimensional analogue to a crown ether, the aluminum-based shells of **1** and **2** can be considered as three-dimensional metallacrowns, thus as metallacryptands (Fig. 6 ▸ *a*). Furthermore, upon binding a metal ion in the central cavity, a cryptand is transformed into a cryptate. Compounds **1** and **2** bind a Dy^III^ ion in the central cavity to produce a metallacryptate (Figs. 6 ▸ *b* and 7 ▸). Each metallacryptate unit consists of one Dy^III^ ion, six Al^III^ ions, and five Na^+^ ions to provide a total charge of 26+, which is counterbalanced by one acetate, seven triply deprotonated shi^3−^ ligands, and two Hshi^2−^ ligands with a total charge of 26-. For the Hshi^2−^ ligands, the phenolate and oxime oxygen atoms are deprotonated, while the oxime nitro­gen atom remains protonated and does not coordinate to any metal ions. Beyond overall mol­ecular charge considerations, the oxidation state assignments of the Dy^III^ and Al^III^ ions are also confirmed by bond-valence-sum (BVS) values (Table 1 ▸) (Brese & O’Keeffe, 1991 ▸; Trzesowska *et al.*, 2004 ▸). The Na^+^ ions do not participate in the metallacryptate structure, but serve to connect neighboring mol­ecules in a two-dimensional sheet.

The metallacryptand structure can best be described as a [3.3.1] complex based on the similar [3.3.1] cryptand (Krakowiak *et al.*, 1993 ▸), where the numbers indicate the number of oxygen atoms in each linkage of the metallacryptand (Fig. 1 ▸). In the metallacryptand, two Al^III^ ions serve as the anchor points of the structure, akin to the nitro­gen atoms of a cryptand (Fig. 1 ▸ & 6). There are two linkages between the anchor Al^III^ ions consisting of an O–N–Al–O–N–Al–O–N pattern and one shorter linkage with an N–O pattern, thus the [3.3.1] nomenclature. This metal–nitro­gen–oxygen repeating pattern is that of the archetypal metallacrown; thus, these structures can be considered three-dimensional metallacrowns. The repeating units of the metallacryptand strands are generated from the seven shi^3−^ ligands with the hydroximate group of the ligand providing the N–O linkage. The oxime oxygen atoms of the linkages bind a Dy^III^ ion in the central cavity to generate the metallacryptate. The two remaining Hshi^2−^ ligands are not involved in the metallacryptand structure but instead bind on the periphery of the structure and provide two additional oxime oxygen atoms to complete the coordination of the Dy^III^ ion and serve as bridging ligands to the Al^III^ ions; however, the Hshi^2−^ ligands do not provide an N–O repeat unit in the structure between the metal ions as the nitro­gen atoms remain protonated, thus negating their participation in the metallacryptand linkages.

Each Dy^III^ is nine-coordinate with nine oxime oxygen atoms providing the coordination sphere. As stated above, seven of the oxime oxygen atoms are provided by the seven shi^3−^ ligands, which participate in the formation of the metallacryptand and form bridges to all six Al^III^ ions. The two remaining oxime oxygen atoms are provided by the two Hshi^2−^ ligands. Each oxime oxygen atom of the Hshi^2−^ ligands also serves as a one atom μ_3_-bridge between the Dy^III^ ion, one Al^III^ ion, and one Na^+^ ion. A SHAPE (*SHAPE 2.1*; Llunell *et al.*, 2013 ▸) analysis of the Dy^III^ geometry (Table 2 ▸; Fig. 8 ▸) yields the lowest continuous shape measure (CShM) value for a spherical capped square anti­prism (1.212 for **1**, 1.305 for **2A**, and 1.225 for **2B**); however, comparable CShM values are obtained for a spherical tricapped trigonal prism (1.370 for **1**, 1.640 for **2A**, and 1.521 for **2B**) and muffin (1.728 for **1**, 1.813 for **2A**, and 1.803 for **2B**) geometries (Llunell *et al.*, 2013 ▸; Pinsky & Avnir, 1998 ▸; Cirera *et al.*, 2005 ▸). A muffin geometry can best be described as a configuration with a trigonal base, a penta­gonal equatorial plane, and a single point vertex (Ruiz-Martínez *et al.*, 2008 ▸).

There are six Al^III^ ions per metallacryptate; two of the Al^III^ ions are five coordinate while the remaining four are six coordinate (Figs. 9 ▸–11 ▸ ▸). For the five-coordinate Al^III^ ions, the geometries are either trigonal bipyramidal or spherical square pyramidal. For Al1 and Al6 of **1**, the CShM values (Table 3 ▸) slightly favor a trigonal–bipyramid geometry over a spherical square pyramid (1.533 *vs* 1.880 for Al1 and 1.220 *vs* 1.797 for Al6). For **2**, Al3 is clearly trigonal pyramidal with a CShM value below 1.0 (CShM = 0.874), where a value less than 1.0 typically indicates only minor distortions from the ideal shape (Cirera *et al.*, 2005 ▸). However, the remaining Al^III^ ions, Al4, Al8, and Al10, are spherical square pyramidal with CShM values of 1.168, 0.540, and 1.324, respectively, though for Al10 the CShM value for a trigonal bipyramid is 1.759, indicating the geometry is between the two ideal scenarios. For all of the five-coordinate Al^III^ ions of **1** and **2**, the coordination environment is composed of two chelate rings, a five-membered ring formed from the oxime oxygen and carboxyl­ate oxygen atoms of one shi^3−^ and a six-membered ring formed from the oxime nitro­gen and phenolate oxygen atoms of a second shi^3−^ ligand. The coordination is completed by a phenolate oxygen atom of a Hshi^2−^ ligand. For the six-coordinate Al^III^ ions (Al2–Al5 for **1** and Al1, Al2, Al5, Al6, Al7, Al9, Al11, Al12 for **2**) the geometries are clearly octa­hedral with all CShM values below 2.0 (Table 4 ▸). All of the six-coordinate Al^III^ ions adopt a propeller configuration with two Δ and two Λ stereoconfigurations per metallacryptate. For Al2 of **1**, the Δ stereoconfiguration is composed of three *cis* chelate rings: one five-membered chelate ring formed by the oxime and carboxyl­ate oxygen atoms of a shi^3−^ ligand, one five-membered chelate ring formed by the oxime and carboxyl­ate oxygen atoms of an Hshi^2−^ ligand, and a six-membered chelate ring formed from the oxime nitro­gen and phenolate oxygen atoms of another shi^3−^ ligand. For Al4, the Δ stereoconfiguration has three chelate rings: two five-membered chelate rings and one six-membered chelate ring from three different shi^3−^ ligands. For Al3, the Λ configuration consists of three *cis* chelate rings: one five-membered ring and two six-membered rings from three shi^3−^ ligands. For Al5, the Λ stereoconfiguration is formed by *cis* five- and six-membered chelate rings of two shi^3−^ ligands and two *cis* oxygen atoms, a μ-carboxyl­ate oxygen atom of an acetate anion and the oxime oxygen atom of an Hshi^2−^ ligand. The μ-carboxyl­ate atom of the acetate anion connects Al5 to Na5. For **2A**, the Δ and Λ Al^III^ ions (Δ: Al1 and Al5; Λ: Al2 and Al6) have the same coordination environment as their Al^III^ ions counterparts in **1**; however, for **2B**, the Δ and Λ environments are reversed relative to **1** and **2A** (Figs. 9 ▸–11 ▸ ▸). The Λ stereoconfiguration of Al7 has three *cis* chelate rings: one five-membered ring of a shi^3−^ ligand, one five-membered ring of an Hshi^2−^ ligand, and one six-membered ring of a shi^3−^ ligand. The Λ stereoconfiguration of Al12 has *cis* chelate rings from three shi^3−^ ligands (two five-membered rings and one six-membered ring), while the Δ stereoconfiguration of Al9 has *cis* chelate rings from three shi^3−^ ligands (one five-membered ring and two six-membered rings). For Al11, the Δ stereoconfiguration is completed by *cis* five- and six-membered chelate rings of two shi^3−^ ligands and two *cis* oxygen atoms, a μ-carboxyl­ate oxygen atom of an acetate anion and the oxime oxygen atom of an Hshi^2−^ ligand. Thus, the metallacryptand portions of **2A** and **2B** can be considered enanti­omers (Fig. 7 ▸).

The Na^+^ ions of **1** and **2** are either five- or six-coordinate. The SHAPE analysis indicates that the geometries are severely distorted in each case with the CShM values typically above 3.0, which can be considered an upper threshold value where below 3.0 is still considered an adequate description of the geometry though there are significant distortions (Cirera *et al.*, 2005 ▸). For the five-coordinate Na^+^ ions (Na4 of **1**, and Na4 and Na7 of **2**), Na4 of **1** can best be described as a spherical square pyramid, while for Na4 and Na7 of **2**, the geometry can best be described as trigonal bipyramidal (Table 5 ▸). The coordination environment of each Na^+^ ion is the same and consists of a five-membered chelate ring from the carboxyl­ate oxygen and oxime oxygen atoms of an Hshi^2−^ ligand, an oxime oxygen atom of a different Hshi^2−^ ligand, a carboxyl­ate oxygen atom of an acetate ion, and a carbonyl oxygen atom of a DMF mol­ecule. In addition to the Al^III^ propeller stereoconfigurations described above, the differences between **1** and **2** stem from the acetate anions and DMF mol­ecules of the five-coordinate Na^+^ ions. In **1**, the acetate anion forms a three-atom bridge to a neighboring Na^+^ ion (Na5) and the acetate anion does not connect to a neighboring metallacryptate. In **2**, the acetate anions not only form a three-atom bridge to a neighboring Na^+^ of the same metallacryptate (Na4 to Na5 of **2A** and Na7 to Na6 of **2B**), but also form μ-oxygen bridges to an Na^+^ ion of a neighboring metallacryptate unit (Na4 to Na6 and Na7 to Na5). In addition, the DMF mol­ecules of Na4 of **1** and Na7 of **2B** bind in a terminal fashion; however, for Na4 of **2A** the carbonyl oxygen atom of the DMF forms a μ-bridge between Na4 and Na6, which contributes to the linkage between **2A** and **2B**. Overall the connections between Na4, Na5, Na6, and Na7 of **2** connect the two metallacryptates to form a dimeric unit.

For the six-coordinate Na^+^ ions, the geometries can best be described as severely distorted octa­hedra with most CShM values above 3.0 and an average value of 3.591 for **1** and 4.039 for **2** (Table 6 ▸). For both **1** and **2**, the six-coordinate Na^+^ ions serve as connection points between neighboring metallacryptate units and this connectivity generates the two-dimensional sheet. A description of the coordination environment of Na1–Na3 and Na5 of **1** and the connectivity to neighboring metallacryptates follows. For Na1, the coordination environment consists of three μ-carbonyl oxygen atoms of three different bridging DMF mol­ecules, a carbonyl oxygen atom of a terminal DMF mol­ecule, a phenolate oxygen atom of a shi^3−^ ligand, and a carboxyl­ate oxygen atom of a different shi^3−^ ligand. The three bridging DMF mol­ecules connect Na1 to the Na3 ion of a neighboring metallacryptate. For Na2, the coordination environment consists of three μ-carbonyl oxygen atoms of bridging DMF mol­ecules, two phenolate oxygen atoms of two different shi^3−^ ligands, and a carboxyl­ate oxygen atom of a third shi^3−^ ligand. The three bridging DMF mol­ecules connect Na2 to the Na5 ion of a neighboring metallacryptate. For Na3, the coordination environment consists of three μ-carbonyl oxygen atoms of bridging DMF mol­ecules, two carboxyl­ate oxygen atoms of two different shi^3−^ ligands, and a phenolate oxygen atom of a third shi^3−^ ligand. The three bridging DMF mol­ecules connect Na3 to the Na1 ion of a neighboring metallacryptate. For Na5, the coordination environment consists of three μ-carbonyl oxygen atoms of bridging DMF mol­ecules, a carboxyl­ate oxygen atom of an acetate anion that bridges between Na5 and Al5, a phenolate oxygen atom of a shi^3−^ ligand, and a carboxyl­ate oxygen atom of an Hshi^2−^ ligand. The three bridging DMF mol­ecules connect Na5 to the Na2 ion of a neighboring metallacryptate. The series of μ-bridging DMF mol­ecules between the peripheral Na^+^ ions of the metallacryptates generate the two-dimensional network of the compound (Fig. 4 ▸). If the shape of the metallacryptate of **1** is approximated as a square, then the Na^+^ ions Na1, Na2, Na3, and Na5 lie at each corner. Each Na^+^ ion connects to one adjacent MC *via* three μ-carbonyl oxygens of the bridging DMF mol­ecules. Thus, each metallacryptate unit is connected to four adjacent metallacryptates *via* the peripheral Na–DMF–Na connections and a two-dimensional sheet of metallacryptates is generated (Na1 to an adjacent Na3, Na2 to an adjacent Na5, Na3 to an adjacent Na1, and Na5 to an adjacent Na2).

**Figure 5:**
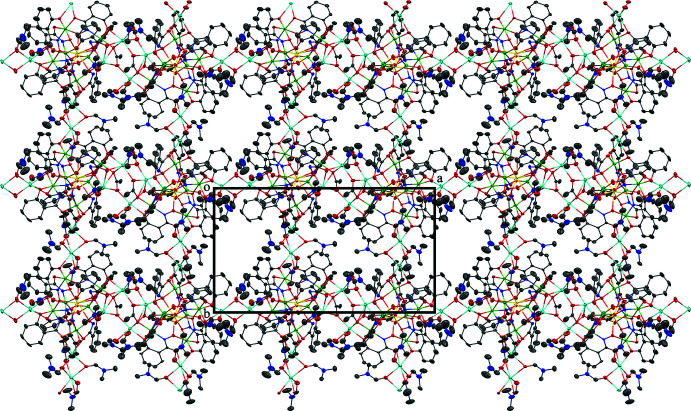
The two-dimensional network of **2** generated by the bridging Na^+^ ions between the dimeric metallacryptate units. Each metallacryptate is connected to four adjacent dimeric metallacryptates. The view is along the *c* axis of the unit cell. See Fig. 2 ▸ for additional display details.

**Figure 6:**
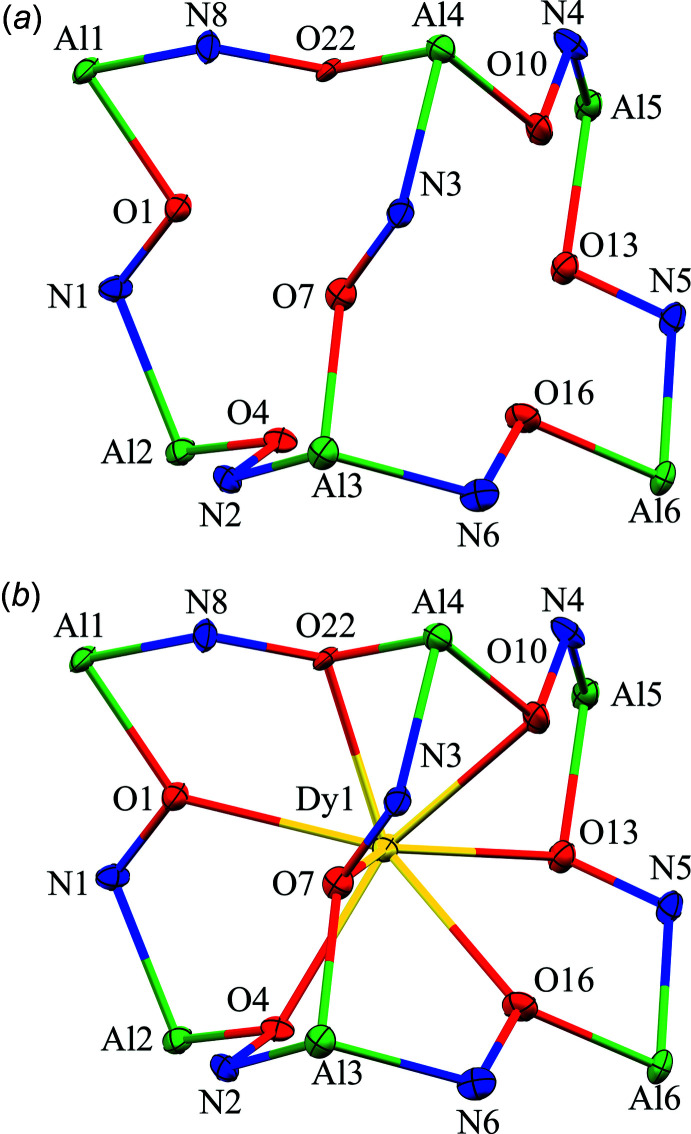
The (*a*) metallacryptand and (*b*) metallacryptate views of **1** highlighting the [3.3.1] connectivity between the metal ions. See Fig. 2 ▸ for additional display details.

**Figure 7:**
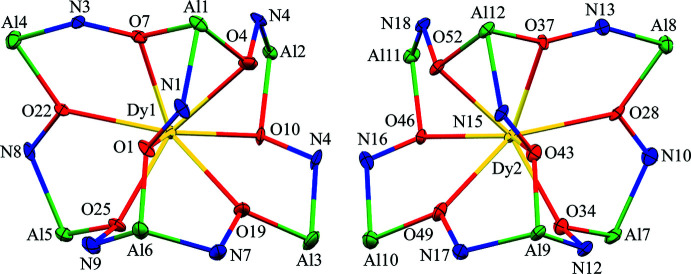
The metallacryptate views of (*a*) **2A** and (*b*) **2B** demonstrating that the two metallacryptates of the dimeric unit are enanti­omers. See Fig. 2 ▸ for additional display details.

**Figure 8:**
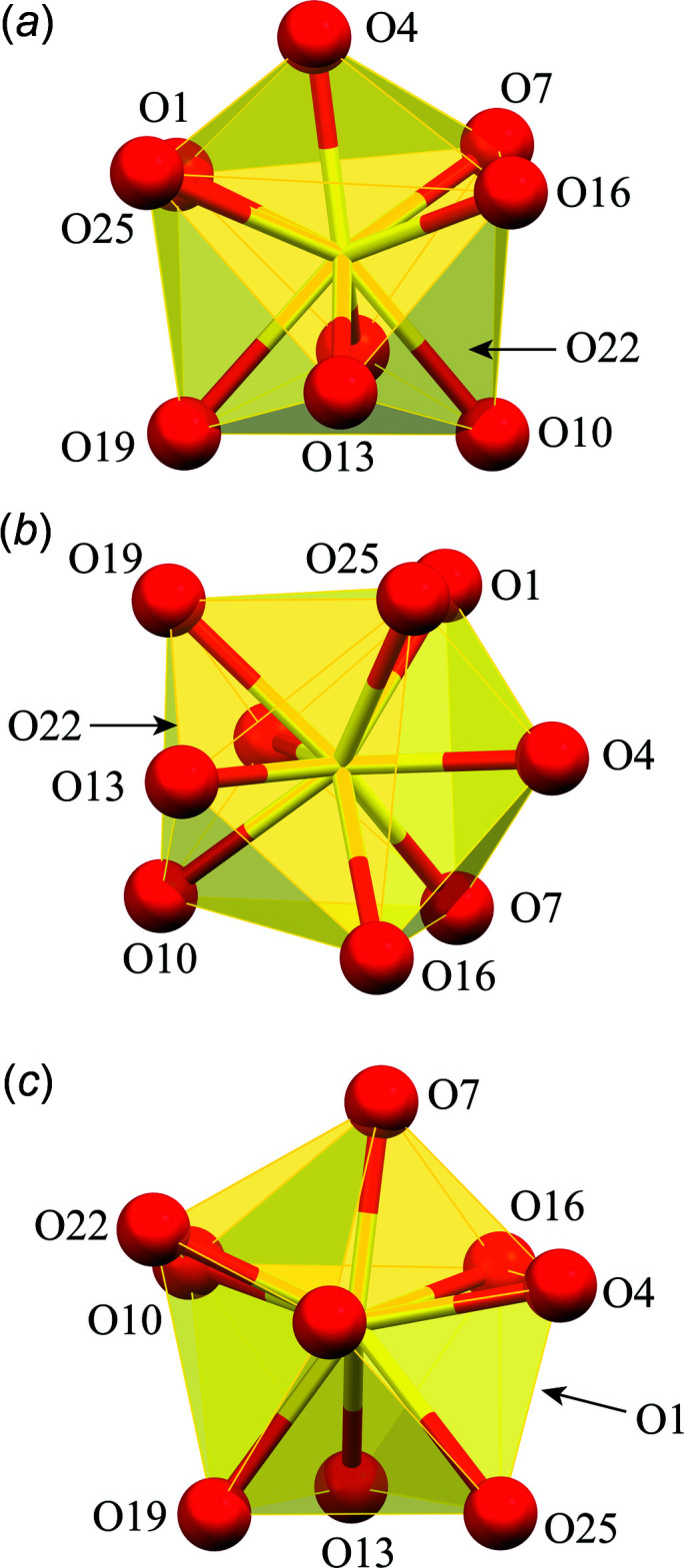
Polyhedral views of the coordination geometry for Dy1 of **1**: (*a*) spherical capped square anti­prism; (*b*) spherical tricapped trigonal prism; (*c*) muffin. See Fig. 2 ▸ for additional display details.

**Figure 9:**
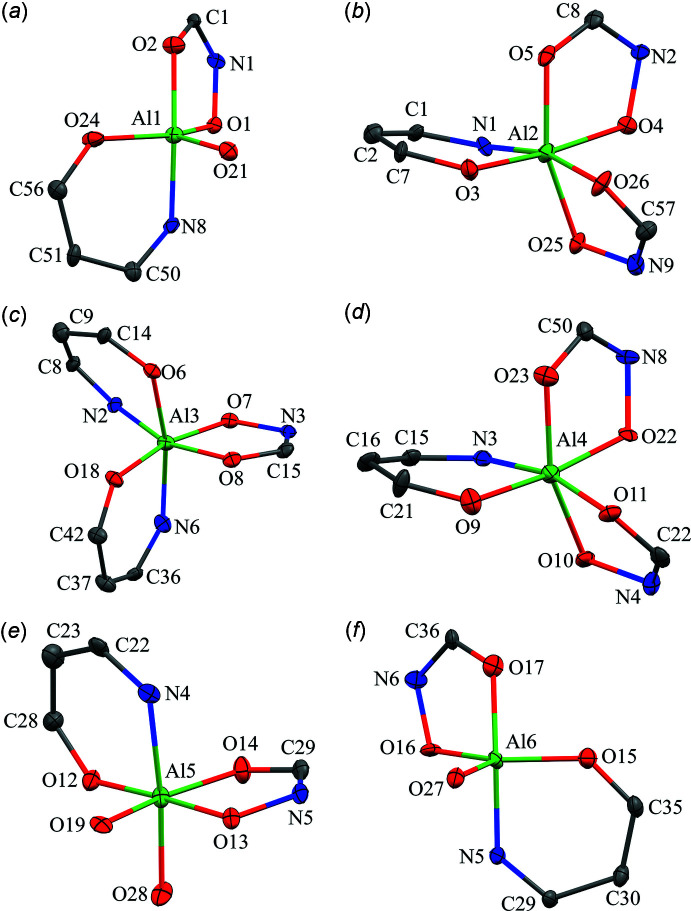
Coordination geometries for the Al^III^ ions of **1**: (*a*) Al1 – trigonal pyramidal; (*b*) Al2 – octa­hedral, Δ; (*c*) Al3 – octa­hedral, Λ; (*d*) Al4 – octa­hedral, Δ; (*e*) Al5 – octa­hedral, Λ; (*f*) Al6 – trigonal pyramidal. See Fig. 2 ▸ for additional display details.

A description of the coordination environment of Na1–Na3, Na5, Na6, and Na8–Na10 of **2** and the connectivity to neighboring metallacryptates follows. For Na1, the coordination environment consists of two μ-carbonyl oxygen atoms of two different bridging DMF mol­ecules, a carbonyl oxygen atom of a terminal DMF mol­ecule, two phenolate oxygen atoms of two different shi^3−^ ligands, and a carboxyl­ate oxygen atom of a third shi^3−^ ligand. The two bridging DMF mol­ecules connect Na1 to the Na10 ion of a neighboring metallacryptate. For Na2, the coordination environment consists of three μ-carbonyl oxygen atoms of bridging DMF mol­ecules, a carbonyl oxygen atom of a terminal DMF mol­ecule, a phenolate oxygen atom of a shi^3−^ ligand, and a carboxyl­ate oxygen atom of a different shi^3−^ ligand. The three bridging DMF mol­ecules connect Na2 to the Na3 ion of a neighboring metallacryptate. For Na3, the coordination environment consists of three μ-carbonyl oxygen atoms of bridging DMF mol­ecules, a carboxyl­ate oxygen atom of a shi^3−^ ligand, a phenolate oxygen atom of a different shi^3−^ ligand, and a carboxyl­ate oxygen atom of an Hshi^2−^ ligand. The three bridging DMF mol­ecules connect Na3 to the Na2 ion of a neighboring metallacryptate. For Na5, the coordination environment consists of two carbonyl oxygen atoms of terminal DMF mol­ecules, a carboxyl­ate oxygen atom of an acetate anion that bridges between Na5 and Al2, a carboxyl­ate oxygen atom of a different acetate anion that bridges between Na5 and Na7 of the same dimeric unit, a phenolate oxygen atom of a shi^3−^ ligand, and a carboxyl­ate oxygen atom of an Hshi^2−^ ligand. Na5 is not connected to any neighboring metallacryptate units. For Na6, the coordination environment consists of one μ-carbonyl oxygen atom of a bridging DMF mol­ecule that bridges between Na6 and Na4 of the same dimeric unit, one carbonyl oxygen atom of terminal DMF mol­ecule, a carboxyl­ate oxygen atom of an acetate anion that bridges between Na6 and Al11, a carboxyl­ate oxygen atom of a different acetate anion that bridges between Na6 and Na4 of the same dimeric unit, a phenolate oxygen atom of a shi^3−^ ligand, and a carboxyl­ate oxygen atom of an Hshi^2−^ ligand. Na6 is not connected to any neighboring metallacryptate units. For Na8, the coordination environment consists of three μ-carbonyl oxygen atoms of three different bridging DMF mol­ecules, a phenolate oxygen atom of a shi^3−^ ligand, a carboxyl­ate oxygen atom of a different shi^3−^ ligand, and a carboxyl­ate oxygen atom of an Hshi^2−^ ligand. The three bridging DMF mol­ecules connect Na8 to the Na9 ion of a neighboring metallacryptate. For Na9, the coordination environment consists of three μ-carbonyl oxygen atoms of three different bridging DMF mol­ecules, a carbonyl oxygen atom of a terminal DMF mol­ecule, a phenolate oxygen atom of a shi^3−^ ligand, and a carboxyl­ate oxygen atom of a different shi^3−^ ligand. The three bridging DMF mol­ecules connect Na9 to the Na8 ion of a neighboring metallacryptate. For Na10, the coordination environment consists of two μ-carbonyl oxygen atoms of two different bridging DMF mol­ecules, a carbonyl oxygen atom of a terminal DMF mol­ecule, two phenolate oxygen atoms of two different shi^3−^ ligands, and a carboxyl­ate oxygen atom of a third shi^3−^ ligand. The two bridging DMF mol­ecules connect Na10 to the Na1 ion of a neighboring metallacryptate. The series of μ-bridging DMF mol­ecules between the peripheral Na^+^ ions of the dimeric metallacryptate units of **2** generate the two-dimensional network of the compound (Fig. 5 ▸). For the dimeric unit, there are six connection points (Na1, Na2, Na3, Na8, Na9, and Na10) to neighboring metallacryptates. If the shape of the dimeric metallacryptate unit of **2** is approximated as a rectangle with Na2 and Na8 along one long edge, Na3 and Na9 along the opposite long edge, and Na1 and Na10 on opposite short edges, then each dimeric unit connects to one other dimeric unit along each edge *via* the μ-carbonyl oxygen atoms of the bridging DMF mol­ecules. Thus, each dimeric unit is connected to four adjacent dimeric metallacryptate units *via* the peripheral Na–DMF–Na connections and a two-dimensional sheet of metallacryptates is generated (Na1 to an adjacent Na10 on a short edge, Na10 to an adjacent Na1 on the opposite short edge, Na2 to an adjacent Na3 and Na8 to an adjacent Na9 along the same long edge, and Na3 to an adjacent Na2 and Na9 to an adjacent Na8 along the other long edge).

For both **1** and **2**, several solvent mol­ecules are located within the two-dimensional networks. For **1** there are four DMF mol­ecules and one water mol­ecule per metallacryptate. One of the DMF mol­ecules (associated with N21) is flip disordered over two sites and refined to 0.50 (2):0.50 (2). For **2** there are 6.335 DMF mol­ecules per dimeric metallacryptate unit. The DMF mol­ecules associated with N36 and N42 are disordered over two positions and refined to 0.700 (13):0.300 (13) and to 0.661 (12):0.339 (12), respectively. In addition, three DMF mol­ecules associated with N38, N40, and N41 are partially occupied and refined to 0.806 (9), 0.682 (16), and 0.847 (14), respectively. Moreover, both **1** and **2** contain solvent-accessible voids of 988 and 832 Å^3^, respectively. The residual electron density peaks were not arranged in an inter­pretable pattern; thus, the SQUEEZE routine as implemented in *PLATON* was used to account for the residual electron density (van der Sluis & Spek, 1990 ▸; Spek, 2015 ▸). The SQUEEZE procedure accounted for 313 and 235 electrons within the solvent-accessible voids of **1** and **2**, respectively.

## Supra­molecular features   

For **1** and **2**, numerous hydrogen bonds and weak C—H⋯O inter­actions exist within each metallacryptate, between components of the metallacryptate and the bridging DMF mol­ecules, and between the two-dimensional network and the solvent mol­ecules (Tables 7 ▸ and 8 ▸). For **1**, the protonated oxime nitro­gen atoms of the two Hshi^2−^ ligands each form a hydrogen bond to the oxime oxygen atom of a neighboring shi^3−^ ligand (N7—H7⋯O22 and N9—H9⋯O13). The water mol­ecule (O42) forms hydrogen bonds to the phenolate oxygen atom of a shi^3−^ ligand (O42—H42*A*⋯O3) and to the carbonyl oxygen atom of a DMF mol­ecule coordinated to Na4 (O42—H42*B*⋯O37). In addition, O42 inter­acts with the methine group of a μ-DMF mol­ecule (C66—H66⋯O42). Two C—H⋯O inter­actions exist between the carbon–hydrogen atoms of the benzene rings of the shi^3−^ ligands with the carbonyl oxygen atoms of the μ-DMF mol­ecules that bridge between two Na^+^ ions (associated with C20 and C41). Furthermore, the DMF mol­ecules form numerous C—H⋯O inter­actions with neighboring oxygen atoms through either the methine or the methyl groups of the DMF mol­ecules. The methine groups form inter­actions with the oxime oxygen atom of a shi^3−^ ligand (associated with C69), the carboxyl­ate oxygen atom of Hshi^2−^ (associated with C78) or shi^3−^ (associated with C84) ligands, and the carbonyl oxygen atom of neighboring DMF mol­ecules (associated with C78). The methyl groups form inter­actions with the carbonyl oxygen atoms of neighboring DMF mol­ecules (associated with C67, C74, C79, C80, C83, C94, C95, and C97), the oxime oxygen atom of a shi^3−^ ligand (associated with C71), and the phenolate oxygen atom of shi^3−^ (associated with C73, C79, C98) or Hshi^2−^ (associated with C92) ligands.

Complex **2** has similar hydrogen bonds and weak C—H⋯O inter­actions as **1**. For **2**, the protonated oxime nitro­gen atoms of the four Hshi^2−^ ligands each form a hydrogen bond to the oxime oxygen atom of a neighboring shi^3−^ ligand (N5—H5*N*⋯O7, N6—H6*N*⋯O10, N11—H11*N*⋯O46, and N14—H14*N*⋯O37). Four C—H⋯O inter­actions exist between the carbon–hydrogen atoms of the benzene rings of the shi^3−^ ligands with the carbonyl oxygen atoms of the DMF mol­ecules coordinated to the Na^+^ ions (associated with C4, C60, C81, and C123). The methyl groups of the acetate anions also form C—H⋯O inter­actions to the carbonyl oxygen atom of a DMF mol­ecule coordinated to a Na^+^ ion (C128—H12*C*⋯O69*B*) and to a phenolate oxygen atom of an Hshi^2−^ ligand (C130—H13*B*⋯O14). Furthermore, the DMF mol­ecules form C—H⋯O inter­actions with neighboring oxygen atoms through either the methine or the methyl groups of the DMF mol­ecules. The methine groups form inter­actions with the oxime oxygen atom of shi^3−^ ligands (associated with C137 and C173) and the carbonyl oxygen atom of neighboring DMF mol­ecules (associated with C140 and C191). The methyl groups form inter­actions with the carbonyl oxygen atoms of neighboring DMF mol­ecules (associated with C144, C157, C159, C169, C171, C172, and C204), the oxime oxygen atoms of shi^3−^ ligands (associated with C139 and C175), the phenolate oxygen atoms of shi^3−^ (associated with C168, C183, and C184) or Hshi^2−^ (associated with C201 and C223) ligands, and the carboxyl­ate oxygen atom of shi^3−^ (associated with C153, C162, C163, C183, and C199) or Hshi^2−^ (associated with C159) ligands.

## Database survey   

A survey of the Cambridge Structural Database (CSD version 5.41, update March 2020, Groom *et al.*, 2016 ▸) reveals that there is only one other comparable metallacryptate, which is based on gallium (DIBLOS; Lutter *et al.*, 2018 ▸). The structure contains a [3.3.1] metallacryptand topology built with six Ga^III^ ions instead of Al^III^ ions and also contains seven shi^3−^ ligands as in **1** and **2**, but the gallium-based structure has one H_2_shi^−^ and one Hshi^2−^ ligand as opposed to the two Hshi^2−^ ligands in **1** and **2**. For the gallium-based structure, a Tb^III^ ion is captured in the central cavity to form the metallacryptate. The major difference between **1** and **2** and the gallium-based structure is that the latter is only a discrete mol­ecule and not a two-dimensional network. For the gallium-based structure, there are no sodium ions. Instead charge neutrality is maintained by the presence of three tri­ethyl­ammonium cations, which are not coordinated to the metallacryptates and which do not form bridges between them.

## Synthesis and crystallization   

**Synthetic materials**

Salicyl­hydroxamic acid (H_3_shi, 99%) and dysprosium(III) nitrate penta­hydrate (99.9%) were purchased from Alfa Aesar. Hy­droxy­aluminum di­acetate (99+%) was purchased from Matheson Coleman and Bell. Sodium acetate trihydrate (certified ACS grade) was purchased from Fisher Scientific. *N,N*-Di­methyl­formamide (DMF, certified ACS grade) and diethyl ether (certified ACS grade) were purchased from Pharmco–Aaper. All reagents were used as received and without further purification.

**General preparation of [3.3.1]DyAl_6_Na_5_ -metallacryptate compounds** The same procedure was used to synthesize **1** and **2**; however, the syntheses resulted in the crystallization of the compound in two different structures. Dysprosium(III) nitrate penta­hydrate (0.125 mmol, 55.7 mg for **1**, 54.8 mg for **2**), sodium acetate trihydrate (4 mmol, 544.7 mg for **1**, 545.3 mg for **2**), and salicyl­hydroxamic acid (2 mmol, 306.4 mg for **1**, 307.2 mg for **2**) were mixed in 10 mL of DMF resulting in a cloudy, slightly pink mixture. In a separate beaker, hy­droxy­aluminum di­acetate (2 mmol, 324.6 mg for **1**, 325.1 mg for **2**) was mixed in 10 mL of DMF, resulting in a cloudy, white mixture. The two solutions were mixed, resulting in a cloudy, slightly pink mixture and allowed to stir overnight. The solution was filtered the next day to remove a white precipitate, which was discarded, and a pale-yellow filtrate was recovered. X-ray quality crystals were grown *via* diethyl ether diffusion at room temperature. White, slightly pink, thin, needle-shaped crystals were recovered after 11 days for **1** and 28 days for **2**.

**[DyAl_6_Na_5_(OAc)(Hshi)_2_(shi)_7_(DMF)_8_]·H_2_O·4DMF, 1:** The percentage yield was 2% (4.8 mg, 1.75x10^−3^ mmol) based on dysprosium(III) nitrate penta­hydrate.

**[DyAl_6_Na_5_(OAc)(Hshi)_2_(shi)_7_(DMF)_8.5_]_2_·6.335DMF, 2:** The percentage yield was 6% (20.4 mg, 3.77x10^−3^ mmol) based on dysprosium(III) nitrate penta­hydrate.

## Refinement   

For complex **1**, the crystal under investigation was found to have two components, but the domains were not related by any meaningful twin relationship. The orientation matrices for the two components were identified using the program *CELL_NOW* (Sheldrick, 2008*b* ▸), with the two components being related by a 3.5° rotation around the reciprocal axis 0 0 1. The structure was solved by direct methods with only the non-overlapping reflections of component 1, and the structure was refined using all reflections of component 1 (including the overlapping ones), resulting in a BASF value of 0.40356 (9). The *R*_int_ value given is for all reflections and is based on agreement between observed single and composite intensities and those calculated from refined unique intensities and domain fractions (*TWINABS*; Sheldrick, 2012 ▸).

For a portion of the metallacryptate mol­ecule (Dy1, Al3, Al5, O1, O5, O7, O10, O13, O14, N2, N3, N4, C8, C9, C22, C29, and C30) anisotropic displacement parameters in the bond direction were restrained to be similar using a RIGU restraint in *SHELXL* (esd = 0.001 Å^2^). In addition, to avoid correlation of the ellipsoids of Al5 and O13, their ADPs were constrained to be identical.

The geometry of the DMF mol­ecule associated with N18 was restrained to be similar to the DMF mol­ecule associated with N10 (esd = 0.02 Å), and the atoms of the DMF mol­ecule were restrained to have similar *U^ij^* components of their ADPs (esd = 0.01 Å^2^; SIMU restraint in *SHELXL*).

A DMF mol­ecule, associated with N21, is flip disordered over two sites. The geometries of the two DMF mol­ecules were restrained to be similar to the DMF mol­ecule associated with N10 (esd = 0.02 Å). The atoms of the DMF mol­ecules were restrained to have similar *U^ij^* components of the ADPs (esd = 0.01 Å^2^; SIMU restraint in *SHELXL*), and the *U^ij^* components of the atoms were restrained to approximate isotropic behavior (esd = 0.01 Å^2^). Subject to these restraints, the occupancy ratio of the disordered DMF mol­ecule refined to 0.50 (2):0.50 (2).

For the water mol­ecule associated with O42, the oxygen–hydrogen bond distances were restrained to 0.84 (2) Å, the hydrogen–hydrogen distance was restrained to 1.36 (2) Å, and the hydrogen atoms were refined as riding on the oxygen atoms. Several hydrogen-bond distances between the hydrogen atom and the acceptor atom were restrained to expected target values (H42*A* to O3, H42*B* to O37, H7 to N7 and O21, and H9 to N9 and O27).

The amide N—H groups were refined subject to a distance constraint of 0.88 Å.

To avoid conflict between the hydrogen atoms and neighboring carbon atoms of DMF mol­ecules, the distances between the atoms (C100 with H94*A*, H94*B*, and H94*C* and C94 with H10*G*, H10*H*, and H10*I*) were restrained to at least 2.80 Å.

For the methyl-group carbon atoms C85, C86, C91, C92, C95, C101, C102, and C103, hydrogen atoms were placed in tetra­hedral positions with an ideal staggered geometry (AFIX 33 in *SHELXL*). All other carbon-bound hydrogen atoms were placed in calculated positions and refined as riding on their carrier atoms with C—H distances of 0.95 Å for *sp* ^2^ carbon atoms and 0.98 Å for methyl carbon atoms. The *U*_iso_ values for hydrogen atoms were set to a multiple of the value of the carrying carbon atom (1.2 × for *sp* ^2^-hybridized carbon and nitro­gen atoms or 1.5 × for methyl carbon atoms and water oxygen atoms).

In addition, the structure of **1** contains solvent-accessible voids of 988 Å^3^. The residual electron density peaks are not arranged in an inter­pretable pattern. The structure factors were instead augmented via reverse Fourier transform methods using the SQUEEZE routine (van der Sluis & Spek, 1990 ▸; Spek, 2015 ▸) as implemented in the program *PLATON* (Spek, 2020 ▸). The resultant files were used in the further refinement. (The ‘FAB’ file with details of the SQUEEZE results is appended to the CIF file). The SQUEEZE procedure accounted for 313 electrons within the solvent-accessible voids. Additional crystal data, data collection and structure refinement details are summarized in Table 9 ▸.

For complex **2**, the structure was refined as a two-component inversion twin. The BASF value refined to 0.097 (3). In addition, several solvate DMF mol­ecules and one salicyl­hydroximate ligand of the mol­ecule show disorder or were partially occupied. Several DMF mol­ecules were also refined as disordered over two positions. Areas with extensively disordered unidentified solvate mol­ecules are present, which were accounted for using the SQUEEZE routine.

To model the disorder of the DMF mol­ecules, the following conditions were applied: For all of the solvate DMF mol­ecules except the DMF associated with N19, neighboring atoms were restrained to have similar *U^ij^* components of their ADPs (SIMU restraints in *SHELXL*). All DMF mol­ecules were restrained to have similar geometries to that of the DMF mol­ecule associated with N19 (esd = 0.02 Å). The atoms of the DMF mol­ecule associated with N36, N37, N39 and N42 and the atoms C1, C6, and C99 were restrained to be approximately isotropic. The atom pairs O60 and O60*B*, O63 and O63*B*, and O70 and O70*B* were each given identical coord­inates and thermal parameters to avoid correlation of their positional and thermal parameters. An anti-bumping restraint was applied to avoid close contacts for partially occupied or disordered DMF mol­ecules (BUMP −0.04 in *SHELXL*). Subject to the above conditions, the occupancy ratio of the DMF mol­ecules associated with N20, N23, N24, N29, N30, N34, N36, and N42 refined to 0.847 (14):0.153 (14), 0.778 (14):0.222 (14), 0.661 (12):0.339 (12), 0.806 (9):0.194 (9), 0.703 (14):0.297 (14), 0.818 (14):0.182 (14), 0.700 (13):0.300 (13), and 0.661 (12):0.339 (12), respectively. Three DMF mol­ecules associated with N38, N40, and N41 were refined as partially occupied and subject to the above conditions, their occupancy fractions refined to 0.806 (9), 0.682 (16), and 0.847 (14), respectively.

To model the disorder of the salicyl­hydroximate ligand associated with N8, the neighboring atoms of the ligand were restrained to have similar *U^ij^* components of their ADPs (SIMU restraint in *SHELXL*). The atom pair C50 and C50*B* were each given identical coordinates and thermal parameters to avoid correlation of their positional and thermal parameters. To maintain planarity of the benzene rings, the carbon atoms were restrained to lie in a common plane (esd = 0.1 Å^3^). Subject to the above conditions, the occupancy ratio for the ligands associated with N8 refined to 0.56 (5):0.44 (5). The amide N—H groups were refined subject to a distance constraint of 0.88 Å. To avoid correlation, the atoms Al9 and N12 were each given identical thermal parameters.

For portions of the benzene rings of four different salicyl­hydroximate ligands (C31–35, C41–42, C43–C49, and C79–C84), the anisotropic displacement parameters in the bond direction were restrained to be similar using a RIGU restraint in *SHELXL* (esd = 0.004 Å^2^).

For the methyl-group carbon atoms C147, C166, C186, C187, C192, C193, C195, C196, C198, C199, C201, C202, C210, C211, C216, C217, C222, C223, C225, C226, C231, and C232, hydrogen atoms were placed in tetra­hedral positions with an ideal staggered geometry (AFIX 33 in *SHELXL*). All other methyl-group hydrogen atoms were allowed to rotate. All other hydrogen atoms were placed in calculated positions and refined as riding on their carrier atoms with C—H distances of 0.95 Å for *sp* ^2^ carbon atoms and 0.98 Å for methyl carbon atoms. The *U*_iso_ values for hydrogen atoms were set to a multiple of the value of the carrying carbon atom (1.2 × for *sp* ^2^-hybridized carbon and nitro­gen atoms and 1.5 × for methyl carbon atoms).

In addition to the disordered solvate mol­ecules described above, there are additional solvent-accessible voids of 832 Å^3^. The structure factors of **2** were augmented *via* reverse Fourier transform methods using the SQUEEZE routine (van der Sluis & Spek, 1990 ▸; Spek, 2015 ▸), which accounted for 235 electrons within the solvent-accessible voids. Lastly, the following low-angle reflection (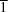 0 0) was obscured by the beam stop and was omitted from the refinement. Further crystal data, data collection and structure refinement details are summarized in Table 9 ▸.

## Supplementary Material

Crystal structure: contains datablock(s) 1, 2. DOI: 10.1107/S2056989020010130/pk2639sup1.cif

Structure factors: contains datablock(s) 1. DOI: 10.1107/S2056989020010130/pk26391sup2.hkl

Structure factors: contains datablock(s) 2. DOI: 10.1107/S2056989020010130/pk26392sup3.hkl

CCDC references: 2018399, 2018398

Additional supporting information:  crystallographic information; 3D view; checkCIF report

## Figures and Tables

**Figure 10:**
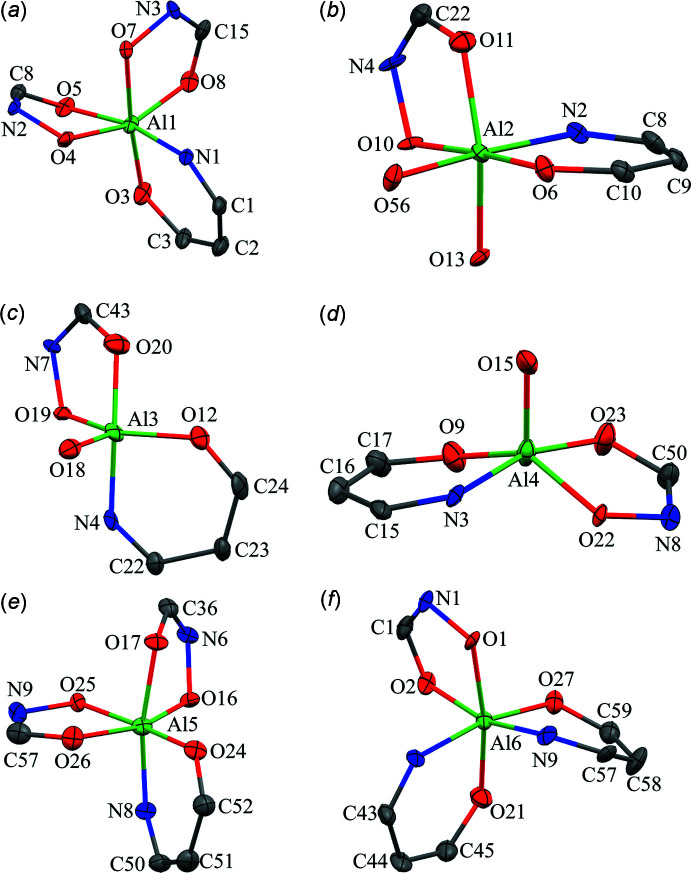
Coordination geometries for the Al^III^ ions of **2A**: (*a*) Al1 – octa­hedral, Δ; (*b*) Al2 – octa­hedral, Λ; (*c*) Al3 – trigonal pyramidal; (*d*) Al4 – spherical square pyramidal; (*e*) Al5 – octa­hedral, Δ; (*f*) Al6 – octa­hedral, Λ. See Fig. 2 ▸ for additional display details.

**Figure 11:**
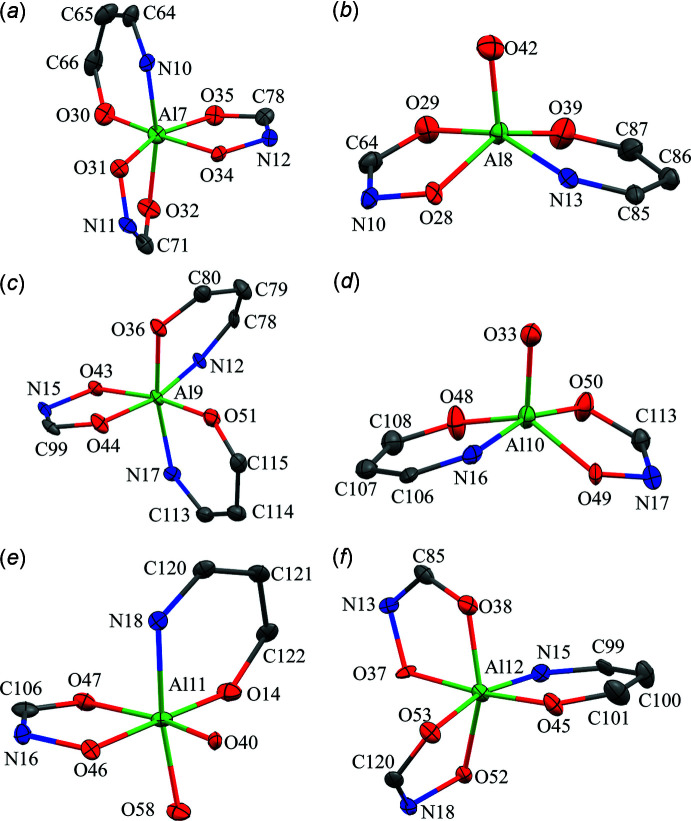
Coordination geometries for the Al^III^ ions of **2B**: (*a*) Al7 – octa­hedral, Λ; (*b*) Al8 – spherical square pyramidal; (*c*) Al9 – octa­hedral, Δ; (*d*) Al10 – spherical square pyramidal; (*e*) Al11 – octa­hedral, Δ; (*f*) Al12 – octa­hedral, Λ. See Fig. 2 ▸ for additional display details.

**Table 1:** Average bond length (Å) and bond-valence-sum (BVS) values (v.u.) used to support assigned oxidation states of the dysprosium and aluminium ions of **1** and **2**

	Avg. bond length	BVS value	Assigned oxidation state
**1**			
Dy1	2.420	3.02	3+
Al1	1.836	3.09	3+
Al2	1.900	3.08	3+
Al3	1.926	3.08	3+
Al4	1.907	3.00	3+
Al5	1.911	2.96	3+
Al6	1.841	3.03	3+
			
**2**			
Dy1	2.426	3.00	3+
Dy2	2.431	2.96	3+
Al1	1.912	2.97	3+
Al2	1.909	2.98	3+
Al3	1.845	3.01	3+
Al4	1.840	3.05	3+
Al5	1.911	2.99	3+
Al6	1.919	3.14	3+
Al7	1.913	2.98	3+
Al8	1.851	2.97	3+
Al9	1.924	309	3+
Al10	1.842	3.03	3+
Al11	1.907	3.00	3+
Al12	1.910	2.97	3+

**Table 2:** Continuous shapes measures (CShM) values for the geometry about the nine-coordinate central Dy^III^ ions of **1** and **2**

Shape	**1**	**2**	
	Dy1	Dy1	Dy2
Enneagon (*D_9h_*)	35.902	35.894	36.224
Octa­gonal pyramid (*C_8v_*)	22.494	22.501	22.625
Heptagonal bipyramid (*D_7h_*)	18.198	18.199	18.434
Johnson triangular cupola (J3; *C_3v_*)	14.832	15.047	15.010
Capped cube (J8; *C_4v_*)	10.814	10.767	10.982
Spherical-relaxed capped cube (*C_4v_*)	8.894	8.825	9.098
Capped square anti­prism (J10; *C_4v_*)	2.279	2.416	2.331
Spherical capped square anti­prism (*C_4v_*)	1.212	1.305	1.225
Tricapped trigonal prism (J51; *D_3h_*)	3.326	3.694	3.656
Spherical tricapped trigonal prism (*D_3h_*)	1.370	1.640	1.521
Tridiminished icosa­hedron (J63; *C_3v_*)	10.378	10.358	10.232
Hula-hoop (*C_2v_*)	11.630	11.649	11.491
Muffin (*C_s_*)	1.728	1.813	1.803

**Table 3:** Continuous shapes measures (CShM) values for the geometry about the five-coordinate Al^III^ ions of **1** and **2**

Shape	Penta­gon (*D_5h_*)	Vacant octa­hedron (*C_4v_*)	Trigonal bipyramid (*D_3h_*)	Spherical square pyramid (*C_4v_*)	Johnson trigonal bipyramid (J12; *D_3h_*)
**1**					
Al1	30.916	3.299	1.533	1.880	3.644
Al6	32.803	3.560	1.220	1.797	3.677
					
**2**					
Al3	33.414	4.086	0.874	2.325	3.263
Al4	32.043	2.315	2.109	1.168	4.370
Al8	31.548	2.102	3.184	0.540	5.340
Al10	31.615	2.912	1.759	1.324	4.191

**Table 4:** Continuous shapes measures (CShM) values for the geometry about the six-coordinate Al^III^ ions of **1** and **2**

Shape	Hexagon (*D_6h_*)	Penta­gonal pyramid (*C_5v_*)	Octa­hedron (*O_h_*)	Trigonal prism (*D_3h_*)	Johnson penta­gonal pyramid (J2; *C_5v_*)
**1**					
Al2	32.362	22.306	1.596	10.843	26.394
Al3	32.252	22.678	1.787	8.768	26.344
Al4	31.297	21.640	1.619	10.460	25.730
Al5	33.212	25.069	0.880	13.274	28.854
					
**2**					
Al1	31.858	22.338	1.532	10.500	26.606
Al2	33.006	24.175	1.027	13.226	27.967
Al5	32.221	22.454	1.569	10.493	26.647
Al6	32.099	23.135	1.305	10.051	27.495
Al7	32.157	21.844	1.748	9.965	26.103
Al9	32.121	23.409	1.309	10.126	27.310
Al11	32.403	24.802	1.027	12.116	28.736
Al12	32.110	22.252	1.547	10.726	26.505

**Table 5:** Continuous shapes measures (CShM) values for the geometry about the five-coordinate Na^+^ ions of **1** and **2**

Shape	Penta­gon (*D_5h_*)	Vacant octa­hedron (*C_4v_*)	Trigonal bipyramid (*D_3h_*)	Spherical square pyramid (*C_4v_*)	Johnson trigonal bipyramid (J12; *D_3h_*)
**1**					
Na4	28.509	5.866	4.799	2.726	8.418
					
**2**					
Na4	24.436	5.808	4.193	4.292	6.350
Na7	32.074	8.161	3.598	4.728	5.923

**Table 6:** Continuous shapes measures (CShM) values for the geometry about the six-coordinate Na^+^ ions of **1** and **2**

Shape	Hexagon (*D_6h_*)	Penta­gonal pyramid (*C_5v_*)	Octa­hedron (*O_h_*)	Trigonal prism (*D_3h_*)	Johnson penta­gonal pyramid (J2; *C_5v_*)
**1**					
Na1	23.743	17.692	4.017	10.799	20.480
Na2	28.605	25.190	3.864	10.420	28.150
Na3	26.941	26.970	4.054	14.900	30.159
Na5	27.270	23.739	2.428	14.501	29.956
					
**2**					
Na1	28.015	22.084	4.608	13.472	23.950
Na2	23.347	17.492	4.089	11.501	20.119
Na3	26.767	27.219	3.890	14.542	30.261
Na5	31.841	18.329	3.676	10.228	21.995
Na6	27.107	21.224	2.932	12.611	25.625
Na8	25.747	26.564	4.280	14.099	29.546
Na9	24.002	17.120	4.074	10.668	19.939
Na10	27.061	21.910	4.759	13.316	23.861

**Table 7:** Hydrogen-bond geometry (Å, °) for **1** [Chem scheme1]

*D*—H⋯*A*	*D*—H	H⋯*A*	*D*⋯*A*	*D*—H⋯*A*
O42—H42*A*⋯O3	0.84	2.08	2.868 (14)	156
O42—H42*B*⋯O37	0.84	2.08	2.820 (15)	146
N7—H7⋯O22	0.90 (3)	2.25 (6)	2.899 (11)	129 (5)
N9—H9⋯O27	0.91 (3)	1.92 (3)	2.693 (11)	142 (6)
C20—H20⋯O35^i^	0.95	2.65	3.483 (16)	147
C41—H41⋯O34	0.95	2.60	3.469 (14)	153
C66—H66⋯O42^i^	0.95	2.36	3.30 (2)	171
C67—H67*A*⋯O31	0.98	2.47	3.41 (2)	159
C69—H69⋯O10	0.95	2.50	3.260 (14)	137
C71—H71*C*⋯O16	0.98	2.56	3.363 (16)	139
C73—H73*A*⋯O6	0.98	2.33	3.268 (14)	159
C74—H74*C*⋯O38^ii^	0.98	2.34	3.32 (3)	177
C78—H78⋯O20^iii^	0.95	2.56	3.260 (14)	131
C78—H78⋯O37^iii^	0.95	2.65	3.524 (16)	154
C79—H79*B*⋯O41	0.98	2.45	3.25 (4)	139
C79—H79*C*⋯O18	0.98	2.48	3.446 (17)	168
C80—H80*A*⋯O37^iii^	0.98	2.52	3.470 (18)	163
C80—H80*B*⋯O41	0.98	2.49	3.31 (4)	140
C83—H83*B*⋯O40	0.98	2.35	3.309 (19)	166
C84—H84⋯O11^iv^	0.95	2.58	3.292 (18)	132
C84—H84⋯O23^iv^	0.95	2.54	3.414 (18)	153
C92—H92*B*⋯O21^iv^	0.98	2.40	3.35 (2)	164
C94—H94*A*⋯O41	0.98	2.47	3.32 (4)	145
C95—H95*A*⋯O41	0.98	2.36	3.23 (4)	147
C97—H97*B*⋯O31^iv^	0.98	2.57	3.35 (3)	137
C98—H98*B*⋯O24^v^	0.98	2.65	3.318 (19)	125

Symmetry codes: (i) 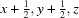; (ii) 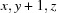; (iii) 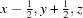; (iv) 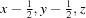; (v) 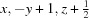.

**Table 8:** Hydrogen-bond geometry (Å, °) for **2** [Chem scheme1]

*D*—H⋯*A*	*D*—H	H⋯*A*	*D*⋯*A*	*D*—H⋯*A*
N5—H5*N*⋯O7	0.89 (3)	2.36 (7)	3.059 (8)	136 (7)
N6—H6*N*⋯O10	0.87 (3)	2.46 (8)	3.004 (9)	121 (7)
N11—H11*N*⋯O46	0.87 (3)	2.27 (6)	3.003 (8)	142 (8)
N14—H14*N*⋯O37	0.89 (3)	2.22 (8)	2.795 (8)	122 (7)
C4—H4⋯O62	0.95	2.64	3.477 (11)	147
C60—H60⋯O74^i^	0.95	2.55	3.287 (19)	135
C81—H81⋯O60^ii^	0.95	2.59	3.293 (12)	131
C123—H123⋯O68	0.95	2.48	3.333 (11)	150
C128—H12*C*⋯O69*B*	0.98	2.39	3.14 (5)	133
C130—H13*B*⋯O14	0.98	2.40	3.251 (10)	145
C137—H137⋯O4	0.95	2.36	3.221 (10)	151
C139—H13*T*⋯O19	0.98	2.53	3.459 (13)	159
C140—H140⋯O61	0.95	2.41	3.137 (11)	134
C144—H14*I*⋯O61	0.98	2.65	3.599 (15)	164
C153—H15*G*⋯O11	0.98	2.60	3.450 (13)	146
C157—H15*P*⋯O77^iii^	0.98	2.35	3.241 (19)	150
C159—H15*S*⋯O78^iv^	0.98	2.38	3.332 (14)	165
C159—H15*U*⋯O41	0.98	2.51	3.486 (13)	175
C162—H16*F*⋯O29	0.98	2.60	3.343 (15)	132
C163—H16*G*⋯O48^iv^	0.98	2.57	3.540 (14)	170
C168—H16*P*⋯O51	0.98	2.63	3.388 (12)	135
C169—H16*T*⋯O80^iii^	0.98	2.65	3.55 (2)	153
C171—H17*C*⋯O73^v^	0.98	2.42	3.360 (12)	161
C172—H17*E*⋯O66	0.98	2.61	3.518 (14)	154
C173—H173⋯O52	0.95	2.37	3.254 (9)	154
C175—H17*J*⋯O49	0.98	2.58	3.434 (13)	146
C183—H18*H*⋯O9	0.98	2.64	3.60 (3)	166
C183—H18*H*⋯O23	0.98	2.43	3.15 (3)	130
C184—H18*K*⋯O9	0.98	2.64	3.56 (2)	156
C191—H191⋯O77^vi^	0.95	2.64	3.48 (3)	148
C199—H19*T*⋯O29	0.98	2.52	3.50 (4)	179
C199—H19*U*⋯O36	0.98	2.59	3.39 (3)	139
C201—H20*D*⋯O15	0.98	2.50	3.30 (3)	139
C204—H20*G*⋯O68^v^	0.98	2.26	3.21 (5)	163
C223—H22*G*⋯O15	0.98	2.42	3.25 (5)	142

Symmetry codes: (i) 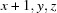; (ii) 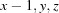; (iii) 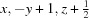; (iv) 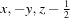; (v) 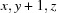; (vi) 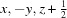.

**Table 9:** Experimental details

	**1**	**2**
Crystal data		
Chemical formula	[DyAl_6_Na_5_(C_7_H_5_NO_3_)_2_(C_7_H_4_NO_3_)_7_(C_2_H_3_O_2_)(C_3_H_7_NO)_8_]·4C_3_H_7_NO·H_2_O	[Dy_2_Al_12_Na_10_(C_7_H_5_NO_3_)_4_(C_7_H_4_NO_3_)_14_(C_2_H_3_O_2_)_2_(C_3_H_7_NO)_17_]·6.335C_3_H_7_NO
*M*_r_	2746.57	5408.50
Crystal system, space group	Monoclinic, *C* *c*	Monoclinic, *P* *c*
Temperature (K)	100	100
*a*, *b*, *c* (Å)	22.6809 (13), 20.5788 (11), 27.6206 (16)	28.1847 (14), 15.3683 (8), 30.7354 (16)
β (°)	101.590 (2)	106.461 (2)
*V* (Å^3^)	12628.9 (12)	12767.4 (11)
*Z*	4	2
Radiation type	Mo *K*α	Cu *K*α
μ (mm^−1^)	0.74	4.44
Crystal size (mm)	0.15 × 0.15 × 0.14	0.25 × 0.23 × 0.19

Data collection		
Diffractometer	Bruker D8 Quest CMOS	Bruker X8 Prospector CCD
Absorption correction	Multi-scan (*SADABS*; Bruker, 2014 ▸)	Multi-scan (*SADABS*; Bruker, 2014 ▸)
*T*_min_, *T*_max_	0.628, 0.746	0.488, 0.753
No. of measured, independent and observed [*I* > 2σ(*I*)] reflections	40899, 40899, 36071	154195, 38274, 36111
*R*_int_	0.071	0.050
(sin θ/λ)_max_ (Å^−1^)	0.738	0.596

Refinement		
*R*[*F* ^2^ > 2σ(*F* ^2^)], *wR*(*F* ^2^), *S*	0.056, 0.159, 1.05	0.053, 0.137, 1.02
No. of reflections	40899	38274
No. of parameters	1634	3599
No. of restraints	393	4954
H-atom treatment	H atoms treated by a mixture of independent and constrained refinement	H atoms treated by a mixture of independent and constrained refinement
Δρ_max_, Δρ_min_ (e Å^−3^)	1.38, −2.04	2.28, −1.57
Absolute structure	Flack *x* determined using 7452 quotients [(*I* ^+^)−(*I* ^−^)]/[(*I* ^+^)+(*I* ^−^)] (Parsons *et al.*, 2013 ▸)	Refined as an inversion twin
Absolute structure parameter	0.026 (5)	0.097 (3)

Computer programs: *APEX2* and *SAINT* (Bruker, 2014 ▸), *SHELXS97* (Sheldrick, 2008*a* ▸), *SHELXL2018* (Sheldrick, 2015 ▸) *shelXle* (Hübschle *et al.*, 2011 ▸), *Mercury* (Macrae *et al.*, 2020 ▸), and *publCIF* (Westrip, 2010 ▸).

## supplementary crystallographic information

### Poly[[µ3-acetato-hexakis(µ-N,N-dimethylformamide)bis(N,N-dimethylformamide)bis[salicylhydroximato(2-)]heptakis[salicylhydroximato(3-)]hexaaluminium(III)dysprosium(III)pentasodium(I)] N,N-dimethylformamide tetrasolvate monohydrate] (1) Crystal data

**Table:**

[DyAl_6_Na_5_(C_7_H_5_NO_3_)_2_(C_7_H_4_NO_3_)_7_(C_2_H_3_O_2_)(C_3_H_7_NO)_8_]·4C_3_H_7_NO·H_2_O	*F*(000) = 5660
*M**_r_* = 2746.57	*D*_x_ = 1.445 Mg m^−^^3^
Monoclinic, *C**c*	Mo *K*α radiation, λ = 0.71073 Å
*a* = 22.6809 (13) Å	Cell parameters from 9947 reflections
*b* = 20.5788 (11) Å	θ = 2.9–30.4°
*c* = 27.6206 (16) Å	µ = 0.74 mm^−^^1^
β = 101.590 (2)°	*T* = 100 K
*V* = 12628.9 (12) Å^3^	Block, colorless
*Z* = 4	0.15 × 0.15 × 0.14 mm

### Poly[[µ3-acetato-hexakis(µ-N,N-dimethylformamide)bis(N,N-dimethylformamide)bis[salicylhydroximato(2-)]heptakis[salicylhydroximato(3-)]hexaaluminium(III)dysprosium(III)pentasodium(I)] N,N-dimethylformamide tetrasolvate monohydrate] (1) Data collection

**Table:**

Bruker D8 Quest CMOS diffractometer	40899 independent reflections
Radiation source: I-mu-S microsource X-ray tube	36071 reflections with *I* > 2σ(*I*)
Laterally graded multilayer (Goebel) mirror monochromator	*R*_int_ = 0.071
ω and phi scans	θ_max_ = 31.7°, θ_min_ = 2.5°
Absorption correction: multi-scan (SADABS; Bruker, 2014)	*h* = −28→31
*T*_min_ = 0.628, *T*_max_ = 0.746	*k* = −26→27
40899 measured reflections	*l* = −36→39

### Poly[[µ3-acetato-hexakis(µ-N,N-dimethylformamide)bis(N,N-dimethylformamide)bis[salicylhydroximato(2-)]heptakis[salicylhydroximato(3-)]hexaaluminium(III)dysprosium(III)pentasodium(I)] N,N-dimethylformamide tetrasolvate monohydrate] (1) Refinement

**Table:**

Refinement on *F*^2^	Hydrogen site location: mixed
Least-squares matrix: full	H atoms treated by a mixture of independent and constrained refinement
*R*[*F*^2^ > 2σ(*F*^2^)] = 0.056	*w* = 1/[σ^2^(*F*_o_^2^) + (0.0559*P*)^2^ + 94.4342*P*] where *P* = (*F*_o_^2^ + 2*F*_c_^2^)/3
*wR*(*F*^2^) = 0.159	(Δ/σ)_max_ = 0.034
*S* = 1.05	Δρ_max_ = 1.38 e Å^−^^3^
40899 reflections	Δρ_min_ = −2.04 e Å^−^^3^
1634 parameters	Absolute structure: Flack *x* determined using 7452 quotients [(*I*^+^)-(*I*^-^)]/[(*I*^+^)+(*I*^-^)] (Parsons *et al.*, 2013)
393 restraints	Absolute structure parameter: 0.026 (5)

### Poly[[µ3-acetato-hexakis(µ-N,N-dimethylformamide)bis(N,N-dimethylformamide)bis[salicylhydroximato(2-)]heptakis[salicylhydroximato(3-)]hexaaluminium(III)dysprosium(III)pentasodium(I)] N,N-dimethylformamide tetrasolvate monohydrate] (1) Special details

**Table:**

Geometry. All esds (except the esd in the dihedral angle between two l.s. planes) are estimated using the full covariance matrix. The cell esds are taken into account individually in the estimation of esds in distances, angles and torsion angles; correlations between esds in cell parameters are only used when they are defined by crystal symmetry. An approximate (isotropic) treatment of cell esds is used for estimating esds involving l.s. planes.
Refinement. For complex 1 the crystal under investigation was found to have two components, but the domains were not related by any meaningful twin relationship.

### Poly[[µ3-acetato-hexakis(µ-N,N-dimethylformamide)bis(N,N-dimethylformamide)bis[salicylhydroximato(2-)]heptakis[salicylhydroximato(3-)]hexaaluminium(III)dysprosium(III)pentasodium(I)] N,N-dimethylformamide tetrasolvate monohydrate] (1) Fractional atomic coordinates and isotropic or equivalent isotropic displacement parameters (Å^2^)

**Table:**

	*x*	*y*	*z*	*U*_iso_*/*U*_eq_	Occ. (<1)
Dy1	0.83641 (2)	0.62140 (2)	0.71524 (2)	0.01077 (10)	
Al1	0.80306 (15)	0.61749 (15)	0.56957 (12)	0.0111 (7)	
Al2	0.72217 (14)	0.50917 (14)	0.68558 (10)	0.0116 (6)	
Al3	0.70939 (14)	0.72285 (14)	0.73084 (11)	0.0115 (5)	
Al4	0.89367 (14)	0.76457 (14)	0.68592 (11)	0.0116 (6)	
Al5	0.98018 (13)	0.57341 (14)	0.75403 (10)	0.0115 (5)	
Al6	0.85062 (15)	0.61738 (15)	0.85757 (12)	0.0105 (7)	
Na1	1.01453 (19)	0.8570 (2)	0.73422 (15)	0.0179 (9)	
Na2	0.63312 (19)	0.8417 (2)	0.72183 (15)	0.0181 (9)	
Na3	0.6110 (2)	0.4410 (2)	0.70212 (17)	0.0207 (10)	
Na4	0.8874 (2)	0.4437 (2)	0.69879 (16)	0.0226 (9)	
Na5	1.0490 (2)	0.4538 (2)	0.70893 (17)	0.0198 (10)	
O1	0.7917 (3)	0.5879 (3)	0.6315 (2)	0.01077 (10)	
O2	0.7609 (3)	0.5423 (3)	0.5478 (2)	0.0154 (14)	
O3	0.6958 (3)	0.4324 (3)	0.6560 (3)	0.0143 (15)	
O4	0.7382 (3)	0.5897 (3)	0.7163 (2)	0.0100 (13)	
O5	0.6440 (3)	0.5460 (3)	0.6661 (2)	0.0132 (14)	
O6	0.6510 (3)	0.7487 (3)	0.6754 (2)	0.0128 (14)	
O7	0.7790 (3)	0.7142 (3)	0.7039 (3)	0.0115 (5)	
O8	0.7369 (3)	0.8103 (3)	0.7377 (3)	0.0134 (15)	
O9	0.9105 (4)	0.8456 (4)	0.7127 (3)	0.0166 (16)	
O10	0.9162 (3)	0.7017 (3)	0.7387 (2)	0.0131 (14)	
O11	0.9759 (3)	0.7563 (3)	0.6839 (3)	0.0133 (15)	
O12	1.0464 (3)	0.5681 (3)	0.7275 (2)	0.0143 (15)	
O13	0.9088 (3)	0.5759 (3)	0.7799 (2)	0.0115 (5)	
O14	1.0170 (3)	0.5978 (3)	0.8188 (3)	0.0162 (14)	
O15	0.8982 (4)	0.6530 (4)	0.9100 (3)	0.0171 (17)	
O16	0.8147 (3)	0.6502 (3)	0.7951 (3)	0.0123 (14)	
O17	0.7846 (3)	0.6521 (3)	0.8758 (3)	0.0156 (15)	
O18	0.6524 (3)	0.7423 (3)	0.7677 (3)	0.0144 (15)	
O19	0.9192 (3)	0.5560 (3)	0.6960 (2)	0.0128 (14)	
O20	0.9621 (4)	0.4584 (4)	0.6491 (3)	0.0210 (16)	
O21	0.8746 (3)	0.6006 (3)	0.5583 (3)	0.0156 (15)	
O22	0.8693 (3)	0.6850 (3)	0.6529 (2)	0.0097 (13)	
O23	0.8659 (3)	0.7982 (3)	0.6213 (3)	0.0153 (15)	
O24	0.7607 (4)	0.6653 (4)	0.5219 (3)	0.0173 (17)	
O25	0.8059 (3)	0.4979 (3)	0.7194 (2)	0.0122 (14)	
O26	0.7064 (3)	0.4712 (3)	0.7448 (2)	0.0150 (15)	
O27	0.8355 (3)	0.5326 (3)	0.8630 (2)	0.0146 (14)	
O28	0.9909 (3)	0.4830 (3)	0.7676 (3)	0.0163 (15)	
O29	0.9223 (4)	0.4118 (5)	0.7800 (4)	0.038 (2)	
O30	1.1174 (4)	0.8391 (4)	0.7431 (3)	0.0254 (18)	
O31	1.0018 (4)	0.8455 (5)	0.8168 (3)	0.037 (2)	
O32	0.6187 (4)	0.9202 (4)	0.6590 (3)	0.0221 (17)	
O33	0.6351 (4)	0.9330 (4)	0.7714 (3)	0.0245 (19)	
O34	0.5298 (4)	0.8395 (4)	0.7185 (3)	0.0238 (18)	
O35	0.5528 (4)	0.4641 (4)	0.7610 (3)	0.0240 (18)	
O36	0.5241 (4)	0.4064 (4)	0.6551 (3)	0.0258 (19)	
O37	0.8423 (4)	0.3771 (4)	0.6335 (3)	0.0326 (19)	
O39	0.3352 (8)	0.5105 (7)	0.9336 (6)	0.089 (5)	
O40	0.5343 (7)	0.4742 (6)	0.9417 (6)	0.078 (4)	
O41	0.4018 (17)	0.7139 (14)	0.8093 (13)	0.098 (9)	0.50 (2)
C99	0.378 (2)	0.7675 (18)	0.8173 (14)	0.077 (7)	0.50 (2)
H99	0.363730	0.794800	0.789607	0.107*	0.50 (2)
N21	0.371 (2)	0.788 (2)	0.8606 (13)	0.078 (6)	0.50 (2)
C100	0.392 (2)	0.7537 (19)	0.9067 (14)	0.090 (9)	0.50 (2)
H10G	0.381771	0.778720	0.934030	0.135*	0.50 (2)
H10H	0.372671	0.710960	0.905056	0.135*	0.50 (2)
H10I	0.435777	0.748172	0.912092	0.135*	0.50 (2)
C101	0.329 (3)	0.841 (2)	0.865 (2)	0.078 (11)	0.50 (2)
H10J	0.329838	0.849515	0.899890	0.117*	0.50 (2)
H10K	0.340467	0.879879	0.848993	0.117*	0.50 (2)
H10L	0.288134	0.827625	0.848675	0.117*	0.50 (2)
O41B	0.4220 (17)	0.7141 (17)	0.9347 (14)	0.115 (10)	0.50 (2)
C99B	0.381 (2)	0.757 (2)	0.9190 (15)	0.084 (7)	0.50 (2)
H99B	0.352992	0.767492	0.939065	0.117*	0.50 (2)
N21B	0.377 (2)	0.786 (2)	0.8755 (14)	0.079 (6)	0.50 (2)
C102	0.418 (2)	0.769 (2)	0.8433 (17)	0.076 (8)	0.50 (2)
H10M	0.408574	0.794794	0.813056	0.114*	0.50 (2)
H10N	0.459560	0.777669	0.860353	0.114*	0.50 (2)
H10O	0.413942	0.722517	0.834884	0.114*	0.50 (2)
C103	0.346 (2)	0.847 (2)	0.862 (2)	0.061 (9)	0.50 (2)
H10P	0.348865	0.858463	0.828014	0.092*	0.50 (2)
H10Q	0.303207	0.841932	0.863568	0.092*	0.50 (2)
H10R	0.363961	0.880998	0.884613	0.092*	0.50 (2)
O42	0.7754 (7)	0.3322 (7)	0.7025 (6)	0.093 (5)	
H42A	0.748851	0.361229	0.697006	0.139*	
H42B	0.791582	0.329229	0.677598	0.139*	
N1	0.7547 (4)	0.5331 (4)	0.6279 (3)	0.0107 (16)	
N2	0.6912 (4)	0.6350 (4)	0.7049 (3)	0.0114 (15)	
N3	0.8148 (4)	0.7705 (4)	0.7062 (3)	0.0116 (16)	
N4	0.9696 (4)	0.6690 (4)	0.7326 (3)	0.0130 (15)	
N5	0.9196 (4)	0.6024 (4)	0.8277 (3)	0.0113 (12)	
N6	0.7599 (4)	0.6836 (4)	0.7954 (3)	0.0156 (18)	
N7	0.9326 (4)	0.5632 (4)	0.6497 (3)	0.0163 (18)	
H7	0.914 (5)	0.595 (3)	0.6306 (16)	0.020*	
N8	0.8326 (4)	0.6952 (4)	0.6056 (3)	0.0125 (17)	
N9	0.8049 (4)	0.4891 (4)	0.7693 (3)	0.0141 (17)	
H9	0.832 (3)	0.502 (5)	0.7966 (17)	0.017*	
N10	1.1657 (5)	0.7559 (6)	0.7897 (4)	0.041 (3)	
N11	0.9118 (5)	0.8366 (5)	0.8402 (4)	0.033 (3)	
N12	0.6703 (5)	0.9210 (5)	0.5959 (4)	0.030 (3)	
N13	0.6806 (5)	0.9559 (5)	0.8511 (4)	0.033 (3)	
N14	0.4608 (5)	0.7563 (5)	0.7104 (4)	0.029 (2)	
N15	0.5869 (4)	0.5428 (5)	0.8173 (3)	0.021 (2)	
N16	0.4933 (7)	0.4073 (7)	0.5715 (5)	0.057 (4)	
N17	0.8230 (5)	0.3455 (5)	0.5519 (4)	0.027 (2)	
N19	0.3857 (9)	0.5680 (10)	0.8816 (7)	0.078 (5)	
N20	0.5776 (8)	0.3823 (7)	0.9812 (5)	0.064 (4)	
C1	0.7400 (4)	0.5108 (4)	0.5822 (3)	0.0104 (18)	
C2	0.7026 (5)	0.4536 (5)	0.5709 (4)	0.014 (2)	
C3	0.6854 (5)	0.4338 (5)	0.5209 (4)	0.021 (2)	
H3	0.698460	0.458335	0.495884	0.025*	
C4	0.6499 (6)	0.3793 (6)	0.5077 (4)	0.027 (3)	
H4	0.638735	0.366597	0.474068	0.032*	
C5	0.6308 (6)	0.3436 (5)	0.5444 (4)	0.024 (3)	
H5	0.606316	0.306256	0.535645	0.029*	
C6	0.6472 (5)	0.3617 (5)	0.5935 (4)	0.021 (2)	
H6	0.634417	0.336096	0.618173	0.025*	
C7	0.6822 (5)	0.4171 (5)	0.6074 (4)	0.015 (2)	
C8	0.6447 (4)	0.6089 (5)	0.6768 (4)	0.0127 (16)	
C9	0.5939 (5)	0.6502 (5)	0.6543 (4)	0.016 (2)	
C10	0.5399 (5)	0.6230 (6)	0.6307 (4)	0.023 (2)	
H10	0.535065	0.577229	0.631572	0.028*	
C11	0.4932 (6)	0.6602 (6)	0.6061 (5)	0.031 (3)	
H11	0.455783	0.640438	0.592145	0.037*	
C12	0.5007 (6)	0.7258 (6)	0.6016 (4)	0.026 (3)	
H12	0.469307	0.751271	0.582793	0.031*	
C13	0.5540 (6)	0.7550 (6)	0.6243 (5)	0.021 (3)	
H13	0.558600	0.800528	0.621009	0.025*	
C14	0.6024 (5)	0.7180 (5)	0.6527 (4)	0.013 (2)	
C15	0.7880 (5)	0.8198 (5)	0.7232 (4)	0.013 (2)	
C16	0.8144 (5)	0.8846 (5)	0.7244 (4)	0.014 (2)	
C17	0.7798 (6)	0.9380 (6)	0.7316 (5)	0.025 (3)	
H17	0.739672	0.931194	0.735802	0.030*	
C18	0.8013 (7)	0.9997 (7)	0.7329 (7)	0.039 (4)	
H18	0.776025	1.035410	0.736473	0.047*	
C19	0.8613 (7)	1.0102 (7)	0.7288 (7)	0.048 (5)	
H19	0.877157	1.053035	0.731110	0.057*	
C20	0.8970 (6)	0.9588 (6)	0.7216 (6)	0.032 (3)	
H20	0.937377	0.966620	0.718518	0.038*	
C21	0.8748 (6)	0.8943 (6)	0.7187 (5)	0.017 (3)	
C22	0.9978 (5)	0.7007 (5)	0.7035 (4)	0.0130 (19)	
C23	1.0550 (5)	0.6757 (5)	0.6926 (4)	0.018 (2)	
C24	1.0885 (6)	0.7157 (6)	0.6682 (5)	0.026 (3)	
H24	1.073091	0.757303	0.657293	0.032*	
C25	1.1434 (6)	0.6969 (6)	0.6593 (5)	0.031 (3)	
H25	1.166092	0.726036	0.643647	0.037*	
C26	1.1657 (6)	0.6355 (6)	0.6733 (5)	0.031 (3)	
H26	1.203657	0.622253	0.667043	0.037*	
C27	1.1320 (5)	0.5935 (6)	0.6964 (4)	0.023 (2)	
H27	1.147210	0.551367	0.705922	0.027*	
C28	1.0762 (5)	0.6123 (5)	0.7060 (4)	0.015 (2)	
C29	0.9779 (4)	0.6117 (4)	0.8453 (3)	0.0113 (12)	
C30	0.9984 (5)	0.6372 (5)	0.8959 (4)	0.014 (2)	
C31	1.0610 (6)	0.6431 (7)	0.9149 (4)	0.021 (3)	
H31	1.088774	0.630288	0.895115	0.026*	
C32	1.0820 (6)	0.6672 (6)	0.9616 (4)	0.029 (3)	
H32	1.124017	0.670666	0.974292	0.035*	
C33	1.0418 (5)	0.6863 (6)	0.9897 (4)	0.026 (3)	
H33	1.056304	0.703006	1.021945	0.031*	
C34	0.9806 (5)	0.6817 (6)	0.9722 (4)	0.022 (2)	
H34	0.953847	0.696025	0.992346	0.026*	
C35	0.9570 (5)	0.6562 (5)	0.9251 (4)	0.015 (2)	
C36	0.7481 (5)	0.6819 (5)	0.8401 (3)	0.0125 (19)	
C37	0.6967 (5)	0.7174 (5)	0.8524 (4)	0.016 (2)	
C38	0.6925 (6)	0.7229 (6)	0.9029 (4)	0.024 (3)	
H38	0.722868	0.703562	0.927418	0.029*	
C39	0.6464 (7)	0.7549 (6)	0.9172 (5)	0.037 (3)	
H39	0.643835	0.757128	0.951122	0.045*	
C40	0.6028 (6)	0.7844 (7)	0.8808 (5)	0.033 (3)	
H40	0.570726	0.807634	0.890290	0.040*	
C41	0.6055 (5)	0.7803 (6)	0.8316 (4)	0.024 (3)	
H41	0.575336	0.801078	0.807729	0.029*	
C42	0.6520 (5)	0.7461 (5)	0.8157 (4)	0.014 (2)	
C43	0.9485 (4)	0.5107 (5)	0.6267 (4)	0.015 (2)	
C44	0.9494 (5)	0.5173 (5)	0.5727 (4)	0.016 (2)	
C45	0.9879 (5)	0.4759 (6)	0.5535 (4)	0.023 (2)	
H45	1.013734	0.447373	0.575040	0.027*	
C46	0.9888 (6)	0.4760 (6)	0.5035 (4)	0.025 (3)	
H46	1.016200	0.449010	0.490949	0.030*	
C47	0.9493 (5)	0.5159 (6)	0.4719 (4)	0.026 (3)	
H47	0.948962	0.515252	0.437442	0.031*	
C48	0.9106 (5)	0.5564 (5)	0.4899 (4)	0.022 (2)	
H48	0.883712	0.583256	0.467778	0.026*	
C49	0.9103 (5)	0.5585 (5)	0.5412 (4)	0.017 (2)	
C50	0.8344 (5)	0.7550 (5)	0.5916 (4)	0.013 (2)	
C51	0.8016 (5)	0.7736 (5)	0.5425 (4)	0.013 (2)	
C52	0.8041 (5)	0.8376 (5)	0.5263 (4)	0.017 (2)	
H52	0.827245	0.868285	0.547867	0.020*	
C53	0.7746 (6)	0.8582 (5)	0.4803 (4)	0.024 (3)	
H53	0.776827	0.902285	0.470632	0.029*	
C54	0.7418 (6)	0.8135 (6)	0.4487 (4)	0.028 (3)	
H54	0.722060	0.826780	0.416533	0.034*	
C55	0.7371 (5)	0.7494 (5)	0.4631 (4)	0.021 (2)	
H55	0.713264	0.719797	0.441042	0.025*	
C56	0.7670 (5)	0.7275 (5)	0.5099 (4)	0.015 (2)	
C57	0.7523 (4)	0.4771 (5)	0.7802 (4)	0.0125 (19)	
C58	0.7468 (5)	0.4691 (5)	0.8323 (3)	0.014 (2)	
C59	0.6987 (5)	0.4318 (5)	0.8412 (4)	0.016 (2)	
H59	0.670856	0.413611	0.814270	0.019*	
C60	0.6914 (5)	0.4214 (5)	0.8889 (4)	0.019 (2)	
H60	0.659653	0.394548	0.895047	0.023*	
C61	0.7310 (5)	0.4505 (5)	0.9282 (4)	0.018 (2)	
H61	0.725666	0.443745	0.961056	0.021*	
C62	0.7775 (5)	0.4887 (5)	0.9196 (4)	0.018 (2)	
H62	0.803690	0.508715	0.946744	0.021*	
C63	0.7871 (5)	0.4989 (4)	0.8709 (3)	0.0144 (19)	
C64	0.9689 (5)	0.4441 (5)	0.7957 (4)	0.022 (2)	
C65	1.0021 (7)	0.4358 (7)	0.8481 (5)	0.040 (3)	
H65A	1.043408	0.421758	0.848250	0.061*	
H65B	0.981740	0.402905	0.864514	0.061*	
H65C	1.002766	0.477218	0.865643	0.061*	
C66	1.1602 (7)	0.8014 (7)	0.7554 (5)	0.039 (3)	
H66	1.192535	0.805669	0.738469	0.047*	
C67	1.1205 (8)	0.7435 (8)	0.8180 (7)	0.050 (5)	
H67A	1.087533	0.774766	0.808819	0.075*	
H67B	1.138025	0.747820	0.853263	0.075*	
H67C	1.104849	0.699283	0.811224	0.075*	
C68	1.2188 (9)	0.7116 (9)	0.7975 (7)	0.070 (6)	
H68A	1.205087	0.666740	0.790663	0.105*	
H68B	1.240703	0.715039	0.831770	0.105*	
H68C	1.245396	0.723881	0.775104	0.105*	
C69	0.9581 (6)	0.8132 (7)	0.8223 (4)	0.029 (3)	
H69	0.957129	0.768613	0.813219	0.035*	
C70	0.9093 (7)	0.9047 (6)	0.8537 (6)	0.039 (3)	
H70A	0.877963	0.926767	0.829753	0.059*	
H70B	0.948294	0.925197	0.853800	0.059*	
H70C	0.899885	0.908055	0.886771	0.059*	
C71	0.8620 (6)	0.7961 (7)	0.8424 (5)	0.038 (3)	
H71A	0.826583	0.811761	0.818882	0.057*	
H71B	0.853755	0.797035	0.875890	0.057*	
H71C	0.871066	0.751473	0.833903	0.057*	
C72	0.6357 (6)	0.9474 (6)	0.6244 (5)	0.028 (3)	
H72	0.622986	0.990969	0.617473	0.033*	
C73	0.6968 (6)	0.8569 (6)	0.6045 (5)	0.027 (3)	
H73A	0.679443	0.834435	0.629671	0.040*	
H73B	0.740389	0.860960	0.616000	0.040*	
H73C	0.688369	0.831932	0.573681	0.040*	
C74	0.6854 (10)	0.9574 (8)	0.5547 (8)	0.071 (7)	
H74A	0.674687	0.931695	0.524336	0.107*	
H74B	0.728733	0.966502	0.561437	0.107*	
H74C	0.663064	0.998448	0.550633	0.107*	
C75	0.6406 (6)	0.9670 (6)	0.8089 (5)	0.031 (3)	
H75	0.614808	1.003541	0.807873	0.037*	
C76	0.7229 (6)	0.9033 (7)	0.8557 (5)	0.035 (3)	
H76A	0.720848	0.882860	0.823401	0.053*	
H76B	0.713128	0.871145	0.879068	0.053*	
H76C	0.763640	0.920042	0.867940	0.053*	
C77	0.6779 (9)	0.9924 (10)	0.8959 (7)	0.070 (6)	
H77A	0.710598	1.024322	0.902093	0.106*	
H77B	0.682043	0.962479	0.923959	0.106*	
H77C	0.639115	1.014962	0.891667	0.106*	
C78	0.4793 (6)	0.8156 (6)	0.7013 (5)	0.026 (3)	
H78	0.451431	0.842310	0.679786	0.031*	
C79	0.5006 (7)	0.7083 (6)	0.7389 (6)	0.036 (3)	
H79A	0.496500	0.666811	0.721066	0.055*	
H79B	0.489728	0.702399	0.771176	0.055*	
H79C	0.542348	0.723365	0.743508	0.055*	
C80	0.4006 (7)	0.7340 (7)	0.6904 (6)	0.046 (4)	
H80A	0.378516	0.768024	0.669335	0.070*	
H80B	0.379954	0.724264	0.717454	0.070*	
H80C	0.402380	0.694732	0.670681	0.070*	
C81	0.5602 (5)	0.4858 (6)	0.8038 (4)	0.024 (3)	
H81	0.546116	0.460836	0.828146	0.029*	
C82	0.6105 (6)	0.5832 (6)	0.7820 (4)	0.027 (3)	
H82A	0.594490	0.567796	0.748308	0.041*	
H82B	0.654559	0.580397	0.788780	0.041*	
H82C	0.598346	0.628439	0.785083	0.041*	
C83	0.6018 (7)	0.5630 (6)	0.8689 (4)	0.034 (3)	
H83A	0.645604	0.566553	0.879459	0.051*	
H83B	0.586577	0.530707	0.889448	0.051*	
H83C	0.583262	0.605234	0.872570	0.051*	
C84	0.4933 (8)	0.3843 (9)	0.6166 (5)	0.054 (5)	
H84	0.467832	0.348393	0.619404	0.065*	
C85	0.5255 (9)	0.4660 (7)	0.5639 (6)	0.053 (5)	
H85A	0.520059	0.474908	0.528424	0.080*	
H85B	0.509768	0.502585	0.580236	0.080*	
H85C	0.568412	0.460381	0.577958	0.080*	
C86	0.4538 (10)	0.3788 (11)	0.5272 (7)	0.081 (7)	
H86A	0.459963	0.401852	0.497580	0.122*	
H86B	0.463771	0.332748	0.524604	0.122*	
H86C	0.411659	0.382932	0.530212	0.122*	
C87	0.8458 (6)	0.3854 (6)	0.5893 (4)	0.030 (3)	
H87	0.866311	0.423162	0.581622	0.036*	
C88	0.7893 (7)	0.2882 (7)	0.5596 (6)	0.038 (3)	
H88A	0.750115	0.288871	0.536801	0.057*	
H88B	0.783216	0.287638	0.593712	0.057*	
H88C	0.811615	0.249342	0.553442	0.057*	
C89	0.8286 (6)	0.3607 (7)	0.5017 (5)	0.038 (3)	
H89A	0.840335	0.321532	0.485824	0.057*	
H89B	0.859272	0.394378	0.502338	0.057*	
H89C	0.789856	0.376503	0.483040	0.057*	
O38	0.6116 (10)	0.0975 (13)	0.5449 (9)	0.163 (9)	
C90	0.5674 (10)	0.1235 (13)	0.5622 (9)	0.105 (7)	
H90	0.560013	0.110993	0.593570	0.126*	
N18	0.5335 (8)	0.1669 (11)	0.5356 (7)	0.096 (5)	
C91	0.5347 (13)	0.1772 (15)	0.4844 (9)	0.119 (9)	
H91A	0.506240	0.211692	0.471173	0.178*	
H91B	0.575386	0.189993	0.481214	0.178*	
H91C	0.523384	0.136936	0.465961	0.178*	
C92	0.4858 (8)	0.2058 (11)	0.5529 (8)	0.080 (7)	
H92A	0.466587	0.235125	0.526388	0.120*	
H92B	0.455575	0.176390	0.561498	0.120*	
H92C	0.504129	0.231350	0.581949	0.120*	
C93	0.3831 (11)	0.5280 (12)	0.9186 (10)	0.083 (7)	
H93	0.420159	0.510455	0.935776	0.099*	
C94	0.3322 (12)	0.5958 (14)	0.8572 (12)	0.138 (13)	
H94A	0.339993	0.623435	0.830301	0.206*	
H94B	0.314964	0.622055	0.880491	0.206*	
H94C	0.303737	0.561386	0.843535	0.206*	
C95	0.4424 (12)	0.5799 (14)	0.8670 (14)	0.129 (12)	
H95A	0.436283	0.610419	0.839263	0.194*	
H95B	0.471011	0.598363	0.894952	0.194*	
H95C	0.458331	0.538843	0.856992	0.194*	
C96	0.5703 (10)	0.4431 (10)	0.9748 (8)	0.064 (5)	
H96	0.595702	0.469458	0.998441	0.076*	
C97	0.5573 (14)	0.3382 (11)	0.9386 (9)	0.110 (10)	
H97A	0.587241	0.303852	0.938734	0.166*	
H97B	0.552477	0.362947	0.907770	0.166*	
H97C	0.518633	0.318589	0.941220	0.166*	
C98	0.6153 (8)	0.3516 (11)	1.0221 (8)	0.058 (5)	
H98A	0.639059	0.384608	1.042997	0.087*	
H98B	0.642370	0.321241	1.010062	0.087*	
H98C	0.590432	0.327877	1.041331	0.087*	

### Poly[[µ3-acetato-hexakis(µ-N,N-dimethylformamide)bis(N,N-dimethylformamide)bis[salicylhydroximato(2-)]heptakis[salicylhydroximato(3-)]hexaaluminium(III)dysprosium(III)pentasodium(I)] N,N-dimethylformamide tetrasolvate monohydrate] (1) Atomic displacement parameters (Å^2^)

**Table:**

	*U*^11^	*U*^22^	*U*^33^	*U*^12^	*U*^13^	*U*^23^
Dy1	0.01184 (19)	0.01240 (17)	0.00799 (16)	0.0002 (3)	0.00181 (14)	0.0003 (2)
Al1	0.0154 (17)	0.0128 (14)	0.0055 (14)	−0.0020 (15)	0.0032 (12)	0.0003 (11)
Al2	0.0135 (14)	0.0125 (13)	0.0089 (13)	−0.0015 (13)	0.0026 (12)	0.0017 (10)
Al3	0.0122 (14)	0.0107 (12)	0.0113 (13)	0.0031 (12)	0.0019 (11)	0.0005 (10)
Al4	0.0104 (14)	0.0126 (14)	0.0121 (14)	−0.0009 (14)	0.0027 (12)	−0.0009 (11)
Al5	0.0106 (12)	0.0143 (12)	0.0092 (12)	0.0044 (12)	0.0011 (10)	0.0007 (9)
Al6	0.0138 (16)	0.0106 (14)	0.0062 (14)	0.0021 (14)	−0.0002 (12)	0.0002 (10)
Na1	0.016 (2)	0.020 (2)	0.018 (2)	−0.006 (2)	0.0031 (18)	0.0004 (16)
Na2	0.018 (2)	0.019 (2)	0.017 (2)	0.009 (2)	0.0036 (18)	0.0020 (16)
Na3	0.019 (2)	0.023 (2)	0.020 (2)	−0.007 (2)	0.0059 (19)	0.0026 (17)
Na4	0.025 (2)	0.023 (2)	0.022 (2)	0.004 (2)	0.0086 (19)	−0.0033 (17)
Na5	0.020 (2)	0.021 (2)	0.020 (2)	0.007 (2)	0.0063 (19)	−0.0002 (17)
O1	0.01184 (19)	0.01240 (17)	0.00799 (16)	0.0002 (3)	0.00181 (14)	0.0003 (2)
O2	0.022 (4)	0.016 (3)	0.009 (3)	−0.006 (3)	0.005 (3)	0.002 (2)
O3	0.021 (4)	0.012 (3)	0.011 (3)	0.001 (3)	0.005 (3)	−0.001 (3)
O4	0.011 (3)	0.007 (3)	0.013 (3)	−0.002 (3)	0.004 (3)	0.002 (2)
O5	0.009 (3)	0.019 (3)	0.011 (3)	−0.004 (3)	−0.001 (3)	−0.002 (2)
O6	0.016 (4)	0.012 (3)	0.012 (3)	0.002 (3)	0.004 (3)	0.002 (3)
O7	0.0122 (14)	0.0107 (12)	0.0113 (13)	0.0031 (12)	0.0019 (11)	0.0005 (10)
O8	0.012 (4)	0.014 (3)	0.014 (3)	0.003 (3)	0.000 (3)	0.001 (3)
O9	0.014 (4)	0.016 (4)	0.021 (4)	−0.004 (4)	0.008 (3)	−0.004 (3)
O10	0.007 (3)	0.021 (3)	0.011 (3)	0.004 (3)	0.000 (2)	−0.001 (3)
O11	0.009 (3)	0.015 (4)	0.014 (3)	−0.001 (3)	−0.001 (3)	0.002 (3)
O12	0.017 (4)	0.015 (3)	0.013 (3)	0.004 (3)	0.006 (3)	0.003 (3)
O13	0.0106 (12)	0.0143 (12)	0.0092 (12)	0.0044 (12)	0.0011 (10)	0.0007 (9)
O14	0.011 (3)	0.025 (4)	0.013 (3)	0.001 (3)	0.003 (2)	−0.003 (3)
O15	0.016 (4)	0.024 (4)	0.010 (4)	0.004 (4)	0.000 (3)	0.000 (3)
O16	0.009 (3)	0.014 (3)	0.015 (3)	0.005 (3)	0.004 (3)	0.001 (3)
O17	0.019 (4)	0.017 (4)	0.010 (3)	0.004 (3)	0.000 (3)	0.006 (3)
O18	0.013 (3)	0.018 (4)	0.013 (3)	0.004 (3)	0.005 (3)	0.003 (3)
O19	0.014 (3)	0.008 (3)	0.015 (3)	0.006 (3)	−0.001 (3)	0.001 (2)
O20	0.024 (4)	0.020 (4)	0.017 (4)	0.006 (4)	0.001 (3)	−0.002 (3)
O21	0.017 (4)	0.015 (3)	0.016 (4)	0.001 (3)	0.005 (3)	0.000 (3)
O22	0.011 (3)	0.015 (3)	0.004 (3)	0.000 (3)	0.003 (3)	0.001 (2)
O23	0.017 (4)	0.016 (3)	0.013 (3)	−0.005 (3)	0.002 (3)	0.001 (3)
O24	0.023 (4)	0.016 (4)	0.011 (4)	−0.009 (4)	0.000 (3)	0.003 (3)
O25	0.014 (3)	0.016 (3)	0.006 (3)	−0.003 (3)	0.003 (3)	0.002 (2)
O26	0.018 (4)	0.019 (4)	0.007 (3)	−0.010 (3)	−0.001 (3)	0.005 (3)
O27	0.013 (3)	0.018 (3)	0.012 (3)	−0.003 (3)	0.001 (3)	0.000 (2)
O28	0.016 (4)	0.018 (4)	0.015 (3)	0.009 (3)	0.004 (3)	0.007 (3)
O29	0.030 (5)	0.045 (5)	0.040 (6)	−0.001 (5)	0.006 (4)	0.009 (4)
O30	0.022 (4)	0.022 (4)	0.030 (5)	0.000 (4)	−0.001 (4)	0.004 (3)
O31	0.031 (5)	0.059 (7)	0.019 (5)	−0.008 (6)	0.003 (4)	0.001 (4)
O32	0.024 (4)	0.020 (4)	0.025 (4)	0.008 (4)	0.011 (4)	0.008 (3)
O33	0.026 (5)	0.026 (4)	0.020 (4)	0.010 (4)	0.002 (4)	−0.004 (3)
O34	0.022 (4)	0.022 (4)	0.028 (4)	0.006 (4)	0.007 (4)	−0.003 (3)
O35	0.021 (4)	0.029 (4)	0.023 (4)	−0.005 (4)	0.004 (4)	−0.007 (3)
O36	0.024 (4)	0.034 (5)	0.018 (4)	−0.011 (4)	0.004 (4)	0.002 (3)
O37	0.032 (5)	0.041 (5)	0.023 (4)	−0.003 (5)	0.000 (4)	−0.003 (4)
O39	0.085 (11)	0.079 (10)	0.103 (12)	0.021 (10)	0.015 (10)	−0.034 (9)
O40	0.094 (11)	0.063 (8)	0.075 (10)	−0.024 (9)	0.015 (9)	0.004 (7)
O41	0.115 (17)	0.087 (16)	0.095 (17)	0.015 (15)	0.034 (15)	0.012 (13)
C99	0.083 (11)	0.074 (10)	0.075 (11)	0.008 (10)	0.020 (10)	0.000 (9)
N21	0.081 (9)	0.079 (9)	0.077 (10)	0.010 (8)	0.018 (9)	0.010 (8)
C100	0.085 (15)	0.100 (14)	0.083 (15)	0.023 (14)	0.014 (13)	0.025 (13)
C101	0.077 (18)	0.079 (16)	0.076 (16)	0.013 (15)	0.010 (16)	0.004 (14)
O41B	0.113 (18)	0.117 (17)	0.118 (18)	0.021 (16)	0.028 (15)	0.040 (15)
C99B	0.083 (11)	0.085 (10)	0.081 (11)	0.013 (10)	0.014 (10)	0.014 (10)
N21B	0.081 (9)	0.078 (9)	0.078 (10)	0.010 (8)	0.016 (9)	0.007 (8)
C102	0.078 (14)	0.085 (14)	0.073 (14)	0.012 (13)	0.036 (12)	0.005 (12)
C103	0.066 (17)	0.062 (15)	0.058 (15)	0.014 (14)	0.021 (14)	0.012 (13)
O42	0.102 (12)	0.091 (10)	0.091 (12)	0.034 (10)	0.033 (10)	0.036 (9)
N1	0.012 (4)	0.010 (4)	0.012 (4)	0.000 (3)	0.006 (3)	−0.002 (3)
N2	0.006 (3)	0.016 (3)	0.012 (3)	0.001 (3)	0.001 (3)	−0.001 (3)
N3	0.012 (4)	0.014 (3)	0.008 (4)	0.001 (3)	0.001 (3)	0.001 (3)
N4	0.007 (3)	0.013 (3)	0.020 (4)	−0.001 (3)	0.004 (3)	−0.004 (3)
N5	0.012 (3)	0.013 (3)	0.008 (2)	0.003 (2)	0.000 (2)	0.0011 (19)
N6	0.016 (4)	0.016 (4)	0.016 (4)	0.001 (4)	0.006 (4)	0.001 (3)
N7	0.018 (4)	0.024 (4)	0.009 (4)	−0.001 (4)	0.005 (3)	0.001 (3)
N8	0.013 (4)	0.014 (4)	0.008 (4)	−0.003 (4)	−0.002 (3)	0.002 (3)
N9	0.023 (5)	0.012 (4)	0.009 (4)	0.002 (4)	0.007 (3)	0.003 (3)
N10	0.038 (7)	0.049 (7)	0.033 (6)	0.015 (7)	0.000 (6)	−0.001 (5)
N11	0.035 (6)	0.030 (6)	0.034 (6)	−0.007 (6)	0.009 (5)	−0.001 (5)
N12	0.043 (7)	0.025 (5)	0.029 (6)	0.008 (6)	0.022 (5)	0.005 (4)
N13	0.032 (6)	0.036 (6)	0.026 (6)	0.017 (6)	−0.009 (5)	−0.010 (5)
N14	0.027 (6)	0.028 (5)	0.034 (6)	0.003 (5)	0.010 (5)	0.000 (4)
N15	0.025 (5)	0.025 (5)	0.014 (4)	−0.004 (5)	0.006 (4)	−0.004 (3)
N16	0.064 (10)	0.068 (9)	0.035 (7)	−0.020 (9)	0.003 (7)	−0.002 (6)
N17	0.026 (5)	0.031 (5)	0.024 (5)	−0.005 (5)	0.006 (5)	−0.001 (4)
N19	0.081 (13)	0.096 (13)	0.056 (11)	0.019 (13)	0.009 (10)	−0.013 (10)
N20	0.085 (12)	0.059 (9)	0.038 (8)	−0.041 (9)	−0.008 (8)	0.011 (6)
C1	0.013 (5)	0.008 (4)	0.009 (4)	−0.001 (4)	−0.002 (4)	0.001 (3)
C2	0.012 (5)	0.014 (5)	0.015 (5)	−0.003 (4)	0.001 (4)	−0.001 (4)
C3	0.024 (6)	0.022 (5)	0.017 (5)	−0.008 (5)	0.005 (5)	−0.005 (4)
C4	0.039 (7)	0.029 (6)	0.012 (5)	−0.023 (6)	0.001 (5)	−0.003 (4)
C5	0.031 (7)	0.020 (6)	0.023 (6)	−0.016 (6)	0.006 (5)	−0.010 (4)
C6	0.023 (6)	0.021 (6)	0.020 (5)	−0.004 (5)	0.004 (5)	−0.004 (4)
C7	0.017 (5)	0.020 (5)	0.008 (5)	0.001 (5)	−0.001 (4)	−0.006 (4)
C8	0.007 (3)	0.019 (3)	0.013 (4)	−0.002 (3)	0.001 (3)	−0.002 (3)
C9	0.013 (4)	0.018 (5)	0.020 (5)	0.002 (4)	0.005 (4)	0.002 (4)
C10	0.015 (5)	0.022 (6)	0.028 (6)	−0.002 (6)	−0.006 (5)	−0.002 (5)
C11	0.016 (6)	0.035 (7)	0.035 (7)	−0.001 (6)	−0.011 (5)	−0.001 (6)
C12	0.020 (6)	0.031 (6)	0.024 (6)	−0.002 (6)	−0.006 (5)	0.001 (5)
C13	0.020 (6)	0.017 (6)	0.025 (6)	0.009 (6)	0.001 (5)	0.007 (4)
C14	0.009 (5)	0.016 (5)	0.015 (5)	−0.003 (5)	0.003 (4)	−0.004 (4)
C15	0.008 (5)	0.017 (5)	0.014 (5)	0.002 (5)	−0.001 (4)	−0.001 (4)
C16	0.006 (5)	0.014 (6)	0.021 (6)	−0.002 (5)	−0.004 (4)	−0.001 (4)
C17	0.009 (6)	0.018 (6)	0.046 (8)	−0.005 (6)	0.002 (6)	−0.001 (5)
C18	0.024 (7)	0.018 (7)	0.076 (11)	−0.002 (7)	0.009 (8)	−0.010 (7)
C19	0.027 (8)	0.018 (6)	0.102 (15)	−0.011 (7)	0.019 (9)	−0.005 (7)
C20	0.017 (6)	0.017 (6)	0.061 (10)	0.000 (6)	0.004 (7)	−0.003 (6)
C21	0.009 (6)	0.011 (5)	0.031 (7)	0.001 (6)	0.005 (5)	−0.002 (5)
C22	0.010 (4)	0.014 (4)	0.015 (4)	−0.004 (4)	0.001 (4)	−0.006 (3)
C23	0.012 (5)	0.021 (6)	0.024 (6)	0.000 (5)	0.008 (5)	−0.003 (4)
C24	0.030 (7)	0.023 (6)	0.031 (6)	0.001 (6)	0.017 (6)	0.002 (5)
C25	0.022 (6)	0.023 (6)	0.054 (9)	0.001 (6)	0.022 (6)	0.006 (6)
C26	0.017 (6)	0.036 (7)	0.043 (8)	0.008 (6)	0.016 (6)	−0.004 (6)
C27	0.019 (6)	0.019 (6)	0.032 (6)	0.001 (6)	0.009 (5)	−0.002 (5)
C28	0.017 (5)	0.014 (5)	0.014 (5)	−0.001 (5)	0.004 (4)	0.001 (4)
C29	0.012 (3)	0.013 (3)	0.008 (2)	0.003 (2)	0.000 (2)	0.0011 (19)
C30	0.013 (5)	0.019 (5)	0.009 (3)	−0.004 (4)	0.000 (3)	0.002 (3)
C31	0.015 (6)	0.036 (7)	0.011 (5)	−0.009 (6)	0.000 (5)	−0.003 (5)
C32	0.022 (6)	0.047 (8)	0.015 (5)	−0.012 (7)	−0.003 (5)	−0.004 (5)
C33	0.021 (6)	0.040 (7)	0.015 (5)	−0.006 (6)	0.001 (5)	−0.009 (5)
C34	0.017 (5)	0.033 (6)	0.014 (5)	−0.005 (5)	−0.001 (4)	−0.009 (4)
C35	0.018 (5)	0.014 (5)	0.010 (4)	0.000 (5)	−0.001 (4)	−0.002 (3)
C36	0.014 (5)	0.012 (4)	0.010 (4)	−0.002 (4)	−0.001 (4)	−0.003 (3)
C37	0.020 (6)	0.014 (5)	0.015 (5)	0.006 (5)	0.006 (5)	0.004 (4)
C38	0.032 (7)	0.026 (6)	0.014 (5)	0.008 (6)	0.003 (5)	−0.001 (4)
C39	0.054 (9)	0.044 (8)	0.018 (6)	0.025 (8)	0.018 (6)	0.011 (5)
C40	0.029 (7)	0.050 (8)	0.024 (6)	0.023 (7)	0.013 (5)	0.014 (5)
C41	0.025 (6)	0.030 (6)	0.019 (6)	0.014 (6)	0.008 (5)	0.005 (4)
C42	0.012 (5)	0.016 (5)	0.014 (5)	0.006 (5)	0.003 (4)	0.002 (4)
C43	0.010 (5)	0.020 (5)	0.012 (5)	0.008 (4)	−0.002 (4)	−0.003 (4)
C44	0.018 (5)	0.020 (5)	0.009 (4)	0.002 (5)	0.002 (4)	−0.004 (4)
C45	0.018 (5)	0.031 (6)	0.018 (5)	0.005 (5)	0.001 (5)	−0.004 (4)
C46	0.026 (6)	0.028 (6)	0.023 (6)	0.003 (6)	0.012 (5)	−0.012 (4)
C47	0.029 (6)	0.037 (7)	0.011 (5)	0.009 (6)	0.005 (5)	−0.004 (4)
C48	0.024 (6)	0.023 (5)	0.017 (5)	−0.004 (5)	0.002 (5)	−0.003 (4)
C49	0.016 (5)	0.018 (5)	0.018 (5)	0.003 (4)	0.005 (4)	−0.001 (4)
C50	0.015 (5)	0.015 (5)	0.011 (5)	0.004 (5)	0.004 (4)	−0.001 (3)
C51	0.009 (5)	0.020 (5)	0.012 (5)	0.004 (5)	0.002 (4)	0.003 (4)
C52	0.020 (6)	0.015 (5)	0.015 (5)	0.004 (5)	0.002 (4)	0.006 (4)
C53	0.027 (6)	0.016 (5)	0.028 (6)	0.000 (6)	0.001 (5)	0.014 (4)
C54	0.030 (7)	0.035 (7)	0.020 (6)	0.009 (7)	0.003 (5)	0.012 (5)
C55	0.018 (5)	0.026 (6)	0.017 (5)	0.007 (5)	−0.002 (5)	0.009 (4)
C56	0.018 (5)	0.015 (5)	0.012 (5)	0.000 (5)	0.005 (4)	0.001 (3)
C57	0.010 (5)	0.015 (5)	0.012 (4)	−0.003 (4)	0.002 (4)	0.003 (3)
C58	0.018 (5)	0.018 (5)	0.006 (4)	−0.009 (4)	0.005 (4)	0.002 (3)
C59	0.014 (5)	0.018 (5)	0.015 (5)	−0.002 (5)	0.001 (4)	0.002 (4)
C60	0.019 (5)	0.025 (5)	0.017 (5)	0.003 (5)	0.008 (4)	0.008 (4)
C61	0.022 (6)	0.020 (5)	0.012 (5)	0.000 (5)	0.005 (4)	0.003 (4)
C62	0.022 (5)	0.019 (5)	0.009 (4)	0.001 (5)	−0.004 (4)	−0.001 (3)
C63	0.020 (5)	0.013 (4)	0.010 (4)	−0.001 (4)	0.002 (4)	0.002 (3)
C64	0.022 (6)	0.021 (6)	0.024 (6)	0.006 (5)	0.008 (5)	0.007 (4)
C65	0.051 (9)	0.053 (9)	0.015 (6)	0.004 (8)	0.000 (6)	0.012 (6)
C66	0.042 (8)	0.043 (8)	0.032 (7)	−0.007 (8)	0.009 (7)	−0.005 (6)
C67	0.049 (10)	0.047 (10)	0.051 (10)	0.001 (9)	−0.001 (8)	0.015 (8)
C68	0.078 (14)	0.069 (12)	0.057 (12)	0.035 (12)	0.000 (11)	0.016 (9)
C69	0.031 (7)	0.034 (7)	0.021 (6)	−0.004 (7)	0.001 (5)	−0.004 (5)
C70	0.041 (8)	0.029 (7)	0.044 (8)	0.014 (8)	−0.002 (7)	−0.009 (6)
C71	0.035 (8)	0.043 (8)	0.035 (8)	−0.020 (8)	0.004 (6)	0.003 (6)
C72	0.035 (7)	0.017 (6)	0.035 (7)	0.008 (6)	0.016 (6)	0.003 (5)
C73	0.031 (7)	0.020 (6)	0.035 (7)	0.014 (6)	0.020 (6)	0.006 (5)
C74	0.110 (17)	0.037 (9)	0.090 (14)	0.032 (11)	0.076 (14)	0.031 (9)
C75	0.028 (7)	0.025 (6)	0.036 (7)	0.006 (6)	0.000 (6)	−0.012 (5)
C76	0.030 (7)	0.044 (8)	0.025 (7)	0.002 (8)	−0.012 (6)	−0.002 (6)
C77	0.068 (12)	0.081 (13)	0.052 (11)	0.026 (12)	−0.011 (9)	−0.043 (10)
C78	0.025 (6)	0.023 (6)	0.033 (7)	0.001 (6)	0.011 (6)	0.003 (5)
C79	0.036 (8)	0.031 (7)	0.043 (8)	−0.002 (7)	0.011 (7)	0.005 (6)
C80	0.043 (9)	0.043 (8)	0.047 (9)	−0.008 (8)	−0.006 (7)	0.006 (7)
C81	0.022 (6)	0.027 (6)	0.026 (6)	0.001 (6)	0.009 (5)	0.004 (5)
C82	0.038 (7)	0.024 (6)	0.022 (6)	−0.008 (6)	0.010 (5)	−0.004 (4)
C83	0.054 (9)	0.030 (7)	0.018 (6)	−0.012 (7)	0.003 (6)	−0.005 (5)
C84	0.048 (9)	0.089 (13)	0.024 (7)	−0.032 (10)	0.003 (7)	0.011 (8)
C85	0.092 (14)	0.042 (8)	0.034 (8)	−0.022 (10)	0.034 (9)	0.012 (6)
C86	0.085 (15)	0.119 (18)	0.033 (9)	−0.015 (15)	−0.003 (10)	−0.009 (10)
C87	0.033 (6)	0.033 (6)	0.022 (6)	−0.005 (6)	0.001 (5)	0.003 (5)
C88	0.041 (8)	0.029 (7)	0.045 (9)	−0.008 (7)	0.010 (7)	−0.001 (6)
C89	0.033 (7)	0.062 (9)	0.022 (6)	−0.008 (7)	0.012 (6)	−0.002 (6)
O38	0.114 (15)	0.22 (2)	0.145 (18)	0.032 (16)	0.013 (14)	−0.092 (16)
C90	0.068 (12)	0.161 (18)	0.080 (13)	−0.006 (13)	0.000 (10)	−0.039 (12)
N18	0.064 (10)	0.161 (15)	0.059 (10)	−0.014 (11)	0.001 (8)	−0.025 (10)
C91	0.115 (18)	0.17 (2)	0.082 (16)	−0.025 (18)	0.036 (15)	0.006 (16)
C92	0.045 (10)	0.122 (17)	0.073 (13)	−0.018 (12)	0.011 (10)	−0.056 (12)
C93	0.061 (14)	0.087 (17)	0.097 (19)	0.026 (15)	0.008 (14)	−0.043 (14)
C94	0.089 (19)	0.16 (3)	0.16 (3)	0.08 (2)	0.00 (2)	0.08 (2)
C95	0.084 (19)	0.10 (2)	0.20 (4)	0.001 (19)	0.04 (2)	0.04 (2)
C96	0.069 (13)	0.064 (12)	0.064 (13)	−0.017 (12)	0.028 (11)	−0.021 (10)
C97	0.15 (3)	0.087 (17)	0.079 (18)	−0.042 (18)	−0.019 (17)	0.010 (13)
C98	0.025 (9)	0.074 (13)	0.073 (14)	0.004 (10)	0.005 (9)	−0.004 (11)

### Poly[[µ3-acetato-hexakis(µ-N,N-dimethylformamide)bis(N,N-dimethylformamide)bis[salicylhydroximato(2-)]heptakis[salicylhydroximato(3-)]hexaaluminium(III)dysprosium(III)pentasodium(I)] N,N-dimethylformamide tetrasolvate monohydrate] (1) Geometric parameters (Å, º)

**Table:**

Dy1—O7	2.297 (7)	N19—C95	1.44 (3)
Dy1—O4	2.327 (6)	N20—C96	1.27 (2)
Dy1—O13	2.364 (7)	N20—C98	1.42 (2)
Dy1—O22	2.398 (6)	N20—C97	1.48 (3)
Dy1—O16	2.427 (7)	C1—C2	1.448 (13)
Dy1—O1	2.430 (6)	C2—C7	1.409 (14)
Dy1—O10	2.439 (7)	C2—C3	1.417 (14)
Dy1—O19	2.454 (7)	C3—C4	1.385 (15)
Dy1—O25	2.643 (6)	C3—H3	0.9500
Dy1—Al5	3.369 (3)	C4—C5	1.389 (15)
Dy1—Al4	3.383 (3)	C4—H4	0.9500
Dy1—Al2	3.445 (3)	C5—C6	1.384 (15)
Al1—O21	1.748 (8)	C5—H5	0.9500
Al1—O24	1.766 (8)	C6—C7	1.400 (15)
Al1—O2	1.855 (7)	C6—H6	0.9500
Al1—O1	1.883 (7)	C8—C9	1.464 (15)
Al1—N8	1.930 (9)	C9—C10	1.385 (16)
Al2—O3	1.823 (7)	C9—C14	1.411 (15)
Al2—O4	1.864 (7)	C10—C11	1.370 (17)
Al2—O5	1.903 (8)	C10—H10	0.9500
Al2—O26	1.909 (7)	C11—C12	1.371 (18)
Al2—N1	1.948 (8)	C11—H11	0.9500
Al2—O25	1.954 (7)	C12—C13	1.381 (18)
Al2—Na3	3.000 (5)	C12—H12	0.9500
Al3—O18	1.843 (7)	C13—C14	1.433 (15)
Al3—O7	1.884 (8)	C13—H13	0.9500
Al3—O6	1.889 (8)	C15—C16	1.460 (15)
Al3—O8	1.902 (8)	C16—C17	1.389 (17)
Al3—N2	1.957 (9)	C16—C21	1.424 (15)
Al3—N6	2.078 (9)	C17—C18	1.358 (18)
Al3—Na2	2.977 (5)	C17—H17	0.9500
Al4—O9	1.834 (8)	C18—C19	1.40 (2)
Al4—O11	1.885 (8)	C18—H18	0.9500
Al4—O23	1.900 (8)	C19—C20	1.371 (19)
Al4—O22	1.902 (7)	C19—H19	0.9500
Al4—O10	1.939 (8)	C20—C21	1.416 (17)
Al4—N3	1.982 (9)	C20—H20	0.9500
Al4—Na1	3.382 (5)	C22—C23	1.481 (15)
Al5—O12	1.802 (7)	C23—C24	1.384 (15)
Al5—O14	1.884 (8)	C23—C28	1.414 (15)
Al5—O13	1.896 (7)	C24—C25	1.371 (17)
Al5—O28	1.903 (7)	C24—H24	0.9500
Al5—O19	1.929 (7)	C25—C26	1.388 (18)
Al5—N4	2.054 (9)	C25—H25	0.9500
Al5—Na5	3.291 (5)	C26—C27	1.392 (17)
Al5—Na4	3.548 (5)	C26—H26	0.9500
Al6—O15	1.781 (9)	C27—C28	1.400 (15)
Al6—O27	1.790 (7)	C27—H27	0.9500
Al6—O17	1.819 (8)	C29—C30	1.477 (13)
Al6—O16	1.881 (8)	C30—C35	1.411 (14)
Al6—N5	1.934 (9)	C30—C31	1.415 (16)
Na1—O30	2.325 (10)	C31—C32	1.377 (16)
Na1—O9	2.325 (9)	C31—H31	0.9500
Na1—O31	2.367 (10)	C32—C33	1.368 (17)
Na1—O35^i^	2.429 (9)	C32—H32	0.9500
Na1—O36^i^	2.458 (9)	C33—C34	1.377 (16)
Na1—O11	2.550 (8)	C33—H33	0.9500
Na1—Na3^i^	3.057 (6)	C34—C35	1.404 (13)
Na1—C69	3.101 (13)	C34—H34	0.9500
Na2—O33	2.319 (9)	C36—C37	1.472 (14)
Na2—O34	2.327 (9)	C37—C42	1.410 (14)
Na2—O32	2.346 (8)	C37—C38	1.423 (14)
Na2—O6	2.384 (8)	C38—C39	1.361 (17)
Na2—O8	2.394 (8)	C38—H38	0.9500
Na2—O18	2.399 (8)	C39—C40	1.399 (18)
Na2—Na5^ii^	2.969 (6)	C39—H39	0.9500
Na3—O36	2.247 (10)	C40—C41	1.377 (16)
Na3—O26	2.330 (8)	C40—H40	0.9500
Na3—O35	2.342 (9)	C41—C42	1.409 (14)
Na3—O30^iii^	2.373 (9)	C41—H41	0.9500
Na3—O3	2.516 (8)	C43—C44	1.504 (13)
Na3—O5	2.555 (8)	C44—C45	1.396 (15)
Na4—O29	2.319 (10)	C44—C49	1.398 (15)
Na4—O25	2.327 (8)	C45—C46	1.386 (15)
Na4—O37	2.331 (9)	C45—H45	0.9500
Na4—O20	2.403 (9)	C46—C47	1.387 (17)
Na4—O19	2.426 (8)	C46—H46	0.9500
Na4—O28	2.826 (9)	C47—C48	1.373 (16)
Na4—C64	2.931 (13)	C47—H47	0.9500
Na4—C43	2.982 (11)	C48—C49	1.420 (14)
Na4—Na5	3.625 (6)	C48—H48	0.9500
Na5—O20	2.305 (9)	C50—C51	1.462 (14)
Na5—O28	2.362 (8)	C51—C52	1.397 (14)
Na5—O33^iv^	2.369 (10)	C51—C56	1.429 (15)
Na5—O32^iv^	2.398 (9)	C52—C53	1.377 (15)
Na5—O12	2.411 (8)	C52—H52	0.9500
Na5—O34^iv^	2.416 (9)	C53—C54	1.380 (18)
Na5—C43	3.106 (11)	C53—H53	0.9500
Na5—C75^iv^	3.116 (14)	C54—C55	1.389 (16)
O1—N1	1.397 (10)	C54—H54	0.9500
O2—C1	1.315 (11)	C55—C56	1.408 (14)
O3—C7	1.352 (12)	C55—H55	0.9500
O4—N2	1.404 (10)	C57—C58	1.479 (12)
O5—C8	1.328 (12)	C58—C59	1.395 (14)
O6—C14	1.315 (13)	C58—C63	1.399 (14)
O7—N3	1.410 (11)	C59—C60	1.377 (13)
O8—C15	1.315 (13)	C59—H59	0.9500
O9—C21	1.319 (14)	C60—C61	1.396 (15)
O10—N4	1.424 (11)	C60—H60	0.9500
O11—C22	1.320 (13)	C61—C62	1.375 (15)
O12—C28	1.341 (12)	C61—H61	0.9500
O13—N5	1.405 (10)	C62—C63	1.421 (13)
O14—C29	1.289 (11)	C62—H62	0.9500
O15—C35	1.318 (13)	C64—C65	1.500 (17)
O16—N6	1.421 (11)	C65—H65A	0.9800
O17—C36	1.306 (12)	C65—H65B	0.9800
O18—C42	1.329 (12)	C65—H65C	0.9800
O19—N7	1.381 (10)	C66—H66	0.9500
O20—C43	1.250 (12)	C67—H67A	0.9800
O21—C49	1.334 (12)	C67—H67B	0.9800
O22—N8	1.416 (10)	C67—H67C	0.9800
O23—C50	1.318 (12)	C68—H68A	0.9800
O24—C56	1.335 (12)	C68—H68B	0.9800
O25—N9	1.394 (9)	C68—H68C	0.9800
O26—C57	1.284 (12)	C69—H69	0.9500
O27—C63	1.353 (12)	C70—H70A	0.9800
O28—C64	1.286 (13)	C70—H70B	0.9800
O29—C64	1.248 (15)	C70—H70C	0.9800
O30—C66	1.236 (15)	C71—H71A	0.9800
O31—C69	1.228 (15)	C71—H71B	0.9800
O32—C72	1.234 (14)	C71—H71C	0.9800
O33—C75	1.236 (14)	C72—H72	0.9500
O34—C78	1.249 (15)	C73—H73A	0.9800
O35—C81	1.244 (14)	C73—H73B	0.9800
O36—C84	1.236 (18)	C73—H73C	0.9800
O37—C87	1.251 (14)	C74—H74A	0.9800
O39—C93	1.29 (3)	C74—H74B	0.9800
O40—C96	1.27 (2)	C74—H74C	0.9800
O41—C99	1.27 (3)	C75—H75	0.9500
C99—N21	1.31 (3)	C76—H76A	0.9800
C99—H99	0.9500	C76—H76B	0.9800
N21—C100	1.45 (3)	C76—H76C	0.9800
N21—C101	1.46 (3)	C77—H77A	0.9800
C100—H10G	0.9800	C77—H77B	0.9800
C100—H10H	0.9800	C77—H77C	0.9800
C100—H10I	0.9800	C78—H78	0.9500
C101—H10J	0.9800	C79—H79A	0.9800
C101—H10K	0.9800	C79—H79B	0.9800
C101—H10L	0.9800	C79—H79C	0.9800
O41B—C99B	1.29 (3)	C80—H80A	0.9800
C99B—N21B	1.33 (3)	C80—H80B	0.9800
C99B—H99B	0.9500	C80—H80C	0.9800
N21B—C102	1.45 (3)	C81—H81	0.9500
N21B—C103	1.46 (3)	C82—H82A	0.9800
C102—H10M	0.9800	C82—H82B	0.9800
C102—H10N	0.9800	C82—H82C	0.9800
C102—H10O	0.9800	C83—H83A	0.9800
C103—H10P	0.9800	C83—H83B	0.9800
C103—H10Q	0.9800	C83—H83C	0.9800
C103—H10R	0.9800	C84—H84	0.9500
O42—H42A	0.8399	C85—H85A	0.9800
O42—H42B	0.8440	C85—H85B	0.9800
N1—C1	1.322 (12)	C85—H85C	0.9800
N2—C8	1.295 (13)	C86—H86A	0.9800
N3—C15	1.316 (13)	C86—H86B	0.9800
N4—C22	1.300 (13)	C86—H86C	0.9800
N5—C29	1.328 (12)	C87—H87	0.9500
N6—C36	1.318 (12)	C88—H88A	0.9800
N7—C43	1.338 (12)	C88—H88B	0.9800
N7—H7	0.90 (3)	C88—H88C	0.9800
N8—C50	1.293 (13)	C89—H89A	0.9800
N9—C57	1.311 (13)	C89—H89B	0.9800
N9—H9	0.91 (3)	C89—H89C	0.9800
N10—C66	1.319 (16)	O38—C90	1.31 (2)
N10—C67	1.432 (18)	C90—N18	1.30 (2)
N10—C68	1.492 (18)	C90—H90	0.9500
N11—C69	1.338 (17)	N18—C91	1.43 (2)
N11—C71	1.414 (17)	N18—C92	1.50 (2)
N11—C70	1.455 (16)	C91—H91A	0.9800
N12—C72	1.333 (15)	C91—H91B	0.9800
N12—C73	1.450 (15)	C91—H91C	0.9800
N12—C74	1.461 (17)	C92—H92A	0.9800
N13—C75	1.344 (17)	C92—H92B	0.9800
N13—C76	1.434 (17)	C92—H92C	0.9800
N13—C77	1.461 (18)	C93—H93	0.9500
N14—C78	1.332 (15)	C94—H94A	0.9800
N14—C80	1.441 (18)	C94—H94B	0.9800
N14—C79	1.457 (17)	C94—H94C	0.9800
N15—C81	1.338 (15)	C95—H95A	0.9800
N15—C83	1.458 (14)	C95—H95B	0.9800
N15—C82	1.463 (14)	C95—H95C	0.9800
N16—C84	1.335 (19)	C96—H96	0.9500
N16—C85	1.45 (2)	C97—H97A	0.9800
N16—C86	1.48 (2)	C97—H97B	0.9800
N17—C87	1.341 (16)	C97—H97C	0.9800
N17—C88	1.444 (17)	C98—H98A	0.9800
N17—C89	1.450 (15)	C98—H98B	0.9800
N19—C93	1.32 (3)	C98—H98C	0.9800
N19—C94	1.39 (3)		
			
O7—Dy1—O4	73.6 (2)	Na2—O33—Na5^ii^	78.6 (3)
O7—Dy1—O13	136.2 (2)	C78—O34—Na2	149.6 (7)
O4—Dy1—O13	113.6 (2)	C78—O34—Na5^ii^	120.9 (8)
O7—Dy1—O22	72.4 (2)	Na2—O34—Na5^ii^	77.5 (3)
O4—Dy1—O22	126.8 (2)	C81—O35—Na3	138.3 (8)
O13—Dy1—O22	119.3 (2)	C81—O35—Na1^iii^	126.1 (8)
O7—Dy1—O16	73.0 (2)	Na3—O35—Na1^iii^	79.7 (3)
O4—Dy1—O16	71.8 (2)	C84—O36—Na3	153.2 (10)
O13—Dy1—O16	69.4 (2)	C84—O36—Na1^iii^	118.5 (9)
O22—Dy1—O16	131.9 (2)	Na3—O36—Na1^iii^	80.9 (3)
O7—Dy1—O1	89.4 (2)	C87—O37—Na4	124.8 (8)
O4—Dy1—O1	73.4 (2)	O41—C99—N21	125 (3)
O13—Dy1—O1	134.4 (2)	O41—C99—H99	117.5
O22—Dy1—O1	66.6 (2)	N21—C99—H99	117.5
O16—Dy1—O1	144.2 (2)	C99—N21—C100	125 (3)
O7—Dy1—O10	81.0 (3)	C99—N21—C101	121 (3)
O4—Dy1—O10	148.0 (2)	C100—N21—C101	113 (3)
O13—Dy1—O10	72.7 (2)	N21—C100—H10G	109.5
O22—Dy1—O10	60.0 (2)	N21—C100—H10H	109.5
O16—Dy1—O10	82.5 (2)	H10G—C100—H10H	109.5
O1—Dy1—O10	126.2 (2)	N21—C100—H10I	109.5
O7—Dy1—O19	149.8 (2)	H10G—C100—H10I	109.5
O4—Dy1—O19	128.7 (2)	H10H—C100—H10I	109.5
O13—Dy1—O19	60.0 (2)	N21—C101—H10J	109.5
O22—Dy1—O19	77.4 (2)	N21—C101—H10K	109.5
O16—Dy1—O19	129.4 (2)	H10J—C101—H10K	109.5
O1—Dy1—O19	80.2 (2)	N21—C101—H10L	109.5
O10—Dy1—O19	82.5 (2)	H10J—C101—H10L	109.5
O7—Dy1—O25	131.2 (2)	H10K—C101—H10L	109.5
O4—Dy1—O25	58.0 (2)	O41B—C99B—N21B	121 (3)
O13—Dy1—O25	74.6 (2)	O41B—C99B—H99B	119.3
O22—Dy1—O25	132.7 (2)	N21B—C99B—H99B	119.3
O16—Dy1—O25	95.3 (2)	C99B—N21B—C102	121 (3)
O1—Dy1—O25	72.9 (2)	C99B—N21B—C103	124 (3)
O10—Dy1—O25	145.7 (2)	C102—N21B—C103	113 (3)
O19—Dy1—O25	72.6 (2)	N21B—C102—H10M	109.5
O7—Dy1—Al5	140.62 (19)	N21B—C102—H10N	109.5
O4—Dy1—Al5	141.39 (16)	H10M—C102—H10N	109.5
O13—Dy1—Al5	33.10 (17)	N21B—C102—H10O	109.5
O22—Dy1—Al5	88.15 (17)	H10M—C102—H10O	109.5
O16—Dy1—Al5	98.22 (17)	H10N—C102—H10O	109.5
O1—Dy1—Al5	114.30 (16)	N21B—C103—H10P	109.5
O10—Dy1—Al5	59.68 (17)	N21B—C103—H10Q	109.5
O19—Dy1—Al5	34.33 (17)	H10P—C103—H10Q	109.5
O25—Dy1—Al5	87.11 (16)	N21B—C103—H10R	109.5
O7—Dy1—Al4	58.53 (19)	H10P—C103—H10R	109.5
O4—Dy1—Al4	131.45 (17)	H10Q—C103—H10R	109.5
O13—Dy1—Al4	106.51 (17)	H42A—O42—H42B	108.6
O22—Dy1—Al4	33.20 (17)	C1—N1—O1	112.3 (7)
O16—Dy1—Al4	99.34 (17)	C1—N1—Al2	129.0 (7)
O1—Dy1—Al4	97.26 (16)	O1—N1—Al2	117.8 (5)
O10—Dy1—Al4	34.29 (17)	C8—N2—O4	110.4 (8)
O19—Dy1—Al4	94.52 (16)	C8—N2—Al3	133.5 (7)
O25—Dy1—Al4	164.69 (14)	O4—N2—Al3	115.9 (6)
Al5—Dy1—Al4	86.44 (7)	C15—N3—O7	110.2 (8)
O7—Dy1—Al2	98.41 (19)	C15—N3—Al4	131.0 (7)
O4—Dy1—Al2	30.54 (16)	O7—N3—Al4	118.8 (5)
O13—Dy1—Al2	106.82 (17)	C22—N4—O10	111.9 (8)
O22—Dy1—Al2	121.50 (17)	C22—N4—Al5	127.9 (7)
O16—Dy1—Al2	95.72 (17)	O10—N4—Al5	118.1 (6)
O1—Dy1—Al2	55.48 (16)	C29—N5—O13	111.8 (7)
O10—Dy1—Al2	178.18 (16)	C29—N5—Al6	130.8 (7)
O19—Dy1—Al2	98.76 (17)	O13—N5—Al6	117.4 (6)
O25—Dy1—Al2	34.36 (16)	C36—N6—O16	109.6 (8)
Al5—Dy1—Al2	120.85 (7)	C36—N6—Al3	129.3 (7)
Al4—Dy1—Al2	146.46 (7)	O16—N6—Al3	121.0 (6)
O21—Al1—O24	111.3 (4)	C43—N7—O19	118.9 (8)
O21—Al1—O2	102.3 (4)	C43—N7—H7	117 (4)
O24—Al1—O2	93.2 (4)	O19—N7—H7	117 (6)
O21—Al1—O1	113.5 (4)	C50—N8—O22	111.6 (8)
O24—Al1—O1	134.9 (4)	C50—N8—Al1	131.5 (7)
O2—Al1—O1	82.5 (3)	O22—N8—Al1	115.6 (5)
O21—Al1—N8	91.0 (4)	C57—N9—O25	116.5 (8)
O24—Al1—N8	90.2 (4)	C57—N9—H9	111 (4)
O2—Al1—N8	164.0 (4)	O25—N9—H9	130 (5)
O1—Al1—N8	84.0 (3)	C66—N10—C67	123.0 (13)
O3—Al2—O4	172.1 (4)	C66—N10—C68	119.6 (13)
O3—Al2—O5	90.9 (3)	C67—N10—C68	117.2 (13)
O4—Al2—O5	82.0 (3)	C69—N11—C71	119.8 (12)
O3—Al2—O26	85.8 (3)	C69—N11—C70	121.0 (12)
O4—Al2—O26	91.5 (3)	C71—N11—C70	118.9 (12)
O5—Al2—O26	94.7 (3)	C72—N12—C73	123.2 (10)
O3—Al2—N1	89.8 (3)	C72—N12—C74	120.7 (11)
O4—Al2—N1	94.4 (3)	C73—N12—C74	116.1 (10)
O5—Al2—N1	98.8 (3)	C75—N13—C76	122.0 (10)
O26—Al2—N1	165.9 (4)	C75—N13—C77	120.7 (12)
O3—Al2—O25	108.1 (3)	C76—N13—C77	117.0 (12)
O4—Al2—O25	78.8 (3)	C78—N14—C80	121.9 (12)
O5—Al2—O25	160.5 (3)	C78—N14—C79	122.7 (12)
O26—Al2—O25	82.7 (3)	C80—N14—C79	115.4 (11)
N1—Al2—O25	86.0 (3)	C81—N15—C83	121.6 (10)
O3—Al2—Na3	56.8 (3)	C81—N15—C82	121.2 (9)
O4—Al2—Na3	116.1 (2)	C83—N15—C82	116.6 (10)
O5—Al2—Na3	57.7 (2)	C84—N16—C85	121.6 (14)
O26—Al2—Na3	51.0 (2)	C84—N16—C86	121.2 (16)
N1—Al2—Na3	135.3 (3)	C85—N16—C86	116.7 (15)
O25—Al2—Na3	129.5 (2)	C87—N17—C88	121.4 (11)
O3—Al2—Dy1	148.3 (3)	C87—N17—C89	120.6 (11)
O4—Al2—Dy1	39.4 (2)	C88—N17—C89	117.9 (11)
O5—Al2—Dy1	114.4 (2)	C93—N19—C94	117 (2)
O26—Al2—Dy1	109.4 (2)	C93—N19—C95	120 (2)
N1—Al2—Dy1	68.6 (2)	C94—N19—C95	122 (2)
O25—Al2—Dy1	49.8 (2)	C96—N20—C98	126.2 (17)
Na3—Al2—Dy1	153.20 (13)	C96—N20—C97	118.7 (17)
O18—Al3—O7	167.2 (4)	C98—N20—C97	113.6 (18)
O18—Al3—O6	86.1 (3)	O2—C1—N1	117.6 (8)
O7—Al3—O6	102.3 (3)	O2—C1—C2	121.6 (8)
O18—Al3—O8	89.6 (3)	N1—C1—C2	120.8 (8)
O7—Al3—O8	81.0 (3)	C7—C2—C3	118.6 (9)
O6—Al3—O8	88.6 (3)	C7—C2—C1	122.8 (9)
O18—Al3—N2	106.4 (4)	C3—C2—C1	118.7 (9)
O7—Al3—N2	84.3 (3)	C4—C3—C2	121.4 (10)
O6—Al3—N2	84.3 (3)	C4—C3—H3	119.3
O8—Al3—N2	162.0 (4)	C2—C3—H3	119.3
O18—Al3—N6	86.4 (3)	C3—C4—C5	119.1 (10)
O7—Al3—N6	86.6 (3)	C3—C4—H4	120.4
O6—Al3—N6	168.7 (4)	C5—C4—H4	120.4
O8—Al3—N6	99.8 (4)	C6—C5—C4	120.7 (10)
N2—Al3—N6	89.7 (4)	C6—C5—H5	119.6
O18—Al3—Na2	53.7 (2)	C4—C5—H5	119.6
O7—Al3—Na2	124.3 (3)	C5—C6—C7	120.9 (10)
O6—Al3—Na2	53.1 (2)	C5—C6—H6	119.5
O8—Al3—Na2	53.4 (2)	C7—C6—H6	119.5
N2—Al3—Na2	130.7 (3)	O3—C7—C6	117.7 (9)
N6—Al3—Na2	127.0 (3)	O3—C7—C2	123.1 (9)
O9—Al4—O11	88.1 (3)	C6—C7—C2	119.2 (9)
O9—Al4—O23	92.9 (3)	N2—C8—O5	120.6 (9)
O11—Al4—O23	98.5 (3)	N2—C8—C9	119.4 (9)
O9—Al4—O22	173.6 (4)	O5—C8—C9	119.9 (9)
O11—Al4—O22	95.9 (3)	C10—C9—C14	119.8 (10)
O23—Al4—O22	81.6 (3)	C10—C9—C8	120.8 (10)
O9—Al4—O10	107.6 (4)	C14—C9—C8	119.1 (10)
O11—Al4—O10	81.3 (3)	C11—C10—C9	122.1 (11)
O23—Al4—O10	159.5 (3)	C11—C10—H10	119.0
O22—Al4—O10	78.0 (3)	C9—C10—H10	119.0
O9—Al4—N3	87.5 (3)	C10—C11—C12	119.8 (12)
O11—Al4—N3	165.5 (4)	C10—C11—H11	120.1
O23—Al4—N3	95.4 (3)	C12—C11—H11	120.1
O22—Al4—N3	89.8 (3)	C11—C12—C13	120.0 (12)
O10—Al4—N3	86.9 (3)	C11—C12—H12	120.0
O9—Al4—Na1	40.8 (3)	C13—C12—H12	120.0
O11—Al4—Na1	48.3 (2)	C12—C13—C14	121.5 (11)
O23—Al4—Na1	104.8 (2)	C12—C13—H13	119.2
O22—Al4—Na1	143.9 (3)	C14—C13—H13	119.2
O10—Al4—Na1	90.5 (2)	O6—C14—C9	124.5 (10)
N3—Al4—Na1	124.0 (3)	O6—C14—C13	118.9 (10)
O9—Al4—Dy1	139.0 (3)	C9—C14—C13	116.6 (10)
O11—Al4—Dy1	111.0 (2)	O8—C15—N3	119.6 (9)
O23—Al4—Dy1	118.1 (2)	O8—C15—C16	120.7 (10)
O22—Al4—Dy1	43.64 (19)	N3—C15—C16	119.7 (9)
O10—Al4—Dy1	45.1 (2)	C17—C16—C21	119.3 (11)
N3—Al4—Dy1	64.8 (2)	C17—C16—C15	119.0 (10)
Na1—Al4—Dy1	135.63 (12)	C21—C16—C15	121.7 (11)
O12—Al5—O14	99.0 (3)	C18—C17—C16	122.2 (12)
O12—Al5—O13	177.3 (4)	C18—C17—H17	118.9
O14—Al5—O13	83.3 (3)	C16—C17—H17	118.9
O12—Al5—O28	86.4 (3)	C17—C18—C19	119.4 (13)
O14—Al5—O28	93.4 (3)	C17—C18—H18	120.3
O13—Al5—O28	92.1 (3)	C19—C18—H18	120.3
O12—Al5—O19	99.6 (3)	C20—C19—C18	120.2 (13)
O14—Al5—O19	161.0 (3)	C20—C19—H19	119.9
O13—Al5—O19	78.1 (3)	C18—C19—H19	119.9
O28—Al5—O19	91.4 (3)	C19—C20—C21	121.1 (12)
O12—Al5—N4	90.0 (3)	C19—C20—H20	119.4
O14—Al5—N4	91.3 (3)	C21—C20—H20	119.4
O13—Al5—N4	91.3 (3)	O9—C21—C20	119.9 (11)
O28—Al5—N4	174.5 (3)	O9—C21—C16	122.4 (11)
O19—Al5—N4	85.2 (3)	C20—C21—C16	117.7 (12)
O12—Al5—Na5	45.8 (2)	N4—C22—O11	119.8 (9)
O14—Al5—Na5	113.9 (3)	N4—C22—C23	120.9 (10)
O13—Al5—Na5	131.9 (2)	O11—C22—C23	119.4 (9)
O28—Al5—Na5	44.9 (2)	C24—C23—C28	119.0 (10)
O19—Al5—Na5	82.1 (2)	C24—C23—C22	118.8 (10)
N4—Al5—Na5	130.1 (3)	C28—C23—C22	122.2 (9)
O12—Al5—Dy1	136.1 (3)	C25—C24—C23	121.7 (12)
O14—Al5—Dy1	116.1 (2)	C25—C24—H24	119.1
O13—Al5—Dy1	42.9 (2)	C23—C24—H24	119.1
O28—Al5—Dy1	115.3 (3)	C24—C25—C26	120.2 (12)
O19—Al5—Dy1	45.9 (2)	C24—C25—H25	119.9
N4—Al5—Dy1	65.0 (2)	C26—C25—H25	119.9
Na5—Al5—Dy1	126.97 (12)	C25—C26—C27	119.3 (11)
O12—Al5—Na4	104.5 (3)	C25—C26—H26	120.4
O14—Al5—Na4	136.1 (3)	C27—C26—H26	120.4
O13—Al5—Na4	72.9 (2)	C26—C27—C28	121.1 (11)
O28—Al5—Na4	52.5 (2)	C26—C27—H27	119.5
O19—Al5—Na4	40.4 (2)	C28—C27—H27	119.5
N4—Al5—Na4	124.9 (3)	O12—C28—C27	116.9 (9)
Na5—Al5—Na4	63.88 (12)	O12—C28—C23	124.5 (9)
Dy1—Al5—Na4	68.39 (9)	C27—C28—C23	118.6 (10)
O15—Al6—O27	115.3 (4)	O14—C29—N5	120.5 (8)
O15—Al6—O17	90.6 (4)	O14—C29—C30	119.6 (9)
O27—Al6—O17	100.2 (4)	N5—C29—C30	119.8 (8)
O15—Al6—O16	132.8 (4)	C35—C30—C31	119.8 (9)
O27—Al6—O16	111.8 (3)	C35—C30—C29	121.4 (9)
O17—Al6—O16	83.4 (3)	C31—C30—C29	118.9 (9)
O15—Al6—N5	90.1 (4)	C32—C31—C30	120.7 (11)
O27—Al6—N5	93.8 (3)	C32—C31—H31	119.6
O17—Al6—N5	164.2 (4)	C30—C31—H31	119.6
O16—Al6—N5	84.5 (3)	C33—C32—C31	119.4 (12)
O30—Na1—O9	162.9 (3)	C33—C32—H32	120.3
O30—Na1—O31	101.3 (4)	C31—C32—H32	120.3
O9—Na1—O31	85.5 (3)	C32—C33—C34	121.3 (10)
O30—Na1—O35^i^	79.8 (3)	C32—C33—H33	119.3
O9—Na1—O35^i^	116.8 (3)	C34—C33—H33	119.3
O31—Na1—O35^i^	84.3 (3)	C33—C34—C35	121.4 (10)
O30—Na1—O36^i^	83.9 (3)	C33—C34—H34	119.3
O9—Na1—O36^i^	94.6 (3)	C35—C34—H34	119.3
O31—Na1—O36^i^	161.2 (4)	O15—C35—C34	119.0 (9)
O35^i^—Na1—O36^i^	78.8 (3)	O15—C35—C30	123.6 (9)
O30—Na1—O11	99.0 (3)	C34—C35—C30	117.4 (10)
O9—Na1—O11	63.9 (3)	O17—C36—N6	120.2 (9)
O31—Na1—O11	110.4 (3)	O17—C36—C37	118.1 (9)
O35^i^—Na1—O11	165.0 (3)	N6—C36—C37	121.5 (9)
O36^i^—Na1—O11	86.2 (3)	C42—C37—C38	119.5 (10)
O30—Na1—Na3^i^	50.1 (2)	C42—C37—C36	122.1 (9)
O9—Na1—Na3^i^	137.0 (3)	C38—C37—C36	118.4 (10)
O31—Na1—Na3^i^	124.7 (3)	C39—C38—C37	122.0 (11)
O35^i^—Na1—Na3^i^	48.9 (2)	C39—C38—H38	119.0
O36^i^—Na1—Na3^i^	46.5 (2)	C37—C38—H38	119.0
O11—Na1—Na3^i^	119.3 (2)	C38—C39—C40	118.3 (11)
O30—Na1—C69	115.7 (4)	C38—C39—H39	120.8
O9—Na1—C69	67.3 (3)	C40—C39—H39	120.8
O31—Na1—C69	20.9 (3)	C41—C40—C39	121.2 (11)
O35^i^—Na1—C69	101.7 (3)	C41—C40—H40	119.4
O36^i^—Na1—C69	160.2 (4)	C39—C40—H40	119.4
O11—Na1—C69	92.3 (3)	C40—C41—C42	121.5 (11)
Na3^i^—Na1—C69	145.5 (3)	C40—C41—H41	119.3
O30—Na1—Al4	132.0 (3)	C42—C41—H41	119.3
O9—Na1—Al4	31.00 (19)	O18—C42—C41	119.7 (9)
O31—Na1—Al4	94.5 (3)	O18—C42—C37	122.9 (9)
O35^i^—Na1—Al4	147.3 (3)	C41—C42—C37	117.4 (9)
O36^i^—Na1—Al4	95.2 (2)	O20—C43—N7	121.6 (9)
O11—Na1—Al4	33.46 (18)	O20—C43—C44	121.1 (9)
Na3^i^—Na1—Al4	140.74 (17)	N7—C43—C44	117.3 (9)
C69—Na1—Al4	73.5 (3)	O20—C43—Na4	51.1 (5)
O33—Na2—O34	86.5 (3)	N7—C43—Na4	81.4 (6)
O33—Na2—O32	82.0 (3)	C44—C43—Na4	143.6 (7)
O34—Na2—O32	89.6 (3)	O20—C43—Na5	40.6 (5)
O33—Na2—O6	169.3 (4)	N7—C43—Na5	100.7 (6)
O34—Na2—O6	104.0 (3)	C44—C43—Na5	127.2 (6)
O32—Na2—O6	99.7 (3)	Na4—C43—Na5	73.1 (3)
O33—Na2—O8	102.1 (3)	C45—C44—C49	120.2 (9)
O34—Na2—O8	161.3 (3)	C45—C44—C43	117.2 (9)
O32—Na2—O8	107.9 (3)	C49—C44—C43	122.4 (9)
O6—Na2—O8	67.3 (3)	C46—C45—C44	120.8 (11)
O33—Na2—O18	113.5 (3)	C46—C45—H45	119.6
O34—Na2—O18	94.6 (3)	C44—C45—H45	119.6
O32—Na2—O18	164.1 (3)	C45—C46—C47	119.4 (10)
O6—Na2—O18	64.4 (2)	C45—C46—H46	120.3
O8—Na2—O18	66.8 (3)	C47—C46—H46	120.3
O33—Na2—Na5^ii^	51.5 (2)	C48—C47—C46	120.6 (10)
O34—Na2—Na5^ii^	52.6 (2)	C48—C47—H47	119.7
O32—Na2—Na5^ii^	52.0 (2)	C46—C47—H47	119.7
O6—Na2—Na5^ii^	137.2 (3)	C47—C48—C49	120.9 (11)
O8—Na2—Na5^ii^	144.6 (3)	C47—C48—H48	119.6
O18—Na2—Na5^ii^	140.4 (2)	C49—C48—H48	119.6
O33—Na2—Al3	132.4 (3)	O21—C49—C44	122.0 (9)
O34—Na2—Al3	123.5 (3)	O21—C49—C48	119.9 (9)
O32—Na2—Al3	128.3 (2)	C44—C49—C48	118.1 (9)
O6—Na2—Al3	39.34 (19)	N8—C50—O23	120.2 (9)
O8—Na2—Al3	39.66 (19)	N8—C50—C51	119.1 (9)
O18—Na2—Al3	38.24 (18)	O23—C50—C51	120.8 (9)
Na5^ii^—Na2—Al3	175.5 (2)	C52—C51—C56	118.5 (9)
O36—Na3—O26	173.8 (4)	C52—C51—C50	119.9 (9)
O36—Na3—O35	85.0 (3)	C56—C51—C50	121.5 (9)
O26—Na3—O35	101.0 (3)	C53—C52—C51	122.8 (10)
O36—Na3—O30^iii^	87.6 (3)	C53—C52—H52	118.6
O26—Na3—O30^iii^	91.9 (3)	C51—C52—H52	118.6
O35—Na3—O30^iii^	80.6 (3)	C52—C53—C54	118.7 (10)
O36—Na3—O3	110.9 (3)	C52—C53—H53	120.7
O26—Na3—O3	63.1 (3)	C54—C53—H53	120.7
O35—Na3—O3	163.9 (3)	C53—C54—C55	120.9 (11)
O30^iii^—Na3—O3	101.5 (3)	C53—C54—H54	119.5
O36—Na3—O5	109.6 (3)	C55—C54—H54	119.5
O26—Na3—O5	69.9 (3)	C54—C55—C56	121.2 (11)
O35—Na3—O5	110.5 (3)	C54—C55—H55	119.4
O30^iii^—Na3—O5	159.9 (3)	C56—C55—H55	119.4
O3—Na3—O5	63.1 (2)	O24—C56—C55	118.6 (10)
O36—Na3—Al2	136.6 (3)	O24—C56—C51	123.6 (9)
O26—Na3—Al2	39.52 (18)	C55—C56—C51	117.9 (9)
O35—Na3—Al2	128.3 (3)	O26—C57—N9	118.6 (9)
O30^iii^—Na3—Al2	120.9 (3)	O26—C57—C58	121.2 (9)
O3—Na3—Al2	37.32 (17)	N9—C57—C58	120.2 (9)
O5—Na3—Al2	39.04 (18)	C59—C58—C63	121.5 (8)
O36—Na3—Na1^iii^	52.6 (2)	C59—C58—C57	117.0 (9)
O26—Na3—Na1^iii^	130.6 (3)	C63—C58—C57	121.5 (8)
O35—Na3—Na1^iii^	51.4 (2)	C60—C59—C58	120.1 (10)
O30^iii^—Na3—Na1^iii^	48.7 (2)	C60—C59—H59	119.9
O3—Na3—Na1^iii^	140.5 (2)	C58—C59—H59	119.9
O5—Na3—Na1^iii^	151.0 (3)	C59—C60—C61	119.7 (10)
Al2—Na3—Na1^iii^	168.9 (2)	C59—C60—H60	120.2
O29—Na4—O25	91.8 (3)	C61—C60—H60	120.2
O29—Na4—O37	126.4 (4)	C62—C61—C60	120.5 (9)
O25—Na4—O37	103.0 (3)	C62—C61—H61	119.7
O29—Na4—O20	116.4 (3)	C60—C61—H61	119.7
O25—Na4—O20	140.0 (3)	C61—C62—C63	121.0 (9)
O37—Na4—O20	83.3 (3)	C61—C62—H62	119.5
O29—Na4—O19	104.7 (3)	C63—C62—H62	119.5
O25—Na4—O19	78.9 (3)	O27—C63—C58	121.9 (8)
O37—Na4—O19	128.5 (3)	O27—C63—C62	120.8 (9)
O20—Na4—O19	67.3 (3)	C58—C63—C62	117.2 (9)
O29—Na4—O28	49.8 (3)	O29—C64—O28	121.7 (11)
O25—Na4—O28	107.0 (3)	O29—C64—C65	120.0 (11)
O37—Na4—O28	149.9 (3)	O28—C64—C65	118.2 (11)
O20—Na4—O28	76.2 (3)	O29—C64—Na4	49.3 (6)
O19—Na4—O28	62.4 (2)	O28—C64—Na4	72.5 (6)
O29—Na4—C64	24.1 (3)	C65—C64—Na4	169.3 (9)
O25—Na4—C64	99.2 (3)	C64—C65—H65A	109.5
O37—Na4—C64	143.7 (4)	C64—C65—H65B	109.5
O20—Na4—C64	97.7 (3)	H65A—C65—H65B	109.5
O19—Na4—C64	83.7 (3)	C64—C65—H65C	109.5
O28—Na4—C64	25.7 (3)	H65A—C65—H65C	109.5
O29—Na4—C43	131.5 (3)	H65B—C65—H65C	109.5
O25—Na4—C43	116.1 (3)	O30—C66—N10	126.7 (13)
O37—Na4—C43	86.9 (3)	O30—C66—H66	116.7
O20—Na4—C43	23.9 (2)	N10—C66—H66	116.7
O19—Na4—C43	50.0 (3)	N10—C67—H67A	109.5
O28—Na4—C43	83.2 (3)	N10—C67—H67B	109.5
C64—Na4—C43	108.5 (3)	H67A—C67—H67B	109.5
O29—Na4—Al5	74.9 (3)	N10—C67—H67C	109.5
O25—Na4—Al5	88.0 (2)	H67A—C67—H67C	109.5
O37—Na4—Al5	154.8 (3)	H67B—C67—H67C	109.5
O20—Na4—Al5	74.1 (2)	N10—C68—H68A	109.5
O19—Na4—Al5	31.01 (18)	N10—C68—H68B	109.5
O28—Na4—Al5	32.29 (15)	H68A—C68—H68B	109.5
C64—Na4—Al5	52.8 (2)	N10—C68—H68C	109.5
C43—Na4—Al5	67.9 (2)	H68A—C68—H68C	109.5
O29—Na4—Na5	78.4 (3)	H68B—C68—H68C	109.5
O25—Na4—Na5	142.6 (2)	O31—C69—N11	124.4 (13)
O37—Na4—Na5	111.9 (3)	O31—C69—Na1	43.5 (6)
O20—Na4—Na5	38.7 (2)	N11—C69—Na1	131.7 (9)
O19—Na4—Na5	69.3 (2)	O31—C69—H69	117.8
O28—Na4—Na5	40.64 (17)	N11—C69—H69	117.8
C64—Na4—Na5	59.2 (2)	Na1—C69—H69	93.7
C43—Na4—Na5	55.1 (2)	N11—C70—H70A	109.5
Al5—Na4—Na5	54.61 (11)	N11—C70—H70B	109.5
O29—Na4—Dy1	101.8 (3)	H70A—C70—H70B	109.5
O25—Na4—Dy1	41.49 (17)	N11—C70—H70C	109.5
O37—Na4—Dy1	123.2 (3)	H70A—C70—H70C	109.5
O20—Na4—Dy1	102.1 (2)	H70B—C70—H70C	109.5
O19—Na4—Dy1	37.38 (17)	N11—C71—H71A	109.5
O28—Na4—Dy1	83.02 (17)	N11—C71—H71B	109.5
C64—Na4—Dy1	92.2 (2)	H71A—C71—H71B	109.5
C43—Na4—Dy1	80.3 (2)	N11—C71—H71C	109.5
Al5—Na4—Dy1	53.63 (8)	H71A—C71—H71C	109.5
Na5—Na4—Dy1	104.89 (13)	H71B—C71—H71C	109.5
O20—Na5—O28	88.1 (3)	O32—C72—N12	125.9 (11)
O20—Na5—O33^iv^	171.7 (4)	O32—C72—H72	117.1
O28—Na5—O33^iv^	92.2 (3)	N12—C72—H72	117.1
O20—Na5—O32^iv^	99.6 (3)	N12—C73—H73A	109.5
O28—Na5—O32^iv^	172.1 (3)	N12—C73—H73B	109.5
O33^iv^—Na5—O32^iv^	79.9 (3)	H73A—C73—H73B	109.5
O20—Na5—O12	93.3 (3)	N12—C73—H73C	109.5
O28—Na5—O12	64.2 (3)	H73A—C73—H73C	109.5
O33^iv^—Na5—O12	94.2 (3)	H73B—C73—H73C	109.5
O32^iv^—Na5—O12	116.8 (3)	N12—C74—H74A	109.5
O20—Na5—O34^iv^	88.3 (3)	N12—C74—H74B	109.5
O28—Na5—O34^iv^	92.0 (3)	H74A—C74—H74B	109.5
O33^iv^—Na5—O34^iv^	83.4 (3)	N12—C74—H74C	109.5
O32^iv^—Na5—O34^iv^	86.4 (3)	H74A—C74—H74C	109.5
O12—Na5—O34^iv^	156.1 (3)	H74B—C74—H74C	109.5
O20—Na5—Na2^iv^	123.6 (3)	O33—C75—N13	124.5 (12)
O28—Na5—Na2^iv^	123.3 (2)	O33—C75—Na5^ii^	43.1 (6)
O33^iv^—Na5—Na2^iv^	50.0 (2)	N13—C75—Na5^ii^	165.1 (9)
O32^iv^—Na5—Na2^iv^	50.5 (2)	O33—C75—H75	117.7
O12—Na5—Na2^iv^	140.9 (3)	N13—C75—H75	117.7
O34^iv^—Na5—Na2^iv^	49.9 (2)	Na5^ii^—C75—H75	75.3
O20—Na5—C43	20.7 (3)	N13—C76—H76A	109.5
O28—Na5—C43	88.6 (3)	N13—C76—H76B	109.5
O33^iv^—Na5—C43	167.6 (3)	H76A—C76—H76B	109.5
O32^iv^—Na5—C43	99.2 (3)	N13—C76—H76C	109.5
O12—Na5—C43	75.0 (3)	H76A—C76—H76C	109.5
O34^iv^—Na5—C43	108.9 (3)	H76B—C76—H76C	109.5
Na2^iv^—Na5—C43	137.8 (2)	N13—C77—H77A	109.5
O20—Na5—C75^iv^	162.4 (4)	N13—C77—H77B	109.5
O28—Na5—C75^iv^	74.6 (3)	H77A—C77—H77B	109.5
O33^iv^—Na5—C75^iv^	20.9 (3)	N13—C77—H77C	109.5
O32^iv^—Na5—C75^iv^	97.8 (4)	H77A—C77—H77C	109.5
O12—Na5—C75^iv^	76.6 (3)	H77B—C77—H77C	109.5
O34^iv^—Na5—C75^iv^	95.2 (3)	O34—C78—N14	126.0 (12)
Na2^iv^—Na5—C75^iv^	70.3 (3)	O34—C78—H78	117.0
C43—Na5—C75^iv^	151.1 (3)	N14—C78—H78	117.0
O20—Na5—Al5	80.7 (2)	N14—C79—H79A	109.5
O28—Na5—Al5	34.66 (18)	N14—C79—H79B	109.5
O33^iv^—Na5—Al5	104.1 (2)	H79A—C79—H79B	109.5
O32^iv^—Na5—Al5	148.3 (3)	N14—C79—H79C	109.5
O12—Na5—Al5	32.41 (18)	H79A—C79—H79C	109.5
O34^iv^—Na5—Al5	125.3 (2)	H79B—C79—H79C	109.5
Na2^iv^—Na5—Al5	151.41 (19)	N14—C80—H80A	109.5
C43—Na5—Al5	70.1 (2)	N14—C80—H80B	109.5
C75^iv^—Na5—Al5	83.2 (3)	H80A—C80—H80B	109.5
O20—Na5—Na4	40.7 (2)	N14—C80—H80C	109.5
O28—Na5—Na4	51.2 (2)	H80A—C80—H80C	109.5
O33^iv^—Na5—Na4	135.9 (3)	H80B—C80—H80C	109.5
O32^iv^—Na5—Na4	135.4 (3)	O35—C81—N15	123.2 (11)
O12—Na5—Na4	90.3 (2)	O35—C81—H81	118.4
O34^iv^—Na5—Na4	75.6 (2)	N15—C81—H81	118.4
Na2^iv^—Na5—Na4	125.43 (18)	N15—C82—H82A	109.5
C43—Na5—Na4	51.9 (2)	N15—C82—H82B	109.5
C75^iv^—Na5—Na4	123.9 (3)	H82A—C82—H82B	109.5
Al5—Na5—Na4	61.51 (11)	N15—C82—H82C	109.5
N1—O1—Al1	112.7 (5)	H82A—C82—H82C	109.5
N1—O1—Dy1	115.3 (5)	H82B—C82—H82C	109.5
Al1—O1—Dy1	131.7 (3)	N15—C83—H83A	109.5
C1—O2—Al1	114.4 (6)	N15—C83—H83B	109.5
C7—O3—Al2	129.4 (6)	H83A—C83—H83B	109.5
C7—O3—Na3	118.3 (6)	N15—C83—H83C	109.5
Al2—O3—Na3	85.9 (3)	H83A—C83—H83C	109.5
N2—O4—Al2	114.8 (6)	H83B—C83—H83C	109.5
N2—O4—Dy1	119.7 (5)	O36—C84—N16	124.6 (15)
Al2—O4—Dy1	110.1 (3)	O36—C84—H84	117.7
C8—O5—Al2	111.0 (6)	N16—C84—H84	117.7
C8—O5—Na3	136.8 (6)	N16—C85—H85A	109.5
Al2—O5—Na3	83.2 (3)	N16—C85—H85B	109.5
C14—O6—Al3	129.4 (6)	H85A—C85—H85B	109.5
C14—O6—Na2	115.1 (6)	N16—C85—H85C	109.5
Al3—O6—Na2	87.5 (3)	H85A—C85—H85C	109.5
N3—O7—Al3	115.2 (5)	H85B—C85—H85C	109.5
N3—O7—Dy1	111.8 (5)	N16—C86—H86A	109.5
Al3—O7—Dy1	121.5 (3)	N16—C86—H86B	109.5
C15—O8—Al3	113.7 (6)	H86A—C86—H86B	109.5
C15—O8—Na2	143.1 (7)	N16—C86—H86C	109.5
Al3—O8—Na2	86.9 (3)	H86A—C86—H86C	109.5
C21—O9—Al4	131.1 (8)	H86B—C86—H86C	109.5
C21—O9—Na1	120.6 (7)	O37—C87—N17	125.3 (12)
Al4—O9—Na1	108.2 (4)	O37—C87—H87	117.4
N4—O10—Al4	109.2 (5)	N17—C87—H87	117.4
N4—O10—Dy1	104.6 (5)	N17—C88—H88A	109.5
Al4—O10—Dy1	100.6 (3)	N17—C88—H88B	109.5
C22—O11—Al4	111.1 (6)	H88A—C88—H88B	109.5
C22—O11—Na1	114.8 (6)	N17—C88—H88C	109.5
Al4—O11—Na1	98.3 (3)	H88A—C88—H88C	109.5
C28—O12—Al5	132.4 (6)	H88B—C88—H88C	109.5
C28—O12—Na5	122.1 (6)	N17—C89—H89A	109.5
Al5—O12—Na5	101.8 (3)	N17—C89—H89B	109.5
N5—O13—Al5	111.4 (5)	H89A—C89—H89B	109.5
N5—O13—Dy1	121.3 (5)	N17—C89—H89C	109.5
Al5—O13—Dy1	104.0 (3)	H89A—C89—H89C	109.5
C29—O14—Al5	112.0 (6)	H89B—C89—H89C	109.5
C35—O15—Al6	133.4 (7)	N18—C90—O38	119 (3)
N6—O16—Al6	112.8 (5)	N18—C90—H90	120.6
N6—O16—Dy1	117.4 (5)	O38—C90—H90	120.6
Al6—O16—Dy1	128.1 (3)	C90—N18—C91	122 (2)
C36—O17—Al6	114.0 (6)	C90—N18—C92	125 (2)
C42—O18—Al3	135.2 (7)	C91—N18—C92	113 (2)
C42—O18—Na2	116.0 (6)	N18—C91—H91A	109.5
Al3—O18—Na2	88.1 (3)	N18—C91—H91B	109.5
N7—O19—Al5	119.7 (6)	H91A—C91—H91B	109.5
N7—O19—Na4	104.7 (5)	N18—C91—H91C	109.5
Al5—O19—Na4	108.6 (3)	H91A—C91—H91C	109.5
N7—O19—Dy1	117.5 (5)	H91B—C91—H91C	109.5
Al5—O19—Dy1	99.8 (3)	N18—C92—H92A	109.5
Na4—O19—Dy1	105.7 (3)	N18—C92—H92B	109.5
C43—O20—Na5	118.7 (7)	H92A—C92—H92B	109.5
C43—O20—Na4	104.9 (6)	N18—C92—H92C	109.5
Na5—O20—Na4	100.7 (3)	H92A—C92—H92C	109.5
C49—O21—Al1	147.4 (7)	H92B—C92—H92C	109.5
N8—O22—Al4	112.0 (5)	O39—C93—N19	126 (2)
N8—O22—Dy1	122.3 (5)	O39—C93—H93	116.8
Al4—O22—Dy1	103.2 (3)	N19—C93—H93	116.8
C50—O23—Al4	112.0 (6)	N19—C94—H94A	109.5
C56—O24—Al1	130.5 (7)	N19—C94—H94B	109.5
N9—O25—Al2	106.4 (5)	H94A—C94—H94B	109.5
N9—O25—Na4	110.5 (5)	N19—C94—H94C	109.5
Al2—O25—Na4	133.1 (3)	H94A—C94—H94C	109.5
N9—O25—Dy1	103.0 (5)	H94B—C94—H94C	109.5
Al2—O25—Dy1	95.9 (3)	N19—C95—H95A	109.5
Na4—O25—Dy1	102.8 (3)	N19—C95—H95B	109.5
C57—O26—Al2	110.6 (6)	H95A—C95—H95B	109.5
C57—O26—Na3	159.9 (6)	N19—C95—H95C	109.5
Al2—O26—Na3	89.5 (3)	H95A—C95—H95C	109.5
C63—O27—Al6	133.5 (6)	H95B—C95—H95C	109.5
C64—O28—Al5	132.7 (7)	O40—C96—N20	130.1 (19)
C64—O28—Na5	126.5 (7)	O40—C96—H96	114.9
Al5—O28—Na5	100.4 (3)	N20—C96—H96	114.9
C64—O28—Na4	81.7 (7)	N20—C97—H97A	109.5
Al5—O28—Na4	95.2 (3)	N20—C97—H97B	109.5
Na5—O28—Na4	88.2 (3)	H97A—C97—H97B	109.5
C64—O29—Na4	106.6 (8)	N20—C97—H97C	109.5
C66—O30—Na1	147.7 (8)	H97A—C97—H97C	109.5
C66—O30—Na3^i^	130.9 (8)	H97B—C97—H97C	109.5
Na1—O30—Na3^i^	81.2 (3)	N20—C98—H98A	109.5
C69—O31—Na1	115.5 (8)	N20—C98—H98B	109.5
C72—O32—Na2	150.0 (8)	H98A—C98—H98B	109.5
C72—O32—Na5^ii^	131.3 (8)	N20—C98—H98C	109.5
Na2—O32—Na5^ii^	77.5 (3)	H98A—C98—H98C	109.5
C75—O33—Na2	160.1 (9)	H98B—C98—H98C	109.5
C75—O33—Na5^ii^	116.0 (8)		
			
O21—Al1—O1—N1	−106.3 (6)	Al3—O6—C14—C9	−23.2 (14)
O24—Al1—O1—N1	80.8 (7)	Na2—O6—C14—C9	−132.0 (9)
O2—Al1—O1—N1	−6.2 (5)	Al3—O6—C14—C13	156.9 (8)
N8—Al1—O1—N1	165.2 (6)	Na2—O6—C14—C13	48.1 (11)
O21—Al1—O1—Dy1	67.2 (5)	C10—C9—C14—O6	177.5 (10)
O24—Al1—O1—Dy1	−105.6 (6)	C8—C9—C14—O6	−8.5 (16)
O2—Al1—O1—Dy1	167.4 (5)	C10—C9—C14—C13	−2.5 (15)
N8—Al1—O1—Dy1	−21.3 (5)	C8—C9—C14—C13	171.5 (9)
O21—Al1—O2—C1	118.6 (7)	C12—C13—C14—O6	−177.1 (10)
O24—Al1—O2—C1	−128.8 (7)	C12—C13—C14—C9	3.0 (16)
O1—Al1—O2—C1	6.0 (7)	Al3—O8—C15—N3	−0.2 (12)
N8—Al1—O2—C1	−26.9 (17)	Na2—O8—C15—N3	−119.2 (10)
O5—Al2—O3—C7	74.1 (9)	Al3—O8—C15—C16	178.0 (8)
O26—Al2—O3—C7	168.7 (9)	Na2—O8—C15—C16	58.9 (15)
N1—Al2—O3—C7	−24.7 (9)	O7—N3—C15—O8	3.8 (12)
O25—Al2—O3—C7	−110.4 (9)	Al4—N3—C15—O8	−175.0 (7)
Na3—Al2—O3—C7	123.7 (9)	O7—N3—C15—C16	−174.4 (9)
Dy1—Al2—O3—C7	−70.2 (10)	Al4—N3—C15—C16	6.8 (15)
O5—Al2—O3—Na3	−49.6 (3)	O8—C15—C16—C17	−12.6 (16)
O26—Al2—O3—Na3	45.0 (3)	N3—C15—C16—C17	165.6 (11)
N1—Al2—O3—Na3	−148.4 (3)	O8—C15—C16—C21	167.0 (11)
O25—Al2—O3—Na3	125.9 (3)	N3—C15—C16—C21	−14.8 (16)
Dy1—Al2—O3—Na3	166.1 (3)	C21—C16—C17—C18	1 (2)
O5—Al2—O4—N2	−7.5 (5)	C15—C16—C17—C18	−179.6 (13)
O26—Al2—O4—N2	−102.0 (6)	C16—C17—C18—C19	−3 (3)
N1—Al2—O4—N2	90.8 (6)	C17—C18—C19—C20	3 (3)
O25—Al2—O4—N2	175.8 (6)	C18—C19—C20—C21	−1 (3)
Na3—Al2—O4—N2	−55.5 (6)	Al4—O9—C21—C20	−154.8 (10)
Dy1—Al2—O4—N2	138.6 (7)	Na1—O9—C21—C20	21.9 (15)
O5—Al2—O4—Dy1	−146.1 (3)	Al4—O9—C21—C16	27.7 (17)
O26—Al2—O4—Dy1	119.4 (3)	Na1—O9—C21—C16	−155.6 (9)
N1—Al2—O4—Dy1	−47.8 (4)	C19—C20—C21—O9	−178.7 (15)
O25—Al2—O4—Dy1	37.2 (3)	C19—C20—C21—C16	−1 (2)
Na3—Al2—O4—Dy1	165.9 (2)	C17—C16—C21—O9	178.7 (11)
O18—Al3—O6—C14	−74.6 (8)	C15—C16—C21—O9	−0.9 (18)
O7—Al3—O6—C14	115.2 (8)	C17—C16—C21—C20	1.1 (18)
O8—Al3—O6—C14	−164.2 (8)	C15—C16—C21—C20	−178.5 (12)
N2—Al3—O6—C14	32.4 (8)	O10—N4—C22—O11	0.1 (13)
N6—Al3—O6—C14	−26 (2)	Al5—N4—C22—O11	163.2 (7)
Na2—Al3—O6—C14	−120.9 (8)	O10—N4—C22—C23	179.3 (8)
O18—Al3—O6—Na2	46.3 (3)	Al5—N4—C22—C23	−17.6 (14)
O7—Al3—O6—Na2	−123.9 (3)	Al4—O11—C22—N4	−18.9 (11)
O8—Al3—O6—Na2	−43.4 (3)	Na1—O11—C22—N4	91.5 (9)
N2—Al3—O6—Na2	153.2 (3)	Al4—O11—C22—C23	161.9 (7)
N6—Al3—O6—Na2	95.0 (18)	Na1—O11—C22—C23	−87.7 (9)
O18—Al3—O7—N3	−38.9 (18)	N4—C22—C23—C24	−169.6 (11)
O6—Al3—O7—N3	91.1 (6)	O11—C22—C23—C24	9.6 (15)
O8—Al3—O7—N3	4.5 (6)	N4—C22—C23—C28	10.8 (15)
N2—Al3—O7—N3	174.0 (6)	O11—C22—C23—C28	−170.0 (9)
N6—Al3—O7—N3	−95.9 (6)	C28—C23—C24—C25	−4.0 (19)
Na2—Al3—O7—N3	37.6 (7)	C22—C23—C24—C25	176.4 (12)
O18—Al3—O7—Dy1	101.5 (15)	C23—C24—C25—C26	2 (2)
O6—Al3—O7—Dy1	−128.5 (4)	C24—C25—C26—C27	0 (2)
O8—Al3—O7—Dy1	144.9 (4)	C25—C26—C27—C28	0 (2)
N2—Al3—O7—Dy1	−45.6 (4)	Al5—O12—C28—C27	170.0 (8)
N6—Al3—O7—Dy1	44.4 (4)	Na5—O12—C28—C27	−35.9 (12)
Na2—Al3—O7—Dy1	178.0 (2)	Al5—O12—C28—C23	−10.0 (16)
O11—Al4—O9—C21	166.0 (10)	Na5—O12—C28—C23	144.1 (8)
O23—Al4—O9—C21	67.5 (10)	C26—C27—C28—O12	178.6 (11)
O10—Al4—O9—C21	−113.7 (10)	C26—C27—C28—C23	−1.4 (17)
N3—Al4—O9—C21	−27.8 (10)	C24—C23—C28—O12	−176.6 (11)
Na1—Al4—O9—C21	177.0 (11)	C22—C23—C28—O12	3.0 (16)
Dy1—Al4—O9—C21	−73.5 (11)	C24—C23—C28—C27	3.5 (16)
O11—Al4—O9—Na1	−11.0 (4)	C22—C23—C28—C27	−177.0 (10)
O23—Al4—O9—Na1	−109.5 (4)	Al5—O14—C29—N5	6.1 (11)
O10—Al4—O9—Na1	69.3 (4)	Al5—O14—C29—C30	−174.9 (7)
N3—Al4—O9—Na1	155.2 (4)	O13—N5—C29—O14	1.2 (12)
Dy1—Al4—O9—Na1	109.5 (4)	Al6—N5—C29—O14	179.5 (7)
O9—Al4—O11—C22	130.3 (7)	O13—N5—C29—C30	−177.8 (8)
O23—Al4—O11—C22	−137.0 (6)	Al6—N5—C29—C30	0.5 (13)
O22—Al4—O11—C22	−54.7 (6)	O14—C29—C30—C35	176.7 (9)
O10—Al4—O11—C22	22.2 (6)	N5—C29—C30—C35	−4.4 (14)
N3—Al4—O11—C22	57.9 (16)	O14—C29—C30—C31	−2.6 (14)
Na1—Al4—O11—C22	120.7 (7)	N5—C29—C30—C31	176.3 (10)
Dy1—Al4—O11—C22	−12.4 (7)	C35—C30—C31—C32	0.0 (18)
O9—Al4—O11—Na1	9.6 (3)	C29—C30—C31—C32	179.3 (11)
O23—Al4—O11—Na1	102.2 (3)	C30—C31—C32—C33	−1 (2)
O22—Al4—O11—Na1	−175.4 (3)	C31—C32—C33—C34	0 (2)
O10—Al4—O11—Na1	−98.5 (3)	C32—C33—C34—C35	1.2 (19)
N3—Al4—O11—Na1	−62.8 (15)	Al6—O15—C35—C34	−172.3 (8)
Dy1—Al4—O11—Na1	−133.15 (19)	Al6—O15—C35—C30	9.9 (16)
O14—Al5—O12—C28	−87.4 (9)	C33—C34—C35—O15	−179.8 (11)
O28—Al5—O12—C28	179.7 (9)	C33—C34—C35—C30	−1.8 (16)
O19—Al5—O12—C28	89.0 (9)	C31—C30—C35—O15	179.1 (10)
N4—Al5—O12—C28	3.9 (9)	C29—C30—C35—O15	−0.2 (15)
Na5—Al5—O12—C28	157.9 (11)	C31—C30—C35—C34	1.2 (15)
Dy1—Al5—O12—C28	56.3 (10)	C29—C30—C35—C34	−178.1 (9)
Na4—Al5—O12—C28	130.0 (9)	Al6—O17—C36—N6	1.6 (12)
O14—Al5—O12—Na5	114.7 (3)	Al6—O17—C36—C37	−173.8 (7)
O28—Al5—O12—Na5	21.9 (3)	O16—N6—C36—O17	−0.4 (13)
O19—Al5—O12—Na5	−68.9 (3)	Al3—N6—C36—O17	176.8 (7)
N4—Al5—O12—Na5	−154.0 (3)	O16—N6—C36—C37	174.8 (9)
Dy1—Al5—O12—Na5	−101.5 (3)	Al3—N6—C36—C37	−8.1 (15)
Na4—Al5—O12—Na5	−27.8 (3)	O17—C36—C37—C42	−174.2 (10)
O14—Al5—O13—N5	8.6 (5)	N6—C36—C37—C42	10.6 (16)
O28—Al5—O13—N5	101.8 (5)	O17—C36—C37—C38	6.1 (15)
O19—Al5—O13—N5	−167.2 (5)	N6—C36—C37—C38	−169.2 (10)
N4—Al5—O13—N5	−82.5 (5)	C42—C37—C38—C39	0.3 (19)
Na5—Al5—O13—N5	125.0 (5)	C36—C37—C38—C39	−180.0 (12)
Dy1—Al5—O13—N5	−132.3 (6)	C37—C38—C39—C40	−2 (2)
Na4—Al5—O13—N5	151.3 (5)	C38—C39—C40—C41	1 (2)
O14—Al5—O13—Dy1	140.9 (3)	C39—C40—C41—C42	0 (2)
O28—Al5—O13—Dy1	−125.9 (3)	Al3—O18—C42—C41	161.6 (8)
O19—Al5—O13—Dy1	−35.0 (3)	Na2—O18—C42—C41	45.5 (12)
N4—Al5—O13—Dy1	49.8 (3)	Al3—O18—C42—C37	−18.1 (16)
Na5—Al5—O13—Dy1	−102.8 (3)	Na2—O18—C42—C37	−134.2 (9)
Na4—Al5—O13—Dy1	−76.4 (2)	C40—C41—C42—O18	178.7 (11)
O12—Al5—O14—C29	173.3 (6)	C40—C41—C42—C37	−1.6 (18)
O13—Al5—O14—C29	−8.1 (6)	C38—C37—C42—O18	−179.0 (10)
O28—Al5—O14—C29	−99.9 (7)	C36—C37—C42—O18	1.2 (17)
O19—Al5—O14—C29	4.3 (14)	C38—C37—C42—C41	1.3 (16)
N4—Al5—O14—C29	83.1 (7)	C36—C37—C42—C41	−178.5 (10)
Na5—Al5—O14—C29	−141.3 (6)	Na5—O20—C43—N7	−67.5 (12)
Dy1—Al5—O14—C29	20.4 (7)	Na4—O20—C43—N7	43.9 (11)
Na4—Al5—O14—C29	−64.8 (8)	Na5—O20—C43—C44	112.4 (9)
O27—Al6—O15—C35	83.6 (10)	Na4—O20—C43—C44	−136.2 (8)
O17—Al6—O15—C35	−174.8 (10)	Na5—O20—C43—Na4	−111.4 (6)
O16—Al6—O15—C35	−93.3 (10)	Na4—O20—C43—Na5	111.4 (6)
N5—Al6—O15—C35	−10.6 (10)	O19—N7—C43—O20	−13.2 (15)
O15—Al6—O16—N6	−83.3 (7)	O19—N7—C43—C44	166.9 (8)
O27—Al6—O16—N6	99.7 (6)	O19—N7—C43—Na4	19.9 (8)
O17—Al6—O16—N6	1.4 (6)	O19—N7—C43—Na5	−51.0 (9)
N5—Al6—O16—N6	−168.4 (6)	O20—C43—C44—C45	−25.9 (15)
O15—Al6—O16—Dy1	112.4 (5)	N7—C43—C44—C45	154.0 (10)
O27—Al6—O16—Dy1	−64.5 (5)	Na4—C43—C44—C45	−91.3 (13)
O17—Al6—O16—Dy1	−162.9 (5)	Na5—C43—C44—C45	23.1 (13)
N5—Al6—O16—Dy1	27.3 (5)	O20—C43—C44—C49	148.6 (10)
O15—Al6—O17—C36	131.5 (7)	N7—C43—C44—C49	−31.5 (15)
O27—Al6—O17—C36	−112.6 (7)	Na4—C43—C44—C49	83.2 (14)
O16—Al6—O17—C36	−1.6 (7)	Na5—C43—C44—C49	−162.3 (8)
N5—Al6—O17—C36	38.8 (18)	C49—C44—C45—C46	1.3 (17)
O7—Al3—O18—C42	−41 (2)	C43—C44—C45—C46	176.0 (10)
O6—Al3—O18—C42	−172.0 (10)	C44—C45—C46—C47	−2.5 (18)
O8—Al3—O18—C42	−83.4 (9)	C45—C46—C47—C48	1.7 (18)
N2—Al3—O18—C42	105.1 (10)	C46—C47—C48—C49	0.3 (18)
N6—Al3—O18—C42	16.4 (10)	Al1—O21—C49—C44	−97.7 (15)
Na2—Al3—O18—C42	−126.2 (10)	Al1—O21—C49—C48	84.5 (15)
O7—Al3—O18—Na2	85.5 (16)	C45—C44—C49—O21	−177.2 (10)
O6—Al3—O18—Na2	−45.9 (3)	C43—C44—C49—O21	8.4 (16)
O8—Al3—O18—Na2	42.8 (3)	C45—C44—C49—C48	0.6 (16)
N2—Al3—O18—Na2	−128.8 (3)	C43—C44—C49—C48	−173.8 (9)
N6—Al3—O18—Na2	142.6 (3)	C47—C48—C49—O21	176.5 (10)
O24—Al1—O21—C49	−97.9 (13)	C47—C48—C49—C44	−1.4 (16)
O2—Al1—O21—C49	0.4 (13)	O22—N8—C50—O23	−2.5 (13)
O1—Al1—O21—C49	87.5 (13)	Al1—N8—C50—O23	−168.7 (7)
N8—Al1—O21—C49	171.5 (13)	O22—N8—C50—C51	176.9 (8)
O9—Al4—O23—C50	−163.7 (7)	Al1—N8—C50—C51	10.7 (15)
O11—Al4—O23—C50	107.8 (7)	Al4—O23—C50—N8	−9.6 (12)
O22—Al4—O23—C50	13.1 (7)	Al4—O23—C50—C51	171.1 (7)
O10—Al4—O23—C50	19.8 (14)	N8—C50—C51—C52	−178.4 (10)
N3—Al4—O23—C50	−75.9 (7)	O23—C50—C51—C52	1.0 (15)
Na1—Al4—O23—C50	156.8 (6)	N8—C50—C51—C56	0.9 (15)
Dy1—Al4—O23—C50	−11.6 (8)	O23—C50—C51—C56	−179.7 (9)
O21—Al1—O24—C56	−70.4 (10)	C56—C51—C52—C53	0.2 (16)
O2—Al1—O24—C56	−174.9 (9)	C50—C51—C52—C53	179.6 (11)
O1—Al1—O24—C56	102.5 (9)	C51—C52—C53—C54	−0.9 (18)
N8—Al1—O24—C56	20.8 (9)	C52—C53—C54—C55	1.7 (19)
O15—Al6—O27—C63	104.3 (9)	C53—C54—C55—C56	−1.8 (19)
O17—Al6—O27—C63	8.7 (9)	Al1—O24—C56—C55	164.1 (8)
O16—Al6—O27—C63	−78.1 (9)	Al1—O24—C56—C51	−17.1 (15)
N5—Al6—O27—C63	−163.7 (8)	C54—C55—C56—O24	179.9 (10)
O41—C99—N21—C100	2 (9)	C54—C55—C56—C51	1.0 (16)
O41—C99—N21—C101	−163 (5)	C52—C51—C56—O24	−179.0 (10)
O41B—C99B—N21B—C102	2 (9)	C50—C51—C56—O24	1.6 (16)
O41B—C99B—N21B—C103	−160 (6)	C52—C51—C56—C55	−0.2 (15)
Al1—O1—N1—C1	5.3 (9)	C50—C51—C56—C55	−179.6 (9)
Dy1—O1—N1—C1	−169.4 (6)	Al2—O26—C57—N9	−18.9 (11)
Al1—O1—N1—Al2	−165.0 (4)	Na3—O26—C57—N9	158.0 (15)
Dy1—O1—N1—Al2	20.3 (7)	Al2—O26—C57—C58	162.5 (7)
Al2—O4—N2—C8	3.5 (9)	Na3—O26—C57—C58	−21 (3)
Dy1—O4—N2—C8	137.8 (7)	O25—N9—C57—O26	3.3 (13)
Al2—O4—N2—Al3	−173.8 (4)	O25—N9—C57—C58	−178.2 (8)
Dy1—O4—N2—Al3	−39.4 (7)	O26—C57—C58—C59	24.0 (14)
Al3—O7—N3—C15	−5.7 (9)	N9—C57—C58—C59	−154.5 (10)
Dy1—O7—N3—C15	−149.8 (7)	O26—C57—C58—C63	−154.4 (10)
Al3—O7—N3—Al4	173.2 (4)	N9—C57—C58—C63	27.0 (14)
Dy1—O7—N3—Al4	29.1 (7)	C63—C58—C59—C60	−2.7 (16)
Al4—O10—N4—C22	17.9 (9)	C57—C58—C59—C60	178.8 (9)
Dy1—O10—N4—C22	124.9 (7)	C58—C59—C60—C61	2.6 (16)
Al4—O10—N4—Al5	−147.0 (4)	C59—C60—C61—C62	−0.8 (16)
Dy1—O10—N4—Al5	−40.0 (6)	C60—C61—C62—C63	−0.9 (16)
Al5—O13—N5—C29	−7.7 (8)	Al6—O27—C63—C58	100.0 (11)
Dy1—O13—N5—C29	−130.5 (6)	Al6—O27—C63—C62	−84.2 (12)
Al5—O13—N5—Al6	173.7 (4)	C59—C58—C63—O27	176.9 (9)
Dy1—O13—N5—Al6	50.9 (7)	C57—C58—C63—O27	−4.7 (15)
Al6—O16—N6—C36	−0.9 (9)	C59—C58—C63—C62	1.0 (15)
Dy1—O16—N6—C36	165.2 (6)	C57—C58—C63—C62	179.3 (9)
Al6—O16—N6—Al3	−178.4 (4)	C61—C62—C63—O27	−175.1 (9)
Dy1—O16—N6—Al3	−12.3 (8)	C61—C62—C63—C58	0.9 (15)
Al5—O19—N7—C43	96.6 (9)	Na4—O29—C64—O28	−3.9 (13)
Na4—O19—N7—C43	−25.3 (10)	Na4—O29—C64—C65	178.8 (10)
Dy1—O19—N7—C43	−142.2 (7)	Al5—O28—C64—O29	92.6 (13)
Al4—O22—N8—C50	13.3 (9)	Na5—O28—C64—O29	−78.4 (13)
Dy1—O22—N8—C50	136.4 (7)	Na4—O28—C64—O29	3.1 (10)
Al4—O22—N8—Al1	−178.1 (4)	Al5—O28—C64—C65	−90.1 (13)
Dy1—O22—N8—Al1	−55.0 (7)	Na5—O28—C64—C65	98.9 (12)
Al2—O25—N9—C57	13.4 (9)	Na4—O28—C64—C65	−179.6 (11)
Na4—O25—N9—C57	−137.1 (7)	Al5—O28—C64—Na4	89.5 (8)
Dy1—O25—N9—C57	113.6 (7)	Na5—O28—C64—Na4	−81.5 (7)
Al1—O2—C1—N1	−4.8 (11)	Na1—O30—C66—N10	−31 (3)
Al1—O2—C1—C2	175.7 (7)	Na3^i^—O30—C66—N10	159.0 (11)
O1—N1—C1—O2	−0.4 (12)	C67—N10—C66—O30	1 (2)
Al2—N1—C1—O2	168.5 (6)	C68—N10—C66—O30	175.8 (15)
O1—N1—C1—C2	179.1 (8)	Na1—O31—C69—N11	−116.7 (12)
Al2—N1—C1—C2	−11.9 (14)	C71—N11—C69—O31	176.3 (12)
O2—C1—C2—C7	176.6 (9)	C70—N11—C69—O31	2 (2)
N1—C1—C2—C7	−2.9 (15)	C71—N11—C69—Na1	120.9 (12)
O2—C1—C2—C3	−3.8 (15)	C70—N11—C69—Na1	−53.8 (17)
N1—C1—C2—C3	176.6 (10)	Na2—O32—C72—N12	27 (3)
C7—C2—C3—C4	−0.7 (17)	Na5^ii^—O32—C72—N12	−172.7 (10)
C1—C2—C3—C4	179.7 (11)	C73—N12—C72—O32	−5 (2)
C2—C3—C4—C5	0.0 (19)	C74—N12—C72—O32	177.5 (16)
C3—C4—C5—C6	0 (2)	Na2—O33—C75—N13	35 (4)
C4—C5—C6—C7	1.3 (19)	Na5^ii^—O33—C75—N13	169.0 (11)
Al2—O3—C7—C6	−162.2 (8)	Na2—O33—C75—Na5^ii^	−134 (3)
Na3—O3—C7—C6	−52.6 (12)	C76—N13—C75—O33	2 (2)
Al2—O3—C7—C2	19.0 (15)	C77—N13—C75—O33	−170.5 (16)
Na3—O3—C7—C2	128.6 (9)	C76—N13—C75—Na5^ii^	33 (5)
C5—C6—C7—O3	179.1 (11)	C77—N13—C75—Na5^ii^	−140 (3)
C5—C6—C7—C2	−2.1 (17)	Na2—O34—C78—N14	69 (2)
C3—C2—C7—O3	−179.5 (10)	Na5^ii^—O34—C78—N14	−169.4 (9)
C1—C2—C7—O3	0.1 (16)	C80—N14—C78—O34	177.4 (12)
C3—C2—C7—C6	1.7 (16)	C79—N14—C78—O34	−5.9 (19)
C1—C2—C7—C6	−178.7 (10)	Na3—O35—C81—N15	54.3 (18)
O4—N2—C8—O5	5.5 (12)	Na1^iii^—O35—C81—N15	173.2 (8)
Al3—N2—C8—O5	−177.9 (7)	C83—N15—C81—O35	−172.0 (12)
O4—N2—C8—C9	−170.6 (8)	C82—N15—C81—O35	−1.1 (18)
Al3—N2—C8—C9	6.0 (15)	Na3—O36—C84—N16	−49 (4)
Al2—O5—C8—N2	−11.5 (11)	Na1^iii^—O36—C84—N16	178.7 (14)
Na3—O5—C8—N2	91.4 (12)	C85—N16—C84—O36	−7 (3)
Al2—O5—C8—C9	164.6 (7)	C86—N16—C84—O36	−178.6 (19)
Na3—O5—C8—C9	−92.5 (11)	Na4—O37—C87—N17	176.0 (10)
N2—C8—C9—C10	−169.6 (10)	C88—N17—C87—O37	2 (2)
O5—C8—C9—C10	14.2 (15)	C89—N17—C87—O37	178.6 (12)
N2—C8—C9—C14	16.4 (14)	O38—C90—N18—C91	13 (4)
O5—C8—C9—C14	−159.8 (9)	O38—C90—N18—C92	−173 (2)
C14—C9—C10—C11	−1.0 (18)	C94—N19—C93—O39	2 (4)
C8—C9—C10—C11	−175.0 (11)	C95—N19—C93—O39	−176 (2)
C9—C10—C11—C12	4 (2)	C98—N20—C96—O40	−174 (2)
C10—C11—C12—C13	−4 (2)	C97—N20—C96—O40	21 (4)
C11—C12—C13—C14	0.2 (19)		

Symmetry codes: (i) *x*+1/2, *y*+1/2, *z*; (ii) *x*−1/2, *y*+1/2, *z*; (iii) *x*−1/2, *y*−1/2, *z*; (iv) *x*+1/2, *y*−1/2, *z*.

### Poly[[µ3-acetato-hexakis(µ-N,N-dimethylformamide)bis(N,N-dimethylformamide)bis[salicylhydroximato(2-)]heptakis[salicylhydroximato(3-)]hexaaluminium(III)dysprosium(III)pentasodium(I)] N,N-dimethylformamide tetrasolvate monohydrate] (1) Hydrogen-bond geometry (Å, º)

**Table:**

*D*—H···*A*	*D*—H	H···*A*	*D*···*A*	*D*—H···*A*
O42—H42*A*···O3	0.84	2.08	2.868 (14)	156
O42—H42*B*···O37	0.84	2.08	2.820 (15)	146
N7—H7···O22	0.90 (3)	2.25 (6)	2.899 (11)	129 (5)
N9—H9···O27	0.91 (3)	1.92 (3)	2.693 (11)	142 (6)
C20—H20···O35^i^	0.95	2.65	3.483 (16)	147
C41—H41···O34	0.95	2.60	3.469 (14)	153
C66—H66···O42^i^	0.95	2.36	3.30 (2)	171
C67—H67*A*···O31	0.98	2.47	3.41 (2)	159
C69—H69···O10	0.95	2.50	3.260 (14)	137
C71—H71*C*···O16	0.98	2.56	3.363 (16)	139
C73—H73*A*···O6	0.98	2.33	3.268 (14)	159
C74—H74*C*···O38^v^	0.98	2.34	3.32 (3)	177
C78—H78···O20^ii^	0.95	2.56	3.260 (14)	131
C78—H78···O37^ii^	0.95	2.65	3.524 (16)	154
C79—H79*B*···O41	0.98	2.45	3.25 (4)	139
C79—H79*C*···O18	0.98	2.48	3.446 (17)	168
C80—H80*A*···O37^ii^	0.98	2.52	3.470 (18)	163
C80—H80*B*···O41	0.98	2.49	3.31 (4)	140
C83—H83*B*···O40	0.98	2.35	3.309 (19)	166
C84—H84···O11^iii^	0.95	2.58	3.292 (18)	132
C84—H84···O23^iii^	0.95	2.54	3.414 (18)	153
C92—H92*B*···O21^iii^	0.98	2.40	3.35 (2)	164
C94—H94*A*···O41	0.98	2.47	3.32 (4)	145
C95—H95*A*···O41	0.98	2.36	3.23 (4)	147
C97—H97*B*···O31^iii^	0.98	2.57	3.35 (3)	137
C98—H98*B*···O24^vi^	0.98	2.65	3.318 (19)	125

Symmetry codes: (i) *x*+1/2, *y*+1/2, *z*; (ii) *x*−1/2, *y*+1/2, *z*; (iii) *x*−1/2, *y*−1/2, *z*; (v) *x*, *y*+1, *z*; (vi) *x*, −*y*+1, *z*+1/2.

### Poly[[di-µ4-acetato-nonakis(µ-N,N-dimethylformamide)octakis(N,N-dimethylformamide)tetrakis[salicylhydroximato(2-)]tetradecakis[salicylhydroximato(3-)]dodecaaluminium(III)didysprosium(III)decasodium(I)] N,N-dimethylformamide 6.335-solvate] (2) Crystal data

**Table:**

[Dy_2_Al_12_Na_10_(C_7_H_5_NO_3_)_4_(C_7_H_4_NO_3_)_14_(C_2_H_3_O_2_)_2_(C_3_H_7_NO)_17_]·6.335C_3_H_7_NO	*F*(000) = 5567
*M**_r_* = 5408.50	*D*_x_ = 1.407 Mg m^−^^3^
Monoclinic, *P**c*	Cu *K*α radiation, λ = 1.54178 Å
*a* = 28.1847 (14) Å	Cell parameters from 9892 reflections
*b* = 15.3683 (8) Å	θ = 2.9–66.7°
*c* = 30.7354 (16) Å	µ = 4.44 mm^−^^1^
β = 106.461 (2)°	*T* = 100 K
*V* = 12767.4 (11) Å^3^	Block, colorless
*Z* = 2	0.25 × 0.23 × 0.19 mm

### Poly[[di-µ4-acetato-nonakis(µ-N,N-dimethylformamide)octakis(N,N-dimethylformamide)tetrakis[salicylhydroximato(2-)]tetradecakis[salicylhydroximato(3-)]dodecaaluminium(III)didysprosium(III)decasodium(I)] N,N-dimethylformamide 6.335-solvate] (2) Data collection

**Table:**

Bruker X8 Prospector CCD diffractometer	38274 independent reflections
Radiation source: I-mu-S microsource X-ray tube	36111 reflections with *I* > 2σ(*I*)
Laterally graded multilayer (Goebel) mirror monochromator	*R*_int_ = 0.050
ω and phi scans	θ_max_ = 66.9°, θ_min_ = 2.9°
Absorption correction: multi-scan (SADABS; Bruker, 2014)	*h* = −33→31
*T*_min_ = 0.488, *T*_max_ = 0.753	*k* = −18→18
154195 measured reflections	*l* = −35→36

### Poly[[di-µ4-acetato-nonakis(µ-N,N-dimethylformamide)octakis(N,N-dimethylformamide)tetrakis[salicylhydroximato(2-)]tetradecakis[salicylhydroximato(3-)]dodecaaluminium(III)didysprosium(III)decasodium(I)] N,N-dimethylformamide 6.335-solvate] (2) Refinement

**Table:**

Refinement on *F*^2^	Secondary atom site location: difference Fourier map
Least-squares matrix: full	Hydrogen site location: mixed
*R*[*F*^2^ > 2σ(*F*^2^)] = 0.053	H atoms treated by a mixture of independent and constrained refinement
*wR*(*F*^2^) = 0.137	*w* = 1/[σ^2^(*F*_o_^2^) + (0.082*P*)^2^ + 21.873*P*] where *P* = (*F*_o_^2^ + 2*F*_c_^2^)/3
*S* = 1.02	(Δ/σ)_max_ = 0.002
38274 reflections	Δρ_max_ = 2.28 e Å^−^^3^
3599 parameters	Δρ_min_ = −1.57 e Å^−^^3^
4954 restraints	Absolute structure: Refined as an inversion twin
Primary atom site location: structure-invariant direct methods	Absolute structure parameter: 0.097 (3)

### Poly[[di-µ4-acetato-nonakis(µ-N,N-dimethylformamide)octakis(N,N-dimethylformamide)tetrakis[salicylhydroximato(2-)]tetradecakis[salicylhydroximato(3-)]dodecaaluminium(III)didysprosium(III)decasodium(I)] N,N-dimethylformamide 6.335-solvate] (2) Special details

**Table:**

Geometry. All esds (except the esd in the dihedral angle between two l.s. planes) are estimated using the full covariance matrix. The cell esds are taken into account individually in the estimation of esds in distances, angles and torsion angles; correlations between esds in cell parameters are only used when they are defined by crystal symmetry. An approximate (isotropic) treatment of cell esds is used for estimating esds involving l.s. planes.
Refinement. Refined as a 2-component inversion twin.

### Poly[[di-µ4-acetato-nonakis(µ-N,N-dimethylformamide)octakis(N,N-dimethylformamide)tetrakis[salicylhydroximato(2-)]tetradecakis[salicylhydroximato(3-)]dodecaaluminium(III)didysprosium(III)decasodium(I)] N,N-dimethylformamide 6.335-solvate] (2) Fractional atomic coordinates and isotropic or equivalent isotropic displacement parameters (Å^2^)

**Table:**

	*x*	*y*	*z*	*U*_iso_*/*U*_eq_	Occ. (<1)
Dy1	0.81505 (5)	0.03132 (2)	0.32176 (5)	0.00908 (10)	
Dy2	0.37482 (5)	−0.07517 (2)	0.29104 (5)	0.00947 (10)	
Al1	0.87169 (9)	0.22632 (13)	0.34445 (9)	0.0129 (4)	
Al2	0.70802 (9)	0.14467 (13)	0.29915 (8)	0.0102 (4)	
Al3	0.76745 (10)	0.01246 (17)	0.19191 (9)	0.0184 (5)	
Al4	0.87180 (10)	0.02323 (14)	0.45422 (9)	0.0157 (5)	
Al5	0.81896 (10)	−0.18923 (14)	0.34637 (9)	0.0146 (4)	
Al6	0.92808 (9)	−0.02834 (14)	0.29235 (9)	0.0142 (4)	
Al7	0.36936 (10)	0.14332 (13)	0.26170 (9)	0.0152 (4)	
Al8	0.32082 (10)	−0.07658 (13)	0.15664 (9)	0.0140 (5)	
Al9	0.26280 (9)	−0.01313 (14)	0.32172 (9)	0.0126 (4)	
Al10	0.42417 (10)	−0.04756 (16)	0.42172 (9)	0.0171 (4)	
Al11	0.48273 (9)	−0.19014 (13)	0.31645 (9)	0.0119 (4)	
Al12	0.31882 (9)	−0.27069 (13)	0.27197 (9)	0.0121 (4)	
Na1	1.03076 (13)	−0.0102 (2)	0.27827 (13)	0.0284 (7)	
Na2	0.84298 (13)	0.42856 (18)	0.30616 (13)	0.0215 (7)	
Na3	0.84048 (14)	−0.37615 (19)	0.33220 (13)	0.0266 (7)	
Na4	0.68710 (12)	−0.06147 (19)	0.33551 (11)	0.0189 (6)	
Na5	0.61385 (12)	0.12999 (19)	0.34704 (12)	0.0192 (6)	
Na6	0.58031 (12)	−0.1777 (2)	0.26870 (12)	0.0201 (6)	
Na7	0.49029 (13)	−0.0001 (2)	0.25056 (12)	0.0252 (7)	
Na8	0.35025 (14)	0.33251 (19)	0.27872 (12)	0.0238 (7)	
Na9	0.34628 (13)	0.52516 (18)	0.30911 (12)	0.0179 (6)	
Na10	0.15902 (13)	−0.0242 (2)	0.33386 (13)	0.0291 (8)	
O1	0.8976 (2)	0.0496 (3)	0.32412 (18)	0.0128 (10)	
O2	0.9560 (2)	0.0765 (3)	0.27868 (19)	0.0176 (11)	
O3	0.8974 (2)	0.3095 (3)	0.3160 (2)	0.0207 (12)	
O4	0.81116 (19)	0.1859 (3)	0.30139 (17)	0.0137 (10)	
O5	0.8275 (2)	0.3118 (3)	0.35571 (19)	0.0164 (11)	
O6	0.67994 (19)	0.2196 (3)	0.33021 (18)	0.0150 (10)	
O7	0.85069 (18)	0.1365 (3)	0.37805 (17)	0.0102 (10)	
O8	0.9213 (2)	0.2395 (3)	0.40025 (19)	0.0182 (11)	
O9	0.9299 (2)	0.0391 (4)	0.49616 (19)	0.0215 (12)	
O10	0.73712 (19)	0.0603 (3)	0.26902 (17)	0.0125 (10)	
O11	0.6886 (2)	0.1963 (3)	0.24107 (18)	0.0166 (11)	
O12	0.7497 (2)	0.0839 (4)	0.14428 (19)	0.0267 (13)	
O13	0.74269 (19)	0.0688 (3)	0.34674 (16)	0.0113 (10)	
O14	0.6783 (2)	0.0283 (3)	0.39335 (17)	0.0164 (11)	
O15	0.8234 (2)	0.0806 (4)	0.46771 (18)	0.0187 (11)	
O16	0.7614 (2)	−0.1142 (3)	0.32209 (17)	0.0139 (10)	
O17	0.7900 (2)	−0.2541 (3)	0.29179 (18)	0.0183 (11)	
O18	0.7272 (2)	−0.0784 (4)	0.1908 (2)	0.0220 (13)	
O19	0.8212 (2)	0.0179 (3)	0.24434 (17)	0.0153 (11)	
O20	0.8128 (2)	−0.0431 (4)	0.16872 (19)	0.0277 (14)	
O21	0.9559 (2)	−0.0894 (4)	0.25405 (19)	0.0221 (12)	
O22	0.8445 (2)	−0.0289 (3)	0.39716 (17)	0.0114 (10)	
O23	0.8638 (2)	−0.0867 (4)	0.47548 (19)	0.0235 (13)	
O24	0.8016 (2)	−0.2788 (3)	0.37742 (18)	0.0180 (11)	
O25	0.84551 (19)	−0.1060 (3)	0.31495 (17)	0.0119 (10)	
O26	0.8835 (2)	−0.2399 (3)	0.35533 (19)	0.0190 (11)	
O27	0.9860 (2)	−0.0562 (4)	0.33733 (19)	0.0197 (12)	
O28	0.3471 (2)	−0.0205 (3)	0.21348 (18)	0.0140 (10)	
O29	0.3193 (2)	0.0365 (3)	0.13468 (19)	0.0208 (12)	
O30	0.3848 (2)	0.2321 (3)	0.2285 (2)	0.0222 (12)	
O31	0.4281 (2)	0.0685 (3)	0.28573 (19)	0.0171 (11)	
O32	0.4012 (2)	0.2111 (3)	0.3141 (2)	0.0201 (12)	
O33	0.4636 (2)	0.0429 (4)	0.4185 (2)	0.0217 (12)	
O34	0.3444 (2)	0.0624 (3)	0.29563 (17)	0.0136 (10)	
O35	0.3060 (2)	0.1957 (3)	0.25542 (19)	0.0171 (11)	
O36	0.2043 (2)	0.0144 (3)	0.27650 (19)	0.0172 (11)	
O37	0.34034 (19)	−0.1846 (3)	0.23649 (16)	0.0111 (10)	
O38	0.2690 (2)	−0.2846 (3)	0.21651 (18)	0.0161 (11)	
O39	0.2637 (2)	−0.0984 (4)	0.1149 (2)	0.0270 (13)	
O40	0.44533 (19)	−0.1212 (3)	0.26508 (17)	0.0114 (10)	
O41	0.5083 (2)	−0.1374 (4)	0.21115 (19)	0.0227 (12)	
O42	0.3703 (2)	−0.1173 (4)	0.1388 (2)	0.0242 (12)	
O43	0.2932 (2)	−0.0924 (3)	0.29054 (18)	0.0126 (10)	
O44	0.2347 (2)	−0.1168 (4)	0.33610 (19)	0.0186 (11)	
O45	0.2924 (2)	−0.3531 (3)	0.3018 (2)	0.0174 (11)	
O46	0.45300 (18)	−0.1033 (3)	0.34447 (16)	0.0104 (9)	
O47	0.5053 (2)	−0.2333 (3)	0.37587 (18)	0.0151 (10)	
O48	0.4479 (2)	−0.1091 (4)	0.47249 (19)	0.0274 (14)	
O49	0.3706 (2)	−0.0545 (3)	0.36934 (18)	0.0158 (10)	
O50	0.3784 (2)	0.0020 (4)	0.44562 (18)	0.0230 (12)	
O51	0.2363 (2)	0.0513 (4)	0.35960 (19)	0.0184 (11)	
O52	0.37844 (18)	−0.2286 (3)	0.31410 (17)	0.0110 (10)	
O53	0.3623 (2)	−0.3577 (3)	0.26194 (19)	0.0146 (10)	
O54	0.5121 (2)	−0.2716 (3)	0.29024 (19)	0.0173 (11)	
O55	0.6295 (2)	−0.0559 (4)	0.2636 (2)	0.0262 (13)	
O56	0.6486 (2)	0.0782 (3)	0.28781 (18)	0.0156 (11)	
O57	0.5551 (2)	0.0218 (3)	0.3193 (2)	0.0224 (12)	
O58	0.53815 (19)	−0.1197 (3)	0.31944 (19)	0.0171 (11)	
N1	0.9073 (2)	0.1380 (4)	0.3175 (2)	0.0134 (12)	
N2	0.7705 (2)	0.2173 (4)	0.3156 (2)	0.0098 (12)	
N3	0.8861 (2)	0.1186 (4)	0.4200 (2)	0.0115 (12)	
N4	0.7337 (2)	0.0861 (4)	0.2244 (2)	0.0154 (13)	
N5	0.7502 (2)	0.0913 (4)	0.3918 (2)	0.0119 (12)	
H5N	0.7806 (16)	0.111 (5)	0.404 (3)	0.014*	
N6	0.7442 (3)	−0.1345 (4)	0.2761 (2)	0.0168 (13)	
H6N	0.725 (3)	−0.098 (5)	0.258 (3)	0.020*	
N7	0.8646 (2)	−0.0230 (4)	0.2390 (2)	0.0157 (13)	
N8	0.8333 (2)	−0.1176 (4)	0.4014 (2)	0.0135 (12)	
N9	0.8962 (2)	−0.1180 (4)	0.3194 (2)	0.0166 (13)	
N10	0.3533 (3)	0.0695 (4)	0.2077 (2)	0.0155 (13)	
N11	0.4464 (2)	0.0903 (4)	0.3310 (2)	0.0169 (13)	
H11N	0.458 (3)	0.044 (4)	0.347 (3)	0.020*	
N12	0.2946 (2)	0.0756 (4)	0.2930 (2)	0.0126 (4)	
N13	0.3064 (2)	−0.1707 (4)	0.1939 (2)	0.0134 (12)	
N14	0.4341 (2)	−0.1648 (4)	0.2238 (2)	0.0172 (13)	
H14N	0.4042 (17)	−0.182 (6)	0.208 (3)	0.021*	
N15	0.2837 (2)	−0.1802 (4)	0.2977 (2)	0.0124 (12)	
N16	0.4576 (2)	−0.1241 (4)	0.3903 (2)	0.0143 (12)	
N17	0.3264 (2)	−0.0183 (4)	0.3753 (2)	0.0140 (12)	
N18	0.4207 (2)	−0.2633 (4)	0.3031 (2)	0.0116 (12)	
C1	0.9391 (3)	0.1456 (5)	0.2935 (3)	0.0181 (16)	
C2	0.9548 (3)	0.2326 (6)	0.2841 (3)	0.0250 (19)	
C3	0.9343 (3)	0.3092 (5)	0.2957 (3)	0.0229 (18)	
C4	0.9527 (4)	0.3886 (6)	0.2858 (4)	0.040 (3)	
H4	0.940061	0.441007	0.294685	0.048*	
C5	0.9889 (4)	0.3931 (7)	0.2634 (5)	0.055 (4)	
H5	1.001295	0.447966	0.257488	0.066*	
C6	1.0072 (4)	0.3169 (7)	0.2496 (5)	0.049 (3)	
H6	1.030676	0.320042	0.232747	0.058*	
C7	0.9915 (4)	0.2381 (6)	0.2600 (4)	0.035 (2)	
H7	1.004864	0.186345	0.251368	0.041*	
C8	0.7827 (3)	0.2827 (5)	0.3440 (3)	0.0134 (14)	
C9	0.7454 (3)	0.3214 (4)	0.3635 (3)	0.0132 (14)	
C10	0.6970 (3)	0.2880 (4)	0.3555 (3)	0.0137 (14)	
C11	0.6646 (3)	0.3309 (5)	0.3759 (3)	0.0181 (15)	
H11	0.631570	0.310415	0.370333	0.022*	
C12	0.6799 (3)	0.4024 (5)	0.4038 (3)	0.0246 (18)	
H12	0.657356	0.429516	0.417426	0.030*	
C13	0.7275 (4)	0.4351 (5)	0.4121 (3)	0.028 (2)	
H13	0.737797	0.484209	0.431233	0.034*	
C14	0.7602 (3)	0.3941 (5)	0.3918 (3)	0.0206 (16)	
H14	0.792899	0.415786	0.397271	0.025*	
C15	0.9209 (3)	0.1781 (5)	0.4288 (3)	0.0165 (15)	
C16	0.9586 (3)	0.1756 (5)	0.4735 (3)	0.0184 (16)	
C17	0.9597 (3)	0.1070 (6)	0.5045 (3)	0.0213 (16)	
C18	0.9965 (3)	0.1117 (7)	0.5470 (3)	0.033 (2)	
H18	0.999069	0.066389	0.568542	0.039*	
C19	1.0285 (3)	0.1816 (7)	0.5572 (3)	0.037 (2)	
H19	1.052646	0.183296	0.585874	0.045*	
C20	1.0267 (3)	0.2488 (7)	0.5271 (4)	0.035 (2)	
H20	1.048707	0.296802	0.535084	0.042*	
C21	0.9920 (3)	0.2448 (6)	0.4852 (3)	0.0265 (18)	
H21	0.990884	0.289897	0.463807	0.032*	
C22	0.7075 (3)	0.1562 (5)	0.2129 (2)	0.0162 (16)	
C23	0.6974 (3)	0.1901 (5)	0.1661 (3)	0.0198 (16)	
C24	0.7207 (3)	0.1519 (6)	0.1353 (3)	0.0245 (18)	
C25	0.7098 (4)	0.1893 (6)	0.0913 (3)	0.034 (2)	
H25	0.725971	0.167833	0.070179	0.041*	
C26	0.6762 (4)	0.2563 (7)	0.0789 (3)	0.041 (3)	
H26	0.669047	0.279221	0.048996	0.050*	
C27	0.6526 (4)	0.2911 (6)	0.1088 (3)	0.035 (2)	
H27	0.629090	0.336725	0.099711	0.042*	
C28	0.6641 (4)	0.2578 (6)	0.1521 (3)	0.031 (2)	
H28	0.648683	0.281929	0.173076	0.037*	
C29	0.7197 (3)	0.0578 (5)	0.4139 (2)	0.0140 (14)	
C30	0.7382 (3)	0.0560 (5)	0.4642 (3)	0.0167 (15)	
C31	0.7883 (3)	0.0584 (5)	0.4882 (3)	0.0179 (15)	
C32	0.8037 (3)	0.0385 (6)	0.5343 (3)	0.030 (2)	
H32	0.837951	0.036725	0.550155	0.036*	
C33	0.7683 (4)	0.0212 (8)	0.5571 (3)	0.040 (3)	
H33	0.778404	0.008400	0.588636	0.049*	
C34	0.7189 (4)	0.0228 (7)	0.5341 (3)	0.037 (2)	
H34	0.695060	0.012386	0.549969	0.044*	
C35	0.7034 (3)	0.0392 (6)	0.4881 (3)	0.0257 (18)	
H35	0.669105	0.039081	0.472543	0.031*	
C36	0.7610 (3)	−0.2050 (5)	0.2621 (3)	0.0163 (15)	
C37	0.7478 (3)	−0.2258 (6)	0.2135 (3)	0.0248 (18)	
C38	0.7328 (3)	−0.1615 (6)	0.1804 (3)	0.0243 (18)	
C39	0.7252 (4)	−0.1850 (7)	0.1346 (3)	0.036 (2)	
H39	0.716413	−0.141964	0.111473	0.043*	
C40	0.7307 (5)	−0.2714 (8)	0.1232 (4)	0.050 (3)	
H40	0.724950	−0.286743	0.092236	0.060*	
C41	0.7440 (5)	−0.3339 (7)	0.1553 (4)	0.048 (3)	
H41	0.747121	−0.392579	0.146723	0.057*	
C42	0.7533 (4)	−0.3122 (6)	0.2011 (3)	0.037 (2)	
H42	0.763221	−0.355794	0.223749	0.045*	
C43	0.8560 (3)	−0.0535 (6)	0.1973 (3)	0.0209 (16)	
C44	0.8945 (3)	−0.0981 (6)	0.1821 (3)	0.0230 (17)	
C45	0.9420 (3)	−0.1133 (5)	0.2108 (3)	0.0242 (17)	
C46	0.9761 (4)	−0.1562 (6)	0.1925 (3)	0.0298 (19)	
H46	1.008329	−0.168199	0.211693	0.036*	
C47	0.9644 (4)	−0.1809 (7)	0.1484 (3)	0.040 (2)	
H47	0.988592	−0.208250	0.136888	0.048*	
C48	0.9171 (4)	−0.1663 (7)	0.1200 (3)	0.041 (2)	
H48	0.908747	−0.183109	0.088966	0.049*	
C49	0.8821 (4)	−0.1267 (7)	0.1376 (3)	0.034 (2)	
H49	0.849206	−0.119195	0.118738	0.041*	
C50	0.8447 (3)	−0.1430 (5)	0.4438 (3)	0.0186 (15)	0.56 (5)
C51	0.8378 (6)	−0.2342 (10)	0.4540 (8)	0.021 (3)	0.56 (5)
C52	0.8168 (9)	−0.2964 (12)	0.4207 (9)	0.020 (3)	0.56 (5)
C53	0.8124 (10)	−0.3824 (14)	0.4346 (8)	0.024 (4)	0.56 (5)
H53	0.799707	−0.425998	0.412468	0.029*	0.56 (5)
C54	0.8266 (12)	−0.4047 (13)	0.4806 (8)	0.027 (4)	0.56 (5)
H54	0.821637	−0.462466	0.489461	0.033*	0.56 (5)
C55	0.8475 (12)	−0.3440 (14)	0.5130 (8)	0.030 (4)	0.56 (5)
H55	0.857553	−0.359333	0.544242	0.036*	0.56 (5)
C56	0.8537 (11)	−0.2603 (13)	0.4994 (8)	0.025 (4)	0.56 (5)
H56	0.869456	−0.218734	0.521761	0.030*	0.56 (5)
C50B	0.8447 (3)	−0.1430 (5)	0.4438 (3)	0.0186 (15)	0.44 (5)
C51B	0.8326 (7)	−0.2302 (11)	0.4559 (10)	0.022 (3)	0.44 (5)
C52B	0.8119 (12)	−0.2921 (16)	0.4225 (11)	0.023 (3)	0.44 (5)
C53B	0.8014 (13)	−0.3747 (18)	0.4366 (10)	0.026 (4)	0.44 (5)
H53B	0.787640	−0.417642	0.414387	0.031*	0.44 (5)
C54B	0.8105 (14)	−0.3956 (17)	0.4826 (10)	0.028 (4)	0.44 (5)
H54B	0.802303	−0.451621	0.491327	0.034*	0.44 (5)
C55B	0.8317 (13)	−0.3341 (17)	0.5153 (9)	0.027 (4)	0.44 (5)
H55B	0.839483	−0.348043	0.546606	0.033*	0.44 (5)
C56B	0.8412 (12)	−0.2522 (17)	0.5015 (10)	0.024 (4)	0.44 (5)
H56B	0.854153	−0.209173	0.523898	0.029*	0.44 (5)
C57	0.9138 (3)	−0.1878 (5)	0.3423 (3)	0.0194 (16)	
C58	0.9673 (3)	−0.2029 (5)	0.3539 (3)	0.0264 (19)	
C59	1.0005 (3)	−0.1348 (5)	0.3524 (3)	0.0215 (17)	
C60	1.0508 (4)	−0.1514 (7)	0.3690 (3)	0.033 (2)	
H60	1.073651	−0.106101	0.368710	0.039*	
C61	1.0688 (4)	−0.2323 (8)	0.3860 (5)	0.053 (3)	
H61	1.103428	−0.240802	0.398379	0.063*	
C62	1.0364 (4)	−0.3009 (8)	0.3851 (6)	0.066 (4)	
H62	1.048628	−0.357427	0.394705	0.079*	
C63	0.9858 (4)	−0.2853 (7)	0.3699 (5)	0.049 (3)	
H63	0.963351	−0.331136	0.370365	0.059*	
C64	0.3385 (3)	0.0930 (5)	0.1656 (3)	0.0206 (17)	
C65	0.3416 (4)	0.1839 (6)	0.1529 (3)	0.0274 (19)	
C66	0.3650 (4)	0.2476 (5)	0.1841 (3)	0.0268 (19)	
C67	0.3685 (4)	0.3325 (6)	0.1687 (4)	0.036 (2)	
H67	0.384339	0.376109	0.189652	0.043*	
C68	0.3490 (5)	0.3538 (7)	0.1229 (4)	0.051 (3)	
H68	0.352825	0.411145	0.112758	0.061*	
C69	0.3243 (6)	0.2917 (7)	0.0923 (4)	0.054 (3)	
H69	0.309742	0.306646	0.061406	0.065*	
C70	0.3209 (5)	0.2081 (7)	0.1070 (4)	0.043 (3)	
H70	0.304234	0.165397	0.085801	0.052*	
C71	0.4305 (3)	0.1624 (5)	0.3447 (3)	0.0190 (16)	
C72	0.4447 (3)	0.1895 (5)	0.3926 (3)	0.0233 (17)	
C73	0.4586 (3)	0.1270 (6)	0.4272 (3)	0.0279 (19)	
C74	0.4695 (4)	0.1581 (6)	0.4720 (3)	0.037 (2)	
H74	0.480211	0.118426	0.496509	0.044*	
C75	0.4649 (5)	0.2461 (8)	0.4810 (4)	0.051 (3)	
H75	0.473410	0.265821	0.511501	0.061*	
C76	0.4481 (5)	0.3053 (7)	0.4457 (4)	0.051 (3)	
H76	0.443615	0.364735	0.452048	0.061*	
C77	0.4381 (4)	0.2773 (6)	0.4024 (4)	0.035 (2)	
H77	0.426438	0.317458	0.378247	0.043*	
C78	0.2763 (3)	0.1447 (5)	0.2705 (3)	0.0161 (15)	
C79	0.2231 (3)	0.1622 (5)	0.2606 (3)	0.0227 (16)	
C80	0.1900 (3)	0.0943 (5)	0.2623 (3)	0.0198 (16)	
C81	0.1393 (3)	0.1121 (6)	0.2470 (3)	0.0285 (19)	
H81	0.116232	0.066615	0.246038	0.034*	
C82	0.1221 (4)	0.1959 (8)	0.2332 (4)	0.047 (3)	
H82	0.087584	0.207296	0.224086	0.057*	
C83	0.1542 (5)	0.2604 (7)	0.2327 (5)	0.058 (3)	
H83	0.142150	0.316942	0.223011	0.069*	
C84	0.2054 (4)	0.2450 (7)	0.2462 (4)	0.044 (3)	
H84	0.227787	0.290830	0.245588	0.053*	
C85	0.2703 (3)	−0.2270 (5)	0.1865 (3)	0.0156 (15)	
C86	0.2305 (3)	−0.2243 (5)	0.1425 (3)	0.0175 (15)	
C87	0.2300 (3)	−0.1600 (6)	0.1101 (3)	0.0235 (17)	
C88	0.1899 (3)	−0.1615 (7)	0.0701 (3)	0.030 (2)	
H88	0.187986	−0.117896	0.047706	0.036*	
C89	0.1536 (3)	−0.2244 (7)	0.0626 (3)	0.034 (2)	
H89	0.127217	−0.223590	0.035358	0.041*	
C90	0.1553 (3)	−0.2888 (6)	0.0945 (3)	0.032 (2)	
H90	0.130735	−0.332959	0.089149	0.038*	
C91	0.1937 (3)	−0.2874 (6)	0.1347 (3)	0.0243 (17)	
H91	0.194860	−0.330378	0.157127	0.029*	
C92	0.4649 (3)	−0.1659 (5)	0.1984 (3)	0.0195 (16)	
C93	0.4427 (3)	−0.2061 (5)	0.1534 (3)	0.0214 (16)	
C94	0.3956 (3)	−0.1795 (5)	0.1262 (3)	0.0233 (17)	
C95	0.3777 (4)	−0.2186 (7)	0.0830 (3)	0.040 (2)	
H95	0.346908	−0.200295	0.063247	0.049*	
C96	0.4044 (5)	−0.2830 (8)	0.0691 (4)	0.055 (3)	
H96	0.390759	−0.310428	0.040618	0.066*	
C97	0.4513 (5)	−0.3088 (8)	0.0963 (3)	0.051 (3)	
H97	0.469745	−0.352140	0.086100	0.061*	
C98	0.4700 (4)	−0.2698 (6)	0.1381 (3)	0.0310 (19)	
H98	0.501676	−0.286246	0.156821	0.037*	
C99	0.2526 (3)	−0.1866 (5)	0.3226 (3)	0.0142 (15)	
C100	0.2372 (3)	−0.2718 (5)	0.3349 (3)	0.0231 (17)	
C101	0.2580 (3)	−0.3490 (6)	0.3240 (3)	0.0269 (19)	
C102	0.2418 (4)	−0.4277 (6)	0.3373 (4)	0.043 (3)	
H102	0.254739	−0.480812	0.329603	0.051*	
C103	0.2067 (5)	−0.4294 (7)	0.3618 (6)	0.064 (4)	
H103	0.196187	−0.483572	0.370755	0.077*	
C104	0.1875 (5)	−0.3528 (7)	0.3731 (5)	0.059 (4)	
H104	0.164373	−0.353859	0.390345	0.071*	
C105	0.2023 (4)	−0.2747 (6)	0.3589 (4)	0.037 (2)	
H105	0.188256	−0.222011	0.365687	0.044*	
C106	0.4858 (3)	−0.1927 (5)	0.4035 (3)	0.0164 (15)	
C107	0.4975 (3)	−0.2208 (5)	0.4510 (3)	0.0193 (16)	
C108	0.4783 (3)	−0.1771 (5)	0.4828 (3)	0.0227 (17)	
C109	0.4934 (4)	−0.2055 (6)	0.5280 (3)	0.0281 (19)	
H109	0.481386	−0.176597	0.550112	0.034*	
C110	0.5255 (3)	−0.2747 (6)	0.5409 (3)	0.0289 (19)	
H110	0.534785	−0.293367	0.571614	0.035*	
C111	0.5445 (4)	−0.3175 (6)	0.5096 (3)	0.0292 (19)	
H111	0.567074	−0.364417	0.518800	0.035*	
C112	0.5299 (3)	−0.2905 (5)	0.4649 (3)	0.0228 (17)	
H112	0.542163	−0.320105	0.443210	0.027*	
C113	0.3345 (3)	0.0097 (5)	0.4168 (3)	0.0173 (15)	
C114	0.2950 (3)	0.0491 (5)	0.4333 (3)	0.0194 (16)	
C115	0.2479 (3)	0.0689 (5)	0.4025 (3)	0.0177 (16)	
C116	0.2132 (3)	0.1122 (6)	0.4206 (3)	0.0263 (18)	
H116	0.182229	0.129790	0.400910	0.032*	
C117	0.2240 (4)	0.1293 (7)	0.4670 (3)	0.032 (2)	
H117	0.200007	0.156941	0.478562	0.038*	
C118	0.2692 (4)	0.1066 (7)	0.4963 (3)	0.033 (2)	
H118	0.275919	0.116735	0.527913	0.039*	
C119	0.3048 (4)	0.0685 (6)	0.4788 (3)	0.0268 (19)	
H119	0.336482	0.055572	0.498730	0.032*	
C120	0.4081 (3)	−0.3295 (4)	0.2749 (2)	0.0124 (14)	
C121	0.4455 (3)	−0.3723 (4)	0.2566 (3)	0.0147 (15)	
C122	0.4940 (3)	−0.3419 (4)	0.2655 (2)	0.0129 (14)	
C123	0.5268 (3)	−0.3867 (5)	0.2460 (3)	0.0208 (16)	
H123	0.560403	−0.368785	0.252893	0.025*	
C124	0.5105 (4)	−0.4571 (5)	0.2168 (3)	0.0270 (19)	
H124	0.532640	−0.485761	0.203283	0.032*	
C125	0.4616 (3)	−0.4850 (5)	0.2076 (3)	0.0257 (18)	
H125	0.450298	−0.532496	0.187535	0.031*	
C126	0.4297 (3)	−0.4438 (5)	0.2274 (3)	0.0196 (16)	
H126	0.396580	−0.463855	0.221381	0.023*	
C127	0.6279 (3)	0.0224 (5)	0.2570 (3)	0.0169 (16)	
C128	0.5987 (4)	0.0559 (8)	0.2111 (3)	0.044 (3)	
H12A	0.618917	0.052539	0.190024	0.065*	
H12B	0.589190	0.116586	0.213954	0.065*	
H12C	0.568816	0.020486	0.199654	0.065*	
C129	0.5537 (3)	−0.0481 (5)	0.3404 (3)	0.0195 (16)	
C130	0.5702 (3)	−0.0492 (6)	0.3907 (3)	0.0253 (17)	
H13A	0.543190	−0.029068	0.402462	0.038*	
H13B	0.598803	−0.010644	0.401607	0.038*	
H13C	0.579472	−0.108608	0.401343	0.038*	
O59	1.0914 (2)	0.0647 (4)	0.3351 (2)	0.0342 (15)	
C131	1.0804 (5)	0.1388 (9)	0.3458 (5)	0.096 (7)	
H131	1.085572	0.183843	0.326472	0.115*	
N19	1.0636 (5)	0.1649 (7)	0.3777 (4)	0.087 (5)	
C132	1.0488 (9)	0.0966 (13)	0.4065 (8)	0.134 (10)	
H13D	1.064828	0.041273	0.403273	0.202*	
H13E	1.012828	0.089141	0.396619	0.202*	
H13F	1.059234	0.114808	0.438357	0.202*	
C133	1.0464 (7)	0.2476 (9)	0.3859 (7)	0.104 (7)	
H13G	1.055764	0.290742	0.366342	0.156*	
H13H	1.061132	0.263479	0.417767	0.156*	
H13I	1.010280	0.246200	0.379324	0.156*	
O60	1.0224 (3)	0.0561 (5)	0.2059 (3)	0.0407 (15)	0.847 (14)
C134	0.9838 (5)	0.0608 (9)	0.1760 (4)	0.044 (3)	0.847 (14)
H134	0.955308	0.038096	0.182698	0.053*	0.847 (14)
N20	0.9773 (5)	0.0940 (10)	0.1354 (4)	0.048 (3)	0.847 (14)
C135	1.0222 (6)	0.1164 (16)	0.1240 (6)	0.086 (5)	0.847 (14)
H13J	1.021008	0.177750	0.114981	0.129*	0.847 (14)
H13K	1.025254	0.079842	0.098750	0.129*	0.847 (14)
H13L	1.050864	0.106811	0.150371	0.129*	0.847 (14)
C136	0.9325 (6)	0.0939 (14)	0.0989 (6)	0.067 (6)	0.847 (14)
H13M	0.940467	0.088095	0.069995	0.101*	0.847 (14)
H13N	0.914693	0.148646	0.099084	0.101*	0.847 (14)
H13O	0.911649	0.045003	0.102563	0.101*	0.847 (14)
O60B	1.0224 (3)	0.0561 (5)	0.2059 (3)	0.0407 (15)	0.153 (14)
C224	0.997 (3)	0.050 (4)	0.1663 (11)	0.046 (4)	0.153 (14)
H224	0.994809	−0.008023	0.154573	0.056*	0.153 (14)
N20B	0.972 (2)	0.107 (4)	0.137 (2)	0.050 (5)	0.153 (14)
C225	0.991 (3)	0.196 (4)	0.137 (2)	0.052 (9)	0.153 (14)
H22J	0.968283	0.229975	0.113587	0.078*	0.153 (14)
H22K	0.994248	0.222847	0.166992	0.078*	0.153 (14)
H22L	1.023767	0.194443	0.131820	0.078*	0.153 (14)
C226	0.922 (2)	0.094 (6)	0.110 (3)	0.054 (11)	0.153 (14)
H22M	0.910643	0.145405	0.090972	0.081*	0.153 (14)
H22N	0.919784	0.042599	0.091352	0.081*	0.153 (14)
H22O	0.900710	0.086668	0.130488	0.081*	0.153 (14)
O61	0.8169 (3)	0.3819 (5)	0.2272 (2)	0.0382 (16)	
C137	0.8237 (4)	0.3054 (6)	0.2193 (3)	0.033 (2)	
H137	0.810363	0.262746	0.234897	0.039*	
N21	0.8480 (4)	0.2768 (5)	0.1907 (3)	0.040 (2)	
C138	0.8732 (5)	0.3335 (8)	0.1666 (5)	0.054 (3)	
H13P	0.865353	0.315357	0.134811	0.080*	
H13Q	0.908965	0.329775	0.180613	0.080*	
H13R	0.862186	0.393604	0.168231	0.080*	
C139	0.8578 (5)	0.1850 (6)	0.1857 (4)	0.053 (3)	
H13S	0.842965	0.167338	0.154074	0.079*	
H13T	0.843368	0.150544	0.205564	0.079*	
H13U	0.893581	0.175259	0.193946	0.079*	
O62	0.8686 (3)	0.5582 (4)	0.2765 (2)	0.0355 (15)	
C140	0.8671 (4)	0.5658 (6)	0.2366 (3)	0.036 (2)	
H140	0.856046	0.516793	0.217527	0.043*	
N22	0.8791 (4)	0.6342 (6)	0.2179 (3)	0.048 (2)	
C141	0.8896 (7)	0.7159 (8)	0.2432 (5)	0.074 (4)	
H14A	0.922459	0.736929	0.243461	0.111*	
H14B	0.888539	0.706225	0.274466	0.111*	
H14C	0.864647	0.759399	0.228727	0.111*	
C142	0.8764 (7)	0.6414 (11)	0.1697 (4)	0.077 (4)	
H14D	0.870481	0.583704	0.155580	0.116*	
H14E	0.907655	0.664703	0.166654	0.116*	
H14F	0.849244	0.680468	0.154674	0.116*	
O63	0.7781 (2)	0.5234 (4)	0.3059 (2)	0.0278 (13)	0.778 (14)
C143	0.7343 (4)	0.5391 (7)	0.3031 (4)	0.030 (2)	0.778 (14)
H143	0.727791	0.587715	0.319587	0.036*	0.778 (14)
N23	0.6958 (4)	0.4942 (8)	0.2791 (5)	0.032 (3)	0.778 (14)
C144	0.7014 (5)	0.4212 (7)	0.2519 (4)	0.033 (3)	0.778 (14)
H14G	0.676144	0.423915	0.222536	0.050*	0.778 (14)
H14H	0.697549	0.367124	0.267473	0.050*	0.778 (14)
H14I	0.734344	0.422765	0.247202	0.050*	0.778 (14)
C145	0.6470 (5)	0.5128 (10)	0.2839 (6)	0.045 (3)	0.778 (14)
H14J	0.624468	0.527498	0.254174	0.068*	0.778 (14)
H14K	0.649128	0.562048	0.304653	0.068*	0.778 (14)
H14L	0.634496	0.461591	0.296115	0.068*	0.778 (14)
O63B	0.7781 (2)	0.5234 (4)	0.3059 (2)	0.0278 (13)	0.222 (14)
C203	0.7348 (16)	0.478 (2)	0.2894 (12)	0.031 (4)	0.222 (14)
H203	0.734834	0.420148	0.278641	0.037*	0.222 (14)
N23B	0.696 (2)	0.515 (4)	0.289 (2)	0.036 (5)	0.222 (14)
C204	0.6888 (19)	0.600 (3)	0.3156 (18)	0.046 (8)	0.222 (14)
H20G	0.664469	0.638245	0.295326	0.068*	0.222 (14)
H20H	0.720550	0.630400	0.326126	0.068*	0.222 (14)
H20I	0.677166	0.584106	0.341679	0.068*	0.222 (14)
C205	0.646 (2)	0.478 (3)	0.271 (2)	0.047 (9)	0.222 (14)
H20J	0.637228	0.475857	0.237763	0.070*	0.222 (14)
H20K	0.621618	0.515123	0.279936	0.070*	0.222 (14)
H20L	0.644837	0.419516	0.283015	0.070*	0.222 (14)
O64	0.8835 (12)	0.513 (3)	0.3748 (10)	0.034 (3)	0.661 (12)
C146	0.9040 (6)	0.4754 (9)	0.4108 (5)	0.035 (3)	0.661 (12)
H146	0.901121	0.413894	0.411659	0.042*	0.661 (12)
N24	0.9299 (5)	0.5151 (7)	0.4481 (4)	0.040 (3)	0.661 (12)
C147	0.9350 (8)	0.6095 (10)	0.4516 (8)	0.045 (5)	0.661 (12)
H14M	0.955526	0.625195	0.482019	0.067*	0.661 (12)
H14O	0.950698	0.630651	0.428941	0.067*	0.661 (12)
H14P	0.902241	0.636123	0.446142	0.067*	0.661 (12)
C148	0.9506 (9)	0.4684 (11)	0.4909 (5)	0.060 (5)	0.661 (12)
H14Q	0.939066	0.407929	0.487562	0.090*	0.661 (12)
H14R	0.939811	0.496530	0.515105	0.090*	0.661 (12)
H14S	0.986816	0.469445	0.498702	0.090*	0.661 (12)
O64B	0.886 (2)	0.509 (6)	0.374 (2)	0.035 (5)	0.339 (12)
C230	0.9249 (12)	0.4738 (18)	0.3961 (10)	0.043 (4)	0.339 (12)
H230	0.931785	0.417619	0.386592	0.052*	0.339 (12)
N24B	0.9575 (9)	0.5076 (16)	0.4315 (9)	0.048 (4)	0.339 (12)
C231	0.9467 (17)	0.589 (3)	0.4510 (17)	0.050 (8)	0.339 (12)
H23A	0.974710	0.605083	0.476929	0.076*	0.339 (12)
H23B	0.941183	0.635345	0.428058	0.076*	0.339 (12)
H23C	0.916942	0.582314	0.461174	0.076*	0.339 (12)
C232	1.0086 (10)	0.478 (2)	0.4464 (18)	0.097 (13)	0.339 (12)
H23D	1.026621	0.511612	0.473145	0.146*	0.339 (12)
H23E	1.009552	0.416273	0.454108	0.146*	0.339 (12)
H23F	1.024166	0.487071	0.421952	0.146*	0.339 (12)
O65	0.6446 (2)	−0.1883 (4)	0.3423 (2)	0.0294 (13)	
C149	0.6367 (4)	−0.2469 (6)	0.3666 (3)	0.036 (2)	
H149	0.610835	−0.286790	0.353444	0.043*	
N25	0.6615 (3)	−0.2585 (6)	0.4098 (3)	0.044 (2)	
C150	0.7014 (6)	−0.1986 (10)	0.4315 (5)	0.075 (4)	
H15A	0.721410	−0.186466	0.410791	0.112*	
H15B	0.722338	−0.224949	0.459407	0.112*	
H15C	0.687340	−0.144126	0.438886	0.112*	
C151	0.6527 (6)	−0.3326 (9)	0.4344 (5)	0.070 (4)	
H15D	0.629576	−0.372000	0.413672	0.106*	
H15E	0.638429	−0.313734	0.458419	0.106*	
H15F	0.683974	−0.362928	0.447855	0.106*	
O66	0.5496 (3)	0.2316 (5)	0.3114 (3)	0.056 (2)	
C152	0.5219 (4)	0.2293 (8)	0.2724 (4)	0.055 (3)	
H152	0.490666	0.202017	0.267865	0.066*	
N26	0.5327 (3)	0.2625 (6)	0.2354 (3)	0.047 (2)	
C153	0.5805 (4)	0.3008 (8)	0.2417 (5)	0.055 (3)	
H15G	0.601016	0.261806	0.229445	0.082*	
H15H	0.596182	0.309977	0.274155	0.082*	
H15I	0.576890	0.356753	0.225826	0.082*	
C154	0.4991 (5)	0.2577 (9)	0.1906 (5)	0.068 (4)	
H15J	0.516341	0.233654	0.169759	0.102*	
H15K	0.486885	0.316125	0.180532	0.102*	
H15L	0.471156	0.220027	0.190967	0.102*	
O67	0.6020 (3)	0.1686 (5)	0.4161 (2)	0.0477 (18)	
C155	0.5826 (4)	0.2299 (6)	0.4286 (3)	0.041 (2)	
H155	0.550020	0.244367	0.411071	0.050*	
N27	0.6028 (4)	0.2795 (6)	0.4650 (3)	0.051 (2)	
C156	0.6509 (5)	0.2619 (9)	0.4939 (4)	0.070 (4)	
H15M	0.668277	0.222746	0.478410	0.105*	
H15N	0.648234	0.234415	0.521846	0.105*	
H15O	0.669378	0.316547	0.501287	0.105*	
C157	0.5745 (6)	0.3504 (9)	0.4772 (6)	0.076 (4)	
H15P	0.596726	0.399028	0.489605	0.115*	
H15Q	0.558835	0.330002	0.499993	0.115*	
H15R	0.548944	0.369879	0.450117	0.115*	
O68	0.6249 (3)	−0.2756 (4)	0.2364 (2)	0.0397 (15)	
C158	0.6215 (4)	−0.3084 (7)	0.1992 (3)	0.043 (2)	
H158	0.627473	−0.369126	0.198233	0.051*	
N28	0.6100 (3)	−0.2653 (5)	0.1604 (3)	0.0427 (19)	
C159	0.6027 (5)	−0.1714 (6)	0.1604 (4)	0.046 (2)	
H15S	0.594011	−0.149695	0.129118	0.069*	
H15T	0.633303	−0.143348	0.178149	0.069*	
H15U	0.575934	−0.157996	0.173829	0.069*	
C160	0.6009 (6)	−0.3070 (9)	0.1164 (4)	0.062 (3)	
H16A	0.570290	−0.283970	0.095793	0.093*	
H16B	0.597623	−0.369955	0.119772	0.093*	
H16C	0.628597	−0.295245	0.103872	0.093*	
O69	0.4676 (4)	0.0509 (11)	0.1757 (4)	0.038 (3)	0.806 (9)
C161	0.4733 (5)	0.0105 (8)	0.1426 (3)	0.036 (2)	0.806 (9)
H161	0.497269	−0.034587	0.149761	0.043*	0.806 (9)
N29	0.4512 (5)	0.0206 (11)	0.0993 (3)	0.031 (3)	0.806 (9)
C162	0.4080 (5)	0.0778 (9)	0.0847 (5)	0.049 (3)	0.806 (9)
H16D	0.406263	0.102362	0.054922	0.073*	0.806 (9)
H16E	0.410855	0.124986	0.106779	0.073*	0.806 (9)
H16F	0.377883	0.044183	0.082789	0.073*	0.806 (9)
C163	0.4602 (6)	−0.0361 (9)	0.0644 (4)	0.047 (3)	0.806 (9)
H16G	0.457106	−0.002456	0.036676	0.070*	0.806 (9)
H16H	0.435979	−0.083497	0.058091	0.070*	0.806 (9)
H16I	0.493672	−0.060449	0.075093	0.070*	0.806 (9)
O69B	0.4826 (17)	0.054 (5)	0.1792 (15)	0.034 (5)	0.194 (9)
C206	0.4470 (14)	0.049 (3)	0.1445 (11)	0.026 (4)	0.194 (9)
H206	0.416134	0.072606	0.145572	0.032*	0.194 (9)
N29B	0.4496 (18)	0.014 (5)	0.1063 (14)	0.032 (5)	0.194 (9)
C207	0.4928 (14)	−0.036 (3)	0.1045 (13)	0.033 (7)	0.194 (9)
H20M	0.482196	−0.089250	0.086804	0.050*	0.194 (9)
H20N	0.512161	−0.051616	0.135365	0.050*	0.194 (9)
H20O	0.513271	−0.001124	0.090126	0.050*	0.194 (9)
C208	0.4084 (14)	0.006 (3)	0.0660 (11)	0.032 (7)	0.194 (9)
H20P	0.379867	0.037624	0.070244	0.048*	0.194 (9)
H20Q	0.399757	−0.055658	0.060261	0.048*	0.194 (9)
H20R	0.417508	0.030330	0.039999	0.048*	0.194 (9)
O70	0.3042 (3)	0.4449 (4)	0.2389 (2)	0.0308 (14)	0.703 (14)
C164	0.2843 (6)	0.4833 (9)	0.2040 (4)	0.045 (3)	0.703 (14)
H164	0.289079	0.544474	0.204127	0.055*	0.703 (14)
N30	0.2571 (6)	0.4490 (9)	0.1662 (4)	0.056 (3)	0.703 (14)
C165	0.2439 (11)	0.3564 (10)	0.1634 (8)	0.060 (5)	0.703 (14)
H16J	0.208626	0.350206	0.160906	0.090*	0.703 (14)
H16K	0.263460	0.326541	0.190827	0.090*	0.703 (14)
H16L	0.250844	0.330563	0.136717	0.090*	0.703 (14)
C166	0.2348 (10)	0.4993 (13)	0.1254 (6)	0.100 (9)	0.703 (14)
H16M	0.216138	0.460369	0.101398	0.150*	0.703 (14)
H16N	0.212471	0.542989	0.131973	0.150*	0.703 (14)
H16O	0.260869	0.528417	0.115456	0.150*	0.703 (14)
O70B	0.3042 (3)	0.4449 (4)	0.2389 (2)	0.0308 (14)	0.297 (14)
C209	0.2617 (8)	0.474 (2)	0.2249 (10)	0.045 (4)	0.297 (14)
H209	0.252243	0.515957	0.243811	0.054*	0.297 (14)
N30B	0.2288 (9)	0.4550 (19)	0.1875 (9)	0.046 (4)	0.297 (14)
C210	0.243 (2)	0.385 (4)	0.1604 (17)	0.063 (9)	0.297 (14)
H21A	0.215329	0.375016	0.132841	0.095*	0.297 (14)
H21B	0.272311	0.401876	0.152068	0.095*	0.297 (14)
H21C	0.249072	0.331241	0.178371	0.095*	0.297 (14)
C211	0.1775 (10)	0.479 (2)	0.1707 (17)	0.077 (12)	0.297 (14)
H21D	0.163157	0.453068	0.140784	0.116*	0.297 (14)
H21E	0.159506	0.458877	0.191723	0.116*	0.297 (14)
H21F	0.174854	0.542894	0.168070	0.116*	0.297 (14)
O71	0.3226 (2)	0.3938 (4)	0.3375 (2)	0.0266 (13)	
C167	0.3241 (4)	0.3843 (5)	0.3779 (3)	0.0289 (18)	
H167	0.331322	0.434255	0.396797	0.035*	
N31	0.3166 (4)	0.3104 (5)	0.3968 (2)	0.0353 (18)	
C168	0.3090 (6)	0.2302 (6)	0.3710 (4)	0.052 (3)	
H16P	0.276002	0.207222	0.368765	0.078*	
H16Q	0.334071	0.187478	0.386250	0.078*	
H16R	0.311964	0.241519	0.340469	0.078*	
C169	0.3184 (6)	0.3031 (8)	0.4446 (3)	0.054 (3)	
H16S	0.286455	0.282278	0.447083	0.081*	
H16T	0.325607	0.360308	0.459073	0.081*	
H16U	0.344455	0.261975	0.459636	0.081*	
O72	0.4140 (2)	0.4342 (3)	0.3084 (2)	0.0230 (12)	
C170	0.4582 (3)	0.4227 (5)	0.3134 (3)	0.0282 (19)	
H170	0.466871	0.378288	0.295522	0.034*	
N32	0.4953 (3)	0.4660 (5)	0.3412 (3)	0.0303 (16)	
C171	0.4859 (4)	0.5334 (6)	0.3714 (3)	0.033 (2)	
H17A	0.491653	0.590847	0.360036	0.049*	
H17B	0.508212	0.525060	0.401981	0.049*	
H17C	0.451513	0.529370	0.372411	0.049*	
C172	0.5462 (4)	0.4514 (8)	0.3424 (4)	0.047 (3)	
H17D	0.558658	0.501817	0.329535	0.071*	
H17E	0.548495	0.399323	0.324712	0.071*	
H17F	0.566120	0.443012	0.373941	0.071*	
O73	0.3746 (3)	−0.4351 (4)	0.3886 (2)	0.0315 (14)	
C173	0.3713 (3)	−0.3566 (5)	0.3963 (3)	0.0262 (18)	
H173	0.384041	−0.317403	0.378566	0.031*	
N33	0.3520 (3)	−0.3218 (5)	0.4264 (3)	0.0353 (18)	
C174	0.3286 (7)	−0.3748 (8)	0.4533 (5)	0.077 (5)	
H17G	0.333660	−0.436463	0.447662	0.115*	
H17H	0.293120	−0.362160	0.444853	0.115*	
H17I	0.343303	−0.361923	0.485523	0.115*	
C175	0.3458 (5)	−0.2290 (6)	0.4292 (4)	0.049 (3)	
H17J	0.359024	−0.199926	0.406704	0.074*	
H17K	0.363612	−0.208617	0.459626	0.074*	
H17L	0.310544	−0.215302	0.422992	0.074*	
O74	0.1660 (6)	−0.0840 (10)	0.4046 (3)	0.031 (3)	0.818 (14)
C176	0.1682 (5)	−0.0725 (8)	0.4449 (3)	0.039 (2)	0.818 (14)
H176	0.141516	−0.042033	0.451182	0.047*	0.818 (14)
N34	0.2044 (4)	−0.0990 (8)	0.4802 (3)	0.041 (3)	0.818 (14)
C177	0.2472 (5)	−0.1439 (11)	0.4743 (5)	0.059 (4)	0.818 (14)
H17M	0.243633	−0.206604	0.478339	0.089*	0.818 (14)
H17N	0.277038	−0.122930	0.496900	0.089*	0.818 (14)
H17O	0.250126	−0.132709	0.443787	0.089*	0.818 (14)
C178	0.2041 (7)	−0.0797 (10)	0.5262 (4)	0.057 (4)	0.818 (14)
H17P	0.175613	−0.042724	0.525720	0.085*	0.818 (14)
H17Q	0.234689	−0.049373	0.542037	0.085*	0.818 (14)
H17R	0.201752	−0.134147	0.542154	0.085*	0.818 (14)
O74B	0.164 (3)	−0.099 (6)	0.3975 (16)	0.035 (5)	0.182 (14)
C212	0.2014 (17)	−0.107 (4)	0.4299 (12)	0.039 (4)	0.182 (14)
H212	0.232664	−0.099298	0.424470	0.046*	0.182 (14)
N34B	0.2009 (15)	−0.125 (4)	0.4718 (12)	0.042 (5)	0.182 (14)
C213	0.1576 (18)	−0.096 (4)	0.4855 (18)	0.051 (8)	0.182 (14)
H21G	0.133827	−0.068207	0.459782	0.077*	0.182 (14)
H21H	0.168063	−0.054626	0.510613	0.077*	0.182 (14)
H21I	0.142108	−0.146668	0.495384	0.077*	0.182 (14)
C214	0.2459 (17)	−0.131 (5)	0.5090 (15)	0.054 (8)	0.182 (14)
H21J	0.273231	−0.149543	0.497355	0.082*	0.182 (14)
H21K	0.241269	−0.173303	0.531238	0.082*	0.182 (14)
H21L	0.253636	−0.073818	0.523575	0.082*	0.182 (14)
O75	0.0977 (2)	−0.1017 (5)	0.2795 (3)	0.0386 (16)	
C179	0.1086 (5)	−0.1750 (9)	0.2712 (4)	0.068 (3)	
H179	0.103502	−0.219082	0.291042	0.081*	
N35	0.1261 (5)	−0.2011 (9)	0.2390 (4)	0.084 (4)	
C180	0.1355 (8)	−0.1346 (11)	0.2071 (6)	0.101 (6)	
H18A	0.115744	−0.148409	0.176160	0.151*	
H18B	0.170656	−0.134651	0.208644	0.151*	
H18C	0.126105	−0.076977	0.215626	0.151*	
C181	0.1443 (6)	−0.2846 (10)	0.2321 (6)	0.084 (5)	
H18D	0.124416	−0.308097	0.203036	0.126*	
H18E	0.142044	−0.323534	0.256721	0.126*	
H18F	0.178860	−0.279683	0.231906	0.126*	
O76	1.0387 (13)	−0.294 (2)	0.5350 (12)	0.201 (12)	0.700 (13)
C182	1.0500 (11)	−0.235 (2)	0.5117 (12)	0.135 (8)	0.700 (13)
H182	1.080552	−0.236175	0.504323	0.162*	0.700 (13)
N36	1.0180 (8)	−0.1733 (14)	0.4981 (8)	0.106 (6)	0.700 (13)
C183	0.9655 (9)	−0.184 (2)	0.4902 (12)	0.130 (10)	0.700 (13)
H18G	0.951148	−0.208998	0.459946	0.195*	0.700 (13)
H18H	0.950290	−0.127710	0.492257	0.195*	0.700 (13)
H18I	0.959435	−0.223714	0.513127	0.195*	0.700 (13)
C184	1.0241 (9)	−0.0940 (15)	0.4748 (8)	0.091 (7)	0.700 (13)
H18J	1.059380	−0.080275	0.481532	0.136*	0.700 (13)
H18K	1.006984	−0.046176	0.485156	0.136*	0.700 (13)
H18L	1.010036	−0.101876	0.442038	0.136*	0.700 (13)
O76B	1.2105 (13)	−0.511 (3)	0.4752 (16)	0.20 (2)	0.300 (13)
C215	1.1997 (9)	−0.453 (3)	0.4989 (15)	0.174 (19)	0.300 (13)
H215	1.227430	−0.427108	0.519928	0.209*	0.300 (13)
N36B	1.1583 (9)	−0.4220 (18)	0.5001 (10)	0.166 (18)	0.300 (13)
C216	1.1127 (9)	−0.456 (3)	0.4658 (16)	0.113 (18)	0.300 (13)
H21P	1.083337	−0.427247	0.470233	0.169*	0.300 (13)
H21Q	1.114868	−0.444060	0.435166	0.169*	0.300 (13)
H21R	1.110103	−0.518965	0.469855	0.169*	0.300 (13)
C217	1.1475 (16)	−0.346 (2)	0.5215 (17)	0.16 (2)	0.300 (13)
H21M	1.111560	−0.341245	0.515738	0.237*	0.300 (13)
H21N	1.163203	−0.350310	0.554215	0.237*	0.300 (13)
H21O	1.160067	−0.295295	0.509278	0.237*	0.300 (13)
O77	0.6391 (6)	0.5142 (11)	0.0515 (6)	0.140 (6)	
C185	0.6734 (5)	0.4904 (10)	0.0825 (6)	0.077 (4)	
H185	0.666899	0.489921	0.111214	0.092*	
N37	0.7187 (4)	0.4647 (7)	0.0833 (4)	0.063 (3)	
C186	0.7283 (9)	0.4622 (17)	0.0394 (6)	0.126 (7)	
H18M	0.762290	0.442662	0.043163	0.189*	
H18N	0.705180	0.421637	0.019485	0.189*	
H18O	0.723788	0.520440	0.025936	0.189*	
C187	0.7526 (7)	0.4342 (10)	0.1240 (6)	0.101 (6)	
H18P	0.781981	0.471577	0.131812	0.152*	
H18Q	0.762389	0.374359	0.119568	0.152*	
H18R	0.736896	0.435519	0.148626	0.152*	
O78	0.5851 (5)	0.1301 (7)	0.5505 (3)	0.063 (3)	0.806 (9)
C188	0.5856 (5)	0.0659 (7)	0.5267 (3)	0.034 (3)	0.806 (9)
H188	0.578070	0.075051	0.494884	0.041*	0.806 (9)
N38	0.5956 (3)	−0.0141 (5)	0.5414 (3)	0.0262 (19)	0.806 (9)
C189	0.6073 (5)	−0.0342 (9)	0.5894 (3)	0.040 (3)	0.806 (9)
H18S	0.580249	−0.014610	0.601144	0.060*	0.806 (9)
H18T	0.637953	−0.004430	0.605651	0.060*	0.806 (9)
H18U	0.611676	−0.097190	0.593745	0.060*	0.806 (9)
C190	0.5978 (5)	−0.0841 (7)	0.5108 (3)	0.032 (3)	0.806 (9)
H19A	0.576936	−0.132321	0.515304	0.048*	0.806 (9)
H19B	0.632065	−0.104217	0.516857	0.048*	0.806 (9)
H19C	0.585918	−0.063325	0.479448	0.048*	0.806 (9)
O79	0.5525 (8)	−0.4533 (12)	0.6275 (6)	0.157 (7)	
C191	0.5341 (7)	−0.4795 (14)	0.5877 (7)	0.112 (5)	
H191	0.555218	−0.510224	0.573810	0.134*	
N39	0.4879 (6)	−0.4679 (9)	0.5637 (5)	0.090 (4)	
C192	0.4545 (8)	−0.4245 (14)	0.5853 (7)	0.119 (7)	
H19D	0.421514	−0.420238	0.563713	0.178*	
H19E	0.452551	−0.458208	0.611848	0.178*	
H19F	0.466990	−0.366045	0.594956	0.178*	
C193	0.4667 (9)	−0.4880 (14)	0.5159 (6)	0.124 (7)	
H19G	0.431470	−0.472791	0.506687	0.186*	
H19H	0.483827	−0.454382	0.497791	0.186*	
H19I	0.470454	−0.550261	0.510881	0.186*	
O80	0.3291 (9)	0.5272 (12)	0.0253 (5)	0.109 (7)	0.682 (16)
C194	0.3385 (9)	0.5561 (13)	0.0638 (7)	0.091 (6)	0.682 (16)
H194	0.370966	0.546251	0.083052	0.110*	0.682 (16)
N40	0.3079 (8)	0.5999 (12)	0.0811 (6)	0.086 (5)	0.682 (16)
C195	0.3244 (10)	0.6260 (17)	0.1294 (6)	0.092 (7)	0.682 (16)
H19J	0.297705	0.657727	0.137105	0.138*	0.682 (16)
H19K	0.332791	0.573972	0.148496	0.138*	0.682 (16)
H19L	0.353566	0.663532	0.134610	0.138*	0.682 (16)
C196	0.2647 (9)	0.6413 (17)	0.0500 (7)	0.090 (7)	0.682 (16)
H19M	0.245442	0.671262	0.067465	0.134*	0.682 (16)
H19N	0.275659	0.683544	0.030964	0.134*	0.682 (16)
H19O	0.244088	0.596890	0.030689	0.134*	0.682 (16)
O81	0.1374 (13)	0.208 (2)	0.1117 (9)	0.225 (13)	0.847 (14)
C197	0.1756 (13)	0.1624 (18)	0.1278 (11)	0.182 (11)	0.847 (14)
H197	0.207261	0.188896	0.133915	0.219*	0.847 (14)
N41	0.1726 (11)	0.0799 (16)	0.1362 (7)	0.155 (9)	0.847 (14)
C198	0.1300 (11)	0.031 (2)	0.1103 (12)	0.179 (13)	0.847 (14)
H19P	0.133162	−0.029875	0.120551	0.268*	0.847 (14)
H19Q	0.099831	0.055966	0.114983	0.268*	0.847 (14)
H19R	0.128120	0.033168	0.078037	0.268*	0.847 (14)
C199	0.2104 (12)	0.031 (2)	0.1682 (9)	0.181 (13)	0.847 (14)
H19S	0.199695	−0.029838	0.168498	0.271*	0.847 (14)
H19T	0.241171	0.032814	0.159295	0.271*	0.847 (14)
H19U	0.216141	0.055894	0.198544	0.271*	0.847 (14)
O82	0.8428 (8)	0.4472 (11)	0.5717 (6)	0.088 (5)	0.661 (12)
C200	0.8588 (9)	0.3926 (15)	0.5494 (9)	0.084 (5)	0.661 (12)
H200	0.893467	0.395547	0.553582	0.101*	0.661 (12)
N42	0.8367 (8)	0.3334 (15)	0.5219 (9)	0.079 (4)	0.661 (12)
C201	0.8606 (11)	0.283 (2)	0.4926 (10)	0.079 (6)	0.661 (12)
H20D	0.836826	0.241244	0.474432	0.119*	0.661 (12)
H20E	0.871491	0.322763	0.472475	0.119*	0.661 (12)
H20F	0.889266	0.251481	0.511682	0.119*	0.661 (12)
C202	0.7884 (9)	0.3038 (19)	0.5191 (11)	0.095 (7)	0.661 (12)
H20A	0.778864	0.258408	0.495868	0.143*	0.661 (12)
H20B	0.787680	0.280027	0.548499	0.143*	0.661 (12)
H20C	0.765099	0.352541	0.510976	0.143*	0.661 (12)
O82B	0.8647 (14)	0.428 (2)	0.5651 (14)	0.092 (7)	0.339 (12)
C221	0.8293 (14)	0.378 (3)	0.5480 (14)	0.076 (5)	0.339 (12)
H221	0.801948	0.385269	0.559898	0.092*	0.339 (12)
N42B	0.8228 (13)	0.318 (3)	0.5179 (17)	0.080 (5)	0.339 (12)
C222	0.7731 (14)	0.276 (3)	0.5004 (17)	0.082 (8)	0.339 (12)
H22D	0.774427	0.232071	0.477555	0.123*	0.339 (12)
H22E	0.764070	0.247655	0.525533	0.123*	0.339 (12)
H22F	0.748208	0.319886	0.486597	0.123*	0.339 (12)
C223	0.8590 (16)	0.276 (3)	0.5020 (15)	0.074 (9)	0.339 (12)
H22G	0.843017	0.233464	0.478789	0.111*	0.339 (12)
H22H	0.876654	0.319112	0.488949	0.111*	0.339 (12)
H22I	0.882434	0.246343	0.527347	0.111*	0.339 (12)

### Poly[[di-µ4-acetato-nonakis(µ-N,N-dimethylformamide)octakis(N,N-dimethylformamide)tetrakis[salicylhydroximato(2-)]tetradecakis[salicylhydroximato(3-)]dodecaaluminium(III)didysprosium(III)decasodium(I)] N,N-dimethylformamide 6.335-solvate] (2) Atomic displacement parameters (Å^2^)

**Table:**

	*U*^11^	*U*^22^	*U*^33^	*U*^12^	*U*^13^	*U*^23^
Dy1	0.01136 (19)	0.00735 (19)	0.00883 (18)	0.00085 (16)	0.00334 (14)	0.00051 (15)
Dy2	0.00990 (19)	0.00604 (19)	0.01269 (19)	0.00072 (16)	0.00355 (14)	0.00120 (15)
Al1	0.0111 (10)	0.0079 (10)	0.0207 (11)	−0.0012 (8)	0.0061 (8)	0.0009 (8)
Al2	0.0110 (10)	0.0078 (9)	0.0117 (9)	0.0005 (8)	0.0033 (8)	0.0007 (7)
Al3	0.0193 (12)	0.0289 (12)	0.0068 (10)	0.0038 (10)	0.0037 (8)	−0.0013 (9)
Al4	0.0193 (12)	0.0141 (10)	0.0110 (10)	−0.0047 (8)	−0.0003 (9)	0.0003 (8)
Al5	0.0195 (11)	0.0083 (10)	0.0157 (10)	0.0002 (8)	0.0046 (8)	−0.0014 (8)
Al6	0.0142 (11)	0.0163 (11)	0.0138 (10)	0.0044 (8)	0.0065 (8)	0.0032 (8)
Al7	0.0192 (11)	0.0060 (9)	0.0201 (11)	0.0003 (8)	0.0052 (9)	0.0016 (8)
Al8	0.0157 (11)	0.0136 (11)	0.0114 (10)	−0.0024 (8)	0.0016 (8)	0.0026 (7)
Al9	0.0133 (10)	0.0086 (9)	0.0163 (10)	0.0038 (8)	0.0047 (8)	0.0022 (7)
Al10	0.0159 (11)	0.0205 (11)	0.0133 (10)	0.0055 (9)	0.0017 (9)	−0.0001 (9)
Al11	0.0095 (10)	0.0094 (9)	0.0165 (10)	−0.0011 (8)	0.0033 (8)	0.0007 (8)
Al12	0.0110 (10)	0.0077 (9)	0.0179 (10)	0.0007 (8)	0.0048 (8)	0.0027 (8)
Na1	0.0155 (16)	0.0318 (18)	0.0405 (19)	0.0064 (14)	0.0123 (14)	0.0064 (15)
Na2	0.0230 (17)	0.0087 (14)	0.0348 (18)	0.0024 (11)	0.0115 (14)	0.0041 (11)
Na3	0.0370 (19)	0.0073 (14)	0.0350 (18)	0.0014 (13)	0.0095 (15)	−0.0033 (12)
Na4	0.0187 (15)	0.0178 (14)	0.0211 (15)	−0.0043 (12)	0.0070 (12)	−0.0050 (11)
Na5	0.0198 (15)	0.0150 (14)	0.0253 (15)	−0.0052 (12)	0.0104 (12)	−0.0048 (12)
Na6	0.0183 (15)	0.0188 (15)	0.0227 (15)	−0.0007 (12)	0.0051 (12)	−0.0038 (12)
Na7	0.0285 (17)	0.0247 (16)	0.0228 (16)	−0.0070 (14)	0.0081 (13)	0.0000 (12)
Na8	0.0314 (17)	0.0079 (13)	0.0313 (17)	0.0023 (13)	0.0076 (13)	−0.0019 (12)
Na9	0.0235 (16)	0.0077 (13)	0.0235 (15)	0.0017 (11)	0.0082 (12)	0.0012 (11)
Na10	0.0163 (16)	0.041 (2)	0.0325 (18)	0.0061 (14)	0.0112 (14)	0.0082 (15)
O1	0.016 (3)	0.005 (2)	0.019 (3)	−0.001 (2)	0.008 (2)	0.0042 (19)
O2	0.020 (3)	0.018 (3)	0.021 (3)	0.004 (2)	0.016 (2)	0.007 (2)
O3	0.019 (3)	0.010 (2)	0.037 (3)	0.004 (2)	0.014 (2)	0.004 (2)
O4	0.015 (3)	0.014 (2)	0.015 (2)	0.003 (2)	0.009 (2)	−0.0018 (19)
O5	0.016 (3)	0.007 (2)	0.027 (3)	−0.004 (2)	0.010 (2)	0.000 (2)
O6	0.011 (2)	0.012 (2)	0.022 (3)	−0.002 (2)	0.006 (2)	−0.002 (2)
O7	0.008 (2)	0.007 (2)	0.014 (2)	−0.0009 (18)	0.0005 (19)	−0.0021 (18)
O8	0.015 (3)	0.009 (2)	0.030 (3)	−0.002 (2)	0.006 (2)	0.003 (2)
O9	0.016 (3)	0.028 (3)	0.017 (3)	−0.005 (2)	−0.001 (2)	0.004 (2)
O10	0.012 (2)	0.017 (3)	0.006 (2)	0.004 (2)	−0.0005 (18)	0.0049 (19)
O11	0.018 (3)	0.017 (3)	0.015 (2)	0.005 (2)	0.004 (2)	0.002 (2)
O12	0.031 (3)	0.039 (3)	0.010 (3)	0.009 (3)	0.005 (2)	0.004 (2)
O13	0.014 (2)	0.014 (2)	0.005 (2)	0.0031 (19)	0.0001 (18)	−0.0012 (17)
O14	0.015 (3)	0.022 (3)	0.011 (2)	−0.007 (2)	0.001 (2)	−0.0006 (19)
O15	0.017 (3)	0.027 (3)	0.011 (2)	−0.009 (2)	0.003 (2)	0.000 (2)
O16	0.017 (3)	0.014 (2)	0.010 (2)	0.002 (2)	0.0034 (19)	−0.0008 (19)
O17	0.025 (3)	0.011 (2)	0.016 (3)	−0.002 (2)	0.003 (2)	−0.0041 (19)
O18	0.023 (3)	0.026 (3)	0.015 (3)	0.007 (2)	0.002 (2)	−0.003 (2)
O19	0.018 (3)	0.019 (3)	0.010 (2)	0.009 (2)	0.006 (2)	−0.0001 (19)
O20	0.023 (3)	0.046 (4)	0.014 (3)	0.008 (3)	0.004 (2)	−0.004 (2)
O21	0.023 (3)	0.028 (3)	0.017 (3)	0.006 (2)	0.007 (2)	−0.001 (2)
O22	0.017 (3)	0.004 (2)	0.012 (2)	−0.0020 (19)	0.003 (2)	−0.0005 (17)
O23	0.034 (3)	0.019 (3)	0.014 (3)	−0.009 (2)	0.000 (2)	−0.002 (2)
O24	0.023 (3)	0.009 (2)	0.020 (3)	−0.001 (2)	0.004 (2)	−0.0018 (19)
O25	0.010 (2)	0.009 (2)	0.017 (2)	0.0038 (19)	0.0043 (19)	−0.0009 (19)
O26	0.023 (3)	0.009 (2)	0.025 (3)	0.002 (2)	0.008 (2)	0.006 (2)
O27	0.020 (3)	0.021 (3)	0.020 (3)	0.005 (2)	0.007 (2)	0.007 (2)
O28	0.020 (3)	0.004 (2)	0.018 (3)	0.002 (2)	0.005 (2)	0.0033 (18)
O29	0.029 (3)	0.019 (3)	0.011 (3)	−0.001 (2)	0.001 (2)	0.007 (2)
O30	0.031 (3)	0.010 (2)	0.028 (3)	−0.002 (2)	0.012 (2)	0.001 (2)
O31	0.018 (3)	0.014 (3)	0.020 (3)	0.001 (2)	0.005 (2)	0.000 (2)
O32	0.022 (3)	0.008 (2)	0.027 (3)	0.003 (2)	0.001 (2)	−0.002 (2)
O33	0.021 (3)	0.022 (3)	0.020 (3)	0.004 (2)	0.001 (2)	−0.002 (2)
O34	0.019 (3)	0.010 (2)	0.013 (2)	−0.002 (2)	0.006 (2)	0.0024 (18)
O35	0.022 (3)	0.007 (2)	0.023 (3)	0.002 (2)	0.007 (2)	0.002 (2)
O36	0.017 (3)	0.014 (3)	0.022 (3)	0.006 (2)	0.008 (2)	0.003 (2)
O37	0.015 (2)	0.008 (2)	0.007 (2)	−0.0039 (19)	−0.0026 (18)	0.0003 (17)
O38	0.014 (3)	0.012 (2)	0.021 (3)	−0.002 (2)	0.004 (2)	0.003 (2)
O39	0.026 (3)	0.032 (3)	0.018 (3)	−0.007 (3)	−0.001 (2)	0.007 (2)
O40	0.017 (3)	0.010 (2)	0.010 (2)	0.000 (2)	0.0080 (19)	−0.0013 (18)
O41	0.018 (3)	0.029 (3)	0.021 (3)	0.001 (2)	0.007 (2)	−0.002 (2)
O42	0.026 (3)	0.021 (3)	0.026 (3)	0.005 (2)	0.008 (2)	0.002 (2)
O43	0.014 (3)	0.011 (2)	0.015 (2)	−0.002 (2)	0.006 (2)	0.0033 (19)
O44	0.018 (3)	0.016 (3)	0.026 (3)	0.009 (2)	0.013 (2)	0.005 (2)
O45	0.013 (2)	0.010 (2)	0.031 (3)	0.000 (2)	0.009 (2)	0.005 (2)
O46	0.006 (2)	0.012 (2)	0.010 (2)	0.0043 (19)	−0.0020 (18)	0.0004 (18)
O47	0.015 (3)	0.010 (2)	0.019 (3)	0.001 (2)	0.003 (2)	−0.0020 (19)
O48	0.032 (3)	0.034 (3)	0.016 (3)	0.018 (3)	0.006 (2)	0.006 (2)
O49	0.012 (3)	0.015 (2)	0.018 (3)	0.006 (2)	0.001 (2)	0.004 (2)
O50	0.021 (3)	0.034 (3)	0.012 (3)	0.009 (3)	0.000 (2)	−0.001 (2)
O51	0.021 (3)	0.017 (3)	0.019 (3)	0.006 (2)	0.009 (2)	0.001 (2)
O52	0.011 (2)	0.007 (2)	0.016 (2)	0.0021 (19)	0.0055 (19)	0.0040 (18)
O53	0.014 (3)	0.005 (2)	0.025 (3)	−0.0013 (19)	0.006 (2)	0.0016 (19)
O54	0.013 (3)	0.015 (3)	0.024 (3)	−0.002 (2)	0.006 (2)	−0.002 (2)
O55	0.035 (3)	0.013 (3)	0.028 (3)	−0.003 (2)	0.004 (3)	−0.004 (2)
O56	0.013 (3)	0.020 (3)	0.014 (3)	−0.005 (2)	0.003 (2)	−0.007 (2)
O57	0.027 (3)	0.012 (3)	0.028 (3)	−0.004 (2)	0.007 (2)	−0.004 (2)
O58	0.010 (2)	0.017 (3)	0.027 (3)	−0.003 (2)	0.009 (2)	−0.007 (2)
N1	0.010 (3)	0.010 (3)	0.021 (3)	0.001 (2)	0.006 (2)	0.006 (2)
N2	0.010 (3)	0.007 (3)	0.016 (3)	0.004 (2)	0.008 (2)	0.001 (2)
N3	0.011 (3)	0.011 (3)	0.009 (3)	0.001 (2)	−0.003 (2)	−0.004 (2)
N4	0.016 (3)	0.024 (3)	0.004 (3)	0.002 (3)	0.000 (2)	0.003 (2)
N5	0.018 (3)	0.014 (3)	0.008 (3)	0.003 (2)	0.010 (2)	0.000 (2)
N6	0.018 (3)	0.016 (3)	0.014 (3)	−0.001 (3)	0.000 (2)	−0.001 (2)
N7	0.020 (3)	0.020 (3)	0.011 (3)	0.007 (3)	0.010 (2)	0.001 (2)
N8	0.021 (3)	0.006 (3)	0.012 (3)	−0.001 (2)	0.003 (2)	0.006 (2)
N9	0.011 (3)	0.016 (3)	0.022 (3)	0.007 (2)	0.003 (2)	−0.001 (2)
N10	0.020 (3)	0.011 (3)	0.016 (3)	−0.003 (3)	0.007 (3)	0.003 (2)
N11	0.015 (3)	0.013 (3)	0.020 (3)	−0.002 (2)	0.001 (3)	−0.003 (2)
N12	0.0133 (10)	0.0086 (9)	0.0163 (10)	0.0038 (8)	0.0047 (8)	0.0022 (7)
N13	0.014 (3)	0.014 (3)	0.011 (3)	0.001 (2)	0.001 (2)	0.000 (2)
N14	0.012 (3)	0.025 (3)	0.015 (3)	0.002 (3)	0.005 (2)	−0.005 (2)
N15	0.015 (3)	0.007 (3)	0.016 (3)	0.001 (2)	0.004 (2)	0.004 (2)
N16	0.016 (3)	0.018 (3)	0.008 (3)	−0.002 (3)	0.001 (2)	0.001 (2)
N17	0.017 (3)	0.017 (3)	0.011 (3)	0.005 (3)	0.009 (2)	0.002 (2)
N18	0.011 (3)	0.009 (3)	0.015 (3)	0.004 (2)	0.005 (2)	0.001 (2)
C1	0.014 (4)	0.016 (4)	0.025 (4)	0.007 (3)	0.008 (3)	0.007 (3)
C2	0.018 (4)	0.025 (5)	0.038 (5)	0.009 (3)	0.017 (4)	0.014 (4)
C3	0.014 (4)	0.018 (4)	0.040 (5)	0.004 (3)	0.012 (3)	0.016 (3)
C4	0.030 (5)	0.016 (4)	0.084 (8)	0.007 (4)	0.032 (5)	0.019 (5)
C5	0.041 (6)	0.029 (6)	0.108 (10)	0.012 (5)	0.043 (7)	0.038 (6)
C6	0.043 (6)	0.037 (5)	0.086 (8)	0.012 (4)	0.050 (6)	0.022 (5)
C7	0.027 (5)	0.029 (5)	0.060 (6)	0.005 (4)	0.032 (5)	0.016 (4)
C8	0.012 (4)	0.008 (3)	0.022 (4)	0.001 (3)	0.008 (3)	0.003 (3)
C9	0.019 (4)	0.004 (3)	0.019 (4)	0.001 (3)	0.008 (3)	−0.001 (3)
C10	0.017 (4)	0.008 (3)	0.018 (3)	0.005 (3)	0.009 (3)	0.001 (3)
C11	0.023 (4)	0.004 (3)	0.029 (4)	0.001 (3)	0.011 (3)	−0.005 (3)
C12	0.024 (4)	0.016 (4)	0.042 (5)	0.000 (3)	0.021 (4)	−0.008 (3)
C13	0.029 (5)	0.017 (4)	0.046 (5)	−0.002 (3)	0.020 (4)	−0.014 (4)
C14	0.022 (4)	0.009 (3)	0.035 (4)	−0.008 (3)	0.014 (3)	−0.008 (3)
C15	0.010 (3)	0.017 (4)	0.024 (4)	−0.001 (3)	0.008 (3)	−0.003 (3)
C16	0.008 (3)	0.022 (4)	0.021 (4)	0.000 (3)	−0.003 (3)	−0.006 (3)
C17	0.013 (4)	0.033 (4)	0.016 (4)	−0.005 (3)	0.001 (3)	−0.001 (3)
C18	0.021 (4)	0.049 (6)	0.022 (4)	−0.007 (4)	−0.003 (3)	−0.002 (4)
C19	0.018 (4)	0.058 (6)	0.031 (5)	−0.009 (4)	−0.002 (4)	−0.007 (4)
C20	0.016 (4)	0.037 (5)	0.046 (6)	−0.009 (4)	−0.002 (4)	−0.016 (4)
C21	0.016 (4)	0.022 (4)	0.037 (5)	−0.002 (3)	0.000 (3)	−0.007 (3)
C22	0.018 (4)	0.018 (4)	0.011 (3)	−0.003 (3)	0.000 (3)	−0.001 (3)
C23	0.020 (4)	0.023 (4)	0.014 (4)	0.000 (3)	0.002 (3)	0.006 (3)
C24	0.028 (4)	0.035 (5)	0.007 (3)	0.000 (4)	−0.001 (3)	0.005 (3)
C25	0.051 (6)	0.040 (5)	0.010 (4)	0.001 (4)	0.006 (4)	0.007 (3)
C26	0.054 (7)	0.045 (6)	0.021 (5)	0.003 (5)	0.003 (4)	0.017 (4)
C27	0.044 (6)	0.030 (5)	0.024 (4)	0.007 (4)	−0.002 (4)	0.009 (4)
C28	0.035 (5)	0.036 (5)	0.021 (4)	0.006 (4)	0.006 (4)	0.009 (4)
C29	0.015 (4)	0.011 (3)	0.018 (4)	−0.003 (3)	0.009 (3)	−0.001 (3)
C30	0.020 (4)	0.019 (4)	0.014 (4)	−0.004 (3)	0.009 (3)	0.002 (3)
C31	0.017 (4)	0.024 (4)	0.013 (3)	−0.010 (3)	0.006 (3)	−0.003 (3)
C32	0.023 (4)	0.054 (6)	0.011 (4)	−0.013 (4)	0.004 (3)	0.000 (3)
C33	0.031 (5)	0.078 (8)	0.011 (4)	−0.019 (5)	0.005 (3)	0.003 (4)
C34	0.032 (4)	0.071 (7)	0.013 (4)	−0.022 (5)	0.015 (3)	−0.003 (4)
C35	0.019 (4)	0.043 (5)	0.016 (4)	−0.009 (4)	0.007 (3)	0.000 (3)
C36	0.018 (4)	0.010 (3)	0.021 (4)	−0.003 (3)	0.005 (3)	−0.006 (3)
C37	0.022 (4)	0.025 (4)	0.026 (4)	0.002 (3)	0.002 (3)	−0.011 (3)
C38	0.024 (4)	0.026 (4)	0.017 (4)	0.008 (3)	−0.003 (3)	−0.010 (3)
C39	0.047 (6)	0.043 (6)	0.013 (4)	0.014 (5)	0.002 (4)	−0.007 (4)
C40	0.064 (8)	0.053 (7)	0.027 (5)	0.014 (6)	0.002 (5)	−0.027 (5)
C41	0.067 (8)	0.033 (5)	0.034 (5)	0.014 (5)	−0.002 (5)	−0.023 (4)
C42	0.049 (6)	0.025 (5)	0.032 (5)	0.005 (4)	0.002 (4)	−0.016 (4)
C43	0.028 (4)	0.024 (4)	0.013 (4)	0.006 (3)	0.009 (3)	0.005 (3)
C44	0.032 (4)	0.028 (4)	0.015 (4)	0.007 (4)	0.016 (3)	0.001 (3)
C45	0.032 (4)	0.024 (4)	0.021 (4)	0.010 (4)	0.014 (3)	0.002 (3)
C46	0.032 (5)	0.031 (5)	0.032 (4)	0.010 (4)	0.019 (4)	0.005 (4)
C47	0.041 (5)	0.055 (6)	0.032 (4)	0.024 (5)	0.025 (4)	0.002 (4)
C48	0.055 (6)	0.054 (6)	0.019 (4)	0.018 (5)	0.019 (4)	−0.011 (4)
C49	0.039 (5)	0.047 (5)	0.018 (4)	0.015 (4)	0.012 (3)	−0.006 (4)
C50	0.025 (4)	0.014 (3)	0.016 (3)	−0.004 (3)	0.005 (3)	−0.001 (3)
C51	0.029 (5)	0.014 (4)	0.021 (5)	−0.004 (4)	0.007 (4)	0.005 (4)
C52	0.026 (6)	0.011 (5)	0.022 (5)	−0.003 (4)	0.007 (5)	0.000 (4)
C53	0.028 (8)	0.016 (5)	0.029 (5)	−0.004 (5)	0.009 (6)	0.005 (5)
C54	0.035 (8)	0.019 (5)	0.030 (5)	−0.001 (6)	0.011 (7)	0.013 (5)
C55	0.038 (8)	0.024 (6)	0.028 (5)	−0.005 (6)	0.008 (7)	0.014 (5)
C56	0.032 (8)	0.020 (5)	0.021 (5)	−0.005 (6)	0.004 (6)	0.008 (5)
C50B	0.025 (4)	0.014 (3)	0.016 (3)	−0.004 (3)	0.005 (3)	−0.001 (3)
C51B	0.029 (5)	0.015 (5)	0.021 (5)	−0.005 (5)	0.005 (5)	0.004 (5)
C52B	0.028 (6)	0.016 (5)	0.022 (5)	−0.003 (5)	0.004 (5)	0.005 (5)
C53B	0.032 (8)	0.017 (6)	0.028 (6)	−0.008 (6)	0.010 (6)	0.005 (5)
C54B	0.036 (9)	0.019 (6)	0.030 (6)	−0.006 (7)	0.009 (7)	0.015 (5)
C55B	0.032 (8)	0.023 (6)	0.026 (6)	−0.005 (7)	0.007 (7)	0.016 (5)
C56B	0.031 (8)	0.022 (6)	0.019 (6)	−0.004 (6)	0.006 (6)	0.006 (5)
C57	0.026 (4)	0.008 (3)	0.023 (4)	0.002 (3)	0.005 (3)	−0.001 (3)
C58	0.025 (5)	0.015 (4)	0.041 (5)	0.008 (3)	0.011 (4)	0.009 (3)
C59	0.024 (4)	0.023 (4)	0.021 (4)	0.006 (3)	0.012 (3)	0.004 (3)
C60	0.021 (4)	0.043 (6)	0.040 (5)	0.013 (4)	0.017 (4)	0.015 (4)
C61	0.021 (5)	0.056 (7)	0.082 (9)	0.022 (5)	0.015 (5)	0.032 (6)
C62	0.034 (6)	0.039 (6)	0.126 (13)	0.019 (5)	0.024 (7)	0.048 (7)
C63	0.029 (6)	0.026 (5)	0.093 (9)	0.009 (4)	0.017 (6)	0.024 (6)
C64	0.026 (4)	0.016 (4)	0.023 (4)	0.001 (3)	0.013 (3)	0.006 (3)
C65	0.045 (5)	0.019 (4)	0.021 (4)	0.000 (4)	0.014 (4)	0.006 (3)
C66	0.037 (5)	0.017 (4)	0.033 (5)	0.001 (4)	0.020 (4)	0.011 (3)
C67	0.048 (6)	0.020 (4)	0.041 (5)	0.000 (4)	0.015 (5)	0.005 (4)
C68	0.072 (8)	0.023 (5)	0.056 (7)	0.003 (5)	0.017 (6)	0.028 (5)
C69	0.094 (10)	0.038 (6)	0.030 (5)	0.006 (6)	0.016 (6)	0.017 (4)
C70	0.066 (8)	0.031 (5)	0.033 (5)	0.006 (5)	0.013 (5)	0.008 (4)
C71	0.019 (4)	0.010 (4)	0.027 (4)	−0.005 (3)	0.005 (3)	0.000 (3)
C72	0.022 (4)	0.016 (4)	0.029 (4)	−0.004 (3)	0.003 (3)	−0.005 (3)
C73	0.022 (4)	0.024 (4)	0.032 (5)	−0.005 (4)	0.000 (4)	−0.006 (4)
C74	0.055 (6)	0.026 (5)	0.024 (4)	0.001 (4)	0.004 (4)	−0.005 (4)
C75	0.077 (9)	0.042 (6)	0.029 (5)	−0.002 (6)	0.005 (5)	−0.019 (4)
C76	0.071 (8)	0.029 (5)	0.046 (6)	0.009 (5)	0.004 (6)	−0.017 (5)
C77	0.036 (5)	0.022 (5)	0.043 (6)	−0.001 (4)	0.002 (4)	−0.007 (4)
C78	0.024 (4)	0.007 (3)	0.018 (4)	0.000 (3)	0.008 (3)	−0.003 (3)
C79	0.024 (4)	0.016 (4)	0.029 (4)	0.007 (3)	0.010 (3)	0.004 (3)
C80	0.025 (4)	0.020 (4)	0.016 (4)	0.004 (3)	0.009 (3)	0.000 (3)
C81	0.021 (4)	0.033 (5)	0.033 (5)	0.009 (4)	0.010 (3)	0.007 (4)
C82	0.022 (5)	0.049 (6)	0.071 (8)	0.024 (4)	0.014 (5)	0.022 (5)
C83	0.042 (6)	0.028 (5)	0.102 (10)	0.028 (4)	0.019 (6)	0.024 (6)
C84	0.036 (5)	0.025 (5)	0.072 (8)	0.018 (4)	0.017 (5)	0.014 (5)
C85	0.012 (4)	0.014 (4)	0.021 (4)	0.000 (3)	0.005 (3)	−0.004 (3)
C86	0.016 (4)	0.019 (4)	0.016 (4)	−0.001 (3)	0.002 (3)	−0.002 (3)
C87	0.018 (4)	0.035 (5)	0.016 (4)	−0.005 (4)	0.004 (3)	−0.003 (3)
C88	0.025 (5)	0.044 (5)	0.018 (4)	−0.003 (4)	0.000 (3)	0.005 (4)
C89	0.019 (4)	0.052 (6)	0.025 (4)	−0.007 (4)	−0.002 (3)	0.002 (4)
C90	0.019 (4)	0.039 (5)	0.035 (5)	−0.013 (4)	0.002 (4)	−0.007 (4)
C91	0.018 (4)	0.027 (4)	0.026 (4)	−0.005 (3)	0.004 (3)	0.000 (3)
C92	0.022 (4)	0.013 (3)	0.023 (4)	0.000 (3)	0.005 (3)	0.003 (3)
C93	0.025 (4)	0.015 (4)	0.024 (4)	0.003 (3)	0.008 (3)	0.002 (3)
C94	0.025 (4)	0.024 (4)	0.020 (4)	0.002 (3)	0.005 (3)	0.001 (3)
C95	0.044 (6)	0.053 (6)	0.022 (4)	0.015 (5)	0.005 (4)	−0.008 (4)
C96	0.068 (8)	0.054 (7)	0.031 (5)	0.024 (6)	−0.004 (5)	−0.023 (5)
C97	0.064 (7)	0.054 (7)	0.029 (5)	0.025 (6)	0.006 (5)	−0.018 (5)
C98	0.036 (5)	0.033 (5)	0.024 (4)	0.012 (4)	0.008 (4)	−0.002 (3)
C99	0.007 (3)	0.013 (3)	0.022 (4)	0.003 (3)	0.004 (3)	0.009 (3)
C100	0.022 (4)	0.016 (4)	0.036 (5)	0.001 (3)	0.016 (4)	0.009 (3)
C101	0.020 (4)	0.018 (4)	0.044 (5)	0.003 (3)	0.011 (4)	0.010 (4)
C102	0.042 (6)	0.024 (5)	0.078 (8)	0.003 (4)	0.042 (6)	0.017 (5)
C103	0.064 (8)	0.029 (6)	0.121 (12)	0.008 (5)	0.062 (9)	0.027 (6)
C104	0.065 (8)	0.034 (6)	0.106 (11)	0.011 (5)	0.070 (8)	0.031 (6)
C105	0.035 (5)	0.023 (5)	0.063 (7)	0.007 (4)	0.031 (5)	0.016 (4)
C106	0.009 (3)	0.014 (4)	0.023 (4)	−0.003 (3)	0.000 (3)	0.000 (3)
C107	0.017 (4)	0.018 (4)	0.021 (4)	−0.003 (3)	0.003 (3)	0.001 (3)
C108	0.025 (4)	0.022 (4)	0.020 (4)	0.001 (3)	0.004 (3)	0.004 (3)
C109	0.038 (5)	0.027 (4)	0.020 (4)	0.007 (4)	0.009 (4)	0.005 (3)
C110	0.034 (5)	0.028 (4)	0.022 (4)	0.008 (4)	0.004 (4)	0.011 (3)
C111	0.039 (5)	0.020 (4)	0.026 (4)	0.004 (4)	0.004 (4)	0.004 (3)
C112	0.025 (4)	0.014 (4)	0.026 (4)	0.000 (3)	0.001 (3)	0.003 (3)
C113	0.019 (4)	0.018 (4)	0.015 (4)	0.003 (3)	0.004 (3)	0.003 (3)
C114	0.021 (4)	0.021 (4)	0.017 (4)	0.006 (3)	0.006 (3)	0.004 (3)
C115	0.019 (4)	0.018 (4)	0.020 (4)	0.004 (3)	0.012 (3)	0.000 (3)
C116	0.027 (4)	0.034 (5)	0.022 (4)	0.011 (4)	0.014 (3)	−0.002 (3)
C117	0.031 (5)	0.046 (5)	0.025 (4)	0.006 (4)	0.019 (4)	−0.008 (4)
C118	0.030 (5)	0.050 (6)	0.024 (4)	0.000 (4)	0.017 (4)	−0.004 (4)
C119	0.034 (5)	0.036 (5)	0.015 (4)	0.000 (4)	0.015 (4)	−0.001 (3)
C120	0.012 (3)	0.008 (3)	0.017 (3)	0.004 (3)	0.004 (3)	0.005 (3)
C121	0.020 (4)	0.006 (3)	0.019 (4)	0.002 (3)	0.007 (3)	0.000 (3)
C122	0.015 (4)	0.007 (3)	0.017 (3)	0.005 (3)	0.006 (3)	0.006 (3)
C123	0.017 (4)	0.013 (4)	0.036 (4)	0.003 (3)	0.013 (3)	0.001 (3)
C124	0.032 (5)	0.015 (4)	0.038 (5)	0.002 (3)	0.016 (4)	−0.007 (3)
C125	0.028 (5)	0.013 (4)	0.039 (5)	−0.003 (3)	0.014 (4)	−0.015 (3)
C126	0.022 (4)	0.010 (3)	0.028 (4)	0.000 (3)	0.009 (3)	−0.001 (3)
C127	0.015 (4)	0.022 (4)	0.015 (4)	−0.005 (3)	0.008 (3)	−0.006 (3)
C128	0.055 (7)	0.049 (6)	0.014 (4)	−0.018 (5)	−0.010 (4)	0.001 (4)
C129	0.018 (4)	0.011 (4)	0.033 (5)	−0.001 (3)	0.013 (3)	−0.001 (3)
C130	0.018 (4)	0.030 (4)	0.027 (4)	−0.007 (3)	0.005 (3)	−0.001 (3)
O59	0.021 (3)	0.040 (4)	0.046 (4)	0.010 (3)	0.016 (3)	0.000 (3)
C131	0.051 (9)	0.118 (16)	0.098 (13)	0.034 (10)	−0.012 (9)	−0.028 (12)
N19	0.106 (11)	0.087 (9)	0.056 (7)	0.063 (9)	0.001 (7)	−0.022 (6)
C132	0.13 (2)	0.13 (2)	0.17 (2)	−0.021 (16)	0.078 (18)	0.044 (18)
C133	0.079 (12)	0.085 (12)	0.135 (18)	0.026 (10)	0.008 (11)	−0.046 (12)
O60	0.027 (3)	0.052 (4)	0.048 (4)	0.008 (3)	0.017 (3)	0.011 (3)
C134	0.037 (5)	0.048 (5)	0.051 (5)	0.008 (4)	0.017 (4)	0.018 (4)
N20	0.055 (6)	0.057 (6)	0.037 (5)	−0.002 (5)	0.021 (4)	0.003 (4)
C135	0.092 (11)	0.105 (11)	0.065 (9)	−0.019 (9)	0.028 (8)	0.007 (9)
C136	0.068 (10)	0.058 (8)	0.067 (10)	0.008 (8)	0.006 (8)	0.023 (8)
O60B	0.027 (3)	0.052 (4)	0.048 (4)	0.008 (3)	0.017 (3)	0.011 (3)
C224	0.042 (7)	0.054 (7)	0.047 (7)	0.004 (7)	0.020 (7)	0.012 (7)
N20B	0.052 (8)	0.056 (8)	0.046 (8)	0.002 (8)	0.020 (7)	0.008 (8)
C225	0.061 (16)	0.063 (16)	0.041 (15)	0.003 (16)	0.028 (14)	−0.003 (15)
C226	0.058 (17)	0.054 (16)	0.051 (17)	0.006 (16)	0.019 (16)	0.007 (16)
O61	0.040 (4)	0.039 (4)	0.031 (3)	−0.002 (3)	0.003 (3)	0.005 (3)
C137	0.041 (5)	0.030 (4)	0.025 (4)	−0.009 (4)	0.005 (4)	0.005 (3)
N21	0.056 (5)	0.035 (4)	0.034 (4)	−0.006 (4)	0.019 (4)	0.006 (3)
C138	0.059 (7)	0.042 (6)	0.068 (8)	−0.003 (5)	0.031 (6)	0.020 (5)
C139	0.074 (8)	0.041 (6)	0.041 (6)	−0.011 (6)	0.012 (6)	0.000 (5)
O62	0.048 (4)	0.021 (3)	0.043 (4)	0.006 (3)	0.023 (3)	0.000 (3)
C140	0.050 (5)	0.018 (4)	0.041 (5)	0.000 (4)	0.015 (4)	0.004 (3)
N22	0.072 (6)	0.039 (5)	0.041 (5)	−0.017 (4)	0.028 (4)	−0.006 (4)
C141	0.127 (13)	0.038 (6)	0.073 (9)	−0.024 (8)	0.054 (9)	−0.002 (6)
C142	0.107 (12)	0.084 (10)	0.040 (7)	−0.021 (9)	0.019 (7)	0.005 (6)
O63	0.025 (3)	0.020 (3)	0.041 (3)	0.004 (2)	0.011 (2)	0.000 (2)
C143	0.030 (5)	0.022 (5)	0.036 (5)	0.002 (4)	0.009 (4)	0.000 (4)
N23	0.028 (4)	0.022 (6)	0.044 (6)	0.004 (4)	0.006 (4)	0.000 (4)
C144	0.024 (6)	0.027 (6)	0.044 (7)	−0.002 (4)	0.002 (5)	0.002 (5)
C145	0.035 (7)	0.030 (7)	0.070 (9)	0.005 (6)	0.015 (6)	−0.008 (6)
O63B	0.025 (3)	0.020 (3)	0.041 (3)	0.004 (2)	0.011 (2)	0.000 (2)
C203	0.028 (6)	0.022 (6)	0.041 (7)	0.005 (6)	0.007 (6)	0.002 (6)
N23B	0.031 (7)	0.028 (8)	0.046 (8)	0.005 (7)	0.005 (7)	0.000 (7)
C204	0.042 (14)	0.036 (14)	0.058 (15)	0.014 (13)	0.013 (13)	−0.006 (13)
C205	0.034 (14)	0.043 (16)	0.060 (16)	0.000 (15)	0.009 (14)	−0.007 (15)
O64	0.040 (6)	0.015 (5)	0.042 (5)	0.002 (5)	0.003 (5)	0.002 (4)
C146	0.043 (6)	0.022 (5)	0.041 (6)	0.007 (5)	0.012 (5)	0.003 (4)
N24	0.049 (6)	0.024 (5)	0.042 (6)	0.009 (5)	0.006 (5)	0.001 (4)
C147	0.038 (10)	0.026 (9)	0.061 (9)	0.014 (7)	−0.001 (8)	−0.004 (7)
C148	0.091 (13)	0.036 (8)	0.045 (9)	0.008 (8)	0.006 (9)	0.010 (7)
O64B	0.041 (8)	0.018 (7)	0.041 (7)	0.005 (7)	0.005 (7)	0.000 (7)
C230	0.050 (7)	0.026 (6)	0.047 (7)	0.005 (6)	0.003 (6)	0.002 (6)
N24B	0.052 (8)	0.035 (7)	0.051 (7)	0.001 (7)	0.002 (7)	0.005 (7)
C231	0.054 (16)	0.041 (14)	0.052 (13)	0.012 (13)	0.007 (13)	−0.010 (13)
C232	0.08 (2)	0.07 (2)	0.11 (2)	−0.02 (2)	−0.02 (2)	0.03 (2)
O65	0.032 (3)	0.025 (3)	0.030 (3)	−0.003 (3)	0.007 (3)	0.004 (2)
C149	0.034 (5)	0.034 (5)	0.041 (5)	−0.004 (4)	0.013 (4)	0.000 (4)
N25	0.044 (5)	0.050 (5)	0.036 (4)	0.002 (4)	0.009 (4)	0.005 (4)
C150	0.072 (9)	0.070 (9)	0.058 (8)	−0.011 (7)	−0.020 (7)	0.007 (7)
C151	0.097 (10)	0.063 (8)	0.056 (7)	0.007 (8)	0.027 (7)	0.028 (6)
O66	0.058 (5)	0.038 (4)	0.065 (5)	0.015 (4)	0.009 (4)	0.011 (4)
C152	0.044 (6)	0.038 (5)	0.085 (7)	0.002 (5)	0.023 (5)	0.008 (5)
N26	0.031 (4)	0.028 (4)	0.076 (6)	0.007 (3)	0.006 (4)	0.011 (4)
C153	0.050 (7)	0.043 (6)	0.074 (8)	0.003 (5)	0.021 (6)	0.009 (5)
C154	0.059 (8)	0.047 (7)	0.086 (10)	0.009 (6)	0.001 (7)	−0.002 (7)
O67	0.075 (5)	0.038 (4)	0.038 (4)	−0.014 (4)	0.028 (4)	−0.015 (3)
C155	0.056 (6)	0.040 (5)	0.033 (5)	−0.008 (4)	0.020 (4)	−0.005 (4)
N27	0.064 (6)	0.048 (5)	0.047 (5)	−0.013 (4)	0.026 (4)	−0.019 (4)
C156	0.100 (11)	0.059 (7)	0.036 (6)	−0.007 (7)	−0.006 (6)	−0.002 (5)
C157	0.076 (9)	0.068 (9)	0.088 (10)	−0.003 (7)	0.026 (8)	−0.043 (8)
O68	0.042 (4)	0.037 (3)	0.050 (4)	−0.003 (3)	0.028 (3)	−0.012 (3)
C158	0.049 (5)	0.036 (5)	0.051 (5)	−0.005 (4)	0.027 (4)	−0.006 (4)
N28	0.042 (5)	0.043 (4)	0.049 (5)	−0.006 (4)	0.022 (4)	−0.008 (4)
C159	0.054 (7)	0.048 (6)	0.041 (6)	0.001 (5)	0.021 (5)	−0.001 (5)
C160	0.081 (9)	0.057 (7)	0.053 (7)	0.001 (7)	0.029 (7)	−0.018 (6)
O69	0.054 (6)	0.036 (4)	0.028 (4)	−0.002 (6)	0.019 (5)	0.001 (3)
C161	0.049 (5)	0.039 (5)	0.023 (4)	−0.006 (5)	0.013 (4)	0.004 (4)
N29	0.032 (4)	0.040 (5)	0.022 (5)	−0.008 (4)	0.012 (4)	0.004 (4)
C162	0.046 (7)	0.060 (8)	0.045 (7)	0.005 (6)	0.022 (6)	0.015 (6)
C163	0.063 (8)	0.048 (7)	0.032 (6)	−0.011 (6)	0.017 (6)	0.002 (5)
O69B	0.048 (10)	0.039 (8)	0.021 (8)	−0.005 (9)	0.016 (8)	0.001 (7)
C206	0.037 (7)	0.036 (7)	0.015 (6)	−0.004 (7)	0.021 (6)	0.003 (6)
N29B	0.039 (7)	0.041 (7)	0.021 (7)	−0.007 (7)	0.015 (7)	0.002 (7)
C207	0.045 (13)	0.047 (13)	0.012 (11)	−0.006 (12)	0.015 (11)	−0.004 (11)
C208	0.031 (12)	0.048 (13)	0.021 (12)	−0.018 (12)	0.014 (11)	0.005 (11)
O70	0.040 (3)	0.014 (2)	0.033 (3)	0.002 (3)	0.000 (3)	0.001 (2)
C164	0.058 (6)	0.037 (5)	0.038 (5)	0.011 (5)	0.009 (5)	−0.005 (5)
N30	0.076 (7)	0.039 (6)	0.045 (6)	0.013 (6)	0.003 (5)	−0.011 (5)
C165	0.079 (11)	0.041 (11)	0.055 (9)	0.005 (9)	0.011 (8)	−0.010 (8)
C166	0.142 (19)	0.082 (14)	0.046 (10)	0.045 (14)	−0.023 (12)	−0.020 (10)
O70B	0.040 (3)	0.014 (2)	0.033 (3)	0.002 (3)	0.000 (3)	0.001 (2)
C209	0.058 (7)	0.029 (6)	0.041 (7)	0.006 (6)	0.004 (6)	−0.003 (6)
N30B	0.058 (8)	0.033 (7)	0.040 (8)	0.007 (7)	0.003 (7)	0.000 (7)
C210	0.082 (15)	0.047 (17)	0.053 (14)	0.013 (16)	0.005 (13)	−0.004 (15)
C211	0.06 (2)	0.06 (2)	0.09 (2)	0.013 (18)	−0.02 (2)	0.003 (19)
O71	0.041 (4)	0.011 (3)	0.033 (3)	0.001 (2)	0.018 (3)	0.006 (2)
C167	0.042 (5)	0.015 (4)	0.032 (4)	−0.001 (3)	0.014 (4)	−0.002 (3)
N31	0.060 (5)	0.023 (4)	0.025 (4)	−0.004 (3)	0.015 (3)	0.002 (3)
C168	0.101 (10)	0.015 (4)	0.045 (6)	−0.016 (5)	0.031 (6)	0.000 (4)
C169	0.086 (9)	0.048 (6)	0.030 (5)	−0.021 (6)	0.018 (5)	0.002 (4)
O72	0.021 (3)	0.010 (3)	0.039 (3)	0.000 (2)	0.009 (2)	−0.002 (2)
C170	0.028 (4)	0.018 (4)	0.039 (4)	0.004 (3)	0.009 (4)	−0.001 (3)
N32	0.026 (4)	0.026 (4)	0.040 (4)	0.005 (3)	0.010 (3)	0.002 (3)
C171	0.028 (5)	0.027 (5)	0.042 (5)	−0.002 (4)	0.008 (4)	−0.010 (4)
C172	0.028 (5)	0.049 (6)	0.065 (7)	0.001 (5)	0.015 (5)	−0.005 (5)
O73	0.040 (4)	0.029 (3)	0.025 (3)	0.003 (3)	0.008 (3)	−0.003 (2)
C173	0.037 (4)	0.022 (4)	0.018 (4)	−0.011 (3)	0.006 (3)	0.005 (3)
N33	0.059 (5)	0.021 (4)	0.032 (4)	−0.008 (3)	0.023 (4)	−0.004 (3)
C174	0.144 (13)	0.041 (6)	0.073 (9)	−0.033 (8)	0.077 (9)	−0.018 (6)
C175	0.066 (8)	0.034 (6)	0.046 (6)	−0.008 (5)	0.015 (5)	−0.009 (5)
O74	0.035 (4)	0.038 (7)	0.026 (4)	0.001 (4)	0.016 (4)	0.005 (4)
C176	0.042 (5)	0.044 (5)	0.036 (5)	0.004 (4)	0.018 (4)	0.006 (4)
N34	0.051 (5)	0.039 (6)	0.033 (5)	0.007 (5)	0.011 (4)	0.005 (4)
C177	0.062 (8)	0.062 (8)	0.047 (7)	0.007 (7)	0.005 (6)	−0.005 (6)
C178	0.079 (9)	0.058 (8)	0.032 (6)	−0.003 (7)	0.015 (6)	0.003 (5)
O74B	0.037 (8)	0.043 (10)	0.030 (9)	0.003 (8)	0.019 (8)	0.003 (8)
C212	0.042 (7)	0.042 (8)	0.034 (7)	0.007 (7)	0.015 (6)	0.004 (7)
N34B	0.050 (8)	0.045 (8)	0.034 (8)	0.006 (8)	0.016 (7)	0.006 (7)
C213	0.062 (14)	0.049 (14)	0.045 (13)	0.000 (13)	0.018 (13)	0.009 (13)
C214	0.062 (14)	0.057 (14)	0.041 (13)	0.006 (13)	0.009 (13)	0.004 (13)
O75	0.020 (3)	0.048 (4)	0.049 (4)	0.009 (3)	0.013 (3)	0.000 (3)
C179	0.062 (7)	0.075 (7)	0.060 (6)	0.033 (6)	0.009 (5)	−0.005 (6)
N35	0.074 (7)	0.098 (8)	0.075 (7)	0.026 (7)	0.011 (6)	−0.030 (6)
C180	0.141 (15)	0.105 (13)	0.080 (10)	−0.056 (11)	0.068 (10)	−0.041 (9)
C181	0.067 (9)	0.095 (11)	0.086 (10)	0.017 (9)	0.016 (8)	−0.027 (9)
O76	0.197 (19)	0.186 (19)	0.191 (19)	−0.007 (17)	0.010 (16)	−0.010 (16)
C182	0.132 (11)	0.133 (12)	0.134 (12)	0.008 (10)	0.029 (9)	−0.013 (10)
N36	0.104 (10)	0.113 (10)	0.105 (10)	0.025 (8)	0.035 (8)	−0.029 (8)
C183	0.121 (17)	0.127 (17)	0.127 (17)	0.012 (15)	0.011 (14)	−0.040 (15)
C184	0.086 (13)	0.121 (15)	0.074 (12)	0.059 (12)	0.038 (10)	0.000 (11)
O76B	0.21 (3)	0.20 (3)	0.19 (3)	−0.01 (2)	0.051 (19)	−0.01 (2)
C215	0.17 (2)	0.17 (2)	0.17 (2)	0.005 (11)	0.046 (11)	0.001 (11)
N36B	0.17 (2)	0.16 (2)	0.17 (2)	0.009 (10)	0.045 (11)	0.005 (10)
C216	0.12 (2)	0.11 (2)	0.12 (2)	0.040 (18)	0.056 (19)	0.019 (18)
C217	0.16 (3)	0.15 (3)	0.15 (3)	0.02 (2)	0.03 (2)	0.01 (2)
O77	0.108 (10)	0.119 (10)	0.165 (13)	0.002 (9)	−0.004 (9)	0.041 (10)
C185	0.075 (7)	0.059 (6)	0.087 (7)	−0.001 (6)	0.006 (6)	0.013 (6)
N37	0.068 (6)	0.051 (5)	0.068 (6)	−0.007 (5)	0.013 (5)	−0.003 (4)
C186	0.145 (15)	0.138 (14)	0.119 (13)	−0.059 (12)	0.078 (12)	−0.034 (11)
C187	0.091 (11)	0.045 (7)	0.125 (12)	0.004 (7)	−0.038 (10)	−0.009 (8)
O78	0.095 (9)	0.056 (7)	0.026 (5)	0.028 (6)	0.001 (5)	0.001 (4)
C188	0.037 (6)	0.044 (6)	0.018 (5)	0.008 (5)	0.005 (4)	0.008 (4)
N38	0.023 (4)	0.037 (5)	0.018 (4)	−0.001 (4)	0.004 (3)	0.008 (4)
C189	0.056 (8)	0.054 (8)	0.015 (5)	0.015 (6)	0.019 (5)	0.010 (5)
C190	0.046 (7)	0.034 (6)	0.013 (5)	−0.020 (5)	0.006 (4)	−0.005 (4)
O79	0.204 (15)	0.132 (11)	0.137 (12)	−0.047 (11)	0.050 (11)	0.053 (10)
C191	0.131 (10)	0.095 (8)	0.117 (9)	−0.017 (8)	0.046 (8)	0.016 (8)
N39	0.121 (8)	0.071 (6)	0.100 (7)	−0.019 (6)	0.068 (7)	−0.009 (6)
C192	0.152 (15)	0.124 (14)	0.118 (13)	−0.021 (12)	0.100 (11)	−0.026 (10)
C193	0.157 (15)	0.106 (12)	0.137 (14)	−0.073 (11)	0.086 (12)	−0.062 (11)
O80	0.194 (19)	0.082 (11)	0.068 (10)	0.041 (11)	0.064 (11)	−0.018 (8)
C194	0.153 (15)	0.040 (9)	0.098 (12)	0.028 (10)	0.063 (11)	0.009 (9)
N40	0.129 (13)	0.052 (8)	0.092 (10)	0.031 (9)	0.054 (10)	−0.002 (8)
C195	0.123 (19)	0.093 (16)	0.069 (13)	0.023 (14)	0.042 (13)	0.017 (12)
C196	0.096 (16)	0.093 (15)	0.085 (15)	0.009 (13)	0.035 (12)	−0.031 (12)
O81	0.32 (3)	0.23 (3)	0.092 (14)	−0.04 (2)	0.003 (18)	−0.011 (16)
C197	0.27 (3)	0.19 (2)	0.090 (13)	−0.04 (2)	0.063 (15)	−0.030 (15)
N41	0.25 (3)	0.18 (2)	0.067 (11)	−0.030 (18)	0.089 (13)	−0.045 (12)
C198	0.25 (3)	0.17 (3)	0.14 (2)	−0.04 (2)	0.09 (2)	−0.027 (19)
C199	0.24 (3)	0.21 (3)	0.113 (19)	−0.01 (2)	0.08 (2)	−0.078 (19)
O82	0.136 (11)	0.065 (8)	0.068 (8)	0.052 (8)	0.039 (8)	0.002 (7)
C200	0.106 (8)	0.073 (7)	0.075 (7)	0.025 (7)	0.027 (7)	−0.007 (6)
N42	0.094 (7)	0.069 (7)	0.073 (6)	0.025 (6)	0.025 (6)	−0.005 (5)
C201	0.103 (12)	0.068 (11)	0.080 (12)	0.013 (10)	0.049 (10)	−0.012 (10)
C202	0.099 (12)	0.086 (11)	0.096 (12)	0.015 (11)	0.019 (11)	0.003 (10)
O82B	0.118 (13)	0.082 (13)	0.080 (12)	0.027 (12)	0.031 (11)	−0.002 (11)
C221	0.093 (8)	0.069 (8)	0.072 (8)	0.023 (7)	0.030 (7)	−0.007 (7)
N42B	0.095 (8)	0.072 (7)	0.075 (7)	0.019 (7)	0.028 (7)	−0.002 (6)
C222	0.101 (15)	0.071 (14)	0.077 (14)	0.021 (13)	0.031 (13)	−0.015 (12)
C223	0.098 (14)	0.070 (14)	0.068 (14)	0.015 (12)	0.045 (12)	−0.008 (12)

### Poly[[di-µ4-acetato-nonakis(µ-N,N-dimethylformamide)octakis(N,N-dimethylformamide)tetrakis[salicylhydroximato(2-)]tetradecakis[salicylhydroximato(3-)]dodecaaluminium(III)didysprosium(III)decasodium(I)] N,N-dimethylformamide 6.335-solvate] (2) Geometric parameters (Å, º)

**Table:**

Dy1—O25	2.310 (5)	C89—H89	0.9500
Dy1—O1	2.324 (5)	C90—C91	1.393 (12)
Dy1—O7	2.373 (5)	C90—H90	0.9500
Dy1—O10	2.373 (5)	C91—H91	0.9500
Dy1—O22	2.414 (5)	C92—C93	1.482 (11)
Dy1—O13	2.443 (5)	C93—C98	1.404 (12)
Dy1—O19	2.443 (5)	C93—C94	1.414 (12)
Dy1—O4	2.451 (5)	C94—C95	1.413 (12)
Dy1—O16	2.702 (5)	C95—C96	1.382 (15)
Dy1—Al1	3.374 (2)	C95—H95	0.9500
Dy1—Al2	3.381 (2)	C96—C97	1.406 (16)
Dy1—Al5	3.468 (2)	C96—H96	0.9500
Dy2—O34	2.300 (5)	C97—C98	1.380 (13)
Dy2—O43	2.310 (5)	C97—H97	0.9500
Dy2—O37	2.379 (5)	C98—H98	0.9500
Dy2—O46	2.385 (4)	C99—C100	1.462 (11)
Dy2—O28	2.438 (5)	C100—C105	1.389 (13)
Dy2—O40	2.446 (5)	C100—C101	1.406 (13)
Dy2—O52	2.457 (5)	C101—C102	1.393 (13)
Dy2—O49	2.463 (5)	C102—C103	1.405 (16)
Dy2—O31	2.701 (5)	C102—H102	0.9500
Dy2—Al12	3.367 (2)	C103—C104	1.380 (17)
Dy2—Al11	3.411 (2)	C103—H103	0.9500
Dy2—Al7	3.469 (2)	C104—C105	1.381 (14)
Al1—O3	1.812 (6)	C104—H104	0.9500
Al1—O8	1.891 (6)	C105—H105	0.9500
Al1—O5	1.907 (6)	C106—C107	1.470 (11)
Al1—O7	1.916 (5)	C107—C112	1.394 (12)
Al1—O4	1.941 (6)	C107—C108	1.412 (12)
Al1—N1	2.002 (7)	C108—C109	1.404 (12)
Al1—Na2	3.341 (3)	C109—C110	1.379 (13)
Al2—O6	1.814 (5)	C109—H109	0.9500
Al2—O11	1.888 (5)	C110—C111	1.392 (13)
Al2—O56	1.908 (6)	C110—H110	0.9500
Al2—O10	1.909 (5)	C111—C112	1.381 (12)
Al2—O13	1.909 (5)	C111—H111	0.9500
Al2—N2	2.024 (6)	C112—H112	0.9500
Al2—Na5	3.388 (4)	C113—C114	1.475 (11)
Al2—Na4	3.465 (4)	C114—C119	1.380 (12)
Al3—O12	1.785 (6)	C114—C115	1.428 (12)
Al3—O18	1.794 (7)	C115—C116	1.422 (11)
Al3—O20	1.839 (6)	C116—C117	1.396 (12)
Al3—O19	1.875 (6)	C116—H116	0.9500
Al3—N4	1.930 (7)	C117—C118	1.379 (14)
Al4—O15	1.768 (6)	C117—H117	0.9500
Al4—O9	1.790 (6)	C118—C119	1.393 (13)
Al4—O23	1.848 (6)	C118—H118	0.9500
Al4—O22	1.883 (5)	C119—H119	0.9500
Al4—N3	1.912 (7)	C120—C121	1.483 (10)
Al5—O24	1.819 (6)	C121—C122	1.397 (11)
Al5—O25	1.881 (6)	C121—C126	1.409 (11)
Al5—O17	1.923 (6)	C122—C123	1.415 (11)
Al5—O26	1.924 (6)	C123—C124	1.398 (12)
Al5—O16	1.958 (5)	C123—H123	0.9500
Al5—N8	1.961 (7)	C124—C125	1.394 (13)
Al5—Na3	2.993 (4)	C124—H124	0.9500
Al6—O21	1.845 (6)	C125—C126	1.376 (12)
Al6—O27	1.866 (6)	C125—H125	0.9500
Al6—O2	1.892 (6)	C126—H126	0.9500
Al6—O1	1.898 (6)	C127—C128	1.508 (12)
Al6—N9	1.956 (7)	C128—H12A	0.9800
Al6—N7	2.056 (7)	C128—H12B	0.9800
Al6—Na1	3.058 (4)	C128—H12C	0.9800
Al7—O30	1.829 (6)	C129—C130	1.483 (12)
Al7—O34	1.883 (5)	C130—H13A	0.9800
Al7—O32	1.913 (6)	C130—H13B	0.9800
Al7—O35	1.919 (6)	C130—H13C	0.9800
Al7—N10	1.953 (7)	O59—C131	1.248 (11)
Al7—O31	1.978 (6)	C131—N19	1.269 (12)
Al7—Na8	3.029 (4)	C131—H131	0.9500
Al8—O42	1.751 (6)	N19—C133	1.409 (12)
Al8—O39	1.784 (6)	N19—C132	1.507 (13)
Al8—O29	1.860 (6)	C132—H13D	0.9800
Al8—O28	1.900 (5)	C132—H13E	0.9800
Al8—N13	1.958 (7)	C132—H13F	0.9800
Al9—O51	1.840 (6)	C133—H13G	0.9800
Al9—O36	1.879 (6)	C133—H13H	0.9800
Al9—O44	1.887 (6)	C133—H13I	0.9800
Al9—O43	1.899 (6)	O60—C134	1.209 (12)
Al9—N12	1.973 (7)	C134—N20	1.312 (13)
Al9—N17	2.064 (7)	C134—H134	0.9500
Al9—Na10	3.057 (4)	N20—C136	1.432 (14)
Al10—O48	1.785 (6)	N20—C135	1.451 (16)
Al10—O33	1.799 (7)	C135—H13J	0.9800
Al10—O50	1.824 (6)	C135—H13K	0.9800
Al10—O49	1.871 (5)	C135—H13L	0.9800
Al10—N16	1.930 (7)	C136—H13M	0.9800
Al11—O54	1.812 (6)	C136—H13N	0.9800
Al11—O47	1.877 (6)	C136—H13O	0.9800
Al11—O58	1.881 (5)	O60B—C224	1.23 (2)
Al11—O46	1.906 (5)	C224—N20B	1.309 (19)
Al11—O40	1.945 (5)	C224—H224	0.9500
Al11—N18	2.021 (7)	N20B—C226	1.45 (2)
Al11—Na6	3.470 (4)	N20B—C225	1.46 (2)
Al11—Na7	3.594 (4)	C225—H22J	0.9800
Al12—O45	1.841 (5)	C225—H22K	0.9800
Al12—O38	1.888 (6)	C225—H22L	0.9800
Al12—O53	1.898 (5)	C226—H22M	0.9800
Al12—O37	1.919 (5)	C226—H22N	0.9800
Al12—O52	1.920 (5)	C226—H22O	0.9800
Al12—N15	1.995 (6)	O61—C137	1.226 (10)
Al12—Na9^i^	3.353 (3)	C137—N21	1.333 (11)
Na1—O75^ii^	2.344 (7)	C137—H137	0.9500
Na1—O21	2.366 (7)	N21—C138	1.451 (11)
Na1—O59	2.367 (8)	N21—C139	1.454 (12)
Na1—O60B	2.397 (8)	C138—H13P	0.9800
Na1—O60	2.397 (8)	C138—H13Q	0.9800
Na1—O2	2.495 (6)	C138—H13R	0.9800
Na1—O27	2.585 (7)	C139—H13S	0.9800
Na1—Na10^ii^	3.540 (4)	C139—H13T	0.9800
Na2—O63B	2.338 (7)	C139—H13U	0.9800
Na2—O63	2.338 (7)	O62—C140	1.223 (10)
Na2—O3	2.353 (6)	C140—N22	1.288 (11)
Na2—O62	2.386 (7)	C140—H140	0.9500
Na2—O61	2.436 (8)	N22—C141	1.464 (12)
Na2—O64B	2.44 (7)	N22—C142	1.465 (12)
Na2—O64	2.46 (4)	C141—H14A	0.9800
Na2—O5	2.471 (6)	C141—H14B	0.9800
Na2—C203	3.04 (4)	C141—H14C	0.9800
Na2—Na3^iii^	3.112 (4)	C142—H14D	0.9800
Na3—O64^i^	2.28 (3)	C142—H14E	0.9800
Na3—O63^i^	2.307 (7)	C142—H14F	0.9800
Na3—O62^i^	2.312 (7)	O63—C143	1.235 (11)
Na3—O26	2.423 (6)	C143—N23	1.321 (13)
Na3—O17	2.466 (6)	C143—H143	0.9500
Na3—O24	2.497 (6)	N23—C144	1.434 (13)
Na4—O14	2.319 (6)	N23—C145	1.452 (14)
Na4—O65	2.327 (6)	C144—H14G	0.9800
Na4—O55	2.344 (7)	C144—H14H	0.9800
Na4—O16	2.387 (6)	C144—H14I	0.9800
Na4—O13	2.505 (6)	C145—H14J	0.9800
Na4—O56	2.652 (6)	C145—H14K	0.9800
Na4—C127	2.825 (9)	C145—H14L	0.9800
Na4—C29	2.959 (8)	O63B—C203	1.37 (4)
Na4—N6	2.975 (8)	C203—N23B	1.24 (7)
Na4—Na6	3.601 (4)	C203—H203	0.9500
Na4—Na5	3.669 (4)	N23B—C205	1.47 (7)
Na5—O67	2.316 (7)	N23B—C204	1.58 (7)
Na5—O57	2.331 (6)	C204—H20G	0.9800
Na5—O66	2.407 (8)	C204—H20H	0.9800
Na5—O56	2.432 (6)	C204—H20I	0.9800
Na5—O6	2.485 (6)	C205—H20J	0.9800
Na5—O14	2.510 (6)	C205—H20K	0.9800
Na6—O68	2.353 (7)	C205—H20L	0.9800
Na6—O55	2.361 (7)	O64—C146	1.232 (19)
Na6—O41	2.367 (6)	C146—N24	1.322 (14)
Na6—O58	2.386 (6)	C146—H146	0.9500
Na6—O65	2.471 (7)	N24—C147	1.459 (15)
Na6—O54	2.633 (6)	N24—C148	1.466 (14)
Na6—Na7	3.660 (5)	C147—H14M	0.9800
Na7—O69B	2.30 (6)	C147—H14O	0.9800
Na7—O69	2.343 (14)	C147—H14P	0.9800
Na7—O40	2.364 (6)	C148—H14Q	0.9800
Na7—O57	2.394 (6)	C148—H14R	0.9800
Na7—O31	2.536 (6)	C148—H14S	0.9800
Na7—O41	2.554 (6)	O64B—C230	1.23 (2)
Na7—O58	2.839 (7)	C230—N24B	1.314 (17)
Na7—C129	2.927 (9)	C230—H230	0.9500
Na7—N14	2.974 (7)	N24B—C232	1.455 (18)
Na7—C92	2.987 (8)	N24B—C231	1.461 (18)
Na8—O70B	2.295 (7)	C231—H23A	0.9800
Na8—O70	2.295 (7)	C231—H23B	0.9800
Na8—O71	2.357 (7)	C231—H23C	0.9800
Na8—O72	2.361 (6)	C232—H23D	0.9800
Na8—O32	2.416 (6)	C232—H23E	0.9800
Na8—O35	2.447 (6)	C232—H23F	0.9800
Na8—O30	2.563 (6)	O65—C149	1.231 (10)
Na8—Na9	3.116 (4)	C149—N25	1.325 (11)
Na9—O71	2.368 (6)	C149—H149	0.9500
Na9—O72	2.371 (6)	N25—C151	1.426 (12)
Na9—O45^iii^	2.380 (6)	N25—C150	1.461 (13)
Na9—O73^iii^	2.425 (7)	C150—H15A	0.9800
Na9—O53^iii^	2.433 (6)	C150—H15B	0.9800
Na9—O70B	2.476 (7)	C150—H15C	0.9800
Na9—O70	2.476 (7)	C151—H15D	0.9800
Na9—C209	3.08 (2)	C151—H15E	0.9800
Na10—O74B	2.24 (7)	C151—H15F	0.9800
Na10—O74	2.316 (13)	O66—C152	1.232 (12)
Na10—O59^iv^	2.355 (7)	C152—N26	1.356 (12)
Na10—O75	2.358 (8)	C152—H152	0.9500
Na10—O51	2.396 (7)	N26—C153	1.432 (12)
Na10—O36	2.523 (6)	N26—C154	1.434 (13)
Na10—O44	2.548 (6)	C153—H15G	0.9800
Na10—C179	3.090 (14)	C153—H15H	0.9800
Na10—C212	3.12 (4)	C153—H15I	0.9800
Na10—C115	3.128 (9)	C154—H15J	0.9800
O1—N1	1.411 (8)	C154—H15K	0.9800
O2—C1	1.299 (10)	C154—H15L	0.9800
O3—C3	1.354 (10)	O67—C155	1.206 (11)
O4—N2	1.422 (8)	C155—N27	1.340 (11)
O5—C8	1.292 (9)	C155—H155	0.9500
O6—C10	1.315 (9)	N27—C156	1.420 (13)
O7—N3	1.416 (7)	N27—C157	1.462 (13)
O8—C15	1.292 (10)	C156—H15M	0.9800
O9—C17	1.318 (10)	C156—H15N	0.9800
O10—N4	1.404 (8)	C156—H15O	0.9800
O11—C22	1.293 (10)	C157—H15P	0.9800
O12—C24	1.307 (11)	C157—H15Q	0.9800
O13—N5	1.386 (7)	C157—H15R	0.9800
O14—C29	1.244 (9)	O68—C158	1.229 (11)
O15—C31	1.358 (9)	C158—N28	1.322 (11)
O16—N6	1.393 (8)	C158—H158	0.9500
O17—C36	1.284 (10)	N28—C160	1.452 (12)
O18—C38	1.337 (10)	N28—C159	1.459 (12)
O19—N7	1.426 (8)	C159—H15S	0.9800
O20—C43	1.294 (10)	C159—H15T	0.9800
O21—C45	1.326 (10)	C159—H15U	0.9800
O22—N8	1.414 (8)	C160—H16A	0.9800
O23—C50B	1.300 (10)	C160—H16B	0.9800
O23—C50	1.300 (10)	C160—H16C	0.9800
O24—C52	1.30 (3)	O69—C161	1.239 (14)
O24—C52B	1.35 (3)	C161—N29	1.307 (12)
O25—N9	1.408 (8)	C161—H161	0.9500
O26—C57	1.314 (10)	N29—C163	1.459 (13)
O27—C59	1.317 (10)	N29—C162	1.466 (14)
O28—N10	1.411 (8)	C162—H16D	0.9800
O29—C64	1.288 (10)	C162—H16E	0.9800
O30—C66	1.342 (11)	C162—H16F	0.9800
O31—N11	1.381 (8)	C163—H16G	0.9800
O32—C71	1.299 (10)	C163—H16H	0.9800
O33—C73	1.335 (11)	C163—H16I	0.9800
O34—N12	1.397 (8)	O69B—C206	1.24 (2)
O35—C78	1.322 (10)	C206—N29B	1.314 (18)
O36—C80	1.326 (10)	C206—H206	0.9500
O37—N13	1.402 (7)	N29B—C208	1.445 (19)
O38—C85	1.286 (10)	N29B—C207	1.454 (19)
O39—C87	1.320 (11)	C207—H20M	0.9800
O40—N14	1.388 (8)	C207—H20N	0.9800
O41—C92	1.252 (10)	C207—H20O	0.9800
O42—C94	1.316 (10)	C208—H20P	0.9800
O43—N15	1.405 (8)	C208—H20Q	0.9800
O44—C99	1.303 (10)	C208—H20R	0.9800
O45—C101	1.335 (11)	O70—C164	1.214 (13)
O46—N16	1.413 (8)	C164—N30	1.309 (14)
O47—C106	1.293 (10)	C164—H164	0.9500
O48—C108	1.331 (10)	N30—C166	1.453 (15)
O49—N17	1.424 (8)	N30—C165	1.468 (15)
O50—C113	1.306 (10)	C165—H16J	0.9800
O51—C115	1.293 (10)	C165—H16K	0.9800
O52—N18	1.430 (8)	C165—H16L	0.9800
O53—C120	1.310 (9)	C166—H16M	0.9800
O54—C122	1.336 (9)	C166—H16N	0.9800
O55—C127	1.219 (10)	C166—H16O	0.9800
O56—C127	1.288 (9)	O70B—C209	1.238 (18)
O57—C129	1.260 (10)	C209—N30B	1.292 (17)
O58—C129	1.288 (10)	C209—H209	0.9500
N1—C1	1.317 (10)	N30B—C211	1.440 (18)
N2—C8	1.312 (10)	N30B—C210	1.479 (19)
N3—C15	1.313 (10)	C210—H21A	0.9800
N4—C22	1.298 (10)	C210—H21B	0.9800
N5—C29	1.341 (10)	C210—H21C	0.9800
N5—H5N	0.89 (3)	C211—H21D	0.9800
N6—C36	1.302 (10)	C211—H21E	0.9800
N6—H6N	0.87 (3)	C211—H21F	0.9800
N7—C43	1.321 (10)	O71—C167	1.238 (9)
N8—C50B	1.310 (10)	C167—N31	1.320 (10)
N8—C50	1.310 (10)	C167—H167	0.9500
N9—C57	1.302 (10)	N31—C168	1.449 (10)
N10—C64	1.295 (11)	N31—C169	1.459 (11)
N11—C71	1.308 (11)	C168—H16P	0.9800
N11—H11N	0.87 (3)	C168—H16Q	0.9800
N12—C78	1.291 (10)	C168—H16R	0.9800
N13—C85	1.304 (10)	C169—H16S	0.9800
N14—C92	1.322 (11)	C169—H16T	0.9800
N14—H14N	0.89 (3)	C169—H16U	0.9800
N15—C99	1.321 (10)	O72—C170	1.225 (10)
N16—C106	1.313 (10)	C170—N32	1.325 (10)
N17—C113	1.305 (10)	C170—H170	0.9500
N18—C120	1.317 (10)	N32—C172	1.443 (11)
C1—C2	1.463 (12)	N32—C171	1.465 (10)
C2—C3	1.401 (13)	C171—H17A	0.9800
C2—C7	1.433 (12)	C171—H17B	0.9800
C3—C4	1.393 (12)	C171—H17C	0.9800
C4—C5	1.387 (15)	C172—H17D	0.9800
C4—H4	0.9500	C172—H17E	0.9800
C5—C6	1.393 (17)	C172—H17F	0.9800
C5—H5	0.9500	O73—C173	1.237 (9)
C6—C7	1.359 (14)	C173—N33	1.313 (10)
C6—H6	0.9500	C173—H173	0.9500
C7—H7	0.9500	N33—C175	1.442 (11)
C8—C9	1.473 (10)	N33—C174	1.444 (11)
C9—C14	1.404 (10)	C174—H17G	0.9800
C9—C10	1.414 (11)	C174—H17H	0.9800
C10—C11	1.408 (10)	C174—H17I	0.9800
C11—C12	1.386 (11)	C175—H17J	0.9800
C11—H11	0.9500	C175—H17K	0.9800
C12—C13	1.389 (13)	C175—H17L	0.9800
C12—H12	0.9500	O74—C176	1.238 (13)
C13—C14	1.399 (12)	C176—N34	1.323 (13)
C13—H13	0.9500	C176—H176	0.9500
C14—H14	0.9500	N34—C177	1.447 (14)
C15—C16	1.480 (11)	N34—C178	1.449 (13)
C16—C21	1.398 (12)	C177—H17M	0.9800
C16—C17	1.414 (12)	C177—H17N	0.9800
C17—C18	1.421 (11)	C177—H17O	0.9800
C18—C19	1.380 (14)	C178—H17P	0.9800
C18—H18	0.9500	C178—H17Q	0.9800
C19—C20	1.377 (16)	C178—H17R	0.9800
C19—H19	0.9500	O74B—C212	1.23 (2)
C20—C21	1.381 (13)	C212—N34B	1.319 (19)
C20—H20	0.9500	C212—H212	0.9500
C21—H21	0.9500	N34B—C214	1.452 (19)
C22—C23	1.482 (10)	N34B—C213	1.46 (2)
C23—C28	1.385 (13)	C213—H21G	0.9800
C23—C24	1.420 (12)	C213—H21H	0.9800
C24—C25	1.423 (11)	C213—H21I	0.9800
C25—C26	1.378 (15)	C214—H21J	0.9800
C25—H25	0.9500	C214—H21K	0.9800
C26—C27	1.389 (16)	C214—H21L	0.9800
C26—H26	0.9500	O75—C179	1.214 (12)
C27—C28	1.375 (12)	C179—N35	1.287 (13)
C27—H27	0.9500	C179—H179	0.9500
C28—H28	0.9500	N35—C181	1.419 (14)
C29—C30	1.486 (10)	N35—C180	1.490 (15)
C30—C31	1.396 (11)	C180—H18A	0.9800
C30—C35	1.405 (11)	C180—H18B	0.9800
C31—C32	1.395 (11)	C180—H18C	0.9800
C32—C33	1.398 (13)	C181—H18D	0.9800
C32—H32	0.9500	C181—H18E	0.9800
C33—C34	1.371 (14)	C181—H18F	0.9800
C33—H33	0.9500	O76—C182	1.244 (19)
C34—C35	1.378 (12)	C182—N36	1.300 (17)
C34—H34	0.9500	C182—H182	0.9500
C35—H35	0.9500	N36—C183	1.438 (18)
C36—C37	1.468 (11)	N36—C184	1.448 (17)
C37—C38	1.395 (13)	C183—H18G	0.9800
C37—C42	1.402 (12)	C183—H18H	0.9800
C38—C39	1.411 (12)	C183—H18I	0.9800
C39—C40	1.392 (15)	C184—H18J	0.9800
C39—H39	0.9500	C184—H18K	0.9800
C40—C41	1.351 (17)	C184—H18L	0.9800
C40—H40	0.9500	O76B—C215	1.248 (12)
C41—C42	1.398 (14)	C215—N36B	1.269 (12)
C41—H41	0.9500	C215—H215	0.9500
C42—H42	0.9500	N36B—C217	1.409 (12)
C43—C44	1.469 (12)	N36B—C216	1.507 (13)
C44—C49	1.386 (12)	C216—H21P	0.9800
C44—C45	1.399 (13)	C216—H21Q	0.9800
C45—C46	1.407 (12)	C216—H21R	0.9800
C46—C47	1.357 (14)	C217—H21M	0.9800
C46—H46	0.9500	C217—H21N	0.9800
C47—C48	1.390 (15)	C217—H21O	0.9800
C47—H47	0.9500	O77—C185	1.205 (14)
C48—C49	1.392 (13)	C185—N37	1.330 (14)
C48—H48	0.9500	C185—H185	0.9500
C49—H49	0.9500	N37—C187	1.421 (14)
C50—C51	1.462 (16)	N37—C186	1.450 (15)
C51—C56	1.399 (17)	C186—H18M	0.9800
C51—C52	1.402 (16)	C186—H18N	0.9800
C52—C53	1.406 (16)	C186—H18O	0.9800
C53—C54	1.398 (18)	C187—H18P	0.9800
C53—H53	0.9500	C187—H18Q	0.9800
C54—C55	1.370 (19)	C187—H18R	0.9800
C54—H54	0.9500	O78—C188	1.231 (12)
C55—C56	1.380 (17)	C188—N38	1.315 (12)
C55—H55	0.9500	C188—H188	0.9500
C56—H56	0.9500	N38—C190	1.442 (12)
C50B—C51B	1.456 (18)	N38—C189	1.449 (11)
C51B—C56B	1.395 (19)	C189—H18S	0.9800
C51B—C52B	1.401 (19)	C189—H18T	0.9800
C52B—C53B	1.399 (19)	C189—H18U	0.9800
C53B—C54B	1.40 (2)	C190—H19A	0.9800
C53B—H53B	0.9500	C190—H19B	0.9800
C54B—C55B	1.39 (2)	C190—H19C	0.9800
C54B—H54B	0.9500	O79—C191	1.252 (16)
C55B—C56B	1.378 (19)	C191—N39	1.314 (16)
C55B—H55B	0.9500	C191—H191	0.9500
C56B—H56B	0.9500	N39—C193	1.456 (15)
C57—C58	1.466 (12)	N39—C192	1.458 (15)
C58—C63	1.404 (13)	C192—H19D	0.9800
C58—C59	1.414 (13)	C192—H19E	0.9800
C59—C60	1.388 (13)	C192—H19F	0.9800
C60—C61	1.387 (15)	C193—H19G	0.9800
C60—H60	0.9500	C193—H19H	0.9800
C61—C62	1.390 (18)	C193—H19I	0.9800
C61—H61	0.9500	O80—C194	1.219 (17)
C62—C63	1.390 (16)	C194—N40	1.319 (16)
C62—H62	0.9500	C194—H194	0.9500
C63—H63	0.9500	N40—C196	1.465 (17)
C64—C65	1.460 (11)	N40—C195	1.479 (16)
C65—C66	1.398 (13)	C195—H19J	0.9800
C65—C70	1.413 (13)	C195—H19K	0.9800
C66—C67	1.401 (12)	C195—H19L	0.9800
C67—C68	1.397 (15)	C196—H19M	0.9800
C67—H67	0.9500	C196—H19N	0.9800
C68—C69	1.382 (18)	C196—H19O	0.9800
C68—H68	0.9500	O81—C197	1.263 (19)
C69—C70	1.374 (15)	C197—N41	1.302 (18)
C69—H69	0.9500	C197—H197	0.9500
C70—H70	0.9500	N41—C199	1.443 (18)
C71—C72	1.473 (12)	N41—C198	1.450 (18)
C72—C73	1.404 (13)	C198—H19P	0.9800
C72—C77	1.406 (13)	C198—H19Q	0.9800
C73—C74	1.406 (13)	C198—H19R	0.9800
C74—C75	1.393 (15)	C199—H19S	0.9800
C74—H74	0.9500	C199—H19T	0.9800
C75—C76	1.391 (17)	C199—H19U	0.9800
C75—H75	0.9500	O82—C200	1.246 (15)
C76—C77	1.351 (15)	C200—N42	1.276 (16)
C76—H76	0.9500	C200—H200	0.9500
C77—H77	0.9500	N42—C202	1.416 (17)
C78—C79	1.467 (12)	N42—C201	1.486 (16)
C79—C84	1.394 (12)	C201—H20D	0.9800
C79—C80	1.410 (12)	C201—H20E	0.9800
C80—C81	1.399 (12)	C201—H20F	0.9800
C81—C82	1.399 (14)	C202—H20A	0.9800
C81—H81	0.9500	C202—H20B	0.9800
C82—C83	1.345 (18)	C202—H20C	0.9800
C82—H82	0.9500	O82B—C221	1.252 (16)
C83—C84	1.402 (16)	C221—N42B	1.275 (16)
C83—H83	0.9500	C221—H221	0.9500
C84—H84	0.9500	N42B—C223	1.408 (17)
C85—C86	1.495 (10)	N42B—C222	1.502 (17)
C86—C91	1.388 (12)	C222—H22D	0.9800
C86—C87	1.400 (12)	C222—H22E	0.9800
C87—C88	1.415 (11)	C222—H22F	0.9800
C88—C89	1.380 (14)	C223—H22G	0.9800
C88—H88	0.9500	C223—H22H	0.9800
C89—C90	1.384 (14)	C223—H22I	0.9800
			
O25—Dy1—O1	73.69 (18)	O2—C1—C2	121.1 (7)
O25—Dy1—O7	126.66 (17)	N1—C1—C2	119.0 (7)
O1—Dy1—O7	71.43 (17)	C3—C2—C7	119.5 (8)
O25—Dy1—O10	113.89 (17)	C3—C2—C1	123.2 (8)
O1—Dy1—O10	136.32 (17)	C7—C2—C1	117.3 (8)
O7—Dy1—O10	119.28 (17)	O3—C3—C4	118.6 (8)
O25—Dy1—O22	72.59 (17)	O3—C3—C2	123.1 (7)
O1—Dy1—O22	86.98 (17)	C4—C3—C2	118.3 (8)
O7—Dy1—O22	66.54 (16)	C5—C4—C3	121.5 (10)
O10—Dy1—O22	136.70 (17)	C5—C4—H4	119.2
O25—Dy1—O13	127.49 (17)	C3—C4—H4	119.2
O1—Dy1—O13	151.53 (17)	C4—C5—C6	119.9 (10)
O7—Dy1—O13	80.15 (16)	C4—C5—H5	120.1
O10—Dy1—O13	58.46 (16)	C6—C5—H5	120.1
O22—Dy1—O13	83.09 (17)	C7—C6—C5	120.2 (10)
O25—Dy1—O19	72.79 (17)	C7—C6—H6	119.9
O1—Dy1—O19	72.18 (18)	C5—C6—H6	119.9
O7—Dy1—O19	129.45 (18)	C6—C7—C2	120.3 (9)
O10—Dy1—O19	69.99 (17)	C6—C7—H7	119.8
O22—Dy1—O19	143.38 (17)	C2—C7—H7	119.8
O13—Dy1—O19	128.45 (16)	O5—C8—N2	120.0 (7)
O25—Dy1—O4	148.56 (17)	O5—C8—C9	120.1 (6)
O1—Dy1—O4	82.10 (17)	N2—C8—C9	119.9 (6)
O7—Dy1—O4	60.34 (16)	C14—C9—C10	119.9 (7)
O10—Dy1—O4	71.22 (17)	C14—C9—C8	116.9 (7)
O22—Dy1—O4	126.53 (16)	C10—C9—C8	123.2 (6)
O13—Dy1—O4	82.57 (16)	O6—C10—C11	117.6 (7)
O19—Dy1—O4	80.98 (17)	O6—C10—C9	124.4 (6)
O25—Dy1—O16	57.78 (17)	C11—C10—C9	118.0 (7)
O1—Dy1—O16	131.06 (16)	C12—C11—C10	121.3 (7)
O7—Dy1—O16	133.08 (16)	C12—C11—H11	119.4
O10—Dy1—O16	75.33 (16)	C10—C11—H11	119.4
O22—Dy1—O16	73.72 (15)	C11—C12—C13	121.1 (8)
O13—Dy1—O16	71.08 (16)	C11—C12—H12	119.5
O19—Dy1—O16	97.37 (17)	C13—C12—H12	119.5
O4—Dy1—O16	144.94 (16)	C12—C13—C14	118.6 (8)
O25—Dy1—Al1	131.60 (13)	C12—C13—H13	120.7
O1—Dy1—Al1	58.34 (13)	C14—C13—H13	120.7
O7—Dy1—Al1	33.55 (12)	C13—C14—C9	121.2 (7)
O10—Dy1—Al1	105.48 (13)	C13—C14—H14	119.4
O22—Dy1—Al1	97.37 (12)	C9—C14—H14	119.4
O13—Dy1—Al1	96.54 (12)	O8—C15—N3	121.2 (7)
O19—Dy1—Al1	96.67 (14)	O8—C15—C16	120.6 (7)
O4—Dy1—Al1	34.55 (12)	N3—C15—C16	118.2 (7)
O16—Dy1—Al1	165.26 (11)	C21—C16—C17	120.7 (7)
O25—Dy1—Al2	141.71 (13)	C21—C16—C15	118.2 (8)
O1—Dy1—Al2	140.44 (13)	C17—C16—C15	121.1 (7)
O7—Dy1—Al2	88.15 (12)	O9—C17—C16	124.7 (7)
O10—Dy1—Al2	33.26 (13)	O9—C17—C18	118.5 (8)
O22—Dy1—Al2	116.07 (13)	C16—C17—C18	116.8 (8)
O13—Dy1—Al2	33.63 (11)	C19—C18—C17	120.5 (9)
O19—Dy1—Al2	98.54 (13)	C19—C18—H18	119.7
O4—Dy1—Al2	58.35 (12)	C17—C18—H18	119.7
O16—Dy1—Al2	87.67 (11)	C20—C19—C18	122.3 (9)
Al1—Dy1—Al2	85.83 (5)	C20—C19—H19	118.9
O25—Dy1—Al5	30.36 (13)	C18—C19—H19	118.9
O1—Dy1—Al5	98.16 (13)	C19—C20—C21	118.3 (9)
O7—Dy1—Al5	121.74 (12)	C19—C20—H20	120.9
O10—Dy1—Al5	107.34 (13)	C21—C20—H20	120.9
O22—Dy1—Al5	55.58 (11)	C20—C21—C16	121.4 (9)
O13—Dy1—Al5	98.03 (12)	C20—C21—H21	119.3
O19—Dy1—Al5	97.09 (13)	C16—C21—H21	119.3
O4—Dy1—Al5	177.88 (12)	O11—C22—N4	121.6 (7)
O16—Dy1—Al5	34.24 (11)	O11—C22—C23	118.8 (7)
Al1—Dy1—Al5	147.05 (5)	N4—C22—C23	119.6 (7)
Al2—Dy1—Al5	121.32 (5)	C28—C23—C24	120.3 (7)
O34—Dy2—O43	73.64 (18)	C28—C23—C22	119.5 (8)
O34—Dy2—O37	127.44 (17)	C24—C23—C22	120.2 (7)
O43—Dy2—O37	72.98 (18)	O12—C24—C23	124.7 (7)
O34—Dy2—O46	114.33 (17)	O12—C24—C25	118.4 (8)
O43—Dy2—O46	135.02 (17)	C23—C24—C25	116.8 (8)
O37—Dy2—O46	118.05 (17)	C26—C25—C24	120.7 (9)
O34—Dy2—O28	73.83 (17)	C26—C25—H25	119.7
O43—Dy2—O28	89.46 (17)	C24—C25—H25	119.7
O37—Dy2—O28	66.47 (16)	C25—C26—C27	121.8 (8)
O46—Dy2—O28	135.50 (17)	C25—C26—H26	119.1
O34—Dy2—O40	129.56 (17)	C27—C26—H26	119.1
O43—Dy2—O40	149.72 (17)	C28—C27—C26	118.1 (9)
O37—Dy2—O40	76.88 (17)	C28—C27—H27	120.9
O46—Dy2—O40	59.65 (16)	C26—C27—H27	120.9
O28—Dy2—O40	81.11 (17)	C27—C28—C23	122.2 (9)
O34—Dy2—O52	148.25 (17)	C27—C28—H28	118.9
O43—Dy2—O52	81.48 (17)	C23—C28—H28	118.9
O37—Dy2—O52	60.00 (16)	O14—C29—N5	121.8 (7)
O46—Dy2—O52	70.88 (16)	O14—C29—C30	121.6 (6)
O28—Dy2—O52	126.06 (16)	N5—C29—C30	116.5 (6)
O40—Dy2—O52	81.10 (16)	O14—C29—Na4	48.0 (4)
O34—Dy2—O49	72.53 (17)	N5—C29—Na4	85.5 (4)
O43—Dy2—O49	72.44 (17)	C30—C29—Na4	140.6 (5)
O37—Dy2—O49	131.05 (17)	C31—C30—C35	118.7 (7)
O46—Dy2—O49	68.98 (17)	C31—C30—C29	123.6 (7)
O28—Dy2—O49	145.10 (17)	C35—C30—C29	117.1 (7)
O40—Dy2—O49	128.61 (16)	O15—C31—C32	117.9 (7)
O52—Dy2—O49	81.46 (17)	O15—C31—C30	121.6 (7)
O34—Dy2—O31	58.36 (18)	C32—C31—C30	120.5 (7)
O43—Dy2—O31	131.48 (17)	C31—C32—C33	119.3 (8)
O37—Dy2—O31	131.17 (16)	C31—C32—H32	120.3
O46—Dy2—O31	76.65 (16)	C33—C32—H32	120.3
O28—Dy2—O31	71.86 (16)	C34—C33—C32	120.2 (8)
O40—Dy2—O31	72.55 (16)	C34—C33—H33	119.9
O52—Dy2—O31	145.49 (16)	C32—C33—H33	119.9
O49—Dy2—O31	97.74 (17)	C33—C34—C35	120.9 (8)
O34—Dy2—Al12	131.78 (13)	C33—C34—H34	119.6
O43—Dy2—Al12	58.67 (13)	C35—C34—H34	119.6
O37—Dy2—Al12	33.79 (12)	C34—C35—C30	120.3 (8)
O46—Dy2—Al12	104.72 (12)	C34—C35—H35	119.9
O28—Dy2—Al12	97.64 (12)	C30—C35—H35	119.9
O40—Dy2—Al12	93.97 (12)	O17—C36—N6	118.2 (7)
O52—Dy2—Al12	34.17 (12)	O17—C36—C37	121.4 (7)
O49—Dy2—Al12	97.78 (13)	N6—C36—C37	120.4 (7)
O31—Dy2—Al12	163.77 (12)	C38—C37—C42	120.3 (8)
O34—Dy2—Al11	141.76 (13)	C38—C37—C36	121.7 (7)
O43—Dy2—Al11	139.75 (13)	C42—C37—C36	117.9 (8)
O37—Dy2—Al11	87.41 (13)	O18—C38—C37	122.3 (7)
O46—Dy2—Al11	32.71 (12)	O18—C38—C39	119.2 (8)
O28—Dy2—Al11	114.85 (13)	C37—C38—C39	118.5 (8)
O40—Dy2—Al11	34.00 (12)	C40—C39—C38	119.8 (9)
O52—Dy2—Al11	58.34 (12)	C40—C39—H39	120.1
O49—Dy2—Al11	97.43 (12)	C38—C39—H39	120.1
O31—Dy2—Al11	87.86 (12)	C41—C40—C39	121.6 (9)
Al12—Dy2—Al11	85.48 (5)	C41—C40—H40	119.2
O34—Dy2—Al7	30.30 (13)	C39—C40—H40	119.2
O43—Dy2—Al7	97.96 (13)	C40—C41—C42	119.9 (9)
O37—Dy2—Al7	121.45 (12)	C40—C41—H41	120.1
O46—Dy2—Al7	108.59 (12)	C42—C41—H41	120.1
O28—Dy2—Al7	55.52 (12)	C41—C42—C37	119.9 (10)
O40—Dy2—Al7	100.10 (12)	C41—C42—H42	120.1
O52—Dy2—Al7	178.26 (12)	C37—C42—H42	120.1
O49—Dy2—Al7	96.80 (13)	O20—C43—N7	119.7 (7)
O31—Dy2—Al7	34.64 (12)	O20—C43—C44	118.7 (7)
Al12—Dy2—Al7	146.56 (5)	N7—C43—C44	121.6 (7)
Al11—Dy2—Al7	122.13 (5)	C49—C44—C45	120.0 (8)
O3—Al1—O8	93.9 (3)	C49—C44—C43	117.4 (8)
O3—Al1—O5	88.5 (2)	C45—C44—C43	122.7 (7)
O8—Al1—O5	96.5 (2)	O21—C45—C44	122.9 (7)
O3—Al1—O7	174.6 (3)	O21—C45—C46	119.3 (8)
O8—Al1—O7	81.4 (2)	C44—C45—C46	117.8 (8)
O5—Al1—O7	94.6 (2)	C47—C46—C45	122.1 (9)
O3—Al1—O4	106.9 (3)	C47—C46—H46	119.0
O8—Al1—O4	159.0 (2)	C45—C46—H46	119.0
O5—Al1—O4	81.5 (2)	C46—C47—C48	120.1 (8)
O7—Al1—O4	77.9 (2)	C46—C47—H47	120.0
O3—Al1—N1	87.8 (3)	C48—C47—H47	120.0
O8—Al1—N1	96.9 (3)	C47—C48—C49	119.1 (8)
O5—Al1—N1	166.3 (3)	C47—C48—H48	120.5
O7—Al1—N1	90.1 (2)	C49—C48—H48	120.5
O4—Al1—N1	87.0 (2)	C44—C49—C48	120.9 (9)
O3—Al1—Na2	42.62 (18)	C44—C49—H49	119.5
O8—Al1—Na2	105.21 (18)	C48—C49—H49	119.5
O5—Al1—Na2	47.00 (17)	O23—C50—N8	118.7 (7)
O7—Al1—Na2	141.20 (18)	O23—C50—C51	121.9 (11)
O4—Al1—Na2	88.82 (17)	N8—C50—C51	119.2 (11)
N1—Al1—Na2	125.8 (2)	C56—C51—C52	118.9 (14)
O3—Al1—Dy1	139.2 (2)	C56—C51—C50	117.6 (15)
O8—Al1—Dy1	117.88 (17)	C52—C51—C50	123.4 (15)
O5—Al1—Dy1	110.74 (17)	O24—C52—C51	123.5 (16)
O7—Al1—Dy1	43.19 (14)	O24—C52—C53	118.2 (15)
O4—Al1—Dy1	45.72 (15)	C51—C52—C53	118.2 (15)
N1—Al1—Dy1	64.88 (18)	C54—C53—C52	120.9 (16)
Na2—Al1—Dy1	134.46 (9)	C54—C53—H53	119.5
O6—Al2—O11	100.3 (2)	C52—C53—H53	119.5
O6—Al2—O56	86.8 (2)	C55—C54—C53	120.6 (14)
O11—Al2—O56	92.8 (2)	C55—C54—H54	119.7
O6—Al2—O10	176.4 (2)	C53—C54—H54	119.7
O11—Al2—O10	82.9 (2)	C54—C55—C56	118.7 (15)
O56—Al2—O10	91.3 (2)	C54—C55—H55	120.6
O6—Al2—O13	100.9 (2)	C56—C55—H55	120.6
O11—Al2—O13	158.4 (2)	C55—C56—C51	122.4 (15)
O56—Al2—O13	92.6 (2)	C55—C56—H56	118.8
O10—Al2—O13	76.1 (2)	C51—C56—H56	118.8
O6—Al2—N2	89.8 (2)	O23—C50B—N8	118.7 (7)
O11—Al2—N2	90.2 (2)	O23—C50B—C51B	119.8 (14)
O56—Al2—N2	175.9 (3)	N8—C50B—C51B	121.2 (13)
O10—Al2—N2	91.9 (2)	C56B—C51B—C52B	119.3 (17)
O13—Al2—N2	85.7 (2)	C56B—C51B—C50B	119.6 (19)
O6—Al2—Dy1	135.70 (18)	C52B—C51B—C50B	121.2 (19)
O11—Al2—Dy1	114.44 (18)	O24—C52B—C53B	117.0 (19)
O56—Al2—Dy1	116.59 (18)	O24—C52B—C51B	125 (2)
O10—Al2—Dy1	42.99 (15)	C53B—C52B—C51B	118.1 (19)
O13—Al2—Dy1	45.13 (16)	C52B—C53B—C54B	121.8 (19)
N2—Al2—Dy1	64.57 (17)	C52B—C53B—H53B	119.1
O6—Al2—Na5	45.66 (17)	C54B—C53B—H53B	119.1
O11—Al2—Na5	112.73 (19)	C55B—C54B—C53B	119.5 (17)
O56—Al2—Na5	44.63 (17)	C55B—C54B—H54B	120.2
O10—Al2—Na5	131.51 (18)	C53B—C54B—H54B	120.2
O13—Al2—Na5	85.43 (18)	C56B—C55B—C54B	118.8 (18)
N2—Al2—Na5	131.39 (19)	C56B—C55B—H55B	120.6
Dy1—Al2—Na5	129.76 (8)	C54B—C55B—H55B	120.6
O6—Al2—Na4	105.71 (19)	C55B—C56B—C51B	122.5 (18)
O11—Al2—Na4	131.26 (19)	C55B—C56B—H56B	118.8
O56—Al2—Na4	49.34 (18)	C51B—C56B—H56B	118.8
O10—Al2—Na4	70.78 (17)	N9—C57—O26	119.0 (7)
O13—Al2—Na4	44.88 (16)	N9—C57—C58	118.0 (7)
N2—Al2—Na4	129.73 (19)	O26—C57—C58	122.9 (7)
Dy1—Al2—Na4	71.70 (6)	C63—C58—C59	119.7 (9)
Na5—Al2—Na4	64.72 (8)	C63—C58—C57	118.8 (8)
O12—Al3—O18	115.8 (3)	C59—C58—C57	121.4 (7)
O12—Al3—O20	91.9 (3)	O27—C59—C60	118.8 (8)
O18—Al3—O20	97.8 (3)	O27—C59—C58	123.2 (8)
O12—Al3—O19	130.4 (3)	C60—C59—C58	117.9 (8)
O18—Al3—O19	113.8 (3)	C61—C60—C59	122.0 (10)
O20—Al3—O19	82.7 (3)	C61—C60—H60	119.0
O12—Al3—N4	89.9 (3)	C59—C60—H60	119.0
O18—Al3—N4	93.7 (3)	C60—C61—C62	120.3 (10)
O20—Al3—N4	166.2 (3)	C60—C61—H61	119.9
O19—Al3—N4	85.7 (2)	C62—C61—H61	119.9
O15—Al4—O9	111.8 (3)	C61—C62—C63	118.8 (10)
O15—Al4—O23	101.1 (3)	C61—C62—H62	120.6
O9—Al4—O23	92.8 (3)	C63—C62—H62	120.6
O15—Al4—O22	107.0 (3)	C62—C63—C58	121.2 (10)
O9—Al4—O22	141.0 (3)	C62—C63—H63	119.4
O23—Al4—O22	83.2 (2)	C58—C63—H63	119.4
O15—Al4—N3	92.5 (3)	O29—C64—N10	119.9 (7)
O9—Al4—N3	90.0 (3)	O29—C64—C65	119.5 (7)
O23—Al4—N3	163.9 (3)	N10—C64—C65	120.5 (8)
O22—Al4—N3	84.7 (2)	C66—C65—C70	118.8 (8)
O24—Al5—O25	171.5 (3)	C66—C65—C64	122.5 (8)
O24—Al5—O17	87.4 (2)	C70—C65—C64	118.6 (8)
O25—Al5—O17	92.1 (2)	O30—C66—C65	123.6 (7)
O24—Al5—O26	90.1 (2)	O30—C66—C67	117.4 (8)
O25—Al5—O26	81.5 (2)	C65—C66—C67	118.9 (8)
O17—Al5—O26	93.7 (3)	C68—C67—C66	121.0 (10)
O24—Al5—O16	109.2 (3)	C68—C67—H67	119.5
O25—Al5—O16	79.1 (2)	C66—C67—H67	119.5
O17—Al5—O16	82.7 (2)	C69—C68—C67	120.0 (9)
O26—Al5—O16	160.1 (3)	C69—C68—H68	120.0
O24—Al5—N8	89.6 (3)	C67—C68—H68	120.0
O25—Al5—N8	92.7 (2)	C70—C69—C68	119.4 (10)
O17—Al5—N8	167.4 (3)	C70—C69—H69	120.3
O26—Al5—N8	98.5 (3)	C68—C69—H69	120.3
O16—Al5—N8	86.8 (2)	C69—C70—C65	121.7 (10)
O24—Al5—Na3	56.35 (19)	C69—C70—H70	119.1
O25—Al5—Na3	116.83 (18)	C65—C70—H70	119.1
O17—Al5—Na3	55.17 (18)	O32—C71—N11	117.4 (7)
O26—Al5—Na3	53.87 (18)	O32—C71—C72	119.9 (7)
O16—Al5—Na3	133.63 (19)	N11—C71—C72	122.7 (7)
N8—Al5—Na3	131.6 (2)	C73—C72—C77	121.3 (8)
O24—Al5—Dy1	149.0 (2)	C73—C72—C71	120.1 (7)
O25—Al5—Dy1	38.38 (15)	C77—C72—C71	118.1 (8)
O17—Al5—Dy1	109.69 (18)	O33—C73—C72	122.3 (8)
O26—Al5—Dy1	113.39 (18)	O33—C73—C74	121.2 (8)
O16—Al5—Dy1	50.92 (16)	C72—C73—C74	116.5 (8)
N8—Al5—Dy1	68.02 (18)	C75—C74—C73	121.1 (9)
Na3—Al5—Dy1	154.37 (10)	C75—C74—H74	119.4
O21—Al6—O27	85.1 (3)	C73—C74—H74	119.4
O21—Al6—O2	90.7 (3)	C76—C75—C74	120.7 (9)
O27—Al6—O2	91.6 (3)	C76—C75—H75	119.7
O21—Al6—O1	170.2 (3)	C74—C75—H75	119.7
O27—Al6—O1	101.1 (3)	C77—C76—C75	119.3 (10)
O2—Al6—O1	81.6 (2)	C77—C76—H76	120.4
O21—Al6—N9	104.1 (3)	C75—C76—H76	120.4
O27—Al6—N9	86.2 (3)	C76—C77—C72	120.9 (10)
O2—Al6—N9	164.8 (3)	C76—C77—H77	119.5
O1—Al6—N9	84.0 (3)	C72—C77—H77	119.5
O21—Al6—N7	86.7 (3)	N12—C78—O35	118.6 (7)
O27—Al6—N7	168.7 (3)	N12—C78—C79	119.4 (7)
O2—Al6—N7	96.4 (3)	O35—C78—C79	122.0 (7)
O1—Al6—N7	88.1 (2)	C84—C79—C80	120.4 (9)
N9—Al6—N7	88.1 (3)	C84—C79—C78	119.1 (8)
O21—Al6—Na1	50.6 (2)	C80—C79—C78	120.3 (7)
O27—Al6—Na1	57.38 (19)	O36—C80—C81	118.5 (8)
O2—Al6—Na1	54.60 (18)	O36—C80—C79	123.7 (8)
O1—Al6—Na1	126.85 (19)	C81—C80—C79	117.8 (8)
N9—Al6—Na1	133.8 (2)	C82—C81—C80	121.0 (9)
N7—Al6—Na1	121.8 (2)	C82—C81—H81	119.5
O30—Al7—O34	170.9 (3)	C80—C81—H81	119.5
O30—Al7—O32	86.2 (3)	C83—C82—C81	120.3 (9)
O34—Al7—O32	93.1 (3)	C83—C82—H82	119.8
O30—Al7—O35	89.6 (3)	C81—C82—H82	119.8
O34—Al7—O35	81.4 (2)	C82—C83—C84	120.7 (9)
O32—Al7—O35	93.3 (2)	C82—C83—H83	119.6
O30—Al7—N10	89.7 (3)	C84—C83—H83	119.6
O34—Al7—N10	93.1 (3)	C79—C84—C83	119.7 (10)
O32—Al7—N10	166.1 (3)	C79—C84—H84	120.2
O35—Al7—N10	99.9 (3)	C83—C84—H84	120.2
O30—Al7—O31	109.6 (3)	O38—C85—N13	120.7 (7)
O34—Al7—O31	79.2 (2)	O38—C85—C86	120.1 (7)
O32—Al7—O31	81.9 (2)	N13—C85—C86	119.2 (7)
O35—Al7—O31	159.7 (3)	C91—C86—C87	120.9 (7)
N10—Al7—O31	87.0 (3)	C91—C86—C85	118.0 (7)
O30—Al7—Na8	57.50 (19)	C87—C86—C85	121.1 (7)
O34—Al7—Na8	115.21 (19)	O39—C87—C86	124.5 (7)
O32—Al7—Na8	52.86 (17)	O39—C87—C88	118.7 (8)
O35—Al7—Na8	53.75 (17)	C86—C87—C88	116.9 (8)
N10—Al7—Na8	133.9 (2)	C89—C88—C87	121.8 (9)
O31—Al7—Na8	131.61 (19)	C89—C88—H88	119.1
O30—Al7—Dy2	150.1 (2)	C87—C88—H88	119.1
O34—Al7—Dy2	38.04 (16)	C88—C89—C90	120.6 (8)
O32—Al7—Dy2	109.42 (18)	C88—C89—H89	119.7
O35—Al7—Dy2	113.80 (18)	C90—C89—H89	119.7
N10—Al7—Dy2	68.98 (19)	C89—C90—C91	118.6 (8)
O31—Al7—Dy2	50.93 (16)	C89—C90—H90	120.7
Na8—Al7—Dy2	151.78 (10)	C91—C90—H90	120.7
O42—Al8—O39	110.1 (3)	C86—C91—C90	121.3 (8)
O42—Al8—O29	99.1 (3)	C86—C91—H91	119.4
O39—Al8—O29	89.5 (3)	C90—C91—H91	119.4
O42—Al8—O28	108.2 (3)	O41—C92—N14	123.8 (7)
O39—Al8—O28	141.6 (3)	O41—C92—C93	123.6 (7)
O29—Al8—O28	82.7 (2)	N14—C92—C93	112.6 (7)
O42—Al8—N13	103.6 (3)	O41—C92—Na7	58.0 (4)
O39—Al8—N13	89.3 (3)	N14—C92—Na7	76.6 (4)
O29—Al8—N13	156.2 (3)	C93—C92—Na7	146.1 (5)
O28—Al8—N13	83.6 (2)	C98—C93—C94	120.9 (8)
O51—Al9—O36	85.3 (2)	C98—C93—C92	118.5 (7)
O51—Al9—O44	91.9 (3)	C94—C93—C92	120.6 (7)
O36—Al9—O44	91.4 (3)	O42—C94—C95	120.4 (8)
O51—Al9—O43	170.8 (3)	O42—C94—C93	122.2 (7)
O36—Al9—O43	101.5 (2)	C95—C94—C93	117.3 (8)
O44—Al9—O43	81.7 (2)	C96—C95—C94	120.9 (9)
O51—Al9—N12	103.2 (3)	C96—C95—H95	119.6
O36—Al9—N12	85.9 (3)	C94—C95—H95	119.6
O44—Al9—N12	164.3 (3)	C95—C96—C97	121.4 (9)
O43—Al9—N12	83.7 (2)	C95—C96—H96	119.3
O51—Al9—N17	86.1 (3)	C97—C96—H96	119.3
O36—Al9—N17	168.7 (3)	C98—C97—C96	118.4 (9)
O44—Al9—N17	96.2 (3)	C98—C97—H97	120.8
O43—Al9—N17	87.9 (2)	C96—C97—H97	120.8
N12—Al9—N17	89.0 (3)	C97—C98—C93	121.0 (9)
O51—Al9—Na10	51.6 (2)	C97—C98—H98	119.5
O36—Al9—Na10	55.48 (19)	C93—C98—H98	119.5
O44—Al9—Na10	56.23 (18)	O44—C99—N15	120.3 (7)
O43—Al9—Na10	127.64 (19)	O44—C99—C100	119.0 (7)
N12—Al9—Na10	131.7 (2)	N15—C99—C100	120.7 (7)
N17—Al9—Na10	123.0 (2)	C105—C100—C101	120.4 (8)
O48—Al10—O33	111.9 (3)	C105—C100—C99	118.3 (8)
O48—Al10—O50	90.1 (3)	C101—C100—C99	121.3 (8)
O33—Al10—O50	102.6 (3)	O45—C101—C102	117.0 (8)
O48—Al10—O49	137.1 (3)	O45—C101—C100	125.0 (8)
O33—Al10—O49	110.9 (3)	C102—C101—C100	118.1 (9)
O50—Al10—O49	83.1 (3)	C101—C102—C103	120.8 (10)
O48—Al10—N16	90.0 (3)	C101—C102—H102	119.6
O33—Al10—N16	92.7 (3)	C103—C102—H102	119.6
O50—Al10—N16	163.5 (3)	C104—C103—C102	120.3 (10)
O49—Al10—N16	85.6 (3)	C104—C103—H103	119.8
O54—Al11—O47	96.7 (2)	C102—C103—H103	119.8
O54—Al11—O58	86.7 (2)	C103—C104—C105	119.2 (10)
O47—Al11—O58	96.2 (2)	C103—C104—H104	120.4
O54—Al11—O46	178.9 (3)	C105—C104—H104	120.4
O47—Al11—O46	83.0 (2)	C104—C105—C100	121.2 (10)
O58—Al11—O46	92.3 (2)	C104—C105—H105	119.4
O54—Al11—O40	103.1 (2)	C100—C105—H105	119.4
O47—Al11—O40	159.8 (2)	O47—C106—N16	121.1 (7)
O58—Al11—O40	88.7 (2)	O47—C106—C107	119.6 (7)
O46—Al11—O40	77.2 (2)	N16—C106—C107	119.2 (7)
O54—Al11—N18	90.2 (2)	C112—C107—C108	120.0 (7)
O47—Al11—N18	92.1 (2)	C112—C107—C106	118.2 (8)
O58—Al11—N18	171.4 (3)	C108—C107—C106	121.7 (7)
O46—Al11—N18	90.9 (2)	O48—C108—C109	118.2 (8)
O40—Al11—N18	84.2 (2)	O48—C108—C107	123.8 (7)
O54—Al11—Dy2	138.31 (19)	C109—C108—C107	117.9 (8)
O47—Al11—Dy2	116.10 (18)	C110—C109—C108	120.9 (8)
O58—Al11—Dy2	112.76 (18)	C110—C109—H109	119.5
O46—Al11—Dy2	42.54 (14)	C108—C109—H109	119.5
O40—Al11—Dy2	44.69 (15)	C109—C110—C111	121.1 (8)
N18—Al11—Dy2	65.02 (17)	C109—C110—H110	119.5
O54—Al11—Na6	48.14 (18)	C111—C110—H110	119.5
O47—Al11—Na6	109.93 (19)	C112—C111—C110	118.7 (8)
O58—Al11—Na6	40.76 (17)	C112—C111—H111	120.6
O46—Al11—Na6	130.93 (18)	C110—C111—H111	120.6
O40—Al11—Na6	86.50 (17)	C111—C112—C107	121.3 (8)
N18—Al11—Na6	133.5 (2)	C111—C112—H112	119.3
Dy2—Al11—Na6	129.55 (8)	C107—C112—H112	119.3
O54—Al11—Na7	101.74 (19)	N17—C113—O50	120.1 (7)
O47—Al11—Na7	141.07 (18)	N17—C113—C114	122.1 (7)
O58—Al11—Na7	51.60 (18)	O50—C113—C114	117.8 (7)
O46—Al11—Na7	77.85 (17)	C119—C114—C115	120.5 (8)
O40—Al11—Na7	37.36 (16)	C119—C114—C113	118.9 (7)
N18—Al11—Na7	121.57 (19)	C115—C114—C113	120.6 (7)
Dy2—Al11—Na7	68.99 (7)	O51—C115—C116	119.6 (7)
Na6—Al11—Na7	62.39 (8)	O51—C115—C114	123.4 (7)
O45—Al12—O38	94.0 (2)	C116—C115—C114	117.0 (7)
O45—Al12—O53	88.2 (2)	O51—C115—Na10	45.1 (4)
O38—Al12—O53	97.1 (2)	C116—C115—Na10	88.3 (5)
O45—Al12—O37	174.5 (3)	C114—C115—Na10	140.0 (6)
O38—Al12—O37	80.7 (2)	C117—C116—C115	120.9 (8)
O53—Al12—O37	94.0 (2)	C117—C116—H116	119.6
O45—Al12—O52	107.2 (2)	C115—C116—H116	119.6
O38—Al12—O52	158.7 (2)	C118—C117—C116	120.9 (8)
O53—Al12—O52	82.2 (2)	C118—C117—H117	119.6
O37—Al12—O52	78.1 (2)	C116—C117—H117	119.6
O45—Al12—N15	88.0 (2)	C117—C118—C119	119.2 (8)
O38—Al12—N15	96.4 (3)	C117—C118—H118	120.4
O53—Al12—N15	166.2 (3)	C119—C118—H118	120.4
O37—Al12—N15	91.0 (2)	C114—C119—C118	121.5 (9)
O52—Al12—N15	86.3 (2)	C114—C119—H119	119.2
O45—Al12—Na9^i^	43.41 (18)	C118—C119—H119	119.2
O38—Al12—Na9^i^	104.03 (18)	O53—C120—N18	121.0 (7)
O53—Al12—Na9^i^	45.47 (17)	O53—C120—C121	118.8 (6)
O37—Al12—Na9^i^	139.37 (19)	N18—C120—C121	120.2 (6)
O52—Al12—Na9^i^	90.68 (16)	C122—C121—C126	120.0 (7)
N15—Al12—Na9^i^	127.6 (2)	C122—C121—C120	122.7 (6)
O45—Al12—Dy2	139.7 (2)	C126—C121—C120	117.2 (7)
O38—Al12—Dy2	116.73 (17)	O54—C122—C121	125.2 (7)
O53—Al12—Dy2	111.37 (17)	O54—C122—C123	116.4 (7)
O37—Al12—Dy2	43.58 (14)	C121—C122—C123	118.4 (7)
O52—Al12—Dy2	45.95 (14)	C124—C123—C122	120.9 (8)
N15—Al12—Dy2	64.52 (18)	C124—C123—H123	119.6
Na9^i^—Al12—Dy2	136.39 (9)	C122—C123—H123	119.6
O75^ii^—Na1—O21	109.8 (3)	C125—C124—C123	119.7 (8)
O75^ii^—Na1—O59	82.6 (2)	C125—C124—H124	120.2
O21—Na1—O59	151.1 (3)	C123—C124—H124	120.2
O75^ii^—Na1—O60B	97.9 (3)	C126—C125—C124	120.1 (7)
O21—Na1—O60B	94.7 (3)	C126—C125—H125	120.0
O59—Na1—O60B	109.8 (3)	C124—C125—H125	120.0
O75^ii^—Na1—O60	97.9 (3)	C125—C126—C121	120.9 (8)
O21—Na1—O60	94.7 (3)	C125—C126—H126	119.6
O59—Na1—O60	109.8 (3)	C121—C126—H126	119.6
O75^ii^—Na1—O2	175.3 (3)	O55—C127—O56	123.2 (7)
O21—Na1—O2	66.2 (2)	O55—C127—C128	118.5 (7)
O59—Na1—O2	99.8 (2)	O56—C127—C128	118.2 (7)
O60B—Na1—O2	85.2 (2)	O55—C127—Na4	54.8 (4)
O60—Na1—O2	85.2 (2)	O56—C127—Na4	69.0 (4)
O75^ii^—Na1—O27	112.0 (3)	C128—C127—Na4	171.2 (7)
O21—Na1—O27	60.8 (2)	C127—C128—H12A	109.5
O59—Na1—O27	90.5 (2)	C127—C128—H12B	109.5
O60B—Na1—O27	145.9 (2)	H12A—C128—H12B	109.5
O60—Na1—O27	145.9 (2)	C127—C128—H12C	109.5
O2—Na1—O27	64.02 (19)	H12A—C128—H12C	109.5
O75^ii^—Na1—Al6	137.1 (2)	H12B—C128—H12C	109.5
O21—Na1—Al6	37.07 (15)	O57—C129—O58	121.7 (8)
O59—Na1—Al6	117.0 (2)	O57—C129—C130	120.0 (7)
O60B—Na1—Al6	108.8 (2)	O58—C129—C130	118.3 (7)
O60—Na1—Al6	108.8 (2)	O57—C129—Na7	53.2 (4)
O2—Na1—Al6	38.17 (13)	O58—C129—Na7	73.3 (5)
O27—Na1—Al6	37.45 (14)	C130—C129—Na7	156.0 (6)
O75^ii^—Na1—Na10^ii^	41.3 (2)	C129—C130—H13A	109.5
O21—Na1—Na10^ii^	144.92 (19)	C129—C130—H13B	109.5
O59—Na1—Na10^ii^	41.30 (16)	H13A—C130—H13B	109.5
O60—Na1—Na10^ii^	107.0 (2)	C129—C130—H13C	109.5
O2—Na1—Na10^ii^	141.09 (19)	H13A—C130—H13C	109.5
O27—Na1—Na10^ii^	106.20 (17)	H13B—C130—H13C	109.5
Al6—Na1—Na10^ii^	143.53 (13)	C131—O59—Na10^ii^	142.7 (8)
O63B—Na2—O3	165.3 (3)	C131—O59—Na1	117.2 (8)
O63—Na2—O3	165.3 (3)	Na10^ii^—O59—Na1	97.1 (3)
O63B—Na2—O62	78.5 (2)	O59—C131—N19	131.8 (13)
O63—Na2—O62	78.5 (2)	O59—C131—H131	114.1
O3—Na2—O62	116.2 (3)	N19—C131—H131	114.1
O63B—Na2—O61	99.6 (3)	C131—N19—C133	130.4 (13)
O63—Na2—O61	99.6 (3)	C131—N19—C132	117.3 (12)
O3—Na2—O61	84.3 (2)	C133—N19—C132	110.9 (12)
O62—Na2—O61	84.5 (3)	N19—C132—H13D	109.5
O63B—Na2—O64B	83.4 (14)	N19—C132—H13E	109.5
O3—Na2—O64B	97.8 (13)	H13D—C132—H13E	109.5
O62—Na2—O64B	77 (2)	N19—C132—H13F	109.5
O61—Na2—O64B	160 (2)	H13D—C132—H13F	109.5
O63—Na2—O64	81.2 (7)	H13E—C132—H13F	109.5
O3—Na2—O64	99.8 (7)	N19—C133—H13G	109.5
O62—Na2—O64	76.8 (10)	N19—C133—H13H	109.5
O61—Na2—O64	160.8 (11)	H13G—C133—H13H	109.5
O63B—Na2—O5	100.4 (2)	N19—C133—H13I	109.5
O63—Na2—O5	100.4 (2)	H13G—C133—H13I	109.5
O3—Na2—O5	65.0 (2)	H13H—C133—H13I	109.5
O62—Na2—O5	165.2 (3)	C134—O60—Na1	124.1 (7)
O61—Na2—O5	110.1 (2)	O60—C134—N20	126.8 (12)
O64B—Na2—O5	88 (2)	O60—C134—H134	116.6
O64—Na2—O5	88.4 (10)	N20—C134—H134	116.6
O63B—Na2—C203	25.5 (8)	C134—N20—C136	127.0 (13)
O3—Na2—C203	143.4 (7)	C134—N20—C135	115.3 (12)
O62—Na2—C203	97.2 (7)	C136—N20—C135	116.5 (13)
O61—Na2—C203	84.3 (8)	N20—C135—H13J	109.5
O64B—Na2—C203	104.5 (17)	N20—C135—H13K	109.5
O5—Na2—C203	86.7 (6)	H13J—C135—H13K	109.5
O63—Na2—Na3^iii^	47.51 (17)	N20—C135—H13L	109.5
O3—Na2—Na3^iii^	141.2 (2)	H13J—C135—H13L	109.5
O62—Na2—Na3^iii^	47.51 (17)	H13K—C135—H13L	109.5
O61—Na2—Na3^iii^	121.2 (2)	N20—C136—H13M	109.5
O64—Na2—Na3^iii^	46.6 (7)	N20—C136—H13N	109.5
O5—Na2—Na3^iii^	121.42 (19)	H13M—C136—H13N	109.5
O63B—Na2—Al1	133.8 (2)	N20—C136—H13O	109.5
O63—Na2—Al1	133.8 (2)	H13M—C136—H13O	109.5
O3—Na2—Al1	31.44 (14)	H13N—C136—H13O	109.5
O62—Na2—Al1	147.3 (2)	C224—O60B—Na1	140 (3)
O61—Na2—Al1	92.94 (19)	O60B—C224—N20B	132 (4)
O64B—Na2—Al1	99.0 (16)	O60B—C224—H224	114.1
O64—Na2—Al1	100.3 (8)	N20B—C224—H224	114.1
O5—Na2—Al1	34.35 (13)	C224—N20B—C226	123 (3)
C203—Na2—Al1	115.0 (7)	C224—N20B—C225	121 (3)
Na3^iii^—Na2—Al1	145.85 (14)	C226—N20B—C225	115 (3)
O64^i^—Na3—O63^i^	85.9 (11)	N20B—C225—H22J	109.5
O64^i^—Na3—O62^i^	81.9 (11)	N20B—C225—H22K	109.5
O63^i^—Na3—O62^i^	80.6 (3)	H22J—C225—H22K	109.5
O64^i^—Na3—O26	109.8 (10)	N20B—C225—H22L	109.5
O63^i^—Na3—O26	161.6 (3)	H22J—C225—H22L	109.5
O62^i^—Na3—O26	110.3 (3)	H22K—C225—H22L	109.5
O64^i^—Na3—O17	174.6 (11)	N20B—C226—H22M	109.5
O63^i^—Na3—O17	93.4 (2)	N20B—C226—H22N	109.5
O62^i^—Na3—O17	103.2 (2)	H22M—C226—H22N	109.5
O26—Na3—O17	70.07 (19)	N20B—C226—H22O	109.5
O64^i^—Na3—O24	112.0 (11)	H22M—C226—H22O	109.5
O63^i^—Na3—O24	100.6 (2)	H22N—C226—H22O	109.5
O62^i^—Na3—O24	166.0 (3)	C137—O61—Na2	117.4 (6)
O26—Na3—O24	65.2 (2)	O61—C137—N21	125.7 (9)
O17—Na3—O24	62.82 (19)	O61—C137—H137	117.1
O64^i^—Na3—Al5	137.1 (10)	N21—C137—H137	117.1
O63^i^—Na3—Al5	121.8 (2)	C137—N21—C138	123.7 (9)
O62^i^—Na3—Al5	130.8 (2)	C137—N21—C139	122.6 (8)
O26—Na3—Al5	39.90 (14)	C138—N21—C139	113.2 (9)
O17—Na3—Al5	39.79 (13)	N21—C138—H13P	109.5
O24—Na3—Al5	37.34 (13)	N21—C138—H13Q	109.5
O64^i^—Na3—Na2^i^	51.5 (10)	H13P—C138—H13Q	109.5
O63^i^—Na3—Na2^i^	48.36 (17)	N21—C138—H13R	109.5
O62^i^—Na3—Na2^i^	49.53 (18)	H13P—C138—H13R	109.5
O26—Na3—Na2^i^	149.7 (2)	H13Q—C138—H13R	109.5
O17—Na3—Na2^i^	130.99 (19)	N21—C139—H13S	109.5
O24—Na3—Na2^i^	140.05 (19)	N21—C139—H13T	109.5
Al5—Na3—Na2^i^	169.50 (15)	H13S—C139—H13T	109.5
O14—Na4—O65	105.1 (2)	N21—C139—H13U	109.5
O14—Na4—O55	120.3 (2)	H13S—C139—H13U	109.5
O65—Na4—O55	83.0 (2)	H13T—C139—H13U	109.5
O14—Na4—O16	128.6 (2)	C140—O62—Na3^iii^	141.3 (6)
O65—Na4—O16	103.3 (2)	C140—O62—Na2	122.4 (6)
O55—Na4—O16	104.9 (2)	Na3^iii^—O62—Na2	83.0 (2)
O14—Na4—O13	67.51 (18)	O62—C140—N22	125.9 (9)
O65—Na4—O13	167.0 (2)	O62—C140—H140	117.0
O55—Na4—O13	109.9 (2)	N22—C140—H140	117.0
O16—Na4—O13	75.54 (19)	C140—N22—C141	120.1 (9)
O14—Na4—O56	79.99 (19)	C140—N22—C142	124.5 (10)
O65—Na4—O56	126.0 (2)	C141—N22—C142	114.7 (10)
O55—Na4—O56	51.94 (18)	N22—C141—H14A	109.5
O16—Na4—O56	114.9 (2)	N22—C141—H14B	109.5
O13—Na4—O56	64.62 (17)	H14A—C141—H14B	109.5
O14—Na4—C127	102.6 (2)	N22—C141—H14C	109.5
O65—Na4—C127	105.3 (2)	H14A—C141—H14C	109.5
O55—Na4—C127	25.1 (2)	H14B—C141—H14C	109.5
O16—Na4—C127	110.0 (2)	N22—C142—H14D	109.5
O13—Na4—C127	87.0 (2)	N22—C142—H14E	109.5
O56—Na4—C127	26.95 (19)	H14D—C142—H14E	109.5
O14—Na4—C29	23.5 (2)	N22—C142—H14F	109.5
O65—Na4—C29	119.7 (2)	H14D—C142—H14F	109.5
O55—Na4—C29	135.8 (2)	H14E—C142—H14F	109.5
O16—Na4—C29	105.4 (2)	C143—O63—Na3^iii^	122.8 (6)
O13—Na4—C29	49.84 (18)	C143—O63—Na2	152.4 (7)
O56—Na4—C29	86.14 (19)	Na3^iii^—O63—Na2	84.1 (2)
C127—Na4—C29	112.6 (2)	O63—C143—N23	125.8 (10)
O14—Na4—N6	152.8 (2)	O63—C143—H143	117.1
O65—Na4—N6	96.9 (2)	N23—C143—H143	117.1
O55—Na4—N6	77.6 (2)	C143—N23—C144	121.6 (11)
O16—Na4—N6	27.41 (17)	C143—N23—C145	119.5 (11)
O13—Na4—N6	87.71 (19)	C144—N23—C145	118.5 (11)
O56—Na4—N6	100.03 (19)	N23—C144—H14G	109.5
C127—Na4—N6	86.5 (2)	N23—C144—H14H	109.5
C29—Na4—N6	129.5 (2)	H14G—C144—H14H	109.5
O14—Na4—Al2	76.59 (15)	N23—C144—H14I	109.5
O65—Na4—Al2	159.0 (2)	H14G—C144—H14I	109.5
O55—Na4—Al2	78.36 (16)	H14H—C144—H14I	109.5
O16—Na4—Al2	90.97 (15)	N23—C145—H14J	109.5
O13—Na4—Al2	32.52 (12)	N23—C145—H14K	109.5
O56—Na4—Al2	33.08 (12)	H14J—C145—H14K	109.5
C127—Na4—Al2	54.60 (16)	N23—C145—H14L	109.5
C29—Na4—Al2	69.78 (15)	H14J—C145—H14L	109.5
N6—Na4—Al2	88.62 (15)	H14K—C145—H14L	109.5
O14—Na4—Na6	118.61 (17)	C203—O63B—Na2	107.3 (17)
O65—Na4—Na6	42.90 (16)	Na3^iii^—O63B—Na2	84.1 (2)
O55—Na4—Na6	40.24 (16)	N23B—C203—O63B	118 (4)
O16—Na4—Na6	111.42 (16)	N23B—C203—Na2	165 (4)
O13—Na4—Na6	149.79 (16)	O63B—C203—Na2	47.2 (14)
O56—Na4—Na6	86.63 (14)	N23B—C203—H203	121.1
C127—Na4—Na6	62.85 (17)	O63B—C203—H203	121.1
C29—Na4—Na6	142.01 (18)	Na2—C203—H203	74.0
N6—Na4—Na6	88.45 (15)	C203—N23B—C205	125 (5)
Al2—Na4—Na6	117.44 (10)	C203—N23B—C204	128 (5)
O14—Na4—Na5	42.54 (14)	C205—N23B—C204	106 (5)
O65—Na4—Na5	110.2 (2)	N23B—C204—H20G	109.5
O55—Na4—Na5	78.67 (17)	N23B—C204—H20H	109.5
O16—Na4—Na5	146.51 (17)	H20G—C204—H20H	109.5
O13—Na4—Na5	72.11 (14)	N23B—C204—H20I	109.5
O56—Na4—Na5	41.49 (13)	H20G—C204—H20I	109.5
C127—Na4—Na5	60.31 (17)	H20H—C204—H20I	109.5
C29—Na4—Na5	58.51 (16)	N23B—C205—H20J	109.5
N6—Na4—Na5	141.18 (17)	N23B—C205—H20K	109.5
Al2—Na4—Na5	56.62 (7)	H20J—C205—H20K	109.5
Na6—Na4—Na5	92.92 (10)	N23B—C205—H20L	109.5
O67—Na5—O57	104.2 (3)	H20J—C205—H20L	109.5
O67—Na5—O66	88.0 (3)	H20K—C205—H20L	109.5
O57—Na5—O66	86.1 (3)	C146—O64—Na3^iii^	154 (3)
O67—Na5—O56	164.2 (3)	C146—O64—Na2	121 (3)
O57—Na5—O56	83.5 (2)	Na3^iii^—O64—Na2	81.9 (6)
O66—Na5—O56	106.4 (3)	O64—C146—N24	125 (2)
O67—Na5—O6	111.1 (3)	O64—C146—H146	117.7
O57—Na5—O6	144.6 (2)	N24—C146—H146	117.7
O66—Na5—O6	93.3 (3)	C146—N24—C147	122.7 (13)
O56—Na5—O6	62.69 (18)	C146—N24—C148	122.3 (12)
O67—Na5—O14	84.8 (3)	C147—N24—C148	114.6 (13)
O57—Na5—O14	95.0 (2)	N24—C147—H14M	109.5
O66—Na5—O14	172.8 (3)	N24—C147—H14O	109.5
O56—Na5—O14	80.8 (2)	H14M—C147—H14O	109.5
O6—Na5—O14	89.90 (19)	N24—C147—H14P	109.5
O67—Na5—Al2	135.6 (2)	H14M—C147—H14P	109.5
O57—Na5—Al2	116.83 (19)	H14O—C147—H14P	109.5
O66—Na5—Al2	109.9 (2)	N24—C148—H14Q	109.5
O56—Na5—Al2	33.44 (13)	N24—C148—H14R	109.5
O6—Na5—Al2	31.47 (13)	H14Q—C148—H14R	109.5
O14—Na5—Al2	75.98 (15)	N24—C148—H14S	109.5
O67—Na5—Na4	121.7 (2)	H14Q—C148—H14S	109.5
O57—Na5—Na4	75.71 (17)	H14R—C148—H14S	109.5
O66—Na5—Na4	148.0 (2)	C230—O64B—Na2	113 (5)
O56—Na5—Na4	46.24 (15)	Na3^iii^—O64B—Na2	81.2 (12)
O6—Na5—Na4	87.06 (15)	O64B—C230—N24B	126 (4)
O14—Na5—Na4	38.65 (13)	O64B—C230—H230	117.2
Al2—Na5—Na4	58.66 (7)	N24B—C230—H230	117.2
O68—Na6—O55	94.9 (3)	C230—N24B—C232	122 (2)
O68—Na6—O41	107.5 (3)	C230—N24B—C231	120 (2)
O55—Na6—O41	97.9 (2)	C232—N24B—C231	116 (2)
O68—Na6—O58	160.0 (3)	N24B—C231—H23A	109.5
O55—Na6—O58	99.0 (2)	N24B—C231—H23B	109.5
O41—Na6—O58	84.8 (2)	H23A—C231—H23B	109.5
O68—Na6—O65	90.1 (3)	N24B—C231—H23C	109.5
O55—Na6—O65	79.7 (2)	H23A—C231—H23C	109.5
O41—Na6—O65	162.4 (2)	H23B—C231—H23C	109.5
O58—Na6—O65	78.4 (2)	N24B—C232—H23D	109.5
O68—Na6—O54	105.7 (2)	N24B—C232—H23E	109.5
O55—Na6—O54	159.3 (2)	H23D—C232—H23E	109.5
O41—Na6—O54	78.4 (2)	N24B—C232—H23F	109.5
O58—Na6—O54	60.43 (18)	H23D—C232—H23F	109.5
O65—Na6—O54	97.6 (2)	H23E—C232—H23F	109.5
O68—Na6—Al11	136.5 (2)	C149—O65—Na4	148.6 (6)
O55—Na6—Al11	128.47 (19)	C149—O65—Na6	113.0 (6)
O41—Na6—Al11	73.47 (16)	Na4—O65—Na6	97.2 (2)
O58—Na6—Al11	30.99 (13)	O65—C149—N25	124.8 (9)
O65—Na6—Al11	94.20 (18)	O65—C149—H149	117.6
O54—Na6—Al11	30.83 (12)	N25—C149—H149	117.6
O68—Na6—Na4	95.6 (2)	C149—N25—C151	121.1 (10)
O55—Na6—Na4	39.90 (16)	C149—N25—C150	119.5 (9)
O41—Na6—Na4	134.24 (19)	C151—N25—C150	119.2 (10)
O58—Na6—Na4	86.11 (16)	N25—C150—H15A	109.5
O65—Na6—Na4	39.88 (15)	N25—C150—H15B	109.5
O54—Na6—Na4	132.85 (16)	H15A—C150—H15B	109.5
Al11—Na6—Na4	114.87 (10)	N25—C150—H15C	109.5
O68—Na6—Na7	147.5 (2)	H15A—C150—H15C	109.5
O55—Na6—Na7	78.06 (18)	H15B—C150—H15C	109.5
O41—Na6—Na7	43.93 (15)	N25—C151—H15D	109.5
O58—Na6—Na7	50.82 (16)	N25—C151—H15E	109.5
O65—Na6—Na7	119.06 (18)	H15D—C151—H15E	109.5
O54—Na6—Na7	85.60 (15)	N25—C151—H15F	109.5
Al11—Na6—Na7	60.47 (8)	H15D—C151—H15F	109.5
Na4—Na6—Na7	98.51 (10)	H15E—C151—H15F	109.5
O69B—Na7—O40	123.7 (15)	C152—O66—Na5	127.6 (8)
O69—Na7—O40	115.9 (4)	O66—C152—N26	125.2 (11)
O69B—Na7—O57	129.3 (13)	O66—C152—H152	117.4
O69—Na7—O57	138.4 (4)	N26—C152—H152	117.4
O40—Na7—O57	105.1 (2)	C152—N26—C153	117.8 (10)
O69B—Na7—O31	111.5 (16)	C152—N26—C154	122.9 (10)
O69—Na7—O31	104.5 (4)	C153—N26—C154	119.3 (10)
O40—Na7—O31	76.98 (19)	N26—C153—H15G	109.5
O57—Na7—O31	90.7 (2)	N26—C153—H15H	109.5
O69B—Na7—O41	79.9 (18)	H15G—C153—H15H	109.5
O69—Na7—O41	81.1 (4)	N26—C153—H15I	109.5
O40—Na7—O41	68.18 (19)	H15G—C153—H15I	109.5
O57—Na7—O41	109.3 (2)	H15H—C153—H15I	109.5
O31—Na7—O41	143.1 (2)	N26—C154—H15J	109.5
O69B—Na7—O58	147.4 (16)	N26—C154—H15K	109.5
O69—Na7—O58	152.8 (4)	H15J—C154—H15K	109.5
O40—Na7—O58	61.10 (17)	N26—C154—H15L	109.5
O57—Na7—O58	49.46 (17)	H15J—C154—H15L	109.5
O31—Na7—O58	101.03 (19)	H15K—C154—H15L	109.5
O41—Na7—O58	72.72 (18)	C155—O67—Na5	133.5 (7)
O69B—Na7—C129	149.2 (12)	O67—C155—N27	125.9 (11)
O69—Na7—C129	159.2 (4)	O67—C155—H155	117.0
O40—Na7—C129	80.8 (2)	N27—C155—H155	117.0
O57—Na7—C129	24.9 (2)	C155—N27—C156	121.3 (10)
O31—Na7—C129	90.8 (2)	C155—N27—C157	120.3 (10)
O41—Na7—C129	95.1 (2)	C156—N27—C157	118.4 (10)
O58—Na7—C129	25.75 (19)	N27—C156—H15M	109.5
O69B—Na7—N14	98.4 (16)	N27—C156—H15N	109.5
O69—Na7—N14	92.2 (4)	H15M—C156—H15N	109.5
O40—Na7—N14	27.20 (17)	N27—C156—H15O	109.5
O57—Na7—N14	125.2 (2)	H15M—C156—H15O	109.5
O31—Na7—N14	95.4 (2)	H15N—C156—H15O	109.5
O41—Na7—N14	47.76 (18)	N27—C157—H15P	109.5
O58—Na7—N14	76.03 (18)	N27—C157—H15Q	109.5
C129—Na7—N14	100.4 (2)	H15P—C157—H15Q	109.5
O69B—Na7—C92	81.6 (17)	N27—C157—H15R	109.5
O69—Na7—C92	78.5 (4)	H15P—C157—H15R	109.5
O40—Na7—C92	51.0 (2)	H15Q—C157—H15R	109.5
O57—Na7—C92	126.9 (2)	C158—O68—Na6	138.1 (7)
O31—Na7—C92	119.6 (2)	O68—C158—N28	124.7 (9)
O41—Na7—C92	24.6 (2)	O68—C158—H158	117.6
O58—Na7—C92	80.8 (2)	N28—C158—H158	117.6
C129—Na7—C92	106.3 (2)	C158—N28—C160	123.6 (9)
N14—Na7—C92	25.6 (2)	C158—N28—C159	119.6 (8)
O69B—Na7—Al11	145.8 (17)	C160—N28—C159	116.6 (9)
O69—Na7—Al11	141.5 (4)	N28—C159—H15S	109.5
O40—Na7—Al11	29.96 (13)	N28—C159—H15T	109.5
O57—Na7—Al11	76.65 (16)	H15S—C159—H15T	109.5
O31—Na7—Al11	86.53 (15)	N28—C159—H15U	109.5
O41—Na7—Al11	69.36 (15)	H15S—C159—H15U	109.5
O58—Na7—Al11	31.29 (11)	H15T—C159—H15U	109.5
C129—Na7—Al11	51.80 (16)	N28—C160—H16A	109.5
N14—Na7—Al11	49.65 (14)	N28—C160—H16B	109.5
C92—Na7—Al11	64.28 (16)	H16A—C160—H16B	109.5
O69B—Na7—Na6	106.8 (16)	N28—C160—H16C	109.5
O69—Na7—Na6	112.6 (4)	H16A—C160—H16C	109.5
O40—Na7—Na6	76.69 (15)	H16B—C160—H16C	109.5
O57—Na7—Na6	69.37 (16)	C161—O69—Na7	125.1 (12)
O31—Na7—Na6	141.14 (17)	O69—C161—N29	129.8 (14)
O41—Na7—Na6	40.01 (14)	O69—C161—H161	115.1
O58—Na7—Na6	40.66 (12)	N29—C161—H161	115.1
C129—Na7—Na6	57.12 (17)	C161—N29—C163	123.1 (12)
N14—Na7—Na6	72.50 (15)	C161—N29—C162	119.8 (11)
C92—Na7—Na6	59.74 (17)	C163—N29—C162	115.8 (10)
Al11—Na7—Na6	57.15 (8)	N29—C162—H16D	109.5
O70B—Na8—O71	81.2 (2)	N29—C162—H16E	109.5
O70—Na8—O71	81.2 (2)	H16D—C162—H16E	109.5
O70B—Na8—O72	87.8 (2)	N29—C162—H16F	109.5
O70—Na8—O72	87.8 (2)	H16D—C162—H16F	109.5
O71—Na8—O72	79.6 (2)	H16E—C162—H16F	109.5
O70B—Na8—O32	174.4 (3)	N29—C163—H16G	109.5
O70—Na8—O32	174.4 (3)	N29—C163—H16H	109.5
O71—Na8—O32	104.3 (2)	H16G—C163—H16H	109.5
O72—Na8—O32	92.7 (2)	N29—C163—H16I	109.5
O70B—Na8—O35	109.0 (2)	H16G—C163—H16I	109.5
O70—Na8—O35	109.0 (2)	H16H—C163—H16I	109.5
O71—Na8—O35	108.4 (2)	C206—O69B—Na7	129 (5)
O72—Na8—O35	162.0 (2)	O69B—C206—N29B	124 (3)
O32—Na8—O35	69.9 (2)	O69B—C206—H206	118.1
O70B—Na8—O30	112.8 (2)	N29B—C206—H206	118.1
O70—Na8—O30	112.8 (2)	C206—N29B—C208	124 (3)
O71—Na8—O30	165.3 (2)	C206—N29B—C207	121 (2)
O72—Na8—O30	104.7 (2)	C208—N29B—C207	114 (2)
O32—Na8—O30	61.8 (2)	N29B—C207—H20M	109.5
O35—Na8—O30	63.61 (19)	N29B—C207—H20N	109.5
O70B—Na8—Al7	137.1 (2)	H20M—C207—H20N	109.5
O70—Na8—Al7	137.1 (2)	N29B—C207—H20O	109.5
O71—Na8—Al7	129.02 (19)	H20M—C207—H20O	109.5
O72—Na8—Al7	123.29 (19)	H20N—C207—H20O	109.5
O32—Na8—Al7	39.13 (14)	N29B—C208—H20P	109.5
O35—Na8—Al7	39.23 (14)	N29B—C208—H20Q	109.5
O30—Na8—Al7	37.00 (13)	H20P—C208—H20Q	109.5
O70B—Na8—Na9	51.80 (17)	N29B—C208—H20R	109.5
O70—Na8—Na9	51.80 (17)	H20P—C208—H20R	109.5
O71—Na8—Na9	48.90 (15)	H20Q—C208—H20R	109.5
O72—Na8—Na9	48.95 (15)	C164—O70—Na8	152.4 (9)
O32—Na8—Na9	131.78 (19)	C164—O70—Na9	121.0 (8)
O35—Na8—Na9	147.7 (2)	Na8—O70—Na9	81.5 (2)
O30—Na8—Na9	143.60 (19)	O70—C164—N30	126.7 (13)
Al7—Na8—Na9	170.72 (14)	O70—C164—H164	116.6
O71—Na9—O72	79.2 (2)	N30—C164—H164	116.6
O71—Na9—O45^iii^	117.8 (2)	C164—N30—C166	123.6 (14)
O72—Na9—O45^iii^	163.0 (2)	C164—N30—C165	121.1 (14)
O71—Na9—O73^iii^	83.7 (2)	C166—N30—C165	115.2 (14)
O72—Na9—O73^iii^	97.0 (2)	N30—C165—H16J	109.5
O45^iii^—Na9—O73^iii^	85.2 (2)	N30—C165—H16K	109.5
O71—Na9—O53^iii^	165.8 (2)	H16J—C165—H16K	109.5
O72—Na9—O53^iii^	98.3 (2)	N30—C165—H16L	109.5
O45^iii^—Na9—O53^iii^	65.41 (19)	H16J—C165—H16L	109.5
O73^iii^—Na9—O53^iii^	110.5 (2)	H16K—C165—H16L	109.5
O71—Na9—O70B	77.4 (2)	N30—C166—H16M	109.5
O72—Na9—O70B	83.5 (2)	N30—C166—H16N	109.5
O45^iii^—Na9—O70B	100.0 (2)	H16M—C166—H16N	109.5
O73^iii^—Na9—O70B	160.6 (3)	N30—C166—H16O	109.5
O53^iii^—Na9—O70B	88.5 (2)	H16M—C166—H16O	109.5
O71—Na9—O70	77.4 (2)	H16N—C166—H16O	109.5
O72—Na9—O70	83.5 (2)	C209—O70B—Na8	143.8 (19)
O45^iii^—Na9—O70	100.0 (2)	C209—O70B—Na9	107.3 (13)
O73^iii^—Na9—O70	160.6 (3)	Na8—O70B—Na9	81.5 (2)
O53^iii^—Na9—O70	88.5 (2)	O70B—C209—N30B	127 (2)
O71—Na9—C209	82.1 (8)	O70B—C209—Na9	50.1 (9)
O72—Na9—C209	106.0 (5)	N30B—C209—Na9	175 (3)
O45^iii^—Na9—C209	78.4 (5)	O70B—C209—H209	116.4
O73^iii^—Na9—C209	150.0 (7)	N30B—C209—H209	116.4
O53^iii^—Na9—C209	85.3 (8)	Na9—C209—H209	66.5
O70B—Na9—C209	22.6 (5)	C209—N30B—C211	131 (2)
O71—Na9—Na8	48.60 (16)	C209—N30B—C210	116 (2)
O72—Na9—Na8	48.67 (15)	C211—N30B—C210	113 (2)
O45^iii^—Na9—Na8	142.66 (19)	N30B—C210—H21A	109.5
O73^iii^—Na9—Na8	120.81 (19)	N30B—C210—H21B	109.5
O53^iii^—Na9—Na8	119.72 (18)	H21A—C210—H21B	109.5
O70B—Na9—Na8	46.74 (15)	N30B—C210—H21C	109.5
O70—Na9—Na8	46.74 (15)	H21A—C210—H21C	109.5
C209—Na9—Na8	66.0 (5)	H21B—C210—H21C	109.5
O71—Na9—Al12^iii^	149.4 (2)	N30B—C211—H21D	109.5
O72—Na9—Al12^iii^	131.08 (19)	N30B—C211—H21E	109.5
O45^iii^—Na9—Al12^iii^	32.10 (13)	H21D—C211—H21E	109.5
O73^iii^—Na9—Al12^iii^	94.91 (17)	N30B—C211—H21F	109.5
O53^iii^—Na9—Al12^iii^	33.79 (13)	H21D—C211—H21F	109.5
O70—Na9—Al12^iii^	99.33 (17)	H21E—C211—H21F	109.5
Na8—Na9—Al12^iii^	144.23 (13)	C167—O71—Na8	142.7 (6)
O74—Na10—O59^iv^	94.0 (4)	C167—O71—Na9	122.5 (5)
O74B—Na10—O75	102 (2)	Na8—O71—Na9	82.5 (2)
O74—Na10—O75	108.4 (4)	O71—C167—N31	125.4 (8)
O59^iv^—Na10—O75	82.5 (3)	O71—C167—H167	117.3
O74B—Na10—O51	97.5 (19)	N31—C167—H167	117.3
O74—Na10—O51	93.1 (4)	C167—N31—C168	120.5 (7)
O59^iv^—Na10—O51	112.2 (3)	C167—N31—C169	123.0 (8)
O75—Na10—O51	153.4 (3)	C168—N31—C169	116.4 (8)
O74B—Na10—O36	144 (2)	N31—C168—H16P	109.5
O74—Na10—O36	145.4 (4)	N31—C168—H16Q	109.5
O59^iv^—Na10—O36	116.5 (2)	H16P—C168—H16Q	109.5
O75—Na10—O36	92.2 (2)	N31—C168—H16R	109.5
O51—Na10—O36	61.6 (2)	H16P—C168—H16R	109.5
O74B—Na10—O44	81 (2)	H16Q—C168—H16R	109.5
O74—Na10—O44	84.5 (4)	N31—C169—H16S	109.5
O59^iv^—Na10—O44	177.2 (3)	N31—C169—H16T	109.5
O75—Na10—O44	100.2 (2)	H16S—C169—H16T	109.5
O51—Na10—O44	65.5 (2)	N31—C169—H16U	109.5
O36—Na10—O44	64.23 (18)	H16S—C169—H16U	109.5
O74B—Na10—Al9	107.9 (19)	H16T—C169—H16U	109.5
O74—Na10—Al9	107.8 (4)	C170—O72—Na8	126.1 (5)
O59^iv^—Na10—Al9	141.0 (2)	C170—O72—Na9	151.3 (5)
O75—Na10—Al9	118.5 (2)	Na8—O72—Na9	82.4 (2)
O51—Na10—Al9	37.00 (14)	O72—C170—N32	126.6 (8)
O36—Na10—Al9	37.86 (14)	O72—C170—H170	116.7
O44—Na10—Al9	38.00 (14)	N32—C170—H170	116.7
O74B—Na10—C179	94 (2)	C170—N32—C172	122.4 (8)
O74—Na10—C179	100.9 (5)	C170—N32—C171	120.8 (7)
O59^iv^—Na10—C179	102.3 (3)	C172—N32—C171	116.8 (8)
O75—Na10—C179	20.7 (3)	N32—C171—H17A	109.5
O51—Na10—C179	141.6 (3)	N32—C171—H17B	109.5
O36—Na10—C179	88.6 (3)	H17A—C171—H17B	109.5
O44—Na10—C179	80.3 (3)	N32—C171—H17C	109.5
Al9—Na10—C179	104.6 (3)	H17A—C171—H17C	109.5
O74B—Na10—C212	18.5 (15)	H17B—C171—H17C	109.5
O59^iv^—Na10—C212	108.3 (9)	N32—C172—H17D	109.5
O75—Na10—C212	118.2 (11)	N32—C172—H17E	109.5
O51—Na10—C212	79.4 (10)	H17D—C172—H17E	109.5
O36—Na10—C212	128.5 (9)	N32—C172—H17F	109.5
O44—Na10—C212	69.8 (9)	H17D—C172—H17F	109.5
Al9—Na10—C212	90.9 (9)	H17E—C172—H17F	109.5
C179—Na10—C212	105.7 (11)	C173—O73—Na9^i^	114.7 (5)
O74B—Na10—C115	78.6 (19)	O73—C173—N33	127.0 (8)
O74—Na10—C115	73.1 (4)	O73—C173—H173	116.5
O59^iv^—Na10—C115	102.8 (3)	N33—C173—H173	116.5
O75—Na10—C115	174.5 (3)	C173—N33—C175	121.7 (8)
O51—Na10—C115	22.5 (2)	C173—N33—C174	121.2 (8)
O36—Na10—C115	84.0 (2)	C175—N33—C174	116.3 (9)
O44—Na10—C115	74.5 (2)	N33—C174—H17G	109.5
Al9—Na10—C115	56.29 (16)	N33—C174—H17H	109.5
C179—Na10—C115	154.5 (3)	H17G—C174—H17H	109.5
C212—Na10—C115	61.9 (10)	N33—C174—H17I	109.5
O74—Na10—Na1^iv^	106.3 (4)	H17G—C174—H17I	109.5
O59^iv^—Na10—Na1^iv^	41.56 (19)	H17H—C174—H17I	109.5
O75—Na10—Na1^iv^	41.02 (18)	N33—C175—H17J	109.5
O51—Na10—Na1^iv^	146.96 (19)	N33—C175—H17K	109.5
O36—Na10—Na1^iv^	107.34 (17)	H17J—C175—H17K	109.5
O44—Na10—Na1^iv^	141.20 (19)	N33—C175—H17L	109.5
Al9—Na10—Na1^iv^	145.02 (13)	H17J—C175—H17L	109.5
C179—Na10—Na1^iv^	61.2 (2)	H17K—C175—H17L	109.5
C115—Na10—Na1^iv^	144.20 (19)	C176—O74—Na10	148.3 (12)
N1—O1—Al6	113.5 (4)	O74—C176—N34	125.7 (13)
N1—O1—Dy1	110.2 (4)	O74—C176—H176	117.2
Al6—O1—Dy1	120.2 (2)	N34—C176—H176	117.2
C1—O2—Al6	113.6 (5)	C176—N34—C177	121.5 (11)
C1—O2—Na1	146.4 (5)	C176—N34—C178	121.4 (11)
Al6—O2—Na1	87.2 (2)	C177—N34—C178	117.0 (11)
C3—O3—Al1	133.2 (5)	N34—C177—H17M	109.5
C3—O3—Na2	120.0 (5)	N34—C177—H17N	109.5
Al1—O3—Na2	105.9 (3)	H17M—C177—H17N	109.5
N2—O4—Al1	108.1 (4)	N34—C177—H17O	109.5
N2—O4—Dy1	103.5 (4)	H17M—C177—H17O	109.5
Al1—O4—Dy1	99.7 (2)	H17N—C177—H17O	109.5
C8—O5—Al1	110.5 (4)	N34—C178—H17P	109.5
C8—O5—Na2	114.1 (4)	N34—C178—H17Q	109.5
Al1—O5—Na2	98.6 (2)	H17P—C178—H17Q	109.5
C10—O6—Al2	132.7 (5)	N34—C178—H17R	109.5
C10—O6—Na5	119.3 (4)	H17P—C178—H17R	109.5
Al2—O6—Na5	102.9 (2)	H17Q—C178—H17R	109.5
N3—O7—Al1	112.7 (4)	C212—O74B—Na10	126 (5)
N3—O7—Dy1	125.1 (4)	O74B—C212—N34B	125 (4)
Al1—O7—Dy1	103.3 (2)	O74B—C212—Na10	35 (4)
C15—O8—Al1	113.1 (5)	N34B—C212—Na10	154 (3)
C17—O9—Al4	130.5 (5)	O74B—C212—H212	117.7
N4—O10—Al2	111.2 (4)	N34B—C212—H212	117.7
N4—O10—Dy1	120.9 (4)	Na10—C212—H212	84.8
Al2—O10—Dy1	103.7 (2)	C212—N34B—C214	122 (3)
C22—O11—Al2	111.6 (5)	C212—N34B—C213	117 (3)
C24—O12—Al3	132.7 (5)	C214—N34B—C213	114 (3)
N5—O13—Al2	120.9 (4)	N34B—C213—H21G	109.5
N5—O13—Dy1	117.9 (4)	N34B—C213—H21H	109.5
Al2—O13—Dy1	101.2 (2)	H21G—C213—H21H	109.5
N5—O13—Na4	104.5 (4)	N34B—C213—H21I	109.5
Al2—O13—Na4	102.6 (2)	H21G—C213—H21I	109.5
Dy1—O13—Na4	108.27 (19)	H21H—C213—H21I	109.5
C29—O14—Na4	108.5 (5)	N34B—C214—H21J	109.5
C29—O14—Na5	119.0 (5)	N34B—C214—H21K	109.5
Na4—O14—Na5	98.8 (2)	H21J—C214—H21K	109.5
C31—O15—Al4	134.0 (5)	N34B—C214—H21L	109.5
N6—O16—Al5	105.5 (4)	H21J—C214—H21L	109.5
N6—O16—Na4	100.5 (4)	H21K—C214—H21L	109.5
Al5—O16—Na4	143.8 (3)	C179—O75—Na1^iv^	143.5 (9)
N6—O16—Dy1	102.5 (4)	C179—O75—Na10	116.1 (8)
Al5—O16—Dy1	94.8 (2)	Na1^iv^—O75—Na10	97.7 (3)
Na4—O16—Dy1	103.83 (19)	O75—C179—N35	128.8 (14)
C36—O17—Al5	110.0 (4)	O75—C179—Na10	43.3 (6)
C36—O17—Na3	164.9 (5)	N35—C179—Na10	120.2 (11)
Al5—O17—Na3	85.0 (2)	O75—C179—H179	115.6
C38—O18—Al3	129.1 (6)	N35—C179—H179	115.6
N7—O19—Al3	113.1 (4)	Na10—C179—H179	105.1
N7—O19—Dy1	116.7 (4)	C179—N35—C181	128.9 (14)
Al3—O19—Dy1	125.3 (3)	C179—N35—C180	118.0 (12)
C43—O20—Al3	114.6 (5)	C181—N35—C180	112.6 (13)
C45—O21—Al6	136.3 (5)	N35—C180—H18A	109.5
C45—O21—Na1	116.2 (5)	N35—C180—H18B	109.5
Al6—O21—Na1	92.3 (3)	H18A—C180—H18B	109.5
N8—O22—Al4	111.6 (4)	N35—C180—H18C	109.5
N8—O22—Dy1	115.6 (3)	H18A—C180—H18C	109.5
Al4—O22—Dy1	132.0 (2)	H18B—C180—H18C	109.5
C50B—O23—Al4	114.2 (5)	N35—C181—H18D	109.5
C50—O23—Al4	114.2 (5)	N35—C181—H18E	109.5
C52—O24—Al5	129.2 (9)	H18D—C181—H18E	109.5
C52B—O24—Al5	130.0 (11)	N35—C181—H18F	109.5
C52—O24—Na3	112.0 (11)	H18D—C181—H18F	109.5
C52B—O24—Na3	118.7 (13)	H18E—C181—H18F	109.5
Al5—O24—Na3	86.3 (2)	O76—C182—N36	117 (3)
N9—O25—Al5	113.6 (4)	O76—C182—H182	121.7
N9—O25—Dy1	120.2 (4)	N36—C182—H182	121.7
Al5—O25—Dy1	111.3 (2)	C182—N36—C183	124 (2)
C57—O26—Al5	112.3 (5)	C182—N36—C184	127 (2)
C57—O26—Na3	138.7 (5)	C183—N36—C184	106.1 (19)
Al5—O26—Na3	86.2 (2)	N36—C183—H18G	109.5
C59—O27—Al6	126.2 (6)	N36—C183—H18H	109.5
C59—O27—Na1	109.5 (5)	H18G—C183—H18H	109.5
Al6—O27—Na1	85.2 (2)	N36—C183—H18I	109.5
N10—O28—Al8	111.1 (4)	H18G—C183—H18I	109.5
N10—O28—Dy2	116.3 (4)	H18H—C183—H18I	109.5
Al8—O28—Dy2	132.5 (2)	N36—C184—H18J	109.5
C64—O29—Al8	113.7 (5)	N36—C184—H18K	109.5
C66—O30—Al7	127.1 (5)	H18J—C184—H18K	109.5
C66—O30—Na8	112.7 (5)	N36—C184—H18L	109.5
Al7—O30—Na8	85.5 (2)	H18J—C184—H18L	109.5
N11—O31—Al7	105.7 (4)	H18K—C184—H18L	109.5
N11—O31—Na7	115.1 (4)	O76B—C215—N36B	131.8 (14)
Al7—O31—Na7	133.5 (3)	O76B—C215—H215	114.1
N11—O31—Dy2	101.1 (4)	N36B—C215—H215	114.1
Al7—O31—Dy2	94.4 (2)	C215—N36B—C217	130.2 (13)
Na7—O31—Dy2	98.55 (18)	C215—N36B—C216	117.2 (12)
C71—O32—Al7	110.2 (5)	C217—N36B—C216	110.9 (12)
C71—O32—Na8	161.0 (5)	N36B—C216—H21P	109.5
Al7—O32—Na8	88.0 (2)	N36B—C216—H21Q	109.5
C73—O33—Al10	129.5 (6)	H21P—C216—H21Q	109.5
N12—O34—Al7	113.4 (4)	N36B—C216—H21R	109.5
N12—O34—Dy2	121.1 (4)	H21P—C216—H21R	109.5
Al7—O34—Dy2	111.7 (2)	H21Q—C216—H21R	109.5
C78—O35—Al7	112.3 (5)	N36B—C217—H21M	109.5
C78—O35—Na8	136.7 (5)	N36B—C217—H21N	109.5
Al7—O35—Na8	87.0 (2)	H21M—C217—H21N	109.5
C80—O36—Al9	125.0 (5)	N36B—C217—H21O	109.5
C80—O36—Na10	106.4 (5)	H21M—C217—H21O	109.5
Al9—O36—Na10	86.7 (2)	H21N—C217—H21O	109.5
N13—O37—Al12	113.2 (4)	O77—C185—N37	130.9 (18)
N13—O37—Dy2	125.8 (4)	O77—C185—H185	114.6
Al12—O37—Dy2	102.6 (2)	N37—C185—H185	114.6
C85—O38—Al12	113.8 (5)	C185—N37—C187	121.2 (14)
C87—O39—Al8	133.5 (5)	C185—N37—C186	114.9 (14)
N14—O40—Al11	114.1 (4)	C187—N37—C186	123.6 (16)
N14—O40—Na7	101.7 (4)	N37—C186—H18M	109.5
Al11—O40—Na7	112.7 (2)	N37—C186—H18N	109.5
N14—O40—Dy2	116.2 (4)	H18M—C186—H18N	109.5
Al11—O40—Dy2	101.3 (2)	N37—C186—H18O	109.5
Na7—O40—Dy2	111.2 (2)	H18M—C186—H18O	109.5
C92—O41—Na6	134.2 (5)	H18N—C186—H18O	109.5
C92—O41—Na7	97.5 (5)	N37—C187—H18P	109.5
Na6—O41—Na7	96.1 (2)	N37—C187—H18Q	109.5
C94—O42—Al8	153.9 (6)	H18P—C187—H18Q	109.5
N15—O43—Al9	113.8 (4)	N37—C187—H18R	109.5
N15—O43—Dy2	110.1 (4)	H18P—C187—H18R	109.5
Al9—O43—Dy2	121.0 (3)	H18Q—C187—H18R	109.5
C99—O44—Al9	113.2 (5)	O78—C188—N38	125.7 (10)
C99—O44—Na10	148.0 (5)	O78—C188—H188	117.2
Al9—O44—Na10	85.8 (2)	N38—C188—H188	117.2
C101—O45—Al12	132.6 (5)	C188—N38—C190	121.2 (8)
C101—O45—Na9^i^	121.7 (5)	C188—N38—C189	121.0 (9)
Al12—O45—Na9^i^	104.5 (3)	C190—N38—C189	117.8 (9)
N16—O46—Al11	111.3 (4)	N38—C189—H18S	109.5
N16—O46—Dy2	122.3 (4)	N38—C189—H18T	109.5
Al11—O46—Dy2	104.8 (2)	H18S—C189—H18T	109.5
C106—O47—Al11	112.2 (5)	N38—C189—H18U	109.5
C108—O48—Al10	132.8 (5)	H18S—C189—H18U	109.5
N17—O49—Al10	112.9 (4)	H18T—C189—H18U	109.5
N17—O49—Dy2	117.5 (4)	N38—C190—H19A	109.5
Al10—O49—Dy2	126.7 (3)	N38—C190—H19B	109.5
C113—O50—Al10	114.1 (5)	H19A—C190—H19B	109.5
C115—O51—Al9	136.6 (5)	N38—C190—H19C	109.5
C115—O51—Na10	112.5 (5)	H19A—C190—H19C	109.5
Al9—O51—Na10	91.4 (2)	H19B—C190—H19C	109.5
N18—O52—Al12	110.1 (4)	O79—C191—N39	125 (2)
N18—O52—Dy2	105.0 (3)	O79—C191—H191	117.5
Al12—O52—Dy2	99.9 (2)	N39—C191—H191	117.5
C120—O53—Al12	110.0 (4)	C191—N39—C193	126.6 (16)
C120—O53—Na9^i^	113.3 (4)	C191—N39—C192	118.2 (16)
Al12—O53—Na9^i^	100.7 (2)	C193—N39—C192	115.0 (15)
C122—O54—Al11	131.5 (5)	N39—C192—H19D	109.5
C122—O54—Na6	119.2 (4)	N39—C192—H19E	109.5
Al11—O54—Na6	101.0 (2)	H19D—C192—H19E	109.5
C127—O55—Na4	100.1 (5)	N39—C192—H19F	109.5
C127—O55—Na6	143.1 (6)	H19D—C192—H19F	109.5
Na4—O55—Na6	99.9 (2)	H19E—C192—H19F	109.5
C127—O56—Al2	132.9 (5)	N39—C193—H19G	109.5
C127—O56—Na5	125.1 (5)	N39—C193—H19H	109.5
Al2—O56—Na5	101.9 (2)	H19G—C193—H19H	109.5
C127—O56—Na4	84.1 (4)	N39—C193—H19I	109.5
Al2—O56—Na4	97.6 (2)	H19G—C193—H19I	109.5
Na5—O56—Na4	92.3 (2)	H19H—C193—H19I	109.5
C129—O57—Na5	122.9 (5)	O80—C194—N40	126 (2)
C129—O57—Na7	101.9 (5)	O80—C194—H194	117.0
Na5—O57—Na7	135.1 (3)	N40—C194—H194	117.0
C129—O58—Al11	132.6 (5)	C194—N40—C196	118.4 (16)
C129—O58—Na6	119.0 (5)	C194—N40—C195	119.2 (18)
Al11—O58—Na6	108.2 (2)	C196—N40—C195	120.3 (15)
C129—O58—Na7	81.0 (5)	N40—C195—H19J	109.5
Al11—O58—Na7	97.1 (2)	N40—C195—H19K	109.5
Na6—O58—Na7	88.5 (2)	H19J—C195—H19K	109.5
C1—N1—O1	110.9 (6)	N40—C195—H19L	109.5
C1—N1—Al1	132.2 (5)	H19J—C195—H19L	109.5
O1—N1—Al1	116.9 (4)	H19K—C195—H19L	109.5
C8—N2—O4	112.5 (6)	N40—C196—H19M	109.5
C8—N2—Al2	129.4 (5)	N40—C196—H19N	109.5
O4—N2—Al2	117.1 (4)	H19M—C196—H19N	109.5
C15—N3—O7	110.2 (6)	N40—C196—H19O	109.5
C15—N3—Al4	133.0 (5)	H19M—C196—H19O	109.5
O7—N3—Al4	116.5 (4)	H19N—C196—H19O	109.5
C22—N4—O10	112.2 (6)	O81—C197—N41	122 (3)
C22—N4—Al3	132.2 (5)	O81—C197—H197	119.2
O10—N4—Al3	115.6 (4)	N41—C197—H197	119.2
C29—N5—O13	118.1 (6)	C197—N41—C199	125 (2)
C29—N5—H5N	127 (6)	C197—N41—C198	119 (2)
O13—N5—H5N	111 (6)	C199—N41—C198	116 (2)
C36—N6—O16	117.9 (6)	N41—C198—H19P	109.5
C36—N6—Na4	145.8 (5)	N41—C198—H19Q	109.5
O16—N6—Na4	52.1 (3)	H19P—C198—H19Q	109.5
C36—N6—H6N	123 (6)	N41—C198—H19R	109.5
O16—N6—H6N	119 (6)	H19P—C198—H19R	109.5
Na4—N6—H6N	79 (7)	H19Q—C198—H19R	109.5
C43—N7—O19	109.8 (6)	N41—C199—H19S	109.5
C43—N7—Al6	129.8 (6)	N41—C199—H19T	109.5
O19—N7—Al6	120.5 (4)	H19S—C199—H19T	109.5
C50B—N8—O22	112.3 (6)	N41—C199—H19U	109.5
C50—N8—O22	112.3 (6)	H19S—C199—H19U	109.5
C50B—N8—Al5	128.6 (5)	H19T—C199—H19U	109.5
C50—N8—Al5	128.6 (5)	O82—C200—N42	131 (2)
O22—N8—Al5	117.3 (4)	O82—C200—H200	114.4
C57—N9—O25	112.5 (7)	N42—C200—H200	114.4
C57—N9—Al6	131.3 (6)	C200—N42—C202	123.3 (17)
O25—N9—Al6	116.2 (4)	C200—N42—C201	123.6 (17)
C64—N10—O28	112.5 (6)	C202—N42—C201	112.8 (15)
C64—N10—Al7	128.3 (6)	N42—C201—H20D	109.5
O28—N10—Al7	118.0 (4)	N42—C201—H20E	109.5
C71—N11—O31	117.6 (6)	H20D—C201—H20E	109.5
C71—N11—H11N	128 (6)	N42—C201—H20F	109.5
O31—N11—H11N	110 (6)	H20D—C201—H20F	109.5
C78—N12—O34	113.2 (6)	H20E—C201—H20F	109.5
C78—N12—Al9	130.1 (6)	N42—C202—H20A	109.5
O34—N12—Al9	116.7 (4)	N42—C202—H20B	109.5
C85—N13—O37	110.7 (6)	H20A—C202—H20B	109.5
C85—N13—Al8	132.0 (5)	N42—C202—H20C	109.5
O37—N13—Al8	117.3 (4)	H20A—C202—H20C	109.5
C92—N14—O40	121.9 (6)	H20B—C202—H20C	109.5
C92—N14—Na7	77.7 (4)	O82B—C221—N42B	133 (3)
O40—N14—Na7	51.1 (3)	O82B—C221—H221	113.6
C92—N14—H14N	110 (6)	N42B—C221—H221	113.6
O40—N14—H14N	126 (6)	C221—N42B—C223	128 (2)
Na7—N14—H14N	139 (6)	C221—N42B—C222	120 (2)
C99—N15—O43	110.5 (6)	C223—N42B—C222	111.6 (18)
C99—N15—Al12	131.5 (5)	N42B—C222—H22D	109.5
O43—N15—Al12	118.0 (4)	N42B—C222—H22E	109.5
C106—N16—O46	111.5 (6)	H22D—C222—H22E	109.5
C106—N16—Al10	132.3 (5)	N42B—C222—H22F	109.5
O46—N16—Al10	116.1 (4)	H22D—C222—H22F	109.5
C113—N17—O49	109.8 (6)	H22E—C222—H22F	109.5
C113—N17—Al9	129.7 (5)	N42B—C223—H22G	109.5
O49—N17—Al9	120.4 (4)	N42B—C223—H22H	109.5
C120—N18—O52	110.8 (6)	H22G—C223—H22H	109.5
C120—N18—Al11	128.3 (5)	N42B—C223—H22I	109.5
O52—N18—Al11	118.3 (4)	H22G—C223—H22I	109.5
O2—C1—N1	119.9 (7)	H22H—C223—H22I	109.5
			
O27—Al6—O1—N1	95.5 (4)	Al4—O15—C31—C30	−112.2 (8)
O2—Al6—O1—N1	5.5 (4)	C35—C30—C31—O15	−175.1 (7)
N9—Al6—O1—N1	−179.6 (5)	C29—C30—C31—O15	13.9 (12)
N7—Al6—O1—N1	−91.3 (4)	C35—C30—C31—C32	4.3 (12)
Na1—Al6—O1—N1	38.1 (5)	C29—C30—C31—C32	−166.6 (8)
O27—Al6—O1—Dy1	−131.0 (3)	O15—C31—C32—C33	175.7 (9)
O2—Al6—O1—Dy1	139.0 (3)	C30—C31—C32—C33	−3.8 (14)
N9—Al6—O1—Dy1	−46.1 (3)	C31—C32—C33—C34	0.8 (17)
N7—Al6—O1—Dy1	42.2 (3)	C32—C33—C34—C35	1.6 (18)
Na1—Al6—O1—Dy1	171.63 (16)	C33—C34—C35—C30	−1.0 (16)
O21—Al6—O2—C1	169.1 (5)	C31—C30—C35—C34	−1.9 (13)
O27—Al6—O2—C1	−105.7 (5)	C29—C30—C35—C34	169.6 (8)
O1—Al6—O2—C1	−4.7 (5)	Al5—O17—C36—N6	−20.2 (9)
N9—Al6—O2—C1	−24.3 (13)	Na3—O17—C36—N6	164.5 (16)
N7—Al6—O2—C1	82.4 (5)	Al5—O17—C36—C37	158.2 (6)
Na1—Al6—O2—C1	−152.8 (6)	Na3—O17—C36—C37	−17 (3)
O21—Al6—O2—Na1	−38.1 (2)	O16—N6—C36—O17	3.9 (11)
O27—Al6—O2—Na1	47.1 (2)	Na4—N6—C36—O17	−58.8 (12)
O1—Al6—O2—Na1	148.0 (2)	O16—N6—C36—C37	−174.5 (7)
N9—Al6—O2—Na1	128.5 (10)	Na4—N6—C36—C37	122.7 (9)
N7—Al6—O2—Na1	−124.9 (2)	O17—C36—C37—C38	−155.4 (8)
O8—Al1—O3—C3	82.5 (8)	N6—C36—C37—C38	22.9 (13)
O5—Al1—O3—C3	179.0 (8)	O17—C36—C37—C42	21.0 (13)
O4—Al1—O3—C3	−100.4 (8)	N6—C36—C37—C42	−160.6 (9)
N1—Al1—O3—C3	−14.2 (8)	Al3—O18—C38—C37	102.5 (10)
Na2—Al1—O3—C3	−169.2 (9)	Al3—O18—C38—C39	−75.1 (10)
Dy1—Al1—O3—C3	−60.3 (9)	C42—C37—C38—O18	−179.9 (9)
O8—Al1—O3—Na2	−108.3 (3)	C36—C37—C38—O18	−3.5 (14)
O5—Al1—O3—Na2	−11.9 (3)	C42—C37—C38—C39	−2.4 (14)
O4—Al1—O3—Na2	68.7 (3)	C36—C37—C38—C39	174.1 (9)
N1—Al1—O3—Na2	154.9 (3)	O18—C38—C39—C40	−179.7 (10)
Dy1—Al1—O3—Na2	108.9 (3)	C37—C38—C39—C40	2.7 (16)
O11—Al2—O6—C10	−94.9 (7)	C38—C39—C40—C41	−1.1 (19)
O56—Al2—O6—C10	172.8 (6)	C39—C40—C41—C42	−1 (2)
O13—Al2—O6—C10	80.8 (7)	C40—C41—C42—C37	1.2 (19)
N2—Al2—O6—C10	−4.8 (7)	C38—C37—C42—C41	0.5 (16)
Dy1—Al2—O6—C10	47.0 (7)	C36—C37—C42—C41	−176.1 (10)
Na5—Al2—O6—C10	153.2 (7)	Al3—O20—C43—N7	−1.6 (11)
Na4—Al2—O6—C10	126.7 (6)	Al3—O20—C43—C44	179.3 (6)
O11—Al2—O6—Na5	111.8 (2)	O19—N7—C43—O20	0.2 (10)
O56—Al2—O6—Na5	19.6 (2)	Al6—N7—C43—O20	179.8 (6)
O13—Al2—O6—Na5	−72.4 (3)	O19—N7—C43—C44	179.3 (7)
N2—Al2—O6—Na5	−158.0 (2)	Al6—N7—C43—C44	−1.1 (12)
Dy1—Al2—O6—Na5	−106.2 (2)	O20—C43—C44—C49	0.0 (13)
Na4—Al2—O6—Na5	−26.5 (2)	N7—C43—C44—C49	−179.1 (9)
O3—Al1—O8—C15	−168.2 (5)	O20—C43—C44—C45	−179.1 (8)
O5—Al1—O8—C15	102.9 (5)	N7—C43—C44—C45	1.8 (13)
O7—Al1—O8—C15	9.2 (5)	Al6—O21—C45—C44	−4.4 (14)
O4—Al1—O8—C15	19.7 (10)	Na1—O21—C45—C44	−129.8 (8)
N1—Al1—O8—C15	−79.9 (5)	Al6—O21—C45—C46	175.8 (6)
Na2—Al1—O8—C15	150.1 (5)	Na1—O21—C45—C46	50.4 (9)
Dy1—Al1—O8—C15	−14.7 (6)	C49—C44—C45—O21	−178.5 (9)
O15—Al4—O9—C17	−74.4 (8)	C43—C44—C45—O21	0.6 (14)
O23—Al4—O9—C17	−177.5 (7)	C49—C44—C45—C46	1.4 (13)
O22—Al4—O9—C17	99.8 (8)	C43—C44—C45—C46	−179.5 (8)
N3—Al4—O9—C17	18.3 (7)	O21—C45—C46—C47	−178.8 (9)
O6—Al2—O11—C22	175.9 (5)	C44—C45—C46—C47	1.4 (14)
O56—Al2—O11—C22	−96.9 (5)	C45—C46—C47—C48	−1.8 (17)
O10—Al2—O11—C22	−5.9 (5)	C46—C47—C48—C49	−0.5 (18)
O13—Al2—O11—C22	7.4 (10)	C45—C44—C49—C48	−3.6 (15)
N2—Al2—O11—C22	86.0 (5)	C43—C44—C49—C48	177.2 (10)
Dy1—Al2—O11—C22	24.1 (5)	C47—C48—C49—C44	3.2 (17)
Na5—Al2—O11—C22	−138.1 (5)	Al4—O23—C50—N8	0.0 (10)
Na4—Al2—O11—C22	−62.5 (6)	Al4—O23—C50—C51	177.0 (9)
O18—Al3—O12—C24	87.3 (8)	O22—N8—C50—O23	0.3 (10)
O20—Al3—O12—C24	−173.1 (8)	Al5—N8—C50—O23	164.3 (6)
O19—Al3—O12—C24	−91.0 (8)	O22—N8—C50—C51	−176.8 (9)
N4—Al3—O12—C24	−6.8 (8)	Al5—N8—C50—C51	−12.8 (12)
O9—Al4—O15—C31	−96.9 (7)	O23—C50—C51—C56	−1.6 (16)
O23—Al4—O15—C31	0.6 (7)	N8—C50—C51—C56	175.4 (14)
O22—Al4—O15—C31	86.9 (7)	O23—C50—C51—C52	179.3 (12)
N3—Al4—O15—C31	172.1 (6)	N8—C50—C51—C52	−3.7 (15)
O12—Al3—O18—C38	101.8 (7)	Al5—O24—C52—C51	21 (3)
O20—Al3—O18—C38	5.9 (7)	Na3—O24—C52—C51	125.3 (15)
O19—Al3—O18—C38	−79.6 (7)	Al5—O24—C52—C53	−157.8 (14)
N4—Al3—O18—C38	−166.5 (7)	Na3—O24—C52—C53	−54 (2)
O12—Al3—O19—N7	−87.8 (5)	C56—C51—C52—O24	−178.8 (19)
O18—Al3—O19—N7	93.8 (5)	C50—C51—C52—O24	0 (2)
O20—Al3—O19—N7	−1.6 (5)	C56—C51—C52—C53	0 (2)
N4—Al3—O19—N7	−174.1 (5)	C50—C51—C52—C53	179.5 (16)
O12—Al3—O19—Dy1	118.0 (4)	O24—C52—C53—C54	−177.9 (19)
O18—Al3—O19—Dy1	−60.4 (4)	C51—C52—C53—C54	3 (3)
O20—Al3—O19—Dy1	−155.8 (4)	C52—C53—C54—C55	−4 (3)
N4—Al3—O19—Dy1	31.7 (3)	C53—C54—C55—C56	1 (3)
O12—Al3—O20—C43	132.3 (6)	C54—C55—C56—C51	2 (3)
O18—Al3—O20—C43	−111.4 (6)	C52—C51—C56—C55	−3 (3)
O19—Al3—O20—C43	1.8 (6)	C50—C51—C56—C55	177.7 (16)
N4—Al3—O20—C43	35.0 (16)	Al4—O23—C50B—N8	0.0 (10)
O27—Al6—O21—C45	176.1 (8)	Al4—O23—C50B—C51B	−175.1 (9)
O2—Al6—O21—C45	−92.3 (8)	O22—N8—C50B—O23	0.3 (10)
N9—Al6—O21—C45	91.2 (8)	Al5—N8—C50B—O23	164.3 (6)
N7—Al6—O21—C45	4.0 (8)	O22—N8—C50B—C51B	175.3 (10)
Na1—Al6—O21—C45	−132.9 (9)	Al5—N8—C50B—C51B	−20.7 (13)
O27—Al6—O21—Na1	−50.9 (2)	O23—C50B—C51B—C56B	0.7 (18)
O2—Al6—O21—Na1	40.6 (2)	N8—C50B—C51B—C56B	−174.2 (16)
N9—Al6—O21—Na1	−135.8 (2)	O23—C50B—C51B—C52B	179.8 (14)
N7—Al6—O21—Na1	137.0 (3)	N8—C50B—C51B—C52B	4.9 (16)
O15—Al4—O22—N8	−99.3 (5)	Al5—O24—C52B—C53B	−168.5 (16)
O9—Al4—O22—N8	86.4 (6)	Na3—O24—C52B—C53B	−57 (3)
O23—Al4—O22—N8	0.3 (5)	Al5—O24—C52B—C51B	11 (3)
N3—Al4—O22—N8	169.7 (5)	Na3—O24—C52B—C51B	122.1 (19)
O15—Al4—O22—Dy1	70.2 (4)	C56B—C51B—C52B—O24	180 (2)
O9—Al4—O22—Dy1	−104.2 (5)	C50B—C51B—C52B—O24	1 (3)
O23—Al4—O22—Dy1	169.8 (4)	C56B—C51B—C52B—C53B	−1 (3)
N3—Al4—O22—Dy1	−20.8 (4)	C50B—C51B—C52B—C53B	179.9 (19)
O15—Al4—O23—C50B	105.9 (6)	O24—C52B—C53B—C54B	−180 (2)
O9—Al4—O23—C50B	−141.2 (6)	C51B—C52B—C53B—C54B	1 (3)
O22—Al4—O23—C50B	−0.2 (6)	C52B—C53B—C54B—C55B	−2 (4)
N3—Al4—O23—C50B	−41.6 (14)	C53B—C54B—C55B—C56B	3 (4)
O15—Al4—O23—C50	105.9 (6)	C54B—C55B—C56B—C51B	−3 (4)
O9—Al4—O23—C50	−141.2 (6)	C52B—C51B—C56B—C55B	2 (3)
O22—Al4—O23—C50	−0.2 (6)	C50B—C51B—C56B—C55B	−178.6 (19)
N3—Al4—O23—C50	−41.6 (14)	O25—N9—C57—O26	4.5 (10)
O17—Al5—O24—C52	164.5 (15)	Al6—N9—C57—O26	−177.2 (5)
O26—Al5—O24—C52	70.8 (15)	O25—N9—C57—C58	−173.3 (7)
O16—Al5—O24—C52	−114.2 (15)	Al6—N9—C57—C58	5.0 (11)
N8—Al5—O24—C52	−27.8 (15)	Al5—O26—C57—N9	−10.5 (9)
Na3—Al5—O24—C52	115.5 (15)	Na3—O26—C57—N9	100.7 (8)
Dy1—Al5—O24—C52	−70.1 (16)	Al5—O26—C57—C58	167.2 (6)
O17—Al5—O24—C52B	174.0 (19)	Na3—O26—C57—C58	−81.6 (10)
O26—Al5—O24—C52B	80.3 (19)	N9—C57—C58—C63	−166.8 (10)
O16—Al5—O24—C52B	−104.7 (19)	O26—C57—C58—C63	15.5 (14)
N8—Al5—O24—C52B	−18.3 (19)	N9—C57—C58—C59	18.0 (13)
Na3—Al5—O24—C52B	125.0 (19)	O26—C57—C58—C59	−159.7 (8)
Dy1—Al5—O24—C52B	−60.6 (19)	Al6—O27—C59—C60	151.0 (7)
O17—Al5—O24—Na3	48.9 (2)	Na1—O27—C59—C60	52.5 (9)
O26—Al5—O24—Na3	−44.8 (2)	Al6—O27—C59—C58	−31.0 (11)
O16—Al5—O24—Na3	130.3 (2)	Na1—O27—C59—C58	−129.5 (7)
N8—Al5—O24—Na3	−143.3 (2)	C63—C58—C59—O27	179.2 (10)
Dy1—Al5—O24—Na3	174.4 (3)	C57—C58—C59—O27	−5.7 (13)
O17—Al5—O25—N9	−100.7 (4)	C63—C58—C59—C60	−2.8 (14)
O26—Al5—O25—N9	−7.3 (4)	C57—C58—C59—C60	172.3 (8)
O16—Al5—O25—N9	177.1 (4)	O27—C59—C60—C61	179.1 (10)
N8—Al5—O25—N9	90.9 (4)	C58—C59—C60—C61	1.0 (15)
Na3—Al5—O25—N9	−49.2 (5)	C59—C60—C61—C62	3 (2)
Dy1—Al5—O25—N9	139.5 (5)	C60—C61—C62—C63	−5 (2)
O17—Al5—O25—Dy1	119.8 (3)	C61—C62—C63—C58	3 (2)
O26—Al5—O25—Dy1	−146.7 (3)	C59—C58—C63—C62	1 (2)
O16—Al5—O25—Dy1	37.7 (2)	C57—C58—C63—C62	−174.2 (12)
N8—Al5—O25—Dy1	−48.5 (3)	Al8—O29—C64—N10	−5.0 (10)
Na3—Al5—O25—Dy1	171.34 (15)	Al8—O29—C64—C65	177.7 (7)
O21—Al6—O27—C59	−65.2 (6)	O28—N10—C64—O29	2.1 (11)
O2—Al6—O27—C59	−155.7 (6)	Al7—N10—C64—O29	−164.9 (6)
O1—Al6—O27—C59	122.5 (6)	O28—N10—C64—C65	179.5 (7)
N9—Al6—O27—C59	39.3 (6)	Al7—N10—C64—C65	12.5 (12)
N7—Al6—O27—C59	−21.0 (17)	O29—C64—C65—C66	−175.5 (8)
Na1—Al6—O27—C59	−110.6 (7)	N10—C64—C65—C66	7.2 (14)
O21—Al6—O27—Na1	45.5 (2)	O29—C64—C65—C70	3.2 (14)
O2—Al6—O27—Na1	−45.1 (2)	N10—C64—C65—C70	−174.2 (9)
O1—Al6—O27—Na1	−126.9 (2)	Al7—O30—C66—C65	−22.4 (12)
N9—Al6—O27—Na1	149.9 (2)	Na8—O30—C66—C65	−124.5 (8)
N7—Al6—O27—Na1	89.7 (14)	Al7—O30—C66—C67	158.0 (7)
O42—Al8—O29—C64	−102.9 (6)	Na8—O30—C66—C67	55.9 (10)
O39—Al8—O29—C64	146.8 (6)	C70—C65—C66—O30	178.5 (9)
O28—Al8—O29—C64	4.4 (6)	C64—C65—C66—O30	−2.8 (15)
N13—Al8—O29—C64	59.7 (10)	C70—C65—C66—C67	−1.9 (15)
O32—Al7—O30—C66	−162.7 (7)	C64—C65—C66—C67	176.7 (9)
O35—Al7—O30—C66	−69.3 (7)	O30—C66—C67—C68	179.6 (10)
N10—Al7—O30—C66	30.6 (7)	C65—C66—C67—C68	0.0 (16)
O31—Al7—O30—C66	117.3 (7)	C66—C67—C68—C69	2.4 (19)
Na8—Al7—O30—C66	−115.2 (7)	C67—C68—C69—C70	−3 (2)
Dy2—Al7—O30—C66	73.7 (8)	C68—C69—C70—C65	1 (2)
O32—Al7—O30—Na8	−47.5 (2)	C66—C65—C70—C69	1.5 (17)
O35—Al7—O30—Na8	45.8 (2)	C64—C65—C70—C69	−177.1 (11)
N10—Al7—O30—Na8	145.7 (3)	Al7—O32—C71—N11	22.7 (9)
O31—Al7—O30—Na8	−127.5 (2)	Na8—O32—C71—N11	−174.6 (12)
Dy2—Al7—O30—Na8	−171.2 (3)	Al7—O32—C71—C72	−157.8 (6)
O48—Al10—O33—C73	−100.9 (7)	Na8—O32—C71—C72	5 (2)
O50—Al10—O33—C73	−5.7 (7)	O31—N11—C71—O32	−4.6 (11)
O49—Al10—O33—C73	81.7 (7)	O31—N11—C71—C72	175.9 (7)
N16—Al10—O33—C73	168.0 (7)	O32—C71—C72—C73	154.5 (8)
O32—Al7—O34—N12	100.8 (4)	N11—C71—C72—C73	−26.0 (13)
O35—Al7—O34—N12	8.0 (4)	O32—C71—C72—C77	−17.8 (12)
N10—Al7—O34—N12	−91.6 (5)	N11—C71—C72—C77	161.7 (9)
O31—Al7—O34—N12	−178.0 (4)	Al10—O33—C73—C72	−106.6 (9)
Na8—Al7—O34—N12	50.9 (4)	Al10—O33—C73—C74	76.5 (11)
Dy2—Al7—O34—N12	−141.0 (5)	C77—C72—C73—O33	177.8 (9)
O32—Al7—O34—Dy2	−118.2 (3)	C71—C72—C73—O33	5.8 (13)
O35—Al7—O34—Dy2	149.0 (3)	C77—C72—C73—C74	−5.2 (14)
N10—Al7—O34—Dy2	49.4 (3)	C71—C72—C73—C74	−177.2 (9)
O31—Al7—O34—Dy2	−37.0 (2)	O33—C73—C74—C75	179.2 (11)
Na8—Al7—O34—Dy2	−168.15 (15)	C72—C73—C74—C75	2.2 (16)
O51—Al9—O36—C80	62.1 (6)	C73—C74—C75—C76	2 (2)
O44—Al9—O36—C80	153.9 (6)	C74—C75—C76—C77	−3 (2)
O43—Al9—O36—C80	−124.2 (6)	C75—C76—C77—C72	−0.3 (19)
N12—Al9—O36—C80	−41.5 (6)	C73—C72—C77—C76	4.4 (16)
N17—Al9—O36—C80	21.4 (17)	C71—C72—C77—C76	176.6 (10)
Na10—Al9—O36—C80	107.7 (6)	O34—N12—C78—O35	−3.7 (9)
O51—Al9—O36—Na10	−45.6 (2)	Al9—N12—C78—O35	178.2 (5)
O44—Al9—O36—Na10	46.2 (2)	O34—N12—C78—C79	173.5 (6)
O43—Al9—O36—Na10	128.1 (2)	Al9—N12—C78—C79	−4.5 (11)
N12—Al9—O36—Na10	−149.2 (2)	Al7—O35—C78—N12	10.3 (8)
N17—Al9—O36—Na10	−86.3 (14)	Na8—O35—C78—N12	−100.4 (8)
O45—Al12—O38—C85	170.8 (5)	Al7—O35—C78—C79	−166.8 (6)
O53—Al12—O38—C85	−100.6 (5)	Na8—O35—C78—C79	82.5 (9)
O37—Al12—O38—C85	−7.6 (5)	N12—C78—C79—C84	164.9 (9)
O52—Al12—O38—C85	−13.9 (10)	O35—C78—C79—C84	−17.9 (13)
N15—Al12—O38—C85	82.3 (5)	N12—C78—C79—C80	−20.0 (12)
Na9^i^—Al12—O38—C85	−146.4 (5)	O35—C78—C79—C80	157.1 (7)
Dy2—Al12—O38—C85	17.7 (6)	Al9—O36—C80—C81	−149.0 (6)
O42—Al8—O39—C87	97.1 (8)	Na10—O36—C80—C81	−51.5 (8)
O29—Al8—O39—C87	−163.4 (8)	Al9—O36—C80—C79	32.5 (11)
O28—Al8—O39—C87	−85.9 (9)	Na10—O36—C80—C79	130.1 (7)
N13—Al8—O39—C87	−7.2 (8)	C84—C79—C80—O36	−178.5 (9)
O39—Al8—O42—C94	−60.6 (13)	C78—C79—C80—O36	6.4 (12)
O29—Al8—O42—C94	−153.4 (12)	C84—C79—C80—C81	3.0 (13)
O28—Al8—O42—C94	121.4 (12)	C78—C79—C80—C81	−172.0 (8)
N13—Al8—O42—C94	33.7 (13)	O36—C80—C81—C82	177.7 (9)
O36—Al9—O43—N15	−95.5 (4)	C79—C80—C81—C82	−3.8 (14)
O44—Al9—O43—N15	−5.7 (4)	C80—C81—C82—C83	2.6 (18)
N12—Al9—O43—N15	−179.9 (5)	C81—C82—C83—C84	0 (2)
N17—Al9—O43—N15	90.9 (4)	C80—C79—C84—C83	−1.0 (17)
Na10—Al9—O43—N15	−40.5 (5)	C78—C79—C84—C83	174.1 (11)
O36—Al9—O43—Dy2	130.0 (3)	C82—C83—C84—C79	0 (2)
O44—Al9—O43—Dy2	−140.2 (3)	Al12—O38—C85—N13	6.0 (9)
N12—Al9—O43—Dy2	45.5 (3)	Al12—O38—C85—C86	−174.2 (5)
N17—Al9—O43—Dy2	−43.7 (3)	O37—N13—C85—O38	0.8 (9)
Na10—Al9—O43—Dy2	−175.04 (16)	Al8—N13—C85—O38	179.1 (5)
O51—Al9—O44—C99	−167.0 (5)	O37—N13—C85—C86	−179.0 (6)
O36—Al9—O44—C99	107.6 (5)	Al8—N13—C85—C86	−0.6 (11)
O43—Al9—O44—C99	6.3 (5)	O38—C85—C86—C91	−2.5 (11)
N12—Al9—O44—C99	27.9 (12)	N13—C85—C86—C91	177.2 (7)
N17—Al9—O44—C99	−80.7 (5)	O38—C85—C86—C87	177.2 (7)
Na10—Al9—O44—C99	153.3 (6)	N13—C85—C86—C87	−3.0 (11)
O51—Al9—O44—Na10	39.7 (2)	Al8—O39—C87—C86	5.9 (14)
O36—Al9—O44—Na10	−45.7 (2)	Al8—O39—C87—C88	−175.1 (7)
O43—Al9—O44—Na10	−147.1 (2)	C91—C86—C87—O39	−179.5 (8)
N12—Al9—O44—Na10	−125.4 (9)	C85—C86—C87—O39	0.8 (13)
N17—Al9—O44—Na10	126.0 (2)	C91—C86—C87—C88	1.5 (12)
O38—Al12—O45—C101	−86.5 (8)	C85—C86—C87—C88	−178.2 (8)
O53—Al12—O45—C101	176.5 (8)	O39—C87—C88—C89	179.4 (9)
O52—Al12—O45—C101	95.3 (8)	C86—C87—C88—C89	−1.6 (14)
N15—Al12—O45—C101	9.7 (8)	C87—C88—C89—C90	0.1 (16)
Na9^i^—Al12—O45—C101	167.3 (9)	C88—C89—C90—C91	1.5 (15)
Dy2—Al12—O45—C101	54.8 (8)	C87—C86—C91—C90	0.0 (13)
O38—Al12—O45—Na9^i^	106.2 (3)	C85—C86—C91—C90	179.7 (8)
O53—Al12—O45—Na9^i^	9.2 (3)	C89—C90—C91—C86	−1.5 (14)
O52—Al12—O45—Na9^i^	−72.0 (3)	Na6—O41—C92—N14	64.3 (11)
N15—Al12—O45—Na9^i^	−157.5 (3)	Na7—O41—C92—N14	−41.7 (8)
Dy2—Al12—O45—Na9^i^	−112.5 (3)	Na6—O41—C92—C93	−114.5 (8)
O54—Al11—O47—C106	−173.3 (5)	Na7—O41—C92—C93	139.5 (7)
O58—Al11—O47—C106	99.3 (5)	Na6—O41—C92—Na7	106.0 (6)
O46—Al11—O47—C106	7.8 (5)	O40—N14—C92—O41	8.7 (12)
O40—Al11—O47—C106	−4.1 (10)	Na7—N14—C92—O41	35.5 (7)
N18—Al11—O47—C106	−82.9 (5)	O40—N14—C92—C93	−172.4 (6)
Dy2—Al11—O47—C106	−19.8 (5)	Na7—N14—C92—C93	−145.6 (6)
Na6—Al11—O47—C106	138.9 (4)	O40—N14—C92—Na7	−26.8 (6)
Na7—Al11—O47—C106	68.5 (6)	O41—C92—C93—C98	47.6 (11)
O33—Al10—O48—C108	−90.1 (8)	N14—C92—C93—C98	−131.3 (8)
O50—Al10—O48—C108	166.3 (8)	Na7—C92—C93—C98	128.9 (9)
O49—Al10—O48—C108	86.4 (9)	O41—C92—C93—C94	−131.2 (9)
N16—Al10—O48—C108	2.8 (8)	N14—C92—C93—C94	49.9 (10)
O48—Al10—O49—N17	83.9 (6)	Na7—C92—C93—C94	−50.0 (14)
O33—Al10—O49—N17	−99.6 (5)	Al8—O42—C94—C95	72.6 (16)
O50—Al10—O49—N17	1.3 (5)	Al8—O42—C94—C93	−111.6 (13)
N16—Al10—O49—N17	169.2 (5)	C98—C93—C94—O42	−177.0 (8)
O48—Al10—O49—Dy2	−115.9 (4)	C92—C93—C94—O42	1.9 (12)
O33—Al10—O49—Dy2	60.6 (4)	C98—C93—C94—C95	−1.0 (13)
O50—Al10—O49—Dy2	161.4 (4)	C92—C93—C94—C95	177.8 (8)
N16—Al10—O49—Dy2	−30.6 (3)	O42—C94—C95—C96	179.0 (11)
O48—Al10—O50—C113	−138.9 (6)	C93—C94—C95—C96	3.0 (16)
O33—Al10—O50—C113	108.6 (6)	C94—C95—C96—C97	−3 (2)
O49—Al10—O50—C113	−1.3 (6)	C95—C96—C97—C98	2 (2)
N16—Al10—O50—C113	−48.6 (13)	C96—C97—C98—C93	0.3 (18)
O36—Al9—O51—C115	174.3 (8)	C94—C93—C98—C97	−0.6 (15)
O44—Al9—O51—C115	83.0 (8)	C92—C93—C98—C97	−179.4 (10)
N12—Al9—O51—C115	−101.1 (8)	Al9—O44—C99—N15	−6.0 (9)
N17—Al9—O51—C115	−13.1 (8)	Na10—O44—C99—N15	116.3 (9)
Na10—Al9—O51—C115	125.6 (8)	Al9—O44—C99—C100	174.8 (6)
O36—Al9—O51—Na10	48.7 (2)	Na10—O44—C99—C100	−62.9 (12)
O44—Al9—O51—Na10	−42.6 (2)	O43—N15—C99—O44	1.3 (9)
N12—Al9—O51—Na10	133.3 (2)	Al12—N15—C99—O44	179.0 (5)
N17—Al9—O51—Na10	−138.7 (2)	O43—N15—C99—C100	−179.6 (6)
O45—Al12—O53—C120	−128.8 (5)	Al12—N15—C99—C100	−1.8 (11)
O38—Al12—O53—C120	137.4 (5)	O44—C99—C100—C105	2.8 (12)
O37—Al12—O53—C120	56.2 (5)	N15—C99—C100—C105	−176.3 (8)
O52—Al12—O53—C120	−21.2 (5)	O44—C99—C100—C101	−175.5 (8)
N15—Al12—O53—C120	−54.8 (12)	N15—C99—C100—C101	5.3 (12)
Na9^i^—Al12—O53—C120	−119.9 (5)	Al12—O45—C101—C102	171.1 (7)
Dy2—Al12—O53—C120	15.0 (5)	Na9^i^—O45—C101—C102	−23.4 (12)
O45—Al12—O53—Na9^i^	−8.9 (2)	Al12—O45—C101—C100	−9.2 (14)
O38—Al12—O53—Na9^i^	−102.7 (2)	Na9^i^—O45—C101—C100	156.2 (7)
O37—Al12—O53—Na9^i^	176.1 (2)	C105—C100—C101—O45	−178.7 (9)
O52—Al12—O53—Na9^i^	98.7 (2)	C99—C100—C101—O45	−0.4 (14)
N15—Al12—O53—Na9^i^	65.0 (11)	C105—C100—C101—C102	1.0 (15)
Dy2—Al12—O53—Na9^i^	134.89 (15)	C99—C100—C101—C102	179.3 (9)
O47—Al11—O54—C122	102.4 (6)	O45—C101—C102—C103	178.2 (12)
O58—Al11—O54—C122	−161.6 (6)	C100—C101—C102—C103	−1.5 (18)
O40—Al11—O54—C122	−73.8 (6)	C101—C102—C103—C104	0 (2)
N18—Al11—O54—C122	10.4 (6)	C102—C103—C104—C105	2 (2)
Dy2—Al11—O54—C122	−40.5 (7)	C103—C104—C105—C100	−2 (2)
Na6—Al11—O54—C122	−146.7 (7)	C101—C100—C105—C104	0.8 (17)
Na7—Al11—O54—C122	−112.0 (6)	C99—C100—C105—C104	−177.5 (11)
O47—Al11—O54—Na6	−110.8 (2)	Al11—O47—C106—N16	−6.2 (9)
O58—Al11—O54—Na6	−14.9 (2)	Al11—O47—C106—C107	177.1 (5)
O40—Al11—O54—Na6	73.0 (2)	O46—N16—C106—O47	−0.6 (9)
N18—Al11—O54—Na6	157.1 (2)	Al10—N16—C106—O47	−178.6 (5)
Dy2—Al11—O54—Na6	106.2 (2)	O46—N16—C106—C107	176.2 (6)
Na7—Al11—O54—Na6	34.7 (2)	Al10—N16—C106—C107	−1.9 (11)
O54—Al11—O58—C129	−167.9 (8)	O47—C106—C107—C112	0.7 (11)
O47—Al11—O58—C129	−71.5 (8)	N16—C106—C107—C112	−176.2 (7)
O46—Al11—O58—C129	11.7 (8)	O47—C106—C107—C108	178.2 (7)
O40—Al11—O58—C129	88.9 (7)	N16—C106—C107—C108	1.4 (11)
Dy2—Al11—O58—C129	50.2 (8)	Al10—O48—C108—C109	174.1 (7)
Na6—Al11—O58—C129	175.0 (9)	Al10—O48—C108—C107	−3.9 (13)
Na7—Al11—O58—C129	84.2 (7)	C112—C107—C108—O48	178.8 (8)
O54—Al11—O58—Na6	17.1 (3)	C106—C107—C108—O48	1.3 (13)
O47—Al11—O58—Na6	113.5 (3)	C112—C107—C108—C109	0.8 (12)
O46—Al11—O58—Na6	−163.3 (3)	C106—C107—C108—C109	−176.7 (8)
O40—Al11—O58—Na6	−86.1 (3)	O48—C108—C109—C110	−178.9 (9)
Dy2—Al11—O58—Na6	−124.81 (19)	C107—C108—C109—C110	−0.8 (13)
Na7—Al11—O58—Na6	−90.8 (3)	C108—C109—C110—C111	1.1 (15)
O54—Al11—O58—Na7	107.8 (2)	C109—C110—C111—C112	−1.2 (14)
O47—Al11—O58—Na7	−155.8 (2)	C110—C111—C112—C107	1.2 (13)
O46—Al11—O58—Na7	−72.5 (2)	C108—C107—C112—C111	−1.0 (13)
O40—Al11—O58—Na7	4.6 (2)	C106—C107—C112—C111	176.6 (8)
Dy2—Al11—O58—Na7	−34.0 (2)	O49—N17—C113—O50	−0.1 (10)
Na6—Al11—O58—Na7	90.8 (3)	Al9—N17—C113—O50	−178.2 (5)
Al6—O1—N1—C1	−5.3 (7)	O49—N17—C113—C114	−178.8 (7)
Dy1—O1—N1—C1	−143.4 (5)	Al9—N17—C113—C114	3.1 (11)
Al6—O1—N1—Al1	174.2 (3)	Al10—O50—C113—N17	1.1 (10)
Dy1—O1—N1—Al1	36.1 (5)	Al10—O50—C113—C114	179.9 (6)
Al1—O4—N2—C8	18.6 (6)	N17—C113—C114—C119	172.9 (8)
Dy1—O4—N2—C8	123.8 (5)	O50—C113—C114—C119	−5.8 (12)
Al1—O4—N2—Al2	−150.9 (3)	N17—C113—C114—C115	−8.8 (12)
Dy1—O4—N2—Al2	−45.8 (4)	O50—C113—C114—C115	172.5 (7)
Al1—O7—N3—C15	10.6 (7)	Al9—O51—C115—C116	−170.4 (6)
Dy1—O7—N3—C15	137.3 (5)	Na10—O51—C115—C116	−52.0 (9)
Al1—O7—N3—Al4	−174.8 (3)	Al9—O51—C115—C114	11.2 (13)
Dy1—O7—N3—Al4	−48.1 (6)	Na10—O51—C115—C114	129.6 (7)
Al2—O10—N4—C22	−6.4 (7)	Al9—O51—C115—Na10	−118.4 (8)
Dy1—O10—N4—C22	−128.4 (5)	C119—C114—C115—O51	−179.0 (8)
Al2—O10—N4—Al3	174.1 (3)	C113—C114—C115—O51	2.7 (12)
Dy1—O10—N4—Al3	52.2 (6)	C119—C114—C115—C116	2.6 (12)
Al2—O13—N5—C29	98.9 (6)	C113—C114—C115—C116	−175.8 (8)
Dy1—O13—N5—C29	−136.0 (5)	C119—C114—C115—Na10	−121.0 (9)
Na4—O13—N5—C29	−15.8 (7)	C113—C114—C115—Na10	60.7 (12)
Al5—O16—N6—C36	13.8 (8)	O51—C115—C116—C117	177.6 (8)
Na4—O16—N6—C36	−140.7 (6)	C114—C115—C116—C117	−3.8 (13)
Dy1—O16—N6—C36	112.5 (6)	Na10—C115—C116—C117	143.7 (9)
Al5—O16—N6—Na4	154.5 (4)	C115—C116—C117—C118	1.6 (15)
Dy1—O16—N6—Na4	−106.8 (3)	C116—C117—C118—C119	2.0 (16)
Al3—O19—N7—C43	1.2 (7)	C115—C114—C119—C118	0.9 (14)
Dy1—O19—N7—C43	157.8 (5)	C113—C114—C119—C118	179.3 (9)
Al3—O19—N7—Al6	−178.4 (3)	C117—C118—C119—C114	−3.3 (15)
Dy1—O19—N7—Al6	−21.8 (6)	Al12—O53—C120—N18	19.2 (8)
Al4—O22—N8—C50B	−0.4 (7)	Na9^i^—O53—C120—N18	−92.7 (7)
Dy1—O22—N8—C50B	−171.8 (5)	Al12—O53—C120—C121	−159.5 (5)
Al4—O22—N8—C50	−0.4 (7)	Na9^i^—O53—C120—C121	88.6 (7)
Dy1—O22—N8—C50	−171.8 (5)	O52—N18—C120—O53	−2.5 (9)
Al4—O22—N8—Al5	−166.4 (3)	Al11—N18—C120—O53	−164.0 (5)
Dy1—O22—N8—Al5	22.3 (6)	O52—N18—C120—C121	176.2 (6)
Al5—O25—N9—C57	3.9 (7)	Al11—N18—C120—C121	14.7 (10)
Dy1—O25—N9—C57	139.4 (5)	O53—C120—C121—C122	174.3 (6)
Al5—O25—N9—Al6	−174.7 (3)	N18—C120—C121—C122	−4.4 (11)
Dy1—O25—N9—Al6	−39.2 (6)	O53—C120—C121—C126	−2.5 (10)
Al8—O28—N10—C64	1.6 (8)	N18—C120—C121—C126	178.7 (7)
Dy2—O28—N10—C64	178.7 (5)	Al11—O54—C122—C121	−4.9 (11)
Al8—O28—N10—Al7	170.1 (3)	Na6—O54—C122—C121	−146.8 (6)
Dy2—O28—N10—Al7	−12.8 (6)	Al11—O54—C122—C123	173.4 (5)
Al7—O31—N11—C71	−14.9 (8)	Na6—O54—C122—C123	31.4 (8)
Na7—O31—N11—C71	142.3 (6)	C126—C121—C122—O54	175.6 (7)
Dy2—O31—N11—C71	−112.7 (6)	C120—C121—C122—O54	−1.1 (11)
Al7—O34—N12—C78	−4.8 (7)	C126—C121—C122—C123	−2.5 (11)
Dy2—O34—N12—C78	−141.8 (5)	C120—C121—C122—C123	−179.3 (7)
Al7—O34—N12—Al9	173.5 (3)	O54—C122—C123—C124	−175.2 (7)
Dy2—O34—N12—Al9	36.5 (6)	C121—C122—C123—C124	3.1 (11)
Al12—O37—N13—C85	−7.1 (7)	C122—C123—C124—C125	−1.7 (13)
Dy2—O37—N13—C85	−134.1 (5)	C123—C124—C125—C126	−0.5 (14)
Al12—O37—N13—Al8	174.3 (3)	C124—C125—C126—C121	1.1 (13)
Dy2—O37—N13—Al8	47.3 (6)	C122—C121—C126—C125	0.5 (12)
Al11—O40—N14—C92	−87.1 (7)	C120—C121—C126—C125	177.4 (7)
Na7—O40—N14—C92	34.5 (7)	Na4—O55—C127—O56	−9.3 (9)
Dy2—O40—N14—C92	155.4 (6)	Na6—O55—C127—O56	112.5 (9)
Al11—O40—N14—Na7	−121.6 (4)	Na4—O55—C127—C128	173.4 (7)
Dy2—O40—N14—Na7	121.0 (4)	Na6—O55—C127—C128	−64.8 (12)
Al9—O43—N15—C99	4.1 (7)	Na6—O55—C127—Na4	121.8 (9)
Dy2—O43—N15—C99	143.5 (5)	Al2—O56—C127—O55	102.9 (9)
Al9—O43—N15—Al12	−174.0 (3)	Na5—O56—C127—O55	−80.5 (9)
Dy2—O43—N15—Al12	−34.6 (5)	Na4—O56—C127—O55	8.1 (8)
Al11—O46—N16—C106	6.9 (7)	Al2—O56—C127—C128	−79.7 (10)
Dy2—O46—N16—C106	131.6 (5)	Na5—O56—C127—C128	96.8 (9)
Al11—O46—N16—Al10	−174.7 (3)	Na4—O56—C127—C128	−174.6 (8)
Dy2—O46—N16—Al10	−50.0 (6)	Al2—O56—C127—Na4	94.8 (6)
Al10—O49—N17—C113	−1.0 (7)	Na5—O56—C127—Na4	−88.6 (4)
Dy2—O49—N17—C113	−163.1 (5)	Na5—O57—C129—O58	−149.1 (6)
Al10—O49—N17—Al9	177.3 (3)	Na7—O57—C129—O58	27.6 (8)
Dy2—O49—N17—Al9	15.2 (6)	Na5—O57—C129—C130	30.9 (10)
Al12—O52—N18—C120	−15.3 (6)	Na7—O57—C129—C130	−152.4 (7)
Dy2—O52—N18—C120	−122.0 (5)	Na5—O57—C129—Na7	−176.7 (6)
Al12—O52—N18—Al11	148.3 (3)	Al11—O58—C129—O57	−114.2 (8)
Dy2—O52—N18—Al11	41.6 (5)	Na6—O58—C129—O57	60.4 (9)
Al6—O2—C1—N1	3.1 (9)	Na7—O58—C129—O57	−22.8 (7)
Na1—O2—C1—N1	−121.2 (8)	Al11—O58—C129—C130	65.8 (10)
Al6—O2—C1—C2	−177.1 (6)	Na6—O58—C129—C130	−119.6 (7)
Na1—O2—C1—C2	58.6 (12)	Na7—O58—C129—C130	157.2 (7)
O1—N1—C1—O2	1.5 (10)	Al11—O58—C129—Na7	−91.4 (6)
Al1—N1—C1—O2	−177.9 (5)	Na6—O58—C129—Na7	83.2 (4)
O1—N1—C1—C2	−178.4 (7)	Na10^ii^—O59—C131—N19	−109.0 (18)
Al1—N1—C1—C2	2.2 (12)	Na1—O59—C131—N19	96.1 (17)
O2—C1—C2—C3	173.9 (8)	O59—C131—N19—C133	−171.9 (16)
N1—C1—C2—C3	−6.3 (13)	O59—C131—N19—C132	−7 (3)
O2—C1—C2—C7	−3.2 (13)	Na1—O60—C134—N20	−177.9 (13)
N1—C1—C2—C7	176.7 (8)	O60—C134—N20—C136	175.0 (17)
Al1—O3—C3—C4	−165.6 (7)	O60—C134—N20—C135	9 (3)
Na2—O3—C3—C4	26.5 (11)	Na1—O60B—C224—N20B	130 (7)
Al1—O3—C3—C2	14.4 (13)	O60B—C224—N20B—C226	−132 (10)
Na2—O3—C3—C2	−153.5 (7)	O60B—C224—N20B—C225	35 (14)
C7—C2—C3—O3	175.9 (9)	Na2—O61—C137—N21	−125.6 (9)
C1—C2—C3—O3	−1.1 (14)	O61—C137—N21—C138	4.3 (17)
C7—C2—C3—C4	−4.1 (14)	O61—C137—N21—C139	175.1 (10)
C1—C2—C3—C4	178.9 (9)	Na3^iii^—O62—C140—N22	54.3 (19)
O3—C3—C4—C5	−177.3 (11)	Na2—O62—C140—N22	178.2 (9)
C2—C3—C4—C5	2.7 (17)	O62—C140—N22—C141	−9 (2)
C3—C4—C5—C6	1 (2)	O62—C140—N22—C142	−179.4 (13)
C4—C5—C6—C7	−3 (2)	Na3^iii^—O63—C143—N23	163.2 (11)
C5—C6—C7—C2	2.0 (19)	Na2—O63—C143—N23	−31 (3)
C3—C2—C7—C6	1.7 (16)	O63—C143—N23—C144	−2 (2)
C1—C2—C7—C6	178.9 (10)	O63—C143—N23—C145	171.6 (14)
Al1—O5—C8—N2	−20.0 (8)	Na3^iii^—O63B—C203—N23B	−15 (10)
Na2—O5—C8—N2	90.1 (7)	Na2—O63B—C203—N23B	176 (4)
Al1—O5—C8—C9	158.2 (5)	Na3^iii^—O63B—C203—Na2	170 (8)
Na2—O5—C8—C9	−91.7 (7)	O63B—C203—N23B—C205	177 (5)
O4—N2—C8—O5	0.5 (9)	Na2—C203—N23B—C205	−171 (9)
Al2—N2—C8—O5	168.4 (5)	O63B—C203—N23B—C204	−16 (8)
O4—N2—C8—C9	−177.8 (6)	Na2—C203—N23B—C204	−4 (18)
Al2—N2—C8—C9	−9.8 (10)	Na3^iii^—O64—C146—N24	−37 (9)
O5—C8—C9—C14	5.0 (10)	Na2—O64—C146—N24	174.5 (16)
N2—C8—C9—C14	−176.8 (7)	O64—C146—N24—C147	3 (4)
O5—C8—C9—C10	−174.1 (7)	O64—C146—N24—C148	176 (3)
N2—C8—C9—C10	4.2 (11)	Na3^iii^—O64B—C230—N24B	54 (15)
Al2—O6—C10—C11	−178.5 (5)	Na2—O64B—C230—N24B	173 (3)
Na5—O6—C10—C11	−28.8 (8)	O64B—C230—N24B—C232	−159 (7)
Al2—O6—C10—C9	1.7 (11)	O64B—C230—N24B—C231	7 (8)
Na5—O6—C10—C9	151.5 (6)	Na4—O65—C149—N25	−6 (2)
C14—C9—C10—O6	−178.9 (7)	Na6—O65—C149—N25	−170.1 (9)
C8—C9—C10—O6	0.1 (11)	O65—C149—N25—C151	−174.4 (12)
C14—C9—C10—C11	1.4 (11)	O65—C149—N25—C150	0.2 (18)
C8—C9—C10—C11	−179.6 (7)	Na5—O66—C152—N26	−86.2 (14)
O6—C10—C11—C12	178.7 (7)	O66—C152—N26—C153	2.3 (18)
C9—C10—C11—C12	−1.6 (11)	O66—C152—N26—C154	179.8 (12)
C10—C11—C12—C13	1.0 (13)	Na5—O67—C155—N27	−128.9 (10)
C11—C12—C13—C14	−0.2 (14)	O67—C155—N27—C156	−1.3 (18)
C12—C13—C14—C9	0.0 (14)	O67—C155—N27—C157	−178.1 (12)
C10—C9—C14—C13	−0.6 (12)	Na6—O68—C158—N28	45.2 (16)
C8—C9—C14—C13	−179.7 (8)	O68—C158—N28—C160	−173.0 (11)
Al1—O8—C15—N3	−5.9 (9)	O68—C158—N28—C159	2.8 (17)
Al1—O8—C15—C16	177.2 (6)	Na7—O69—C161—N29	159.7 (13)
O7—N3—C15—O8	−3.1 (9)	O69—C161—N29—C163	−176.4 (16)
Al4—N3—C15—O8	−176.6 (5)	O69—C161—N29—C162	−10 (3)
O7—N3—C15—C16	173.8 (6)	Na7—O69B—C206—N29B	−121 (7)
Al4—N3—C15—C16	0.3 (11)	O69B—C206—N29B—C208	179 (7)
O8—C15—C16—C21	4.6 (11)	O69B—C206—N29B—C207	11 (12)
N3—C15—C16—C21	−172.3 (7)	Na8—O70—C164—N30	46 (3)
O8—C15—C16—C17	−177.7 (7)	Na9—O70—C164—N30	−173.0 (15)
N3—C15—C16—C17	5.4 (11)	O70—C164—N30—C166	−179 (2)
Al4—O9—C17—C16	−18.4 (12)	O70—C164—N30—C165	5 (3)
Al4—O9—C17—C18	163.0 (7)	Na8—O70B—C209—N30B	−86 (5)
C21—C16—C17—O9	−179.4 (8)	Na9—O70B—C209—N30B	175 (4)
C15—C16—C17—O9	3.0 (13)	Na8—O70B—C209—Na9	99 (2)
C21—C16—C17—C18	−0.8 (12)	O70B—C209—N30B—C211	173 (4)
C15—C16—C17—C18	−178.4 (8)	O70B—C209—N30B—C210	5 (7)
O9—C17—C18—C19	−180.0 (9)	Na8—O71—C167—N31	−46.8 (16)
C16—C17—C18—C19	1.3 (14)	Na9—O71—C167—N31	−172.4 (8)
C17—C18—C19—C20	−0.4 (16)	O71—C167—N31—C168	4.5 (17)
C18—C19—C20—C21	−1.2 (16)	O71—C167—N31—C169	−179.5 (11)
C19—C20—C21—C16	1.7 (14)	Na8—O72—C170—N32	−158.6 (7)
C17—C16—C21—C20	−0.7 (13)	Na9—O72—C170—N32	29 (2)
C15—C16—C21—C20	177.0 (8)	O72—C170—N32—C172	−175.8 (10)
Al2—O11—C22—N4	4.0 (9)	O72—C170—N32—C171	2.4 (15)
Al2—O11—C22—C23	−177.9 (5)	Na9^i^—O73—C173—N33	131.2 (9)
O10—N4—C22—O11	1.7 (10)	O73—C173—N33—C175	−173.7 (10)
Al3—N4—C22—O11	−178.9 (5)	O73—C173—N33—C174	−4.0 (18)
O10—N4—C22—C23	−176.4 (6)	Na10—O74—C176—N34	−123 (2)
Al3—N4—C22—C23	3.0 (11)	O74—C176—N34—C177	2 (2)
O11—C22—C23—C28	−6.8 (12)	O74—C176—N34—C178	178.0 (15)
N4—C22—C23—C28	171.3 (8)	Na10—O74B—C212—N34B	156 (5)
O11—C22—C23—C24	175.0 (7)	O74B—C212—N34B—C214	−178 (7)
N4—C22—C23—C24	−6.9 (12)	Na10—C212—N34B—C214	−146 (7)
Al3—O12—C24—C23	4.7 (14)	O74B—C212—N34B—C213	−28 (10)
Al3—O12—C24—C25	−173.0 (7)	Na10—C212—N34B—C213	3 (12)
C28—C23—C24—O12	−174.7 (9)	Na1^iv^—O75—C179—N35	109.2 (19)
C22—C23—C24—O12	3.5 (13)	Na10—O75—C179—N35	−95.0 (17)
C28—C23—C24—C25	3.0 (13)	Na1^iv^—O75—C179—Na10	−155.8 (17)
C22—C23—C24—C25	−178.8 (8)	O75—C179—N35—C181	171.9 (15)
O12—C24—C25—C26	174.5 (9)	Na10—C179—N35—C181	119.7 (16)
C23—C24—C25—C26	−3.4 (14)	O75—C179—N35—C180	1 (3)
C24—C25—C26—C27	1.5 (16)	Na10—C179—N35—C180	−51.4 (18)
C25—C26—C27—C28	1.0 (16)	O76—C182—N36—C183	−28 (5)
C26—C27—C28—C23	−1.3 (15)	O76—C182—N36—C184	173 (3)
C24—C23—C28—C27	−0.7 (14)	O76B—C215—N36B—C217	−167 (7)
C22—C23—C28—C27	−178.9 (9)	O76B—C215—N36B—C216	−4 (7)
Na4—O14—C29—N5	46.9 (8)	O77—C185—N37—C187	−176.2 (18)
Na5—O14—C29—N5	−64.8 (8)	O77—C185—N37—C186	−2 (3)
Na4—O14—C29—C30	−131.8 (6)	O78—C188—N38—C190	−176.6 (13)
Na5—O14—C29—C30	116.5 (6)	O78—C188—N38—C189	0 (2)
Na5—O14—C29—Na4	−111.7 (5)	O79—C191—N39—C193	172.1 (19)
O13—N5—C29—O14	−20.0 (10)	O79—C191—N39—C192	−3 (3)
O13—N5—C29—C30	158.7 (6)	O80—C194—N40—C196	−20 (4)
O13—N5—C29—Na4	12.9 (5)	O80—C194—N40—C195	176 (2)
O14—C29—C30—C31	156.4 (7)	O81—C197—N41—C199	−157 (3)
N5—C29—C30—C31	−22.3 (11)	O81—C197—N41—C198	27 (5)
Na4—C29—C30—C31	95.7 (9)	O82—C200—N42—C202	−16 (5)
O14—C29—C30—C35	−14.7 (11)	O82—C200—N42—C201	170 (3)
N5—C29—C30—C35	166.6 (7)	O82B—C221—N42B—C223	17 (11)
Na4—C29—C30—C35	−75.4 (10)	O82B—C221—N42B—C222	−173 (6)
Al4—O15—C31—C32	68.4 (10)		

Symmetry codes: (i) *x*, *y*−1, *z*; (ii) *x*+1, *y*, *z*; (iii) *x*, *y*+1, *z*; (iv) *x*−1, *y*, *z*.

### Poly[[di-µ4-acetato-nonakis(µ-N,N-dimethylformamide)octakis(N,N-dimethylformamide)tetrakis[salicylhydroximato(2-)]tetradecakis[salicylhydroximato(3-)]dodecaaluminium(III)didysprosium(III)decasodium(I)] N,N-dimethylformamide 6.335-solvate] (2) Hydrogen-bond geometry (Å, º)

**Table:**

*D*—H···*A*	*D*—H	H···*A*	*D*···*A*	*D*—H···*A*
N5—H5*N*···O7	0.89 (3)	2.36 (7)	3.059 (8)	136 (7)
N6—H6*N*···O10	0.87 (3)	2.46 (8)	3.004 (9)	121 (7)
N11—H11*N*···O46	0.87 (3)	2.27 (6)	3.003 (8)	142 (8)
N14—H14*N*···O37	0.89 (3)	2.22 (8)	2.795 (8)	122 (7)
C4—H4···O62	0.95	2.64	3.477 (11)	147
C60—H60···O74^ii^	0.95	2.55	3.287 (19)	135
C81—H81···O60^iv^	0.95	2.59	3.293 (12)	131
C123—H123···O68	0.95	2.48	3.333 (11)	150
C128—H12*C*···O69*B*	0.98	2.39	3.14 (5)	133
C130—H13*B*···O14	0.98	2.40	3.251 (10)	145
C137—H137···O4	0.95	2.36	3.221 (10)	151
C139—H13*T*···O19	0.98	2.53	3.459 (13)	159
C140—H140···O61	0.95	2.41	3.137 (11)	134
C144—H14*I*···O61	0.98	2.65	3.599 (15)	164
C153—H15*G*···O11	0.98	2.60	3.450 (13)	146
C157—H15*P*···O77^v^	0.98	2.35	3.241 (19)	150
C159—H15*S*···O78^vi^	0.98	2.38	3.332 (14)	165
C159—H15*U*···O41	0.98	2.51	3.486 (13)	175
C162—H16*F*···O29	0.98	2.60	3.343 (15)	132
C163—H16*G*···O48^vi^	0.98	2.57	3.540 (14)	170
C168—H16*P*···O51	0.98	2.63	3.388 (12)	135
C169—H16*T*···O80^v^	0.98	2.65	3.55 (2)	153
C171—H17*C*···O73^iii^	0.98	2.42	3.360 (12)	161
C172—H17*E*···O66	0.98	2.61	3.518 (14)	154
C173—H173···O52	0.95	2.37	3.254 (9)	154
C175—H17*J*···O49	0.98	2.58	3.434 (13)	146
C183—H18*H*···O9	0.98	2.64	3.60 (3)	166
C183—H18*H*···O23	0.98	2.43	3.15 (3)	130
C184—H18*K*···O9	0.98	2.64	3.56 (2)	156
C191—H191···O77^vii^	0.95	2.64	3.48 (3)	148
C199—H19*T*···O29	0.98	2.52	3.50 (4)	179
C199—H19*U*···O36	0.98	2.59	3.39 (3)	139
C201—H20*D*···O15	0.98	2.50	3.30 (3)	139
C204—H20*G*···O68^iii^	0.98	2.26	3.21 (5)	163
C223—H22*G*···O15	0.98	2.42	3.25 (5)	142

Symmetry codes: (ii) *x*+1, *y*, *z*; (iii) *x*, *y*+1, *z*; (iv) *x*−1, *y*, *z*; (v) *x*, −*y*+1, *z*+1/2; (vi) *x*, −*y*, *z*−1/2; (vii) *x*, −*y*, *z*+1/2.
